# Meplazumab in hospitalized adults with severe COVID-19 (DEFLECT): a multicenter, seamless phase 2/3, randomized, third-party double-blind clinical trial

**DOI:** 10.1038/s41392-023-01323-9

**Published:** 2023-01-30

**Authors:** Huijie Bian, Liang Chen, Zhao-Hui Zheng, Xiu-Xuan Sun, Jie-Jie Geng, Ruo Chen, Ke Wang, Xu Yang, Shi-Rui Chen, Si-Yu Chen, Rong-Hua Xie, Kui Zhang, Jin-Lin Miao, Jun-Feng Jia, Hao Tang, Shuang-Shuang Liu, Hong-Wei Shi, Yong Yang, Xiao-Chun Chen, Vinay Malhotra, Nosheen Nasir, Iffat Khanum, Faisal Mahmood, Saeed Hamid, Claudio Marcel Berdun Stadnik, Kengi Itinose, Caroline Cândida Carvalho de Oliveira, Cesar Dusilek, Lucas Rivabem, Adilson Joaquim Westheimer Cavalcante, Suzara Souto Lopes, Wladmir Faustino Saporito, Fábio José Concilio Fucci, Jesus Abraham Simon-Campos, Ling Wang, Lin-Na Liu, Qing-Yi Wang, Ding Wei, Zheng Zhang, Zhi-Nan Chen, Ping Zhu

**Affiliations:** 1 Department of Cell Biology of National Translational Science Center for Molecular Medicine and Department of Clinical Immunology of Xijing Hospital, Fourth Military Medical University, Xi’an, China; 2 Shanghai Engineering Research Center of Organ Repair, School of Medicine, Shanghai University, Shanghai, China; 3 Jiangsu Pacific Meinuoke Biopharmaceutical Co. Ltd, Changzhou, China; 4 Pulse Heart Institute Cardiology Services, MultiCare Institute for Research & Innovation, Tacoma, WA USA; 5 Department of Medicine, The Aga Khan University, Karachi, Pakistan; 6 Infection Control Service, Irmandade Santa Casa de Misericord de Porto Alegre, Porto Alegre, Brazil; 7 Clinical Research Department, Hospital do Rocio, Campo Largo, Brazil; 8 CEMEC-Centro Multidisciplinar de Estudos Clínicos, São Bernardo do Campo, Brazil; 9 Chronos Pesquisa Clinica, Brasília, Brazil; 10 Pesquisare Saúde, Santo André, Brazil; 11 Instituto de Moléstias Cardiovasculares Tatuí, Tatuí, Brazil; 12 Kohler & Milstein Research /Hospital Agustín O’Horán, Mérida, Mexico; 13 College of Military Preventive Medicine, Fourth Military Medical University, Xi’an, China; 14 Department of Pharmaceutics, Tangdu Hospital, Fourth Military Medical University, Xi’an, China; 15 Department of Foreign Languages, Fourth Military Medical University, Xi’an, China

**Keywords:** Molecular medicine, Drug development

## Abstract

Meplazumab, a humanized CD147 antibody, has shown favourable safety and efficacy in our previous clinical studies. In DEFLECT (NCT04586153), 167 patients with severe COVID-19 were enroled and randomized to receive three dosages of meplazumab and a placebo. Meplazumab at 0.12 mg/kg, compared to the placebo group, showed clinical benefits in significantly reducing mortality by 83.6% (2.4% vs. 14.6%, *p* = 0.0150), increasing the proportion of patients alive and discharged without supplemental oxygen (82.9% vs. 70.7%, *p* = 0.0337) and increasing the proportion of patients who achieved sustained clinical improvement (41.5% vs. 31.7%). The response rate in the 0.2 mg/kg group was relatively increased by 16.0% compared with the placebo group (53.7% vs. 46.3%). Meplazumab also reduced the viral loads and multiple cytokine levels. Compare with the placebo group, the 0.3 mg/kg significantly increased the virus negative rate by 40.6% (*p* = 0.0363) and reduced IL-8 level (*p* = 0.0460); the 0.2 mg/kg increased the negative conversion rate by 36.9%, and reduced IL-4 (*p* = 0.0365) and IL-8 levels (*p* = 0.0484). In this study, the adverse events occurred at a comparable rate across the four groups, with no unexpected safety findings observed. In conclusion, meplazumab promoted COVID-19 convalescence and reduced mortality, viral load, and cytokine levels in severe COVID-19 population with good safety profile.

## Introduction

The COVID-19 pandemic has swept across the world since 2019, infecting >630 million people and reporting a death toll of over six million. Severe and critical COVID-19 is life-threatening, especially for elder or unvaccinated individuals.^1,2^

Currently, neutralizing antibodies (casirivimab/imdevimab and amubarvimab/romlusevimab) and small molecular compounds (paxlovid and molnupiravir) have obtained approval for treating mild to moderate COVID-19.^3–7^ Although less prevalent, the severe COVID-19 usually have a poor prognosis, hence it is vital to find an effective and specific treatment for severe cases. Meanwhile, the rapid evolution and emerging variants of SARS-CoV-2 posed a great challenge to containing the pandemic. It was reported that the variants with multiple spike protein mutations could attenuate the neutralization potency and therapeutic effect of neutralization antibody, necessitating the need for innovative therapeutic agents and a unique approach.^8^

CD147, a type I transmembrane receptor, has been reported as a novel receptor for SARS-CoV-2 and its variants of concern (VOCs), including alpha, beta, gamma, delta,^9,10^ and omicron (data not shown), which could interact with the spike protein. It is also a vital mediator of cytokine storm, which has been linked to severe COVID-19.^11,12^ We previously developed a CD147 humanized antibody, meplazumab, which was demonstrated as a receptor blocker and could effectively inhibit the viral entry and cytokine storm caused by SARS-CoV-2. Our latest study revealed that meplazumab alleviates the progression of pulmonary fibrosis of COVID-19 by inhibiting the accumulation of activated fibroblasts and the production of ECM proteins.^13^

In phase 1 clinical study (NCT04369586), meplazumab (0.06 mg/kg to 0.56 mg/kg) showed favourable safety results, with no serious adverse event or adverse event grade ≥3 observed in healthy volunteers. The biodistribution study revealed that having reached the lung tissue and remained for >14 days, meplazumab effectively facilitated the treatment of COVID-19 pneumonia.

The therapeutic effect of meplazumab was preliminary investigated in an exploratory phase 2 study (NCT04275245) in 17 COVID-19 patients (six severe cases and seven critical cases). The results indicated that meplazumab could improve the discharged and case severity, and reduce the time to virus negative with favourable safety profile, suggesting that meplazumab has the potential of treating severe COVID-19 and this potential needs to be validated in phase 2/3 study with large sample size.^14^

Here, we report the results of DEFLECT, an ongoing international multi-centre, seamless, randomized, third-party double-blind, placebo-controlled phase 2/3 trial in hospitalized patients with severe COVID-19, to examine the efficacy and safety of meplazumab. The findings indicated that meplazumab has displayed beneficial results in patients with severe COVID-19, including decreased mortality, improved live discharge without oxygen supplement, and reduced level of viral load and cytokine, along with the established safety and tolerability.

## Results

### Patients

**Fig. 1:**
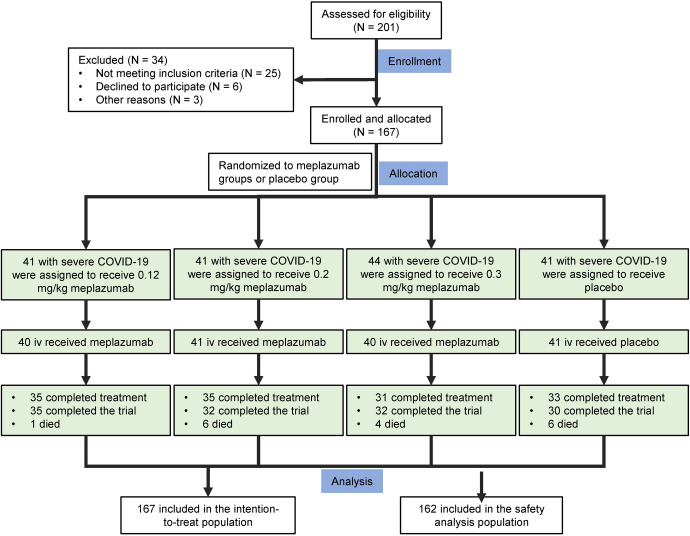
Enrolment and trial design

From 10 November 2020 to 18 November 2021, 201 patients underwent screening (Fig. 1). Of these patients, 167 patients underwent randomization and were assigned to three meplazumab groups (0.12 mg/kg group: 41 cases; 0.2 mg/kg group: 41 cases; 0.3 mg/kg group: 44 cases) or placebo group (41 cases) in a 1:1:1:1 ratio. Totally 167 patients were enroled in the intention-to-treat population, with 162 (97.0%) subjected to the safety analysis (0.12 mg/kg group: 40 [97.6%]; 0.2 mg/kg group: 41 [100.0%]; 0.3 mg/kg group: 40 [90.9%]; and placebo group: 41 [100.0%]). The 29-day follow-up evaluation was completed in 134 cases (80.2%), with 35 cases (85.4%) in the 0.12 mg/kg group, 35 (85.4%) in the 0.2 mg/kg group, 31 (70.5%) in the 0.3 mg/kg group, and 33 (80.5%) in the placebo group. In addition to the patients who died and were discharged, trial discontinuation before day 29 occurred in 18 patients (18/167, 10.8%) in the meplazumab group and 6 (6/167, 3.6%) in the placebo group.

As shown in Table 1, the disease characteristics and baseline demographic were generally balanced among the four groups. The study population had a median age of 47 years (range: 18–80 years). A total of 153 (91.6%) patients were <65 years, and 117 (70.1%) were male. A total of 161 (96.4%) did not receive COVID-19 vaccination at the time of enrolment; 149 (89.2%) did not use a combination of antiviral drugs; 17 (10.2%) used one antiviral drug; only 1 (0.6%) used multiple antiviral drugs; 160 (95.8%) had not used remdesivir in the previous treatment; and 159 (95.2%) did not use remdesivir in combination. The clinical disease severity was mainly grades 3–4 (grade 3: 48.4%; grade 4: 49.7%).

**Table 1:** Baseline demographic and clinical characteristics of patients with severe COVID-19

Characteristics	Meplazumab + SoC			
	0.12 mg/kg (*N* = 41) *n* (%)	0.2 mg/kg (*N* = 41) *n* (%)	0.3 mg/kg (*N* = 44) *n* (%)	Placebo + SoC (*N* = 41) *n* (%)
Median age (Min–Max)^a^	46.0 (18–74)	48.0 (19–80)	49.0 (27–77)	48.0 (25–73)
<65 years	37 (90.2)	38 (92.7)	40 (90.9)	38 (92.7)
≥65 years	4 (9.8)	3 (7.3)	4 (9.1)	3 (7.3)
Male	31 (75.6)	31 (75.6)	28 (63.6)	27 (65.9)
Race or ethnic group	41	39	44	39
Multiple^b^	0	0	0	0
American Indian or Alaska Native	0	0	0	0
Asian	2 (5.7)	3 (10.3)	4 (11.8)	1 (3.0)
Black or African American	4 (11.4)	3 (10.3)	5 (14.7)	4 (12.1)
Native Hawaiian or Other Pacific Islanders	0	0	1 (2.9)	0
White	29 (82.9)	21 (72.4)	24 (70.6)	26 (78.8)
Not reported	0	3 (7.3)	0	2 (4.9)
Unknown	6 (14.6)	9 (22.0)	10 (22.7)	6 (14.6)
Baseline weight (kg)				
No. of patients	40	41	41	41
Median body weight (Min–Max)	89.50 (67.0–129.0)	84.00 (62.9–133.4)	93.00 (60.0–146.3)	80.00 (59.0–127.0)
Concomitant antiviral agents used	41	41	44	41
None	36 (87.8)	37 (90.2)	40 (90.9)	36 (87.8)
Single antiviral	5 (12.2)	4 (9.8)	4 (9.1)	4 (9.8)
Multiple antiviral	0	0	0	1 (2.4)
Prior Remdesivir used	41	41	44	41
Yes	2 (4.9)	2 (4.9)	2 (4.5)	1 (2.4)
No	39 (95.1)	39 (95.1)	42 (95.5)	40 (97.6)
Concomitant Remdesivir used	41	41	44	41
Yes	2 (4.9)	2 (4.9)	3 (6.8)	1 (2.4)
No	39 (95.1)	39 (95.1)	41 (93.2)	40 (97.6)
Vaccine received	41	41	44	41
Yes	3 (7.3)	2 (4.9)	1 (2.3)	0
No	38 (92.7)	39 (95.1)	43 (97.7)	41 (100.0)
Baseline ordinal scale for clinical severity	37	41	41	40
Grade 3	17 (45.9)	20 (48.8)	21 (51.2)	19 (47.5)
Grade 4	20 (54.1)	21 (51.2)	17 (41.5)	21 (52.5)
Grade 5	0	0	3 (7.3)	0
Conditions meeting the risk factors of COVID-19^c^	41	41	43	40
Yes	21 (51.2)	18 (43.9)	27 (62.8)	22 (55.0)
No	20 (48.8)	23 (56.1)	16 (37.2)	18 (45.0)
Chest imaging performed^d^	31	31	33	34
Yes	31 (100.0)	31 (100.0)	33 (100.0)	34 (100.0)
Normal	0	1 (3.2)	2 (6.1)	2 (5.9)
Abnormal	31 (100.0)	29 (93.5)	31 (93.9)	30 (88.2)
No	0	0	0	0

Note: Baseline is defined as the last non-missing measurement before randomization (including unscheduled measurements, if any)

Note: For first level summaries, percentages are based on the number of patients with available data (n) in the analysis set by treatment group. For second level summaries, percentages are based on the number of patients in the first level

^a^Age, in years, is relative to the date of signed informed consent

^b^Patients who reported more than one race are reported under ‘Multiple’

^c^COVID-19 risk factors include: chronic lung disease, chronic renal disease, diabetes, heart disease, hypertension, autoimmune disease, women within 2 weeks postpartum and not breastfeeding, residents of long-term care facility, cancer, and organ transplant

^d^Chest imaging includes: X-ray, computerized tomography scan, MRI, PET, or others

Abbreviations: *N* Number of patients in analysis set, *n* Number of patients, *SoC* Standard of Care

In the safety analysis set, the median time of patients’ COVID-19 symptom onset was 11 days (range from 1 to 36 days) at study entry, and the median time to diagnose symptoms was 2 days (range from 0 to 38 days) at study entry. At the time of enrolment, 89.5% of the patients had shortness of breath, 78.4% had a cough, and 69.8% experienced fatigue or unwell. The median hospitalization days were 2 days (range from 1 to 63 days) since enrolment, and 97.5% were not in intensive care or high dependency units. 59.3% patients received oxygen therapy upon enrolment, with a median duration of 1 day (range from 0 to 32 days); none of the patients received invasive mechanical ventilation.

### Clinical efficacy

#### Mortality

All-cause death was observed from the day 1 to day 29. On day 29, 2.4% patients (1/41) in the 0.12 mg/kg group, 14.6% (6/41) in the 0.2 mg/kg, 9.1% (4/44) in the 0.3 mg/kg, and 14.6% (6/41) in the placebo group had died. The mortality of the 0.12 mg/kg group dropped by 83.6% when compared to the placebo group, marking a significant difference (*p* = 0.015, Table 2).

**Table 2:** Mortality and proportion of patients alive and discharged without supplemental oxygen on day 29

Characteristics	Statistics	Meplazumab + SoC			Placebo + SoC (*N* = 41)
		0.12 mg/kg (*N* = 41)	0.2 mg/kg (*N* = 41)	0.3 mg/kg (*N* = 44)	
Patients who died by day 29	n (%)	1 (2.4)	6 (14.6)	4 (9.1)	6 (14.6)
	Difference in proportions^a^	−12.2	0	−5.5	
CMH^†^	p-value	0.0150	0.9923	0.7291	
Patients alive and discharged without supplemental oxygen by day 29	n (%)	34 (82.9)	27 (65.9)	31 (70.5)	29 (70.7)
	Difference in proportions^a^	12.2	−4.8	−0.2	
CMH^†^	p-value	0.0337	0.4943	0.8286	

Note: Day of death was derived as (Date of death—Date of randomization + 1)

Note: Percentages are based on the number of patients in the analysis set by treatment group

Note: Patients with no data on day 29 are treated as a non-responder

^a^The difference in proportions is calculated as: the proportion of dose group—the proportion of control group

^†^p-value from a CMH test adjusted for age group (age < 65 years versus ≥65 years) and baseline severity grade

Abbreviations: *CMH* Cochran-Mantel-Haenszel, *N* number of patients in analysis set, *n* number of patients, *SoC* Standard of Care

#### Alive and discharged without supplemental oxygen

The number of patients alive and discharged without supplemental oxygen in all groups was recorded. On day 29, 34 (82.9%) of 41 patients in the 0.12 mg/kg group, 27 (65.9%) of 41 in the 0.2 mg/kg group, 31 (70.5%) of 44 in the 0.3 mg/kg group, and 29 (70.7%) of 41 in the placebo group were discharged without supplemental oxygen. The difference between the 0.12 mg/kg group and the placebo group is statistically significant (*p* = 0.0337, Table 2), and it is relatively higher in the 0.12 mg/kg group than the placebo group by 17.3%.

#### Response rate

On day 29, the response rate was 19 (46.3%) of 41 patients in the 0.12 mg/kg group, 22 (53.7%) of 41 in the 0.2 mg/kg group, 22 (50.0%) of 44 in the 0.3 mg/kg group, and 19 (46.3%) of 41 in the placebo group. In the 0.2 mg/kg and 0.3 mg/kg groups, the response rate rose by 16.0% and 8.0%, respectively, in comparison to the placebo group.

#### Sustained clinical improvement

By day 29, 17 (41.5%) of 41 patients in the 0.12 mg/kg group, 13 (31.7%) of 41 patients in the 0.2 mg/kg group, 13 (29.5%) of 44 patients in the 0.3 mg/kg group, and 13 of 41 patients (31.7%) in the placebo group achieved sustained clinical improvement. When compared to the placebo group, the percentage of patients who experienced sustained clinical improvement in the meplazumab 0.12 mg/kg group increased by 30.9% (Supplementary Table S1).

### Viral load

**Fig. 2:**
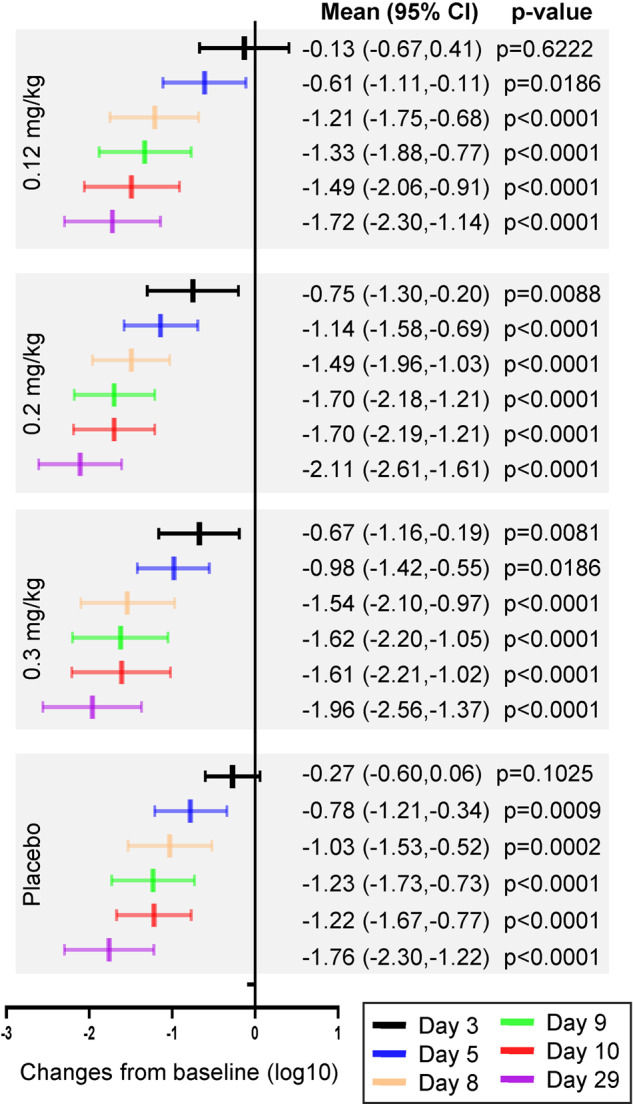
Forest plot of viral load changes from baseline (log10). The paired t-test was used to compare the viral load at each time point with baseline in each group

Viral load is associated with the body’s immune response, disease severity, and mortality.^15,16^ In this study, we monitored the viral load and analysed the negative rates. As shown in Fig. 2, on day 3, viral load was significantly decreased in the 0.2 and 0.3 mg/kg groups compared with the baseline (*p* = 0.0088 and *p* = 0.0081); no significant difference was observed in the 0.12 mg/kg group and the placebo group. As shown in Supplementary Table S2, the negative rates on day 29 were 53.9% in the 0.12 mg/kg group, 81.0% in the 0.2 mg/kg group, 91.7% in the 0.3 mg/kg group, and 65.2% in the placebo group, which were significantly different among the four groups analysed using the Chi-square test (*p* = 0.0163). The 0.2 and 0.3 mg/kg groups, compared with the placebo group, had a negative rate relatively increased by 24.1% and 40.6% respectively, with a significant difference observed in the 0.3 mg/kg group by Fisher’s exact probability (*p* = 0.0363).

The viral negative conversion rate was analysed using the Kaplan–Meier method, as shown in Supplementary Table S2. By day 10, the viral negative conversation rates in the 0.12 mg/kg and placebo groups were 12.4% (95% CI 4.9–29.8) and 6.9% (95% CI 1.8–24.9), respectively, which relatively increased by 79.9% compared with the placebo group. By day 29, the rates in the 0.2 mg/kg, 0.3 mg/kg, and placebo groups were 76.0% (95% CI 56.6–91.2), 71.8% (95% CI 53.8–87.5), and 55.5% (95% CI 37.5–75.1), respectively, which relatively increased by 36.9% (0.2 mg/kg group) and 29.5% (0.3 mg/kg group) compared with the placebo group. These results indicate that three dosages of meplazumab effectively reduces the viral load, increases the negative rate and the negative conversion rate. In the early stage of treatment, the 0.2 mg/kg dose showed greater effects on reducing viral load than that of the 0.3 mg/kg dose. Generally, 0.2 mg/kg dose had the most prominent anti-virus effect.

### Cytokine/chemokine’s level

Cytokine storm, featuring elevated cytokine and chemokine levels in serum, has a crucial role in the aggravation of COVID-19. We measured the serum level of 16 cytokines/chemokines, including IL-2, IL-4, IL-6, IL-8, IL-10, IL-12p70, IL-1ra, MCP-1, IL-15, IL-7, MIP-1β, IP-10, TNF-α, IL-17A, IL-2Rα, and IFN-γ; the results are shown in Supplementary Tables S3–S18, Fig. 3 and Supplementary Figs. S1–S32. The Chi-square test indicated no significant differences in the 16 cytokines/chemokines except for MCP-1 among the four groups at the baseline. On the other hand, IL-4 on day 2, and IFN-γ on day 8 were significantly different among the four groups (*p* = 0.0303 and *p* = 0.0459).

**Fig. 3:**
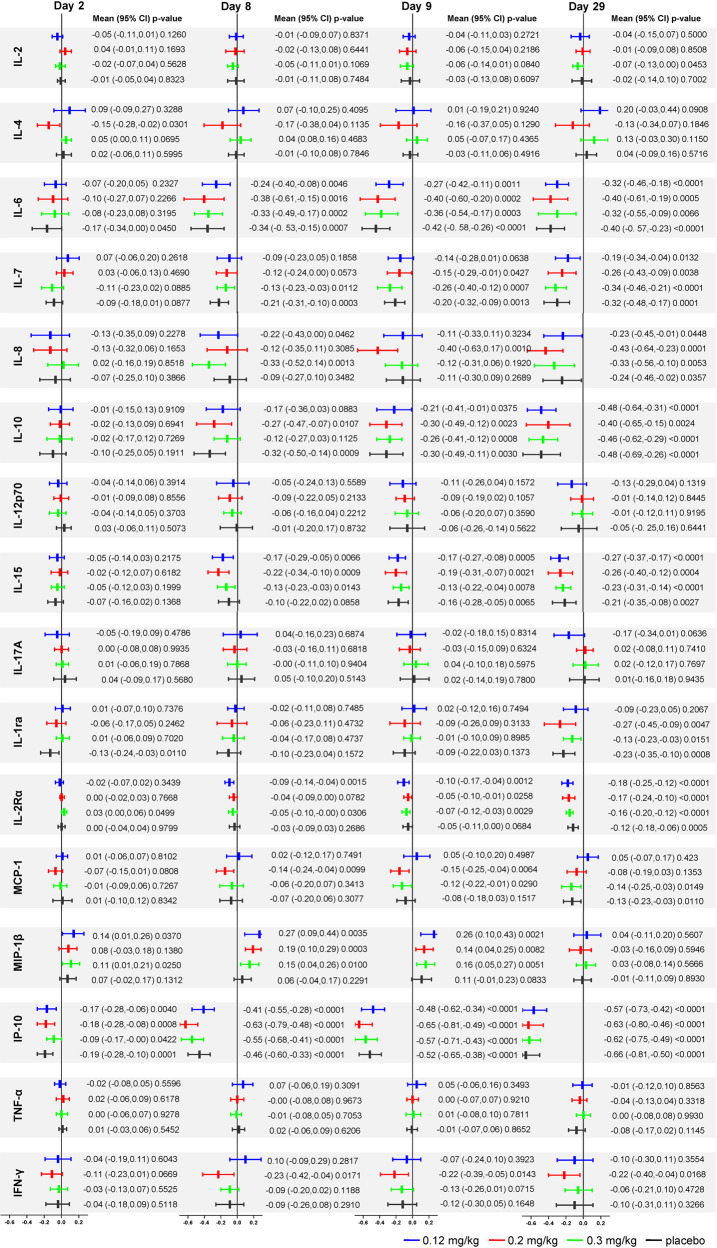
Forest plot of cytokine level changes from baseline (log10). The levels of cytokine/chemokines were converted to logarithmic form before statistical analysis and presented in the form of mean (95% CI). The paired t-test was used to compare the cytokine level at each time point with baseline in each group

Compared with the placebo group, the serum levels of 12 cytokines/chemokines decreased in all the meplazumab groups at multiple visit time points, among which 6 cytokines/chemokines were in the 0.12 mg/kg group, 10 in the 0.2 mg/kg group, and 10 in the 0.3 mg/kg group. Six kinds of cytokines/chemokines were in the 0.12 mg/kg group, 9 kinds in the 0.2 mg/kg group, and 6 kinds in the 0.3 mg/kg group showed decreased tendency from day 2 to day 29. The 0.2 mg/kg dose significantly reduced the IL-4 levels on day 2 (*p* = 0.0365) and IL-8 on day 9 (*p* = 0.0484). In addition, the 0.3 mg/kg meplazumab reduced the IL-8 levels on day 8 (*p* = 0.0460).

Compared with the baseline, the IL-8, IL-15, IL-2Rα, IFN-γ, and MCP-1 levels in the meplazumab groups declined significantly on days 8 and 9 (*p* < 0.05), among which 3 cytokines/chemokines were in the 0.12 mg/kg, 5 in the 0.2 mg/kg, and 4 in the 0.3 mg/kg group, whereas no significant differences were detected in the placebo group on days 8 and 9. These results indicate that the three dosages of meplazumab effectively reduce cytokine levels, especially on days 8 and 9. Generally, 0.2 mg/kg dose had the most prominent anti-cytokine storm effect.

### Safety

In this study, all meplazumab doses were associated with few, mainly low-grade, toxic effects. Serious treatment-emergent adverse events (TESAEs) occurred in 7 (17.5%) of 40 patients in the 0.12 mg/kg, 10 (24.4%) of 41 in the 0.2 mg/kg, 9 (22.5%) of 40 in the 0.3 mg/kg, and 13 (31.7%) of 41 in the placebo group. TEAEs were reported in 32 (80.0%) of 40 patients in the 0.12 mg/kg group, 36 (87.8%) of 41 in the 0.2 mg/kg group, 32 (80.0%) of 40 in the 0.3 mg/kg group, and 37 (90.2%) of 41 in the placebo group. Most adverse events were coincide with the reported COVID-19 complications and the majority of adverse events were not classified by the investigators as related to the trial drug.^17^ Drugs related to possible drug-related adverse events (DRAEs) were found in 3 (7.5%) of 40 patients in the 0.12 mg/kg group, 12 (29.3%) of 41 in the 0.2 mg/kg group, 4 (10%) of 40 in the 0.3 mg/kg group, and 4 (9.8%) of 41 in the placebo group. One patient had infusion-related reactions in the 0.12 mg/kg and 0.3 mg/kg groups (Table 3). The results of the ECG examination showed no differences among the four groups or between each dosage of the meplazumab and placebo groups, indicating that meplazumab has no cardiotoxicity (Supplementary Table S19). All safety results showed a consistent safety profile among the meplazumab and placebo groups, with no discernible disparity in safety events.

**Table 3:** Serious adverse events and adverse events of special interest in the safety analysis set

Characteristics	Meplazumab + SoC			Placebo + SoC (*N* = 41) *n* (%)
	0.12 mg/kg (*N* = 40) *n* (%)	0.2 mg/kg (*N* = 41) *n* (%)	0.3 mg/kg (*N* = 40) *n* (%)	
TEAE^a^	32 (80.0)	36 (87.8)	32 (80.0)	37 (90.2)
TEAE by severity^b^				
Severe TEAE	7 (17.5)	10 (24.4)	9 (22.5)	13 (31.7)
Moderate TEAE	6 (15.0)	13 (31.7)	5 (12.5)	6 (14.6)
Mild TEAE	19 (47.5)	13 (31.7)	18 (45.0)	18 (43.9)
TEAE by relationship to study drug^c^				
Definitely related	0	1 (2.4)	0	0
Probably related	1 (2.5)	3 (7.3)	0	0
Possibly related	2 (5.0)	8 (19.5)	4 (10.0)	4 (9.8)
Unlikely related	5 (12.5)	2 (4.9)	4 (10.0)	2 (4.9)
Not related	24 (60.0)	22 (53.7)	24 (60.0)	31 (75.6)
TEAE with an outcome of death	2 (5.0)	7 (17.1)	4 (10.0)	7 (17.1)
TEAE leading to permanent discontinuation of study drug	2 (5.0)	7 (17.1)	4 (10.0)	7 (17.1)
Serious TEAE (TESAE)	8 (20.0)	12 (29.3)	10 (25.0)	12 (29.3)
SAE related to study drug	1 (2.5)	0	1 (2.5)	0
TEAE of special interest	7 (17.5)	11 (26.8)	9 (22.5)	9 (22.0)
Disease related secondary infection complications	4 (10.0)	8 (19.5)	5 (12.5)	8 (19.5)
Grade 4 (CTCAE V5) neutropenia and lymphopenia	1 (2.5)	2 (4.9)	0	4 (9.8)
Anaphylactic reactions defined by clinical criteria	0	0	0	0
20% decline in oxygen saturation (SpO_2_) between start and end of 1-h study drug infusion	1 (2.5)	1 (2.4)	1 (2.5)	0
ALT or ASTå 3 × ULN and TBLå 2 × ULN	0	0	1 (2.5)	1 (2.4)
Evidence of RBC haemolysis^d^	1 (2.5)	1 (2.4)	2 (5.0)	0
Infusion related reaction	1 (2.5)	0	1 (2.5)	0

Note: Percentages are based on the number of patients in the analysis set by treatment group

Note: Patients with multiple events within a category are counted only once for that category

Note: Numbers within TEAE of special interest are not mutually exclusive

^a^TEAEs are defined as any adverse event that started or worsened in severity on or after the date of randomization

^b^TEAEs with a missing severity have been classified as severe

^c^A related TEAE is defined as a TEAE with a relationship of possibly related, probably related or definitely related. TEAEs with a missing relationship have been classified as related

^d^Evidence of RBC haemolysis is defined by 2 of the following three findings: Anaemia that is not due to another obvious cause; Increased reticulocyte count that is not explained by an obvious cause; and Signs of RBC destruction, such as increased lactate dehydrogenase, low haptoglobin <25 mg/dL, and increased unconjugated bilirubin

Abbreviations: *ALT* Alanine transaminase, *AST* Aspartate transaminase, *N* Number of patients in analysis set, *n* Number of patients, *RBC* Red blood cell, *SoC* Standard of Care, *TBL* Total bilirubin, *TEAE* Treatment-Emergent Adverse Event, *ULN* Upper limit of normal

## Discussion

Since the pandemic outbreak, several COVID-19 drugs, including neutralizing monoclonal antibodies, have been approved for commercialization.^18–21^ Still, severe COVID-19 reportedly may lead to multiple organ failure and even death.^22^ According to a recent study, patients with severe COVID-19 had a higher 12-month adjusted all-cause death risk than those with mild COVID-19.^23^ However, up to now, few antiviral drugs for severe and critical COVID-19 were approved. Emerging SARS-CoV-2 VOCs, such as delta and omicron, presented an ongoing risk of attenuating the efficacy of neutralizing antibody drugs, which makes it extremely difficult to control the pandemic.^8,24^

In DEFLECT, we assessed the therapeutic effect and safety of meplazumab in treating severe COVID-19. This clinical trial was designed to enrol hospitalized patients with severe COVID-19. The therapeutic efficacy was evaluated based on clinical outcomes, including mortality, live discharge, response rate, sustained clinical improvement, viral load, and cytokine/chemokine levels. Our data has showed that meplazumab reduced mortality (*p* = 0.015), increased the proportion of patients alive and discharged without supplemental oxygen (*p* = 0.0337), significantly improved the viral negative rate (*p* = 0.0363), and reduced the levels of several cytokines/chemokines. The response rate, sustained clinical improvement, and viral load were also improved by meplazumab treatment.

CD147, as a universal receptor of the spike protein, is identified to be able to mediate the cellular entry of SARS-CoV-2 and its VOCs, and meplazumab could effectively block the infection and replication, and alleviate the progression of pulmonary fibrosis.^9–11,13^ To validate these actions of meplazumab in the clinic, we analysed the viral load, negative rate, and viral negative conversation rate in this study. We found that 0.2 mg/kg and 0.3 mg/kg of meplazumab decreased the viral load as early as 3 days post administration, and the effect sustained until the end of observation on day 29, with virus negative rate ranging from 81.0% to 91.7%.

Cytokine storms have been reported as a key aspect of COVID-19, and infected patients showed high levels of several key proinflammatory cytokines such as IL-1RA, IL-4, IL-8, IFN-ɣ, MCP-1, MIP-1α, and TNF-α, and higher levels of IL-2 and IL-6 was observed in severe infection.^25^ Mortality of severe COVID-19 cases has been linked to cytokine storms, which frequently result in acute respiratory distress syndrome and multiple organ failure.^26–28^ Calming a cytokine storm is an important strategy for mitigating severe COVID-19. CD147 could induce cytokine storms by binding with the proinflammatory cytokine cyclophilin A (CyPA). It was reported that CD147 contributes to T cell metabolic dysfunction and dysregulation of inflammation in patients with COVID-19.^29^ We have proved that meplazumab could effectively block the SARS-CoV-2-induced cytokine release.^11^ In this study, several cytokine storm-related cytokines, including the IL-4, IL-8, MCP-1, and IFN-γ levels were significantly reduced by meplazumab. Notably, the decrease in IL-4 level occurred as early as 2 days after administration, with a significant difference, and 12 cytokines/chemokines showed reduced trends on days 8 and 9. Our data indicated that meplazumab treatment could restrain immune dysregulation in severe COVID-19 patients within 10 days.^30^

In DEFLECT, meplazumab is clinically effective at all three doses, and there are differences between treated groups and the placebo group in terms of mortality and proportion of subjects alive and discharge without supplemental oxygen on D29. The 0.12 mg/kg dose could decrease mortality, and enhance live discharge without oxygen supplementary significantly (*p* = 0.015 and 0.0337, respectively), and increase sustained clinical improvement by 30.9% compared with the placebo group. Meanwhile, the improved viral negative conversation rate and anti-cytokine storm effects were observed in 0.12 mg/kg group, which might contribute to the clinical benefits. The 0.2 mg/kg group could increase the response rate by 16.0% compared with the placebo group. For anti-virus effect, the 0.2 mg/kg dose could reduce the viral load, enhance the virus negative rate and viral negative conversation rate. For anti-inflammation effect, the 0.2 mg/kg dose could decrease serum levels of 9 cytokines/chemokines, including IL-4, IL-8, IL-15, MCP-1, and IFN-γ from day 2 through day 29 significantly (*p* < 0.05 for all). Based on the clinical benefits, viral load, negative rate, negative conversation rate, and anti-cytokine storm effect, the effective therapeutic doses were 0.12 mg/kg and 0.2 mg/kg.

The limitation of this study was that the bias in some demographic variable including the gender and age (elder vs younger). It has reported that males and older age was associated with the disease severity and this demographic bias may influence the therapeutic effect of meplazumab.^31^

SARS-CoV-2 is continuously mutating over the past 3 years of the COVID-19 epidemic. Currently, the omicron variants is dominating the world. There is an urgent need to develop new therapeutic strategies for emerging VOCs to control the epidemic. In DEFLECT, for the first time, we develop a first-in-class, receptor blocking, humanized antibody with dual action in alleviating viral infection and cytokine storms with a good safety profile for treatment of COVID-19.

## Materials and methods

### Study design and participants

There are two stages in the DEFLECT. Stage 1 is designated to determine the optimal dose and primary endpoints for stage 2. The trial was registered at ClinicalTrials.gov (NCT04586153).

This randomized controlled trial was carried out at nine sites in United States, Brazil, Pakistan, and Mexico in accordance with the ethical principles of the Declaration of Helsinki, the Good Clinical Practice guidelines, and all applicable regulatory requirements. The trial conduct and documentation were overseen by the local institutional review board or ethics committee at each trial centre. Additional details regarding the trial design are provided in the protocol (Supplementary Note 1).

The analysis described here involved 201 patients enroled in the trial to examine the safety and efficacy of meplazumab. All enroled patients were adults (≥18 years of age) with severe COVID-19 (grade 3 or 4 on a six-point ordinal scale, a modified ordinal scale recommended by the World Health Organization R&D Blueprint Group) and without COVID-19 vaccination within 2 weeks prior to randomization. The details of six-point ordinal scale were described in Supplementary Note 1. These patients had SARS-CoV-2 infection as confirmed by PCR in the laboratory or other public or commercial health tests in the patients’ country within 72 h. The investigators reviewed the symptoms, medical history, risk factors, and inclusion and exclusion criteria. If the investigator judged that the safety of the patients could be jeopardized in the study or if there was evidence of critical COVID-19 illness, the patients would be excluded. Concomitant medications were recorded in this study, and details of the excluded medications are provided in the Supplementary Note 1. Informed consent, either written or oral one from the patient’s legally authorized representative, was obtained from all patients. The inclusion and exclusion criteria is provided in the Supplementary Note 1.

### Randomization and masking

The study is a third-party double-blind study for all investigational staff who make determinations related to the study. The baseline data was collected, including information on demographic characteristics, concomitant antiviral agents, SARS-CoV-2 vaccination status, and clinical severity. Patients meet the criteria were assigned to four groups (0.12 mg/kg, 0.2 mg/kg, 0.3 mg/kg, or placebo) using a randomization code at a ratio of 1:1:1:1. Randomization was stratified by severity and age (age < 65 years or ≥65 years). The randomization schedule was created by the designee and stored in a secure area, and the randomization list was transferred via E-mail by unblinded study team leader. All patients received the standard of care (SoC) in compliance with the local practice. On the day of infusion, meplazumab (diluted in 0.9% saline) or placebo (0.9% saline) was administered intravenously. The solutions were indistinguishable, prepared by an unblinded third party (e.g., a pharmacist or nurse), and administered by an authorized blinded site staff member.

### Procedures

Meplazumab or placebo was administered intravenously in 100 mL 0.9% saline solution within 1 h after randomization. A single dose of 0.12 mg/kg was administered on day 1; and a double dose of 0.2 mg/kg, a double dose of 0.3 mg/kg, and a placebo was administered on days 1 and 8. The dose range of meplazumab was determined based on data of phase 1 clinical trial data from healthy patients (NCT04369586) and exploratory clinical trial from patients with COVID-19 (NCT04275245) to optimize the efficacy and safety.^14^ The schedule of assessments is described in the Supplementary Note 1.

### Outcomes

Outcomes were assessed on day 29 after randomization. The stage 1 was principally designed to specify the optimal dose and primary endpoints for stage 2, and no formal hypothesis testing was performed. We designed multiple pre-specified clinical endpoints for stage 1, including response rate on day 29, mortality on day 29, and proportion of patients alive and discharged without supplemental oxygen on day 29. The response was defined as a sustained clinical improvement of a two-point reduction on a six-point ordinal scale without subsequent worsening on day 29. The viral load of SARS-CoV-2 and cytokines/chemokines related to the inflammatory and immune status were analysed in a central laboratory, as described in the Supplementary Materials. Briefly, the viral load in nasopharyngeal swab samples was measured by quantitative reverse transcription (RT)-PCR using three pairs of unique primers for the ORF1ab, N protein, and S protein genes. The viral copy number was determined using the quantification cycle (Cq) data for the N protein gene only. Negative SARS-CoV-2 nucleic acid was defined as all three gene targets below the limit of quantitation (Cqs > 37). To assess the levels of cytokines and chemokines, venous blood samples were collected. The concentrations of a series of cytokines/chemokines, including tumour necrosis factor alpha (TNF-α), interferon gamma (IFN-γ), interleukin 1 receptor antagonist (IL-1ra), IL-2, IL-2Rα, IL-4, IL-6, IL-7, IL-8, IL-10, IL-15, IL-17A, IL-12p70, monocyte chemoattractant protein 1 (MCP-1), C-X-C motif chemokine 10 (IP-10), and macrophage inflammatory protein 1-beta (MIP-1β) were analysed. The statistical analysis plan for viral load and cytokines/chemokines was defined as the average change from baseline (day 1) to day 29.

For safety assessment, data from physical examination, clinical laboratory examinations, electrocardiograph (ECG) examination, vital signs, Treatment Emergent Adverse Events (TEAE), serious adverse events (SAE), adverse events of special interest, higher hypersensitivity, and infusion-related reactions were collected by day 29.

### Statistical analysis

In stage 1, we determined that the enrolment of 168 patients would provide a power of 81% to distinguish the response rate on day 29 of the dose groups from the placebo, assuming an 80% response rate for the meplazumab groups and 50% for the placebo. Additional details regarding the calculations of the sample size and total sample size are provided in the Supplementary Note 1.

Mortality on day 29 and the proportion of patients alive and discharged without supplemental oxygen on day 29 were analysed by the Cochran-Mantel-Haenszel (CMH) test and patients were stratified according to their age group and baseline severity grade. The *p*-value associated with the CMH statistic was compared at a two-sided 0.05 alpha level.

Changes from baseline of viral load and cytokine levels were statistically described by each group, and difference among groups were analysed statistically according to logarithmic converted ANOVA; the difference between the two groups were statistically tested using the logarithmic converted group t-test. Qualitative results of viral load were summarized by each group, and the difference among groups were statistically tested according to Chi-square Test or Fisher’s exact probability method. The negative conversion rate and the corresponding 95% confidence interval were calculated by Kaplan–Meier method (Greenwood method: log-log transformation) for each group.

All the statistical analyses were carried out by Statistical Analysis System (SAS) software, version 9.4 or higher (SAS Institute). The statistical analysis methods have been described in the Supplementary Note 2 and Note 3.

## Supplementary information

Sigtrans_Supplementary_Materials

Sigtrans_Supplementary_Note_1

Sigtrans_Supplementary_Note_2

Sigtrans_Supplementary_Note_3

CONSORT 2010 checklist

## Data Availability

The authors confirm that all data supporting the findings of this study are available upon reasonable request via E-mail. Participants data without names and identifiers will be made available after approval from all corresponding authors. The study protocol and statistical analysis plan (SAP) are available in the Supplementary Note 2 and Note 3.
