# Supplementary material for: Meplazumab in hospitalized adults with severe COVID-19 (DEFLECT): a multicenter, seamless phase 2/3, randomized, third-party double-blind clinical trial
Source: Signal Transduct Target Ther. 2023 Jan 30;8:46. doi: 10.1038/s41392-023-01323-9 (PMC9885411; doi:10.1038/s41392-023-01323-9)

Supplementary Materials for

Meplazumab in hospitalised adults with severe COVID-19 (DEFLECT): a multicenter, seamless phase 2/3, randomised, third-party double-blind clinical trial

Huijie Bian*, Liang Chen*, Zhao-Hui Zheng*, Xiu-Xuan Sun, Jie-Jie Geng, Ruo Chen, Ke Wang, Xu Yang, Shi-Rui Chen, Si-Yu Chen, Rong-Hua Xie, Kui Zhang, Jin-Lin Miao, Jun-Feng Jia, Hao Tang, Shuang-Shuang Liu, Hong-Wei Shi, Yong Yang, Xiao-Chun Chen, Vinay Malhotra, Nosheen Nasir, Iffat Khanum, Faisal Mahmood, Saeed Hamid, Claudio Marcel Berdun Stadnik, Kengi Itinose, Caroline Cândida Carvalho de Oliveira, Cesar Dusilek, Lucas Rivabem, Adilson Joaquim Westheimer Cavalcante, SuzaraSouto Lopes, Wladmir Faustino Saporito, Fábio José Co\ncilio Fucci, Jesus Abraham Simon-Campos, Ling Wang, Lin-Na Liu, Qing-Yi Wang, Ding Wei†, Zheng Zhang†, Zhi-Nan Chen†, and Ping Zhu†

Correspondence to: Dr. Zhu (zhuping@fmmu.edu.cn), Dr. Chen (znchen@fmmu.edu.cn), Dr. Zhang (cerc@aliyun.com ) and Dr. Wei (weidcq@fmmu.edu.cn)

**This file includes:**

Supplementary Methods

Supplementary Tables S1 to S19

Supplementary Figures. S1 to S32

Supplementary Methods

**Quantitation assay of SARS-CoV-2 viral load**

The SARS-CoV-2 Viral Load Quantitation Assay utilizes the MagMAX Viral/Pathogen Nucleic Acid Isolation Kit on the KingFisher system and reagents from the TaqPath COVID-19 Combo Kit, which includes the TaqPath RT-PCR COVID-19 Kit. The SARS-CoV-2 Viral Load Quantitation Assay is a laboratory developed test that uses components of the TaqPath COVID-19 Combo Kit along with calibration standards to generate a calibration curve that converts Cq (Quantitation Cycle) values into viral genome copy number for quantitation of SARS-CoV-2 viral load.

Briefly, nucleic acids are extracted from these specimens using the MagMAX Viral/Pathogen Nucleic Acid Isolation Kit, via an automated process using the KingFisher, and are reverse transcribed and amplified by polymerase chain reaction (RT-PCR) using the reagents from the TaqPath RT-PCR COVID-19 Kit on either the 7500 Fast Real-Time PCR or the 7500 Fast Dx Real-Time instruments. During RT-PCR, the probes anneal to three specific SARS-CoV-2 target sequences located between three unique forward and reverse primers for the ORF1ab, N Protein, and S Protein genes. During the extension phase of the PCR cycle, the 5’ nuclease activity of Taq polymerase degrades the probe, causing the reporter dye to separate from the quencher dye, generating a fluorescent signal. With each cycle, additional reporter dye molecules are cleaved from their respective probes, increasing the fluorescence intensity. Fluorescence intensity is monitored at each PCR cycle by the real-time PCR instrument. The data are analyzed and converted to viral genome copies/mL of sample using a calibration curve generated from seven standards ranging from 500,000,000 (5E8) copies/mL to 500 (5E2) copies/mL, performed as replicates of two through both extraction and amplification. Viral copy number is determined using the Cq data for the N Protein gene only. The negative of virus is defined as all three genes’ Cqs > 37.

The quantitation assay of SARS-CoV-2 viral load was executed in the Q2 Solutions clinical trials laboratory which located in Durham, NC, USA.

**Concentration assay of cytokines and chemokines**

Venous blood samples are collected to assess changes from baseline in cytokine, and chemokines related to inflammatory and immune status. The cytokine and chemokines includes tumor necrosis factor alpha (TNFα); interferon gamma (IFN γ); interleukin 1 receptor antagonist (IL-1ra); interleukin 2 (IL-2); interleukin-2 receptor alpha (IL-2 Rα); interleukin 4 (IL-4); interleukin 6 (IL-6); interleukin 7 (IL-7); interleukin 8 (IL-8); interleukin 10 (IL-10); interleukin 15 (IL-15); interleukin 17A (IL-17A); interleukin12p70 (IL-12p70); monocyte chemoattractant protein 1 (MCP 1), C-X-C motif chemokine 10 (IP-10) and macrophage inflammatory protein 1-beta (MIP-1β).

The concentration of MCP-1 was measured by Human CCL2/MCP-1 Quantikine ELISA Kit (DCP00, R&D Systems, Minneapolis, MN). The concentration of IL-1ra and IL-2Rα was measured by Magnetic Luminex Screening Kit (LXSAHM-02, R&D Systems, Minneapolis, MN). The concentration of TNFα, IFN γ, IL-1ra, IL-2, IL-2Rα, IL-4, IL-6, IL-7, IL-8, IL-10, IL-15, IL-17, IL-12p70, IP-10 and MIP-1β were measured by Milliplex Magnetic Bead Panel (HCYTOMAG-60K, Millipore, Burlington, MA). Analysis was done at a central laboratory.

The concentration assay of cytokines and chemokines was executed in the Q2 Solutions clinical trials laboratory (West Lothian, Scotland, UK). The performance characteristics of the assay kit were verified by Q2 Solutions Laboratory. Serum sample was contained a volume of 0.5 mL and was shipped and stored at -80°C until analysis. All samples were assayed in duplicate and the assay was performed according to the manufacturers protocol.

Table S1.

Time to sustained clinical improvement or live discharge from hospital by day 29.

| **Table S1. Time to sustained clinical improvement or live discharge from hospital by day 29** | | | | | | | |
| --- | --- | --- | --- | --- | --- | --- | --- |
| **Characteristics** |  | **Statistics** | **Meplazumab + SoC** | | |  | **Placebo + SoC**  **(N = 41)** |
|  |  |  | **0.12 mg/kg (N = 41)** | **0.2 mg/kg  (N = 41)** | **0.3 mg/kg  (N = 44)** |  |  |
| Number of subjects with event |  | n (%) | 36 (87.8) | 28 (68.3) | 33 (75.0) |  | 32 (78.0) |
| Type of event * |  |  |  |  |  |  |  |
| Sustained clinical improvement † |  | n (%) | 17 (41.5) | 13 (31.7) | 13 (29.5) |  | 13 (31.7) |
| Live discharge from hospital |  | n (%) | 19 (46.3) | 15 (36.6) | 20 (45.5) |  | 19 (46.3) |
|  |  |  |  |  |  |  |  |

* If more than 1 event occurs, the earliest event is counted.

† Sustained clinical improvement is defined as improvement of at least 2 points (from randomization) on a 6-point ordinal scale without subsequent worsening.

Note: Subjects not meeting the criteria for an event are censored at day 29.

Note: Percentages are based on the number of subjects in the analysis set by treatment group.

Abbreviations: SoC = Standard of Care; N = Number of subjects in analysis set; n = Number of subjects.

Table S2.

SARS-CoV-2 negative rate and viral load assessment

|  | **Meplazumab + Soc** | | | **Placebo + SoC** | **p*** |
| --- | --- | --- | --- | --- | --- |
|  | **0.12 mg/kg** | **0.2 mg/kg** | **0.3 mg/kg** |  |  |
| **Baseline** |  |  |  |  |  |
| Positive n (%) | 34 (100.0) | 33 (100.0) | 33 (100.0) | 34 (100.0) | 1.0000 |
| p (vs. placebo) † | 1.0000 | 1.0000 | 1.0000 | NA |  |
| **Day 3** |  |  |  |  |  |
| Negative n (%) | 3 (10.0) | 5 (17.2) | 2 (9.1) | 4 (13.8) | 0.8004 |
| p (vs. placebo) † | 0.7065 | 1.0000 | 0.6875 | NA |  |
| Change from baseline of viral load (mean ± SD) § | -0.13 ± 1.55 | -0.75 ± 1.54 | -0.67 ± 1.37 | -0.27 ± 0.94 | 0.1911 |
| p (vs. placebo) ‡ | 0.6562 | 0.1304 | 0.1669 | NA |  |
| Negative conversation rate % | 0 | 0 | 0 | 0 |  |
| 95% CI | (0–0) | (0–0) | (0–0) | (0–0) |  |
| **Day 5** |  |  |  |  |  |
| Negative n (%) | 2 (8.0) | 2 (6.9) | 4 (18.2) | 2 (9.5) | 0.6314 |
| p (vs. placebo) † | 1.0000 | 1.0000 | 0.6640 | NA |  |
| Change from baseline of viral load (mean ± SD) § | -0.61 ± 1.44 | -1.14 ± 1.27 | -0.98 ± 1.22 | -0.78 ± 1.24 | 0.3655 |
| p (vs. placebo) ‡ | 0.6117 | 0.2451 | 0.4900 | NA |  |
| negative conversation rate % | 0 | 0 | 0 | 0 |  |
| 95% CI | (0–0) | (0–0) | (0–0) | (0–0) |  |
| **Day 8** |  |  |  |  |  |
| Negative n (%) | 4 (15.4) | 6 (21.4) | 6 (22.2) | 6 (28.6) | 0.7575 |
| p (vs. placebo) † | 0.3064 | 0.5650 | 0.6143 | NA |  |
| Change from baseline of viral load (mean ± SD) § | -1.21 ± 1.52 | -1.49 ± 1.32 | -1.54 ± 1.59 | -1.03 ± 1.45 | 0.4427 |
| p (vs. placebo) ‡ | 0.6013 | 0.1711 | 0.1738 | NA |  |
| Negative conversation rate % | 3.0 | 0 | 3.5 | 3.5 |  |
| 95% CI | (0.4–19.6) | (0–0) | (0.5–22.1) | (0.5–22.1) |  |
| **Day 9** |  |  |  |  |  |
| Negative n (%) | 3 (15.8) | 6 (40.0) | 5 (45.5) | 5 (25.0) | 0.2462 |
| p (vs. placebo) † | 0.6948 | 0.4674 | 0.4232 | NA |  |
| Change from baseline of viral load (mean ± SD) § | -1.33 ± 1.60 | -1.70 ± 1.37 | -1.62 ± 1.63 | -1.23 ± 1.43 | 0.5264 |
| p (vs. placebo) ‡ | 0.7957 | 0.1790 | 0.2968 | NA |  |
| Negative conversation rate % | 3.1 | 3.9 | 3.5 | 6.9 |  |
| 95% CI | (0.4–19.6) | (0.6–24.3) | (0.5–22.1) | (1.8–24.9) |  |
| **Day 10** |  |  |  |  |  |
| Negative n (%) | 8 (42.1) | 4 (36.4) | 4 (26.7) | 4 (28.6) | 0.7888 |
| p (vs. placebo) † | 0.4244 | 1.0000 | 1.0000 | NA |  |
| Change from baseline of viral load (mean ± SD) § | -1.49 ± 1.65 | -1.70 ± 1.37 | -1.31 ± 1.68 | -1.22 ± 1.29 | 0.5882 |
| p (vs. placebo) ‡ | 0.4573 | 0.1468 | 0.2845 | NA |  |
| Negative conversation rate % | 12.4 | 3.9 | 3.5 | 6.9 |  |
| 95% CI | (4.9–29.8) | (0.6–24.3) | (0.5–22.1) | (1.8–24.9) |  |
| **Day 29** |  |  |  |  |  |
| Negative n (%) | 14 (53.9) | 17 (81.0) | 22 (91.7) | 15 (65.2) | 0.0163 |
| p (vs. placebo) † | 0.4190 | 0.2418 | 0.0363 | NA |  |
| Change from baseline of viral load (mean ± SD) § | -1.72 ± 1.67 | -2.11 ± 1.41 | -1.96 ± 1.69 | -1.76 ± 1.56 | 0.7257 |
| p (vs. placebo) ‡ | 0.9204 | 0.3416 | 0.6058 | NA |  |
| Negative conversation rate % | 52.8 | 76.0 | 71.8 | 55.5 |  |
| 95% CI | (36.5–71.2) | (56.6–91.2) | (53.8–87.5) | (37.5–75.1) |  |

*Number of patients with a positive or negative result compared among four groups using a Fisher’s exact test or chi-square test.

†Number of patients with a positive or negative result compared against that of the placebo group using a Fisher’s exact test or chi-square test.

§ Mean and standard deviation (SD) of change from baseline were calculated using logarithmic viral load (log10 copies/mL) in each group.

‡ Viral load result compared against that of the placebo group using an unpaired t-test.

NA=not applicable.

Table S3.

Level of IL-2 post administration (log10 pg/mL)

|  | **Meplazumab + SoC** | | | | **Placebo + Soc**  **(N=41)** | **p-value**‡ |
| --- | --- | --- | --- | --- | --- | --- |
|  | **0.12 mg/kg (N=39)** | | **0.2 mg/kg (N=41)** | **0.3 mg/kg (N=39)** |  |  |
| **Baseline*** |  | |  |  |  |  |
| n (Missing) | 39 (0) | | 41 (0) | 39 (0) | 39 (2) |  |
| Mean (SD) | 0.38 (0c39) | | 0.50 (0.55) | 0.35 (0.31) | 0.35 (0.33) | 0.3001 |
| Median | 0.20 | | 0.20 | 0.20 | 0.20 |  |
| Min, Max | 0.2, 2.1 | | 0.2, 2.2 | 0.2, 1.4 | 0.2, 1.5 |  |
| 95% CI | 0.26 – 0.51 | | 0.33 – 0.67 | 0.25 – 0.45 | 0.24 – 0.46 |  |
| p-value (vs. placebo) † | 0.7017 | | 0.1450 | 0.9992 | NA |  |
| **Day 2** |  | |  |  |  |  |
| n (Missing) | 39 (0) | | 41 (0) | 39 (0) | 41 (0) |  |
| Mean (SD) | 0.34 (0.37) | | 0.55 (0.58) | 0.33 (0.30) | 0.37 (0.34) | 0.0676 |
| Median | 0.20 | | 0.20 | 0.20 | 0.20 |  |
| Min, Max | 0.2, 2.1 | | 0.2, 2.0 | 0.2, 1.5 | 0.2, 1.4 |  |
| 95% CI | 0.21 – 0.46 | | 0.36 – 0.73 | 0.24 – 0.43 | 0.26 – 0.47 |  |
| p-value (vs. placebo) † | 0.7070 | | 0.0888 | 0.6695 | NA |  |
| **Change from baseline on day 2** |  | |  |  |  |  |
| n (Missing) | 39 (0) | | 41 (0) | 39 (0) | 39 (2) |  |
| Mean (SD) | -0.05 (0.19) | | 0.04 (0.21) | -0.02 (0.17) | -0.01 (0.15) | 0.1486 |
| Median | -0.00 | | -0.00 | -0.00 | -0.00 |  |
| Min, Max | -0.7, 0.3 | | -0.5, 0.6 | -0.7, 0.4 | -0.6, 0.4 |  |
| 95% CI | -0.11 – 0.01 | | -0.02 – 0.11 | -0.07 – 0.04 | -0.05 – 0.04 |  |
| p-value (vs. baseline) § | 0.1260 | | 0.1693 | 0.5628 | 0.8323 |  |
| p-value (vs. placebo) † | 0.2776 | | 0.2174 | 0.7609 | NA |  |
| **Day 8** |  | |  |  |  |  |
| n (Missing) | 39 (0) | | 41 (0) | 39 (0) | 41 (0) |  |
| Mean (SD) | 0.37 (0.30) | | 0.48 (0.47) | 0.30 (0.27) | 0.33 (0.29) | 0.1117 |
| Median | 0.20 | | 0.20 | 0.20 | 0.20 |  |
| Min, Max | 0.2, 1.5 | | 0.2, 1.7 | 0.2, 1.4 | 0.2, 1.4 |  |
| 95% CI | 0.28 – 0.47 | | 0.33 – 0.63 | 0.21 – 0.39 | 0.24 – 0.42 |  |
| p-value (vs. placebo) † | 0.4993 | | 0.0939 | 0.6339 | NA |  |
| **Change from baseline on day 8** |  | |  |  |  |  |
| n (Missing) | 39 (0) | | 41 (0) | 39 (0) | 39 (2) |  |
| Mean (SD) | -0.01 (0.24) | | -0.02 (0.33) | -0.05 (0.19) | -0.01 (0.29) | 0.9003 |
| Median | -0.00 | | -0.00 | -0.00 | -0.00 |  |
| Min, Max | -0.7, 0.7 | | -1.3, 0.9 | -0.9, 0.3 | -1.1, 0.5 |  |
| 95% CI | -0.09 – 0.07 | | -0.13 – 0.08 | -0.11 – 0.01 | -0.11 – 0.08 |  |
| p-value (vs. baseline) § | 0.8371 | | 0.6441 | 0.1069 | 0.7484 |  |
| p-value (vs. placebo) † | 0.9089 | | 0.8966 | 0.5155 | NA |  |
| **Day 9** |  | |  |  |  |  |
| n (Missing) | 39 (0) | | 41 (0) | 39 (0) | 41 (0) |  |
| Mean (SD) | 0.34 (0.36) | | 0.44 (0.46) | 0.29 (0.24) | 0.32 (0.30) | 0.2224 |
| Median | 0.20 | | 0.20 | 0.20 | 0.20 |  |
| Min, Max | 0.2, 2.1 | | 0.2, 1.8 | 0.2, 1.2 | 0.2, 1.3 |  |
| 95% CI | 0.23 – 0.46 | | 0.30 – 0.59 | 0.21 – 0.37 | 0.22 – 0.41 |  |
| p-value (vs. placebo) † | 0.7407 | | 0.1525 | 0.6185 | NA |  |
| **Change from baseline on day 9** |  | |  |  |  |  |
| n (Missing) | 39 (0) | | 41 (0) | 39 (0) | 39 (2) |  |
| Mean (SD) | -0.04 (0.22) | | -0.06 (0.30) | -0.06 (0.22) | -0.03 (0.33) | 0.9288 |
| Median | -0.00 | | -0.00 | -0.00 | -0.00 |  |
| Min, Max | -0.7, 0.6 | | -1.0, 0.5 | -0.9, 0.5 | -1.1, 1.1 |  |
| 95% CI | -0.11 – 0.03 | | -0.15 – 0.04 | -0.14 – 0.01 | -0.13 – 0.08 |  |
| p-value (vs. baseline) § | 0.2721 | | 0.2186 | 0.0840 | 0.6097 |  |
| p-value (vs. placebo) † | 0.8435 | | 0.6580 | 0.5661 | NA |  |
| **Day 29** |  | |  |  |  |  |
| n (Missing) | 39 (0) | | 41 (0) | 39 (0) | 41 (0) |  |
| Mean (SD) | 0.35 (0.40) | | 0.49 (0.48) | 0.28 (0.25) | 0.32 (0.33) | 0.0716 |
| Median | 0.20 | | 0.20 | 0.20 | 0.20 |  |
| Min, Max | 0.2, 2.3 | | 0.2, 1.8 | 0.2, 1.4 | 0.2, 1.3 |  |
| 95% CI | 0.21 – 0.48 | | 0.34 – 0.64 | 0.20 – 0.36 | 0.22 – 0.42 |  |
| p-value (vs. placebo) † | | 0.7766 | 0.0647 | 0.5649 | NA |  |
| **Change from baseline on day 29** | | |  |  |  |  |
| n (Missing) | 39 (0) | | 41 (0) | 39 (0) | 39 (2) |  |
| Mean (SD) | -0.04 (0.34) | | -0.01 (0.26) | -0.07 (0.20) | -0.02 (0.37) | 0.8406 |
| Median | -0.00 | | -0.00 | -0.00 | -0.00 |  |
| Min, Max | -0.8, 0.8 | | -1.0, 0.8 | -0.9, 0.3 | -1.1, 1.1 |  |
| 95% CI | -0.15 – 0.07 | | -0.09 – 0.08 | -0.13 – -0.00 | -0.14 – 0.10 |  |
| p-value (vs. baseline) § | 0.5000 | | 0.8508 | 0.0453 | 0.7002 |  |
| p-value (vs. placebo) † | 0.8619 | | 0.8325 | 0.5165 | NA |  |

* Baseline is defined as the last value before the first dose.

‡ Difference of cytokine’s level among the four groups was analyzed by the ANOVA.

† Difference of cytokine’s level compared with placebo group was analyzed by the independent samples t-test.

§ Difference of cytokine’s level compared with baseline in each group was analyzed by paired t-test.

Abbreviations: N = Number of patients in analysis set; n = Number of patients; SoC = Standard of Care; NA= Not Applicable.

Table S4.

Level of IL-4 post administration (log10 pg/mL)

|  | **Meplazumab + SoC** | | | | | **Placebo + Soc  (N=41)** | **p-value** ‡ |
| --- | --- | --- | --- | --- | --- | --- | --- |
|  | **0.12 mg/kg (N=39)** | **0.2 mg/kg (N=41)** | | **0.3 mg/kg (N=39)** | |  |  |
| **Baseline*** |  |  | |  | |  |  |
| n (Missing) | 32 (7) | 36 (5) | | 30 (9) | | 32 (9) |  |
| Mean (SD) | 1.43 (0.79) | 1.46 (0.73) | | 1.34 (0.75) | | 1.37 (0.75) | 0.9224 |
| Median | 0.90 | 0.90 | | 0.90 | | 0.90 |  |
| Min, Max | 0.9, 3.1 | 0.9, 3.4 | | 0.9, 3.1 | | 0.9, 3.0 |  |
| 95% CI | 1.14 – 1.71 | 1.21 – 1.70 | | 1.06 – 1.62 | | 1.10 – 1.64 |  |
| p-value (vs. placebo) † | 0.7648 | 0.6229 | | 0.8873 | | NA |  |
| **Day 2** |  |  | |  | |  |  |
| n (Missing) | 36 (3) | 41 (0) | | 36 (3) | | 39 (2) |  |
| Mean (SD) | 1.48 (0.78) | 1.34 (0.72) | | 1.37 (0.77) | | 1.40 (0.73) | 0.8784 |
| Median | 0.90 | 0.90 | | 0.90 | | 0.90 |  |
| Min, Max | 0.9, 3.2 | 0.9, 3.3 | | 0.9, 3.2 | | 0.9, 3.1 |  |
| 95% CI | 1.21 – 1.74 | 1.11 – 1.57 | | 1.11 – 1.63 | | 1.16 – 1.63 |  |
| p-value (vs. placebo) † | 0.6553 | 0.7215 | | 0.8845 | | NA |  |
| **Change from baseline on day 2** |  |  | |  | |  |  |
| n (Missing) | 32 (7) | 36 (5) | | 30 (9) | | 32 (9) |  |
| Mean (SD) | 0.09 (0.50) | -0.15 (0.40) | | 0.05 (0.15) | | 0.02 (0.24) | 0.0303 |
| Median | -0.00 | -0.00 | | -0.00 | | -0.00 |  |
| Min, Max | -1.6, 1.6 | -1.5, 0.4 | | -0.2, 0.5 | | -0.7, 0.8 |  |
| 95% CI | -0.09 – 0.27 | -0.28 – -0.02 | | -0.00 – 0.11 | | -0.06 – 0.11 |  |
| p-value (vs. baseline) § | 0.3288 | 0.0301 | | 0.0695 | | 0.5995 |  |
| p-value (vs. placebo) † | 0.5031 | 0.0365 | | 0.5713 | | NA |  |
| **Day 8** |  |  | |  | |  |  |
| n (Missing) | 39 (0) | 41 (0) | | 38 (1) | | 40 (1) |  |
| Mean (SD) | 1.43 (0.74) | 1.32 (0.71) | | 1.34 (0.78) | | 1.38 (0.72) | 0.9137 |
| Median | 0.90 | 0.90 | | 0.90 | | 0.90 |  |
| Min, Max | 0.9, 3.2 | 0.9, 3.4 | | 0.9, 3.3 | | 0.9, 3.1 |  |
| 95% CI | 1.19 – 1.68 | 1.10 – 1.55 | | 1.09 – 1.60 | | 1.15 – 1.61 |  |
| p-value (vs. placebo) † | 0.7427 | 0.7198 | | 0.8248 | | NA |  |
| **Change from baseline on day 8** |  |  | |  | |  |  |
| n (Missing) | 32 (7) | 36 (5) | | 30 (9) | | 32 (9) |  |
| Mean (SD) | 0.07 (0.48) | -0.17 (0.62) | | 0.04 (0.32) | | -0.01 (0.25) | 0.1284 |
| Median | -0.00 | -0.00 | | -0.00 | | -0.00 |  |
| Min, Max | -1.5, 1.5 | -1.5, 1.3 | | -0.8, 1.1 | | -0.7, 0.7 |  |
| 95% CI | -0.10 – 0.25 | -0.38 – 0.04 | | -0.08 – 0.16 | | -0.10 – 0.08 |  |
| p-value (vs. baseline) § | 0.4095 | 0.1135 | | 0.4683 | | 0.7846 |  |
| p-value (vs. placebo) † | 0.3884 | 0.1896 | | 0.4512 | | NA |  |
| **Day 9** |  |  | |  | |  |  |
| n (Missing) | 39 (0) | 41 (0) | | 38 (1) | | 41 (0) |  |
| Mean (SD) | 1.38 (0.73) | 1.32 (0.70) | | 1.41 (0.77) | | 1.37 (0.72) | 0.9587 |
| Median | 0.90 | 0.90 | | 0.90 | | 0.90 |  |
| Min, Max | 0.9, 3.1 | 0.9, 3.3 | | 0.9, 3.3 | | 0.9, 3.1 |  |
| 95% CI | 1.15 – 1.62 | 1.10 – 1.54 | | 1.15 – 1.66 | | 1.14 – 1.59 |  |
| p-value (vs. placebo) † | 0.9114 | 0.7747 | | 0.8008 | | NA |  |
| **Change from baseline on day 9** |  |  | |  | |  |  |
| n (Missing) | 32 (7) | 36 (5) | | 30 (9) | | 32 (9) |  |
| Mean (SD) | 0.01 (0.56) | -0.16 (0.62) | | 0.05 (0.32) | | -0.03 (0.23) | 0.2940 |
| Median | -0.00 | -0.00 | | -0.00 | | -0.00 |  |
| Min, Max | -1.6, 1.4 | -1.5, 1.3 | | -0.8, 1.1 | | -0.7, 0.7 |  |
| 95% CI | -0.19 – 0.21 | -0.37 – 0.05 | | -0.07 – 0.17 | | -0.11 – 0.06 |  |
| p-value (vs. baseline) § | 0.9240 | 0.1290 | | 0.4365 | | 0.4916 |  |
| p-value (vs. placebo) † | 0.7233 | 0.2604 | | 0.2951 | | NA |  |
| **Day 29** |  |  | |  | |  |  |
| n (Missing) | 39 (0) | 41 (0) | | 38 (1) | | 41 (0) |  |
| Mean (SD) | 1.54 (0.72) | | 1.34 (0.68) | | 1.44 (0.77) | 1.41 (0.74) | 0.6718 |
| Median | 1.31 | | 0.90 | | 0.90 | 0.90 |  |
| Min, Max | 0.9, 3.1 | | 0.9, 3.3 | | 0.9, 3.1 | 0.9, 3.1 |  |
| 95% CI | 1.31 – 1.78 | | 1.13 – 1.56 | | 1.18 – 1.69 | 1.17 – 1.64 |  |
| p-value (vs. placebo) † | 0.4144 | | 0.6806 | | 0.8691 | NA |  |
| **Change from baseline on day 29** |  | |  | |  |  |  |
| n (Missing) | 32 (7) | | 36 (5) | | 30 (9) | 32 (9) |  |
| Mean (SD) | 0.20 (0.66) | | -0.13 (0.59) | | 0.13 (0.45) | 0.04 (0.35) | 0.0560 |
| Median | -0.00 | | -0.00 | | -0.00 | -0.00 |  |
| Min, Max | -1.6, 1.4 | | -1.5, 1.6 | | -0.8, 1.5 | -0.7, 1.3 |  |
| 95% CI | -0.03 – 0.44 | | -0.34 – 0.07 | | -0.03 – 0.30 | -0.09 – 0.16 |  |
| p-value (vs. baseline) § | 0.0908 | | 0.1846 | | 0.1150 | 0.5716 |  |
| p-value (vs. placebo) † | 0.2074 | | 0.1634 | | 0.3374 | NA |  |

* Baseline is defined as the last value before the first dose.

‡ Difference of cytokine’s level among the four groups was analyzed by the ANOVA.

† Difference of cytokine’s level compared with placebo group was analyzed by the independent samples t-test.

§ Difference of cytokine’s level compared with baseline in each group was analyzed by paired t-test.

Abbreviations: N = Number of patients in analysis set; n = Number of patients; SoC = Standard of Care; NA= Not Applicable.

Table S5.

Level of IL-6 post administration (log10 pg/mL)

|  | **Meplazumab + SoC** | | | | | **placebo  (N=41)** | **p-value** ‡ |
| --- | --- | --- | --- | --- | --- | --- | --- |
|  | **0.12 mg/kg (N=39)** | **0.2 mg/kg (N=41)** | | **0.3 mg/kg (N=39)** | |  |  |
| **Baseline*** |  |  | |  | |  |  |
| n (Missing) | 39 (0) | 41 (0) | | 39 (0) | | 40 (1) |  |
| Mean (SD) | 0.97 (0.54) | 1.13 (0.57) | | 1.05 (0.71) | | 1.05 (0.54) | 0.6725 |
| Median | 1.02 | 1.28 | | 1.01 | | 1.22 |  |
| Min, Max | 0.2, 2.0 | 0.2, 2.3 | | 0.2, 2.5 | | 0.2, 1.8 |  |
| 95% CI | 0.79 – 1.14 | 0.95 – 1.31 | | 0.82 – 1.28 | | 0.88 – 1.23 |  |
| p-value (vs. placebo) † | 0.4784 | 0.5295 | | 0.9999 | | NA |  |
| **Day 2** |  |  | |  | |  |  |
| n (Missing) | 39 (0) | 41 (0) | | 39 (0) | | 41 (0) |  |
| Mean (SD) | 0.89 (0.64) | 1.03 (0.60) | | 0.98 (0.69) | | 0.89 (0.62) | 0.7011 |
| Median | 0.78 | 1.03 | | 0.85 | | 0.90 |  |
| Min, Max | 0.2, 2.1 | 0.2, 2.3 | | 0.2, 2.5 | | 0.2, 2.6 |  |
| 95% CI | 0.68 – 1.10 | 0.84 – 1.21 | | 0.75 – 1.20 | | 0.69 – 1.08 |  |
| p-value (vs. placebo) † | 0.9698 | 0.2939 | | 0.5359 | | NA |  |
| **Change from baseline on day 2** |  |  | |  | |  |  |
| n (Missing) | 39 (0) | 41 (0) | | 39 (0) | | 40 (1) |  |
| Mean (SD) | -0.07 (0.39) | -0.10 (0.54) | | -0.08 (0.47) | | -0.17 (0.53) | 0.7797 |
| Median | -0.00 | -0.07 | | -0.00 | | -0.01 |  |
| Min, Max | -0.9, 0.9 | -1.2, 1.8 | | -1.8, 0.8 | | -1.6, 1.4 |  |
| 95% CI | -0.20 – 0.05 | -0.27 – 0.07 | | -0.23 – 0.08 | | -0.34 – -0.00 |  |
| p-value (vs. baseline) § | 0.2327 | 0.2266 | | 0.3195 | | 0.0450 |  |
| p-value (vs. placebo) † | 0.3460 | 0.5545 | | 0.3890 | | NA |  |
| **Day 8** |  |  | |  | |  |  |
| n (Missing) | 39 (0) | 41 (0) | | 39 (0) | | 41 (0) |  |
| Mean (SD) | 0.73 (0.65) | 0.75 (0.60) | | 0.72 (0.72) | | 0.70 (0.68) | 0.9889 |
| Median | 0.20 | 0.61 | | 0.20 | | 0.20 |  |
| Min, Max | 0.2, 2.8 | 0.2, 2.3 | | 0.2, 2.5 | | 0.2, 2.8 |  |
| 95% CI | 0.52 – 0.94 | 0.56 – 0.94 | | 0.49 – 0.95 | | 0.49 – 0.91 |  |
| p-value (vs. placebo) † | 0.8488 | 0.7207 | | 0.8936 | | NA |  |
| **Change from baseline on day 8** |  |  | |  | |  |  |
| n (Missing) | 39 (0) | 41 (0) | | 39 (0) | | 40 (1) |  |
| Mean (SD) | -0.24 (0.49) | -0.38 (0.72) | | -0.33 (0.50) | | -0.34 (0.59) | 0.7360 |
| Median | -0.10 | -0.35 | | -0.17 | | -0.17 |  |
| Min, Max | -1.4, 0.8 | -1.8, 1.6 | | -1.8, 0.7 | | -1.4, 1.0 |  |
| 95% CI | -0.40 – -0.08 | -0.61 – -0.15 | | -0.49 – -0.17 | | -0.53 – -0.15 |  |
| p-value (vs. baseline) § | 0.0046 | 0.0016 | | 0.0002 | | 0.0007 |  |
| p-value (vs. placebo) † | 0.4025 | 0.7854 | | 0.9441 | | NA |  |
| **Day 9** |  |  | |  | |  |  |
| n (Missing) | 39 (0) | 41 (0) | | 39 (0) | | 41 (0) |  |
| Mean (SD) | 0.70 (0.60) | 0.73 (0.57) | | 0.70 (0.69) | | 0.62 (0.58) | 0.8825 |
| Median | 0.53 | 0.67 | | 0.20 | | 0.20 |  |
| Min, Max | 0.2, 2.1 | 0.2, 2.0 | | 0.2, 2.5 | | 0.2, 2.5 |  |
| 95% CI | 0.50 – 0.89 | 0.55 – 0.91 | | 0.47 – 0.92 | | 0.44 – 0.81 |  |
| p-value (vs. placebo) † | 0.5760 | 0.4058 | | 0.6133 | | NA |  |
| **Change from baseline on day 9** |  |  | |  | |  |  |
| n (Missing) | 39 (0) | 41 (0) | | 39 (0) | | 40 (1) |  |
| Mean (SD) | -0.27 (0.47) | -0.40 (0.63) | | -0.36 (0.56) | | -0.42 (0.50) | 0.6166 |
| Median | -0.08 | -0.45 | | -0.18 | | -0.36 |  |
| Min, Max | -1.4, 0.8 | -1.2, 1.8 | | -1.8, 0.9 | | -1.4, 0.7 |  |
| 95% CI | -0.42 – -0.11 | -0.60 – -0.20 | | -0.54 – -0.17 | | -0.58 – -0.26 |  |
| p-value (vs. baseline) § | 0.0011 | 0.0002 | | 0.0003 | | <0.0001 |  |
| p-value (vs. placebo) † | 0.1757 | 0.8941 | | 0.6084 | | NA |  |
| **Day 29** |  |  | |  | |  |  |
| n (Missing) | 39 (0) | 41 (0) | | 39 (0) | | 41 (0) |  |
| Mean (SD) | 0.65 (0.55) | | 0.73 (0.60) | | 0.73 (0.76) | 0.64 (0.55) | 0.8380 |
| Median | 0.20 | | 0.64 | | 0.20 | 0.20 |  |
| Min, Max | 0.2, 2.0 | | 0.2, 2.0 | | 0.2, 2.5 | 0.2, 1.9 |  |
| 95% CI | 0.47 – 0.82 | | 0.54 – 0.92 | | 0.49 – 0.98 | 0.46 – 0.81 |  |
| p-value (vs. placebo) † | 0.9612 | | 0.4644 | | 0.5344 | NA |  |
| **Change from baseline on day 29** |  | |  | |  |  |  |
| n (Missing) | 39 (0) | | 41 (0) | | 39 (0) | 40 (1) |  |
| Mean (SD) | -0.32 (0.44) | | -0.40 (0.67) | | -0.32 (0.70) | -0.40 (0.54) | 0.8733 |
| Median | -0.27 | | -0.38 | | -0.16 | -0.28 |  |
| Min, Max | -1.2, 0.5 | | -1.6, 1.8 | | -1.8, 2.3 | -1.4, 0.9 |  |
| 95% CI | -0.46 – -0.18 | | -0.61 – -0.19 | | -0.55 – -0.09 | -0.57 – -0.23 |  |
| p-value | <0.0001 | | 0.0005 | | 0.0066 | <0.0001 |  |
| p-value (vs. placebo) † | 0.4644 | | 0.9713 | | 0.5636 | NA |  |

* Baseline is defined as the last value before the first dose.

‡ Difference of cytokine’s level among the four groups was analyzed by the ANOVA.

† Difference of cytokine’s level compared with placebo group was analyzed by the independent samples t-test.

§ Difference of cytokine’s level compared with baseline in each group was analyzed by paired t-test.

Abbreviations: N = Number of patients in analysis set; n = Number of patients; SoC = Standard of Care; NA= Not Applicable.

Table S6.

Level of IL-7 post administration (log10 pg/mL)

|  | **Meplazumab + SoC** | | | | | **Placebo + Soc  (N=41)** | **p-value**‡ |
| --- | --- | --- | --- | --- | --- | --- | --- |
|  | **0.12 mg/kg (N=39)** | **0.2 mg/kg (N=41)** | | **0.3 mg/kg (N=39)** | |  |  |
| **Baseline*** |  |  | |  | |  |  |
| n (Missing) | 39 (0) | 41 (0) | | 38 (1) | | 39 (2) |  |
| Mean (SD) | 1.02 (0.41) | 1.09 (0.52) | | 0.95 (0.37) | | 1.09 (0.44) | 0.4569 |
| Median | 1.06 | 1.16 | | 1.02 | | 1.19 |  |
| Min, Max | 0.2, 1.7 | 0.2, 2.4 | | 0.2, 1.6 | | 0.2, 1.7 |  |
| 95% CI | 0.88 – 1.15 | 0.92 – 1.25 | | 0.83 – 1.07 | | 0.95 – 1.23 |  |
| p-value (vs. placebo) † | 0.4364 | 0.9554 | | 0.1349 | | NA |  |
| **Day 2** |  |  | |  | |  |  |
| n (Missing) | 39 (0) | 41 (0) | | 39 (0) | | 41 (0) |  |
| Mean (SD) | 1.09 (0.36) | 1.12 (0.48) | | 0.83 (0.40) | | 1.01 (0.48) | 0.0157 |
| Median | 1.17 | 1.19 | | 0.87 | | 1.13 |  |
| Min, Max | 0.2, 1.8 | 0.2, 2.2 | | 0.2, 1.6 | | 0.2, 1.8 |  |
| 95% CI | 0.97 – 1.20 | 0.97 – 1.27 | | 0.70 – 0.96 | | 0.86 – 1.16 |  |
| p-value (vs. placebo) † | 0.4383 | 0.3216 | | 0.0637 | | NA |  |
| **Change from baseline on day 2** |  |  | |  | |  |  |
| n (Missing) | 39 (0) | 41 (0) | | 38 (1) | | 39 (2) |  |
| Mean (SD) | 0.07 (0.39) | 0.03 (0.30) | | -0.11 (0.38) | | -0.09 (0.30) | 0.0591 |
| Median | 0.04 | 0.04 | | -0.01 | | -0.01 |  |
| Min, Max | -0.8, 1.0 | -0.9, 0.7 | | -0.8, 0.7 | | -1.0, 0.5 |  |
| 95% CI | -0.06 – 0.20 | -0.06 – 0.13 | | -0.23 – 0.02 | | -0.18 – 0.01 |  |
| p-value (vs. baseline) § | 0.2618 | 0.4690 | | 0.0885 | | 0.0877 |  |
| p-value (vs. placebo) † | 0.0518 | 0.0800 | | 0.7768 | | NA |  |
| **Day 8** |  |  | |  | |  |  |
| n (Missing) | 39 (0) | 41 (0) | | 39 (0) | | 41 (0) |  |
| Mean (SD) | 0.93 (0.50) | 0.97 (0.42) | | 0.81 (0.36) | | 0.89 (0.42) | 0.4282 |
| Median | 0.92 | 1.04 | | 0.79 | | 0.90 |  |
| Min, Max | 0.2, 1.8 | | 0.2, 2.0 | | 0.2, 1.5 | 0.2, 1.8 |  |
| 95% CI | 0.76 – 1.09 | | 0.84 – 1.10 | | 0.70 – 0.93 | 0.76 – 1.02 |  |
| p-value (vs. placebo) † | 0.7448 | | 0.4036 | | 0.3866 | NA |  |
| **Change from baseline on day 8** |  | |  | |  |  |  |
| n (Missing) | 39 (0) | | 41 (0) | | 38 (1) | 39 (2) |  |
| Mean (SD) | -0.09 (0.42) | | -0.12 (0.38) | | -0.13 (0.31) | -0.21 (0.32) | 0.5202 |
| Median | -0.09 | | -0.05 | | -0.12 | -0.13 |  |
| Min, Max | -0.9, 0.7 | | -1.2, 0.6 | | -1.1, 0.5 | -1.1, 0.5 |  |
| 95% CI | -0.23 – 0.05 | | -0.24 – 0.00 | | -0.23 – -0.03 | -0.31 – -0.10 |  |
| p-value (vs. baseline) § | 0.1858 | | 0.0573 | | 0.0112 | 0.0003 |  |
| p-value (vs. placebo) † | 0.1756 | | 0.2497 | | 0.2915 | NA |  |
| **Day 9** |  | |  | |  |  |  |
| n (Missing) | 39 (0) | | 41 (0) | | 39 (0) | 41 (0) |  |
| Mean (SD) | 0.88 (0.49) | | 0.94 (0.45) | | 0.69 (0.40) | 0.89 (0.44) | 0.0751 |
| Median | 0.83 | | 1.00 | | 0.69 | 0.93 |  |
| Min, Max | 0.2, 1.8 | | 0.2, 1.9 | | 0.2, 1.5 | 0.2, 1.8 |  |
| 95% CI | 0.72 – 1.04 | | 0.80 – 1.08 | | 0.56 – 0.82 | 0.75 – 1.03 |  |
| p-value (vs. placebo) † | 0.9288 | | 0.6260 | | 0.0373 | NA |  |
| **Change from baseline on day 9** |  | |  | |  |  |  |
| n (Missing) | 39 (0) | | 41 (0) | | 38 (1) | 39 (2) |  |
| Mean (SD) | -0.14 (0.45) | | -0.15 (0.45) | | -0.26 (0.43) | -0.20 (0.37) | 0.5568 |
| Median | -0.14 | | -0.11 | | -0.17 | -0.16 |  |
| Min, Max | -1.4, 0.7 | | -1.2, 0.9 | | -1.3, 0.7 | -1.1, 0.5 |  |
| 95% CI | -0.28 – 0.01 | | -0.29 – -0.01 | | -0.40 – -0.12 | -0.32 – -0.09 |  |
| p-value (vs. baseline) § | 0.0638 | | 0.0427 | | 0.0007 | 0.0013 |  |
| p-value (vs. placebo) † | 0.4625 | | 0.5397 | | 0.5463 | NA |  |
| **Day 29** |  | |  | |  |  |  |
| n (Missing) | 39 (0) | | 41 (0) | | 39 (0) | 41 (0) |  |
| Mean (SD) | 0.82 (0.48) | | 0.82 (0.48) | | 0.64 (0.42) | 0.77 (0.50) | 0.2563 |
| Median | 0.81 | | 0.80 | | 0.61 | 0.83 |  |
| Min, Max | 0.2, 1.7 | | 0.2, 1.9 | | 0.2, 1.6 | 0.2, 2.1 |  |
| 95% CI | 0.67 – 0.98 | | 0.67 – 0.97 | | 0.50 – 0.77 | 0.61 – 0.93 |  |
| p-value (vs. placebo) † | 0.6258 | | 0.6226 | | 0.2073 | NA |  |
| **Change from baseline on day 29** |  | |  | |  |  |  |
| n (Missing) | 39 (0) | | 41 (0) | | 38 (1) | 39 (2) |  |
| Mean (SD) | -0.19 (0.46) | | -0.26 (0.54) | | -0.34 (0.39) | -0.32 (0.48) | 0.5087 |
| Median | -0.10 | | -0.17 | | -0.36 | -0.31 |  |
| Min, M ax | -1.4, 0.7 | | -1.3, 1.0 | | -1.3, 0.4 | -1.4, 0.9 |  |
| 95% CI | -0.34 – -0.04 | | -0.43 – -0.09 | | -0.46 – -0.21 | -0.48 – -0.17 |  |
| p-value (vs. baseline) § | 0.0132 | | 0.0038 | | <0.0001 | 0.0001 |  |
| p-value (vs. placebo) † | 0.2151 | | 0.6226 | | 0.8985 | NA |  |

* Baseline is defined as the last value before the first dose.

‡ Difference of cytokine’s level among the four groups was analyzed by the ANOVA.

† Difference of cytokine’s level compared with placebo group was analyzed by the independent samples t-test.

§ Difference of cytokine’s level compared with baseline in each group was analyzed by paired t-test.

Abbreviations: N = Number of patients in analysis set; n = Number of patients; SoC = Standard of Care; NA= Not Applicable.

Table S7.

Level of IL-8 post administration (log10 pg/mL)

|  | **Meplazumab + SoC** | | | **Placebo + Soc  (N=41)** | **p-value** ‡ |
| --- | --- | --- | --- | --- | --- |
|  | **0.12 mg/kg (N=39)** | **0.2 mg/kg (N=41)** | **0.3 mg/kg (N=39)** |  |  |
| **Baseline*** |  |  |  |  |  |
| n (Missing) | 39 (0) | 41 (0) | 39 (0) | 40 (1) |  |
| Mean (SD) | 1.65 (0.52) | 1.70 (0.49) | 1.62 (0.52) | 1.63 (0.44) | 0.8978 |
| Median | 1.51 | 1.60 | 1.60 | 1.71 |  |
| Min, Max | 0.8, 2.6 | 0.6, 2.7 | 0.6, 3.0 | 0.2, 2.4 |  |
| 95% CI | 1.49 – 1.82 | 1.54 – 1.85 | 1.45 – 1.79 | 1.50 – 1.77 |  |
| p-value (vs. placebo) † | 0.8657 | 0.5422 | 0.8782 | NA |  |
| **Day 2** |  |  |  |  |  |
| n (Missing) | 39 (0) | 41 (0) | 39 (0) | 41 (0) |  |
| Mean (SD) | 1.52 (0.61) | 1.56 (0.68) | 1.63 (0.60) | 1.55 (0.61) | 0.8776 |
| Median | 1.52 | 1.49 | 1.48 | 1.60 |  |
| Min, Max | 0.2, 3.3 | 0.2, 3.4 | 0.2, 3.6 | 0.2, 3.0 |  |
| 95% CI | 1.32 – 1.72 | 1.35 – 1.78 | 1.44 – 1.83 | 1.36 – 1.75 |  |
| p-value (vs. placebo) † | 0.8117 | 0.9359 | 0.5529 | NA |  |
| **Change from baseline on day 2** |  |  |  |  |  |
| n (Missing) | 39 (0) | 41 (0) | 39 (0) | 40 (1) |  |
| Mean (SD) | -0.13 (0.67) | -0.13 (0.60) | 0.02 (0.53) | -0.07 (0.54) | 0.6439 |
| Median | -0.08 | -0.14 | 0.00 | -0.05 |  |
| Min, Max | -1.6, 1.9 | -1.2, 1.7 | -1.4, 1.3 | -1.2, 1.5 |  |
| 95% CI | -0.35 – 0.09 | -0.32 – 0.06 | -0.16 – 0.19 | -0.25 – 0.10 |  |
| p-value (vs. baseline) § | 0.2278 | 0.1653 | 0.8518 | 0.3866 |  |
| p-value (vs. placebo) † | 0.6784 | 0.6487 | 0.4536 | NA |  |
| **Day 8** |  |  |  |  |  |
| n (Missing) | 39 (0) | 41 (0) | 39 (0) | 41 (0) |  |
| Mean (SD) | 1.43 (0.70) | 1.58 (0.76) | 1.29 (0.52) | 1.54 (0.59) | 0.1891 |
| Median | 1.39 | 1.67 | 1.33 | 1.56 |  |
| Min, Max | 0.2, 3.4 | 0.2, 3.1 | 0.2, 2.2 | 0.2, 3.0 |  |
| 95% CI | 1.21 – 1.66 | 1.34 – 1.82 | 1.12 – 1.46 | 1.35 – 1.73 |  |
| p-value (vs. placebo) † | 0.4658 | 0.7991 | 0.0460 | NA |  |
| **Change from baseline on day 8** |  |  |  |  |  |
| n (Missing) | 39 (0) | 41 (0) | 39 (0) | 40 (1) |  |
| Mean (SD) | -0.22 (0.66) | -0.12 (0.74) | -0.33 (0.60) | -0.09 (0.57) | 0.3251 |
| Median | -0.18 | -0.08 | -0.18 | -0.07 |  |
| Min, Max | -2.1, 1.9 | -2.2, 1.6 | -2.0, 0.9 | -1.4, 1.1 |  |
| 95% CI | -0.43 – -0.00 | -0.35 – 0.11 | -0.52 – -0.14 | -0.27 – 0.10 |  |
| p-value (vs. baseline) § | 0.0462 | 0.3085 | 0.0013 | 0.3482 |  |
| p-value (vs. placebo) † | 0.3428 | 0.8220 | 0.0647 | NA |  |
| **Day 9** |  |  |  |  |  |
| n (Missing) | 39 (0) | 41 (0) | 39 (0) | 41 (0) |  |
| Mean (SD) | 1.54 (0.68) | 1.29 (0.72) | 1.50 (0.59) | 1.53 (0.64) | 0.3018 |
| Median | 1.40 | 1.13 | 1.42 | 1.45 |  |
| Min, Max | 0.2, 3.5 | 0.2, 3.1 | 0.2, 3.6 | 0.2, 3.0 |  |
| 95% CI | 1.32 – 1.76 | 1.07 – 1.52 | 1.30 – 1.69 | 1.32 – 1.73 |  |
| p-value (vs. placebo) † | 0.9043 | 0.1302 | 0.8358 | NA |  |
| **Change from baseline on day 9** |  |  |  |  |  |
| n (Missing) | 39 (0) | 41 (0) | 39 (0) | 40 (1) |  |
| Mean (SD) | -0.11 (0.69) | -0.40 (0.73) | -0.12 (0.57) | -0.11 (0.60) | 0.1109 |
| Median | -0.18 | -0.32 | -0.15 | -0.08 |  |
| Min, Max | -1.5, 1.8 | -2.3, 1.4 | -1.4, 1.9 | -1.6, 1.1 |  |
| 95% CI | -0.33 – 0.11 | -0.63 – -0.17 | -0.31 – 0.06 | -0.30 – 0.09 |  |
| p-value (vs. baseline) § | 0.3234 | 0.0010 | 0.1920 | 0.2689 |  |
| p-value (vs. placebo) † | 0.9791 | 0.0484 | 0.9050 | NA |  |
| **Day 29** |  |  |  |  |  |
| n (Missing) | 39 (0) | 41 (0) | 39 (0) | 41 (0) |  |
| Mean (SD) | 1.42 (0.58) | 1.26 (0.54) | 1.29 (0.67) | 1.37 (0.57) | 0.6085 |
| Median | 1.28 | 1.13 | 1.22 | 1.26 |  |
| Min, Max | 0.2, 2.7 | 0.2, 3.1 | 0.2, 3.7 | 0.2, 3.0 |  |
| 95% CI | 1.24 – 1.61 | 1.09 – 1.44 | 1.07 – 1.51 | 1.19 – 1.55 |  |
| p-value (vs. placebo) † | 0.6709 | 0.4013 | 0.5617 | NA |  |
| **Change from baseline on day 29** |  |  |  |  |  |
| n (Missing) | 39 (0) | 41 (0) | 39 (0) | 40 (1) |  |
| Mean (SD) | -0.23 (0.69) | -0.43 (0.65) | -0.33 (0.70) | -0.24 (0.69) | 0.4984 |
| Median | -0.17 | -0.36 | -0.25 | -0.19 |  |
| Min, Max | -1.7, 1.3 | -2.0, 1.4 | -1.6, 2.7 | -1.6, 2.1 |  |
| 95% CI | -0.45 – -0.01 | -0.64 – -0.23 | -0.56 – -0.10 | -0.46 – -0.02 |  |
| p-value (vs. baseline) § | 0.0448 | 0.0001 | 0.0053 | 0.0357 |  |
| p-value (vs. placebo) † | 0.9603 | 0.1912 | 0.5492 | NA |  |

* Baseline is defined as the last value before the first dose.

‡ Difference of cytokine’s level among the four groups was analyzed by the ANOVA.

† Difference of cytokine’s level compared with placebo group was analyzed by the independent samples t-test.

§ Difference of cytokine’s level compared with baseline in each group was analyzed by paired t-test.

Abbreviations: N = Number of patients in analysis set; n = Number of patients; SoC = Standard of Care; NA= Not Applicable.

Table S8.

Level of IL-10 post administration (log10 pg/mL)

|  | **Meplazumab + SoC** | | | **Placebo + Soc  (N=41)** | **p-value** ‡ |
| --- | --- | --- | --- | --- | --- |
|  | **0.12 mg/kg (N=39)** | **0.2 mg/kg (N=41)** | **0.3 mg/kg (N=39)** |  |  |
| **Baseline*** |  |  |  |  |  |
| n (Missing) | 37 (2) | 38 (3) | 34 (5) | 39 (2) |  |
| Mean (SD) | 1.36 (0.36) | 1.48 (0.54) | 1.46 (0.34) | 1.45 (0.49) | 0.6554 |
| Median | 1.41 | 1.43 | 1.44 | 1.44 |  |
| Min, Max | 0.2, 2.0 | 0.2, 2.6 | 0.8, 2.1 | 0.2, 2.8 |  |
| 95% CI | 1.24 – 1.48 | 1.30 – 1.66 | 1.34 – 1.58 | 1.29 – 1.61 |  |
| p-value (vs. placebo) † | 0.3359 | 0.8354 | 0.9808 | NA |  |
| **Day 2** |  |  |  |  |  |
| n (Missing) | 39 (0) | 41 (0) | 38 (1) | 41 (0) |  |
| Mean (SD) | 1.37 (0.43) | 1.49 (0.45) | 1.37 (0.44) | 1.36 (0.40) | 0.5042 |
| Median | 1.33 | 1.51 | 1.39 | 1.34 |  |
| Min, Max | 0.2, 2.4 | 0.5, 2.4 | 0.2, 2.2 | 0.7, 2.8 |  |
| 95% CI | 1.23 – 1.51 | 1.34 – 1.63 | 1.22 – 1.51 | 1.23 – 1.49 |  |
| p-value (vs. placebo) † | 0.9239 | 0.1849 | 0.9268 | NA |  |
| **Change from baseline on day 2** |  |  |  |  |  |
| n (Missing) | 37 (2) | 38 (3) | 34 (5) | 39 (2) |  |
| Mean (SD) | -0.01 (0.41) | -0.02 (0.33) | -0.02 (0.41) | -0.10 (0.47) | 0.7505 |
| Median | -0.01 | -0.06 | -0.00 | -0.07 |  |
| Min, Max | -0.8, 1.0 | -0.5, 0.8 | -0.6, 1.2 | -1.1, 1.3 |  |
| 95% CI | -0.15 – 0.13 | -0.13 – 0.09 | -0.17 – 0.12 | -0.25 – 0.05 |  |
| p-value (vs. baseline) § | 0.9109 | 0.6941 | 0.7269 | 0.1911 |  |
| p-value (vs. placebo) † | 0.3636 | 0.3983 | 0.4685 | NA |  |
| **Day 8** |  |  |  |  |  |
| n (Missing) | 39 (0) | 41 (0) | 38 (1) | 41 (0) |  |
| Mean (SD) | 1.19 (0.62) | 1.21 (0.51) | 1.30 (0.47) | 1.14 (0.52) | 0.5896 |
| Median | 1.20 | 1.25 | 1.25 | 1.10 |  |
| Min, Max | 0.2, 3.7 | 0.2, 2.2 | 0.2, 2.4 | 0.2, 2.8 |  |
| 95% CI | 0.98 – 1.39 | 1.05 – 1.37 | 1.15 – 1.46 | 0.98 – 1.30 |  |
| p-value (vs. placebo) † | 0.7292 | 0.5394 | 0.1499 | NA |  |
| **Change from baseline on day 8** |  |  |  |  |  |
| n (Missing) | 37 (2) | 38 (3) | 34 (5) | 39 (2) |  |
| Mean (SD) | -0.17 (0.58) | -0.27 (0.61) | -0.12 (0.44) | -0.32 (0.55) | 0.4099 |
| Median | -0.08 | -0.15 | -0.17 | -0.35 |  |
| Min, Max | -1.3, 2.1 | -2.1, 0.7 | -1.0, 1.0 | -1.4, 1.3 |  |
| 95% CI | -0.36 – 0.03 | -0.47 – -0.07 | -0.27 – 0.03 | -0.50 – -0.14 |  |
| p-value (vs. baseline) § | 0.0883 | 0.0107 | 0.1125 | 0.0009 |  |
| p-value (vs. placebo) † | 0.2531 | 0.6957 | 0.0982 | NA |  |
| **Day 9** |  |  |  |  |  |
| n (Missing) | 39 (0) | 41 (0) | 38 (1) | 41 (0) |  |
| Mean (SD) | 1.15 (0.65) | 1.17 (0.48) | 1.17 (0.40) | 1.16 (0.51) | 0.9984 |
| Median | 1.08 | 1.14 | 1.11 | 1.15 |  |
| Min, Max | 0.2, 3.8 | 0.2, 2.1 | 0.6, 2.2 | 0.2, 2.8 |  |
| 95% CI | 0.94 – 1.36 | 1.02 – 1.32 | 1.04 – 1.30 | 0.99 – 1.32 |  |
| p-value (vs. placebo) † | 0.9603 | 0.9114 | 0.9266 | NA |  |
| **Change from baseline on day 9** |  |  |  |  |  |
| n (Missing) | 37 (2) | 38 (3) | 34 (5) | 39 (2) |  |
| Mean (SD) | -0.21 (0.60) | -0.30 (0.57) | -0.26 (0.42) | -0.30 (0.59) | 0.8849 |
| Median | -0.25 | -0.21 | -0.23 | -0.37 |  |
| Min, Max | -1.3, 2.2 | -1.5, 0.7 | -1.1, 0.7 | -1.5, 1.3 |  |
| 95% CI | -0.41 – -0.01 | -0.49 – -0.12 | -0.41 – -0.12 | -0.49 – -0.11 |  |
| p-value (vs. baseline) § | 0.0375 | 0.0023 | 0.0008 | 0.0030 |  |
| p-value (vs. placebo) † | 0.5283 | 0.9754 | 0.7686 | NA |  |
| **Day 29** |  |  |  |  |  |
| n (Missing) | 39 (0) | 41 (0) | 39 (0) | 41 (0) |  |
| Mean (SD) | 0.94 (0.51) | 1.09 (0.55) | 0.98 (0.43) | 0.97 (0.64) | 0.6387 |
| Median | 0.85 | 1.04 | 1.10 | 0.90 |  |
| Min, Max | 0.2, 2.7 | 0.2, 2.3 | 0.2, 1.8 | 0.2, 2.9 |  |
| 95% CI | 0.77 – 1.11 | 0.91 – 1.26 | 0.84 – 1.12 | 0.77 – 1.17 |  |
| p-value (vs. placebo) † | 0.8211 | 0.3802 | 0.9543 | NA |  |
| **Change from baseline on day 29** |  |  |  |  |  |
| n (Missing) | 37 (2) | 38 (3) | 34 (5) | 39 (2) |  |
| Mean (SD) | -0.48 (0.49) | -0.40 (0.76) | -0.46 (0.46) | -0.48 (0.67) | 0.9475 |
| Median | -0.41 | -0.42 | -0.39 | -0.56 |  |
| Min, Max | -1.4, 0.6 | -2.2, 1.1 | -1.4, 0.4 | -1.7, 1.3 |  |
| 95% CI | -0.64 – -0.31 | -0.65 – -0.15 | -0.62 – -0.29 | -0.69 – -0.26 |  |
| p-value (vs. baseline) § | <0.0001 | 0.0024 | <0.0001 | <0.0001 |  |
| p-value (vs. placebo) † | 0.9939 | 0.6523 | 0.8818 | NA |  |

* Baseline is defined as the last value before the first dose.

‡ Difference of cytokine’s level among the four groups was analyzed by the ANOVA.

† Difference of cytokine’s level compared with placebo group was analyzed by the independent samples t-test.

§ Difference of cytokine’s level compared with baseline in each group was analyzed by paired t-test.

Abbreviations: N = Number of patients in analysis set; n = Number of patients; SoC = Standard of Care; NA= Not Applicable.

Table S9.

Level of IL-12p70 post administration (log10 pg/mL)

|  | **Meplazumab + SoC** | | | **Placebo + Soc  (N=41)** | **p-value** ‡ |
| --- | --- | --- | --- | --- | --- |
|  | **0.12 mg/kg (N=39)** | **0.2 mg/kg (N=41)** | **0.3 mg/kg (N=39)** |  |  |
| **Baseline*** |  |  |  |  |  |
| n (Missing) | 39 (0) | 41 (0) | 39 (0) | 40 (1) |  |
| Mean (SD) | 0.64 (0.52) | 0.76 (0.74) | 0.53 (0.54) | 0.58 (0.59) | 0.3780 |
| Median | 0.20 | 0.20 | 0.20 | 0.20 |  |
| Min, Max | 0.2, 1.7 | 0.2, 3.0 | 0.2, 2.0 | 0.2, 2.0 |  |
| 95% CI | 0.47 – 0.81 | 0.52 – 0.99 | 0.36 – 0.71 | 0.39 – 0.76 |  |
| p-value (vs. placebo) † | 0.6097 | 0.2275 | 0.7438 | NA |  |
| **Day 2** |  |  |  |  |  |
| n (Missing) | 39 (0) | 41 (0) | 39 (0) | 41 (0) |  |
| Mean (SD) | 0.60 (0.54) | 0.75 (0.77) | 0.49 (0.53) | 0.59 (0.57) | 0.3019 |
| Median | 0.20 | 0.20 | 0.20 | 0.20 |  |
| Min, Max | 0.2, 1.9 | 0.2, 2.6 | 0.2, 2.2 | 0.2, 1.9 |  |
| 95% CI | 0.42 – 0.77 | 0.51 – 0.99 | 0.32 – 0.66 | 0.41 – 0.77 |  |
| p-value (vs. placebo) † | 0.9671 | 0.2965 | 0.4088 | NA |  |
| **Change from baseline on day 2** |  |  |  |  |  |
| n (Missing) | 39 (0) | 41 (0) | 39 (0) | 40 (1) |  |
| Mean (SD) | -0.04 (0.30) | -0.01 (0.27) | -0.04 (0.29) | 0.03 (0.26) | 0.6491 |
| Median | -0.00 | -0.00 | -0.00 | -0.00 |  |
| Min, Max | -1.1, 0.5 | -0.7, 0.9 | -1.2, 0.5 | -0.6, 0.9 |  |
| 95% CI | -0.14 – 0.06 | -0.09 – 0.08 | -0.14 – 0.05 | -0.06 – 0.11 |  |
| p-value (vs. baseline) § | 0.3914 | 0.8556 | 0.3703 | 0.5073 |  |
| p-value (vs. placebo) † | 0.2764 | 0.5543 | 0.2643 | NA |  |
| **Day 8** |  |  |  |  |  |
| n (Missing) | 39 (0) | 41 (0) | 39 (0) | 41 (0) |  |
| Mean (SD) | 0.59 (0.57) | 0.67 (0.66) | 0.47 (0.46) | 0.55 (0.52) | 0.4682 |
| Median | 0.20 | 0.20 | 0.20 | 0.20 |  |
| Min, Max | 0.2, 2.8 | 0.2, 2.3 | 0.2, 1.9 | 0.2, 1.7 |  |
| 95% CI | 0.40 – 0.77 | 0.46 – 0.88 | 0.32 – 0.62 | 0.39 – 0.72 |  |
| p-value (vs. placebo) † | 0.7898 | 0.3684 | 0.4822 | NA |  |
| **Change from baseline on day 8** |  |  |  |  |  |
| n (Missing) | 39 (0) | 41 (0) | 39 (0) | 40 (1) |  |
| Mean (SD) | -0.05 (0.58) | -0.09 (0.43) | -0.06 (0.30) | -0.01 (0.57) | 0.9295 |
| Median | -0.00 | -0.00 | -0.00 | -0.00 |  |
| Min, Max | -1.1, 2.6 | -1.6, 0.6 | -0.8, 0.7 | -1.6, 1.4 |  |
| 95% CI | -0.24 – 0.13 | -0.22 – 0.05 | -0.16 – 0.04 | -0.20 – 0.17 |  |
| p-value (vs. baseline) § | 0.5589 | 0.2133 | 0.2212 | 0.8732 |  |
| p-value (vs. placebo) † | 0.7554 | 0.5259 | 0.6622 | NA |  |
| **Day 9** |  |  |  |  |  |
| n (Missing) | 39 (0) | 41 (0) | 39 (0) | 41 (0) |  |
| Mean (SD) | 0.53 (0.47) | 0.67 (0.67) | 0.47 (0.49) | 0.51 (0.50) | 0.3760 |
| Median | 0.20 | 0.20 | 0.20 | 0.20 |  |
| Min, Max | 0.2, 1.7 | 0.2, 2.4 | 0.2, 1.8 | 0.2, 2.0 |  |
| 95% CI | 0.38 – 0.69 | 0.46 – 0.88 | 0.31 – 0.63 | 0.35 – 0.67 |  |
| p-value (vs. placebo) † | 0.8331 | 0.2231 | 0.7228 | NA |  |
| **Change from baseline on day 9** |  |  |  |  |  |
| n (Missing) | 39 (0) | 41 (0) | 39 (0) | 40 (1) |  |
| Mean (SD) | -0.11 (0.46) | -0.09 (0.34) | -0.06 (0.42) | -0.06 (0.62) | 0.9639 |
| Median | -0.00 | -0.00 | -0.00 | -0.00 |  |
| Min, Max | -1.4, 0.8 | -0.8, 0.5 | -0.9, 1.1 | -1.6, 1.8 |  |
| 95% CI | -0.26 – 0.04 | -0.19 – 0.02 | -0.20 – 0.07 | -0.26 – 0.14 |  |
| p-value (vs. baseline) § | 0.1572 | 0.1057 | 0.3590 | 0.5622 |  |
| p-value (vs. placebo) † | 0.6920 | 0.7861 | 0.9671 | NA |  |
| **Day 29** |  |  |  |  |  |
| n (Missing) | 39 (0) | 41 (0) | 39 (0) | 41 (0) |  |
| Mean (SD) | 0.51 (0.51) | 0.74 (0.69) | 0.53 (0.55) | 0.52 (0.50) | 0.1946 |
| Median | 0.20 | 0.20 | 0.20 | 0.20 |  |
| Min, Max | 0.2, 2.1 | 0.2, 2.4 | 0.2, 1.9 | 0.2, 2.0 |  |
| 95% CI | 0.35 – 0.68 | 0.53 – 0.96 | 0.35 – 0.70 | 0.36 – 0.68 |  |
| p-value (vs. placebo) † | 0.9551 | 0.0963 | 0.9531 | NA |  |
| **Change from baseline on day 29** |  |  |  |  |  |
| n (Missing) | 39 (0) | 41 (0) | 39 (0) | 40 (1) |  |
| Mean (SD) | -0.13 (0.51) | -0.01 (0.42) | -0.01 (0.36) | -0.05 (0.63) | 0.6903 |
| Median | -0.00 | -0.00 | -0.00 | -0.00 |  |
| Min, Max | -1.4, 1.1 | -1.3, 1.0 | -0.8, 0.8 | -1.6, 1.8 |  |
| 95% CI | -0.29 – 0.04 | -0.14 – 0.12 | -0.12 – 0.11 | -0.25 – 0.16 |  |
| p-value (vs. baseline) § | 0.1319 | 0.8445 | 0.9195 | 0.6441 |  |
| p-value (vs. placebo) † | 0.5460 | 0.7763 | 0.7260 | NA |  |

* Baseline is defined as the last value before the first dose.

‡ Difference of cytokine’s level among the four groups was analyzed by the ANOVA.

† Difference of cytokine’s level compared with placebo group was analyzed by the independent samples t-test.

§ Difference of cytokine’s level compared with baseline in each group was analyzed by paired t-test.

Abbreviations: N = Number of patients in analysis set; n = Number of patients; SoC = Standard of Care; NA= Not Applicable.

Table S10.

Level of IL-15 post administration (log10 pg/mL)

|  | **Meplazumab + SoC** | | | **Placebo + Soc  (N=41)** | **p-value** ‡ |
| --- | --- | --- | --- | --- | --- |
|  | **0.12 mg/kg (N=39)** | **0.2 mg/kg (N=41)** | **0.3 mg/kg (N=39)** |  |  |
| **Baseline*** |  |  |  |  |  |
| n (Missing) | 39 (0) | 41 (0) | 36 (3) | 39 (2) |  |
| Mean (SD) | 0.76 (0.30) | 0.86 (0.39) | 0.68 (0.33) | 0.73 (0.32) | 0.1083 |
| Median | 0.80 | 0.89 | 0.77 | 0.77 |  |
| Min, Max | 0.2, 1.2 | 0.2, 2.0 | 0.2, 1.4 | 0.2, 1.3 |  |
| 95% CI | 0.66 – 0.85 | 0.74 – 0.99 | 0.57 – 0.79 | 0.62 – 0.83 |  |
| p-value (vs. placebo) † | 0.6757 | 0.0887 | 0.5606 | NA |  |
| **Day 2** |  |  |  |  |  |
| n (Missing) | 39 (0) | 41 (0) | 37 (2) | 40 (1) |  |
| Mean (SD) | 0.70 (0.31) | 0.84 (0.44) | 0.63 (0.36) | 0.66 (0.34) | 0.0567 |
| Median | 0.72 | 0.89 | 0.68 | 0.73 |  |
| Min, Max | 0.2, 1.3 | 0.2, 2.0 | 0.2, 1.3 | 0.2, 1.3 |  |
| 95% CI | 0.60 – 0.80 | 0.70 – 0.98 | 0.51 – 0.75 | 0.55 – 0.76 |  |
| p-value (vs. placebo) † | 0.5312 | 0.0381 | 0.7773 | NA |  |
| **Change from baseline on day 2** |  |  |  |  |  |
| n (Missing) | 39 (0) | 41 (0) | 36 (3) | 39 (2) |  |
| Mean (SD) | -0.05 (0.27) | -0.02 (0.30) | -0.05 (0.22) | -0.07 (0.27) | 0.9091 |
| Median | -0.00 | -0.00 | -0.00 | -0.06 |  |
| Min, Max | -0.6, 0.5 | -0.9, 0.7 | -0.6, 0.4 | -0.6, 0.6 |  |
| 95% CI | -0.14 – 0.03 | -0.12 – 0.07 | -0.12 – 0.03 | -0.16 – 0.02 |  |
| p-value (vs. baseline) § | 0.2175 | 0.6182 | 0.1999 | 0.1368 |  |
| p-value (vs. placebo) † | 0.8288 | 0.5055 | 0.7546 | NA |  |
| **Day 8** |  |  |  |  |  |
| n (Missing) | 39 (0) | 41 (0) | 37 (2) | 41 (0) |  |
| Mean (SD) | 0.59 (0.34) | 0.64 (0.47) | 0.56 (0.36) | 0.64 (0.35) | 0.7085 |
| Median | 0.57 | 0.61 | 0.60 | 0.73 |  |
| Min, Max | 0.2, 1.5 | 0.2, 2.0 | 0.2, 1.3 | 0.2, 1.3 |  |
| 95% CI | 0.47 – 0.70 | 0.50 – 0.79 | 0.44 – 0.68 | 0.53 – 0.75 |  |
| p-value (vs. placebo) † | 0.5058 | 0.9519 | 0.3117 | NA |  |
| **Change from baseline on day 8** |  |  |  |  |  |
| n (Missing) | 39 (0) | 41 (0) | 36 (3) | 39 (2) |  |
| Mean (SD) | -0.17 (0.37) | -0.22 (0.39) | -0.13 (0.30) | -0.10 (0.36) | 0.4872 |
| Median | -0.12 | -0.16 | -0.00 | -0.06 |  |
| Min, Max | -1.0, 0.4 | -1.6, 0.5 | -0.7, 0.5 | -0.7, 0.6 |  |
| 95% CI | -0.29 – -0.05 | -0.34 – -0.10 | -0.23 – -0.03 | -0.22 – 0.02 |  |
| p-value (vs. baseline) § | 0.0066 | 0.0009 | 0.0143 | 0.0858 |  |
| p-value (vs. placebo) † | 0.4173 | 0.1653 | 0.7197 | NA |  |
| **Day 9** |  |  |  |  |  |
| n (Missing) | 39 (0) | 41 (0) | 37 (2) | 41 (0) |  |
| Mean (SD) | 0.58 (0.33) | 0.67 (0.47) | 0.56 (0.34) | 0.57 (0.36) | 0.5383 |
| Median | 0.63 | 0.61 | 0.59 | 0.56 |  |
| Min, Max | 0.2, 1.3 | 0.2, 2.0 | 0.2, 1.2 | 0.2, 1.3 |  |
| 95% CI | 0.47 – 0.69 | 0.52 – 0.82 | 0.44 – 0.67 | 0.46 – 0.69 |  |
| p-value (vs. placebo) † | 0.9349 | 0.2955 | 0.8318 | NA |  |
| **Change from baseline on day 9** |  |  |  |  |  |
| n (Missing) | 39 (0) | 41 (0) | 36 (3) | 39 (2) |  |
| Mean (SD) | -0.17 (0.29) | -0.19 (0.37) | -0.13 (0.28) | -0.16 (0.36) | 0.8701 |
| Median | -0.13 | -0.20 | -0.09 | -0.14 |  |
| Min, Max | -0.9, 0.4 | -0.9, 0.5 | -0.7, 0.3 | -0.9, 0.6 |  |
| 95% CI | -0.27 – -0.08 | -0.31 – -0.07 | -0.22 – -0.04 | -0.28 – -0.05 |  |
| p-value (vs. baseline) § | 0.0005 | 0.0021 | 0.0078 | 0.0065 |  |
| p-value (vs. placebo) † | 0.8855 | 0.7339 | 0.6502 | NA |  |
| **Day 29** |  |  |  |  |  |
| n (Missing) | 39 (0) | 41 (0) | 38 (1) | 41 (0) |  |
| Mean (SD) | 0.49 (0.34) | 0.60 (0.47) | 0.44 (0.32) | 0.52 (0.40) | 0.3155 |
| Median | 0.51 | 0.51 | 0.20 | 0.20 |  |
| Min, Max | 0.2, 1.4 | 0.2, 1.5 | 0.2, 1.1 | 0.2, 1.6 |  |
| 95% CI | 0.38 – 0.60 | 0.45 – 0.75 | 0.34 – 0.55 | 0.39 – 0.64 |  |
| p-value (vs. placebo) † | 0.7541 | 0.3732 | 0.375 | NA |  |
| **Change from baseline on day 29** |  |  |  |  |  |
| n (Missing) | 39 (0) | 41 (0) | 36 (3) | 39 (2) |  |
| Mean (SD) | -0.27 (0.31) | -0.26 (0.44) | -0.23 (0.25) | -0.21 (0.42) | 0.9017 |
| Median | -0.14 | -0.26 | -0.20 | -0.20 |  |
| Min, Max | -0.9, 0.3 | -1.3, 0.5 | -0.7, 0.2 | -0.9, 0.9 |  |
| 95% CI | -0.37 – -0.17 | -0.40 – -0.12 | -0.31 – -0.14 | -0.35 – -0.08 |  |
| p-value (vs. baseline) § | <0.0001 | 0.0004 | <0.0001 | 0.0027 |  |
| p-value (vs. placebo) † | 0.5354 | 0.6223 | 0.8794 | NA |  |

* Baseline is defined as the last value before the first dose.

‡ Difference of cytokine’s level among the four groups was analyzed by the ANOVA.

† Difference of cytokine’s level compared with placebo group was analyzed by the independent samples t-test.

§ Difference of cytokine’s level compared with baseline in each group was analyzed by paired t-test.

Abbreviations: N = Number of patients in analysis set; n = Number of patients; SoC = Standard of Care; NA= Not Applicable.

Table S11.

Level of IL-17A post administration (log10 pg/mL)

|  | **Meplazumab + SoC** | | | **Placebo + Soc  (N=41)** | **p-value** ‡ |
| --- | --- | --- | --- | --- | --- |
|  | **0.12 mg/kg (N=39)** | **0.2 mg/kg (N=41)** | **0.3 mg/kg (N=39)** |  |  |
| **Baseline*** |  |  |  |  |  |
| n (Missing) | 39 (0) | 41 (0) | 39 (0) | 39 (2) |  |
| Mean (SD) | 0.75 (0.55) | 0.83 (0.61) | 0.70 (0.50) | 0.70 (0.58) | 0.6844 |
| Median | 0.65 | 0.80 | 0.65 | 0.59 |  |
| Min, Max | 0.2, 2.1 | 0.2, 2.5 | 0.2, 1.9 | 0.2, 2.0 |  |
| 95% CI | 0.57 – 0.93 | 0.64 – 1.02 | 0.53 – 0.86 | 0.51 – 0.89 |  |
| p-value (vs. placebo) † | 0.6934 | 0.3267 | 0.9885 | NA |  |
| **Day 2** |  |  |  |  |  |
| n (Missing) | 39 (0) | 41 (0) | 39 (0) | 41 (0) |  |
| Mean (SD) | 0.70 (0.51) | 0.83 (0.64) | 0.71 (0.46) | 0.74 (0.56) | 0.6921 |
| Median | 0.70 | 0.73 | 0.71 | 0.83 |  |
| Min, Max | 0.2, 2.1 | 0.2, 2.2 | 0.2, 1.8 | 0.2, 2.0 |  |
| 95% CI | 0.54 – 0.86 | 0.63 – 1.03 | 0.56 – 0.86 | 0.56 – 0.92 |  |
| p-value (vs. placebo) † | 0.7525 | 0.488 | 0.786 | NA |  |
| **Change from baseline on day 2** |  |  |  |  |  |
| n (Missing) | 39 (0) | 41 (0) | 39 (0) | 39 (2) |  |
| Mean (SD) | -0.05 (0.43) | 0.00 (0.27) | 0.01 (0.22) | 0.04 (0.40) | 0.7289 |
| Median | -0.00 | -0.00 | -0.00 | -0.00 |  |
| Min, Max | -1.4, 0.9 | -0.6, 0.5 | -0.5, 0.5 | -1.5, 0.9 |  |
| 95% CI | -0.19 – 0.09 | -0.08 – 0.08 | -0.06 – 0.08 | -0.09 – 0.17 |  |
| p-value (vs. baseline) § | 0.4786 | 0.9935 | 0.7868 | 0.5680 |  |
| p-value (vs. placebo) † | 0.3623 | 0.6317 | 0.7144 | NA |  |
| **Day 8** |  |  |  |  |  |
| n (Missing) | 39 (0) | 41 (0) | 39 (0) | 41 (0) |  |
| Mean (SD) | 0.79 (0.56) | 0.80 (0.63) | 0.69 (0.52) | 0.74 (0.51) | 0.8131 |
| Median | 0.77 | 0.63 | 0.63 | 0.72 |  |
| Min, Max | 0.2, 2.1 | 0.2, 2.1 | 0.2, 1.9 | 0.2, 1.8 |  |
| 95% CI | 0.61 – 0.97 | 0.60 – 1.00 | 0.53 – 0.86 | 0.58 – 0.90 |  |
| p-value (vs. placebo) † | 0.696 | 0.6312 | 0.6707 | NA |  |
| **Change from baseline on day 8** |  |  |  |  |  |
| n (Missing) | 39 (0) | 41 (0) | 39 (0) | 39 (2) |  |
| Mean (SD) | 0.04 (0.60) | -0.03 (0.43) | -0.00 (0.33) | 0.05 (0.47) | 0.8685 |
| Median | -0.00 | -0.00 | -0.00 | -0.00 |  |
| Min, Max | -1.4, 1.7 | -1.1, 0.9 | -0.9, 1.2 | -1.1, 1.2 |  |
| 95% CI | -0.16 – 0.23 | -0.16 – 0.11 | -0.11 – 0.10 | -0.10 – 0.20 |  |
| p-value (vs. baseline) § | 0.6874 | 0.6818 | 0.9404 | 0.5143 |  |
| p-value (vs. placebo) † | 0.9315 | 0.4443 | 0.5629 | NA |  |
| **Day 9** |  |  |  |  |  |
| n (Missing) | 39 (0) | 41 (0) | 39 (0) | 41 (0) |  |
| Mean (SD) | 0.73 (0.53) | 0.80 (0.65) | 0.73 (0.51) | 0.72 (0.52) | 0.9066 |
| Median | 0.79 | 0.63 | 0.68 | 0.71 |  |
| Min, Max | 0.2, 2.2 | 0.2, 2.3 | 0.2, 1.8 | 0.2, 1.8 |  |
| 95% CI | 0.56 – 0.90 | 0.59 – 1.01 | 0.57 – 0.90 | 0.55 – 0.88 |  |
| p-value (vs. placebo) † | 0.898 | 0.5209 | 0.8849 | NA |  |
| **Change from baseline on day 9** |  |  |  |  |  |
| n (Missing) | 39 (0) | 41 (0) | 39 (0) | 39 (2) |  |
| Mean (SD) | -0.02 (0.51) | -0.03 (0.39) | 0.04 (0.43) | 0.02 (0.51) | 0.9057 |
| Median | -0.00 | -0.00 | -0.00 | -0.00 |  |
| Min, Max | -1.4, 1.6 | -0.8, 0.7 | -0.6, 1.6 | -1.5, 1.1 |  |
| 95% CI | -0.18 – 0.15 | -0.15 – 0.09 | -0.10 – 0.18 | -0.14 – 0.19 |  |
| p-value (vs. baseline) § | 0.8314 | 0.6324 | 0.5975 | 0.7800 |  |
| p-value (vs. placebo) † | 0.7268 | 0.607 | 0.8988 | NA |  |
| **Day 29** |  |  |  |  |  |
| n (Missing) | 39 (0) | 41 (0) | 39 (0) | 41 (0) |  |
| Mean (SD) | 0.58 (0.50) | 0.84 (0.66) | 0.72 (0.51) | 0.70 (0.53) | 0.2160 |
| Median | 0.20 | 0.79 | 0.73 | 0.59 |  |
| Min, Max | 0.2, 2.2 | 0.2, 2.2 | 0.2, 2.0 | 0.2, 1.8 |  |
| 95% CI | 0.42 – 0.75 | 0.64 – 1.05 | 0.55 – 0.88 | 0.53 – 0.87 |  |
| p-value (vs. placebo) † | 0.3117 | 0.2751 | 0.8806 | NA |  |
| **Change from baseline on day 29** |  |  |  |  |  |
| n (Missing) | 39 (0) | 41 (0) | 39 (0) | 39 (2) |  |
| Mean (SD) | -0.17 (0.54) | 0.02 (0.29) | 0.02 (0.45) | 0.01 (0.53) | 0.2175 |
| Median | -0.00 | -0.00 | -0.00 | -0.00 |  |
| Min, Max | -1.4, 1.2 | -0.8, 0.6 | -0.9, 1.1 | -1.5, 0.9 |  |
| 95% CI | -0.34 – 0.01 | -0.08 – 0.11 | -0.12 – 0.17 | -0.16 – 0.18 |  |
| p-value (vs. baseline) § | 0.0636 | 0.7410 | 0.7697 | 0.9435 |  |
| p-value (vs. placebo) † | 0.1595 | 0.923 | 0.891 | NA |  |

* Baseline is defined as the last value before the first dose.

‡ Difference of cytokine’s level among the four groups was analyzed by the ANOVA.

† Difference of cytokine’s level compared with placebo group was analyzed by the independent samples t-test.

§ Difference of cytokine’s level compared with baseline in each group was analyzed by paired t-test.

Abbreviations: N = Number of patients in analysis set; n = Number of patients; SoC = Standard of Care; NA= Not Applicable.

Table S12.

Level of IL-1RA post administration (log10 pg/mL)

|  | **Meplazumab + SoC** | | | **Placebo + Soc  (N=41)** | **p-value** ‡ |
| --- | --- | --- | --- | --- | --- |
|  | **0.12 mg/kg (N=39)** | **0.2 mg/kg (N=41)** | **0.3 mg/kg (N=39)** |  |  |
| **Baseline*** |  |  |  |  |  |
| n (Missing) | 38 (1) | 40 (1) | 38 (1) | 40 (1) |  |
| Mean (SD) | 3.42 (0.26) | 3.48 (0.35) | 3.50 (0.28) | 3.44 (0.33) | 0.6358 |
| Median | 3.42 | 3.53 | 3.45 | 3.51 |  |
| Min, Max | 3.0, 4.0 | 2.7, 4.3 | 2.9, 4.1 | 2.8, 4.2 |  |
| 95% CI | 3.33 – 3.50 | 3.37 – 3.59 | 3.41 – 3.59 | 3.34 – 3.55 |  |
| p-value (vs. placebo) † | 0.6613 | 0.6699 | 0.4172 | NA |  |
| **Day 2** |  |  |  |  |  |
| n (Missing) | 39 (0) | 41 (0) | 39 (0) | 41 (0) |  |
| Mean (SD) | 3.42 (0.30) | 3.41 (0.33) | 3.51 (0.29) | 3.31 (0.35) | 0.0590 |
| Median | 3.38 | 3.39 | 3.49 | 3.28 |  |
| Min, Max | 2.9, 4.2 | 2.8, 4.5 | 2.8, 4.1 | 2.7, 4.2 |  |
| 95% CI | 3.33 – 3.52 | 3.31 – 3.52 | 3.42 – 3.60 | 3.21 – 3.42 |  |
| p-value (vs. placebo) † | 0.136 | 0.1938 | 0.0075 | NA |  |
| **Change from baseline on day 2** |  |  |  |  |  |
| n (Missing) | 38 (1) | 40 (1) | 38 (1) | 40 (1) |  |
| Mean (SD) | 0.01 (0.25) | -0.06 (0.34) | 0.01 (0.23) | -0.13 (0.32) | 0.0735 |
| Median | -0.03 | -0.03 | 0.02 | -0.11 |  |
| Min, Max | -0.5, 0.7 | -0.8, 0.6 | -0.6, 0.4 | -0.9, 0.6 |  |
| 95% CI | -0.07 – 0.10 | -0.17 – 0.05 | -0.06 – 0.09 | -0.24 – -0.03 |  |
| p-value (vs. baseline) § | 0.7376 | 0.2462 | 0.7020 | 0.0110 |  |
| p-value (vs. placebo) † | 0.0263 | 0.3443 | 0.0205 | NA |  |
| **Day 8** |  |  |  |  |  |
| n (Missing) | 39 (0) | 41 (0) | 39 (0) | 41 (0) |  |
| Mean (SD) | 3.40 (0.29) | 3.42 (0.42) | 3.45 (0.36) | 3.35 (0.41) | 0.6338 |
| Median | 3.36 | 3.37 | 3.40 | 3.25 |  |
| Min, Max | 2.8, 4.2 | 2.6, 4.6 | 2.7, 4.3 | 2.7, 4.5 |  |
| 95% CI | 3.30 – 3.49 | 3.28 – 3.55 | 3.34 – 3.57 | 3.21 – 3.48 |  |
| p-value (vs. placebo) † | 0.5311 | 0.4474 | 0.2192 | NA |  |
| **Change from baseline on day 8** |  |  |  |  |  |
| n (Missing) | 38 (1) | 40 (1) | 38 (1) | 40 (1) |  |
| Mean (SD) | -0.02 (0.29) | -0.06 (0.52) | -0.04 (0.37) | -0.10 (0.42) | 0.8525 |
| Median | -0.01 | -0.10 | -0.03 | -0.07 |  |
| Min, Max | -0.7, 0.7 | -1.0, 1.6 | -0.9, 0.9 | -0.9, 1.4 |  |
| 95% CI | -0.11 – 0.08 | -0.23 – 0.11 | -0.17 – 0.08 | -0.23 – 0.04 |  |
| p-value (vs. baseline) § | 0.7485 | 0.4732 | 0.4737 | 0.1572 |  |
| P-value (vs. placebo) † | 0.3294 | 0.7344 | 0.5609 | NA |  |
| **Day 9** |  |  |  |  |  |
| n (Missing) | 39 (0) | 41 (0) | 39 (0) | 41 (0) |  |
| Mean (SD) | 3.43 (0.42) | 3.39 (0.39) | 3.49 (0.30) | 3.35 (0.39) | 0.4025 |
| Median | 3.35 | 3.35 | 3.44 | 3.28 |  |
| Min, Max | 2.9, 5.1 | 2.6, 4.5 | 2.9, 4.0 | 2.8, 4.5 |  |
| 95% CI | 3.29 – 3.56 | 3.27 – 3.52 | 3.40 – 3.59 | 3.23 – 3.47 |  |
| p-value (vs. placebo) † | 0.4024 | 0.6259 | 0.0746 | NA |  |
| **Change from baseline on day 9** |  |  |  |  |  |
| n (Missing) | 38 (1) | 40 (1) | 38 (1) | 40 (1) |  |
| Mean (SD) | 0.02 (0.42) | -0.09 (0.55) | -0.01 (0.28) | -0.09 (0.39) | 0.5256 |
| Median | -0.01 | -0.13 | -0.02 | -0.12 |  |
| Min, Max | -0.8, 1.8 | -1.0, 1.3 | -0.8, 0.7 | -1.0, 0.8 |  |
| 95% CI | -0.12 – 0.16 | -0.26 – 0.09 | -0.10 – 0.09 | -0.22 – 0.03 |  |
| p-value (vs. baseline) § | 0.7494 | 0.3133 | 0.8985 | 0.1373 |  |
| p-value (vs. placebo) † | 0.2107 | 0.9637 | 0.258 | NA |  |
| **Day 29** |  |  |  |  |  |
| n (Missing) | 39 (0) | 41 (0) | 39 (0) | 41 (0) |  |
| Mean (SD) | 3.32 (0.42) | 3.21 (0.41) | 3.37 (0.33) | 3.21 (0.38) | 0.1627 |
| Median | 3.21 | 3.08 | 3.31 | 3.12 |  |
| Min, Max | 2.7, 5.0 | 2.6, 4.5 | 2.8, 4.0 | 2.6, 4.2 |  |
| 95% CI | 3.18 – 3.46 | 3.08 – 3.33 | 3.26 – 3.47 | 3.09 – 3.33 |  |
| p-value (vs. placebo) † | 0.2281 | 0.9643 | 0.0533 | NA |  |
| **Change from baseline on day 29** |  |  |  |  |  |
| n (Missing) | 38 (1) | 40 (1) | 38 (1) | 40 (1) |  |
| Mean (SD) | -0.09 (0.43) | -0.27 (0.57) | -0.13 (0.31) | -0.23 (0.40) | 0.2323 |
| Median | -0.13 | -0.34 | -0.11 | -0.21 |  |
| Min, Max | -1.0, 1.2 | -1.3, 1.3 | -1.1, 0.4 | -1.0, 0.8 |  |
| 95% CI | -0.23 – 0.05 | -0.45 – -0.09 | -0.23 – -0.03 | -0.35 – -0.10 |  |
| p-value (vs. baseline) § | 0.2067 | 0.0047 | 0.0151 | 0.0008 |  |
| p-value (vs. placebo) † | 0.1456 | 0.6878 | 0.2273 | NA |  |

* Baseline is defined as the last value before the first dose.

‡ Difference of cytokine’s level among the four groups was analyzed by the ANOVA.

† Difference of cytokine’s level compared with placebo group was analyzed by the independent samples t-test.

§ Difference of cytokine’s level compared with baseline in each group was analyzed by paired t-test.

Abbreviations: N = Number of patients in analysis set; n = Number of patients; SoC = Standard of Care; NA= Not Applicable.

Table S13.

Level of IL-2Rα post administration (log10 pg/mL)

|  | **Meplazumab + SoC** | | | **Placebo + Soc  (N=41)** | **p-value** ‡ |
| --- | --- | --- | --- | --- | --- |
|  | **0.12 mg/kg (N=39)** | **0.2 mg/kg (N=41)** | **0.3 mg/kg (N=39)** |  |  |
| **Baseline*** |  |  |  |  |  |
| n (Missing) | 38 (1) | 40 (1) | 38 (1) | 40 (1) |  |
| Mean (SD) | 3.03 (0.17) | 3.04 (0.18) | 3.00 (0.16) | 3.03 (0.19) | 0.8079 |
| Median | 3.03 | 3.05 | 2.97 | 2.99 |  |
| Min, Max | 2.8, 3.5 | 2.6, 3.3 | 2.7, 3.4 | 2.6, 3.5 |  |
| 95% CI | 2.97 – 3.08 | 2.98 – 3.09 | 2.95 – 3.05 | 2.97 – 3.09 |  |
| p-value (vs. placebo) † | 0.99 | 0.8501 | 0.4703 | NA |  |
| **Day 2** |  |  |  |  |  |
| n (Missing) | 39 (0) | 41 (0) | 39 (0) | 41 (0) |  |
| Mean (SD) | 3.01 (0.17) | 3.05 (0.20) | 3.04 (0.17) | 3.03 (0.20) | 0.8525 |
| Median | 3.00 | 3.08 | 3.04 | 3.02 |  |
| Min, Max | 2.7, 3.4 | 2.5, 3.3 | 2.6, 3.4 | 2.5, 3.6 |  |
| 95% CI | 2.96 – 3.06 | 2.98 – 3.11 | 2.98 – 3.09 | 2.97 – 3.09 |  |
| p-value (vs. placebo) † | 0.628 | 0.7298 | 0.8717 | NA |  |
| **Change from baseline on day 2** |  |  |  |  |  |
| n (Missing) | 38 (1) | 40 (1) | 38 (1) | 40 (1) |  |
| Mean (SD) | -0.02 (0.13) | 0.00 (0.08) | 0.03 (0.10) | 0.00 (0.13) | 0.2351 |
| Median | -0.02 | 0.00 | 0.01 | -0.01 |  |
| Min, Max | -0.4, 0.3 | -0.2, 0.2 | -0.2, 0.2 | -0.2, 0.7 |  |
| 95% CI | -0.07 – 0.02 | -0.02 – 0.03 | 0.00 – 0.06 | -0.04 – 0.04 |  |
| p-value (vs. baseline) § | 0.3439 | 0.7668 | 0.0499 | 0.9799 |  |
| p-value (vs. placebo) † | 0.4703 | 0.8949 | 0.2292 | NA |  |
| **Day 8** |  |  |  |  |  |
| n (Missing) | 39 (0) | 41 (0) | 39 (0) | 41 (0) |  |
| Mean (SD) | 2.94 (0.20) | 3.00 (0.21) | 2.96 (0.17) | 3.00 (0.21) | 0.4631 |
| Median | 2.94 | 3.01 | 2.95 | 2.98 |  |
| Min, Max | 2.6, 3.4 | 2.5, 3.6 | 2.6, 3.3 | 2.5, 3.6 |  |
| 95% CI | 2.87 – 3.00 | 2.93 – 3.07 | 2.90 – 3.01 | 2.93 – 3.06 |  |
| p-value (vs. placebo) † | 0.2232 | 0.9457 | 0.3866 | NA |  |
| **Change from baseline on day 8** |  |  |  |  |  |
| n (Missing) | 38 (1) | 40 (1) | 38 (1) | 40 (1) |  |
| Mean (SD) | -0.09 (0.16) | -0.04 (0.14) | -0.05 (0.14) | -0.03 (0.18) | 0.3911 |
| Median | -0.09 | -0.05 | -0.05 | -0.05 |  |
| Min, Max | -0.4, 0.3 | -0.3, 0.4 | -0.4, 0.2 | -0.4, 0.7 |  |
| 95% CI | -0.14 – -0.04 | -0.09 – 0.00 | -0.10 – -0.00 | -0.09 – 0.03 |  |
| p-value (vs. baseline) § | 0.0015 | 0.0782 | 0.0306 | 0.2686 |  |
| p-value (vs. placebo) † | 0.1406 | 0.7981 | 0.6144 | NA |  |
| **Day 9** |  |  |  |  |  |
| n (Missing) | 39 (0) | 41 (0) | 39 (0) | 41 (0) |  |
| Mean (SD) | 2.93 (0.22) | 2.99 (0.21) | 2.94 (0.18) | 2.98 (0.20) | 0.4643 |
| Median | 2.92 | 3.00 | 2.93 | 2.96 |  |
| Min, Max | 2.5, 3.5 | 2.4, 3.5 | 2.6, 3.3 | 2.5, 3.5 |  |
| 95% CI | 2.86 – 3.00 | 2.92 – 3.05 | 2.88 – 3.00 | 2.92 – 3.04 |  |
| p-value (vs. placebo) † | 0.266 | 0.8538 | 0.3374 | NA |  |
| **Change from baseline on day 9** |  |  |  |  |  |
| n (Missing) | 38 (1) | 40 (1) | 38 (1) | 40 (1) |  |
| Mean (SD) | -0.10 (0.18) | -0.05 (0.15) | -0.07 (0.14) | -0.05 (0.17) | 0.4425 |
| Median | -0.11 | -0.08 | -0.09 | -0.06 |  |
| Min, Max | -0.4, 0.5 | -0.3, 0.3 | -0.4, 0.2 | -0.4, 0.6 |  |
| 95% CI | -0.17 – -0.04 | -0.10 – -0.01 | -0.12 – -0.03 | -0.11 – 0.00 |  |
| p-value (vs. baseline) § | 0.0012 | 0.0258 | 0.0029 | 0.0684 |  |
| p-value (vs. placebo) † | 0.1831 | 0.9183 | 0.5579 | NA |  |
| **Day 29** |  |  |  |  |  |
| n (Missing) | 39 (0) | 41 (0) | 39 (0) | 41 (0) |  |
| Mean (SD) | 2.85 (0.22) | 2.87 (0.23) | 2.84 (0.19) | 2.90 (0.20) | 0.5725 |
| Median | 2.81 | 2.90 | 2.80 | 2.89 |  |
| Min, Max | 2.5, 3.7 | 2.3, 3.4 | 2.5, 3.3 | 2.4, 3.5 |  |
| 95% CI | 2.78 – 2.92 | 2.80 – 2.94 | 2.78 – 2.90 | 2.84 – 2.97 |  |
| p-value (vs. placebo) † | 0.2495 | 0.4577 | 0.1762 | NA |  |
| **Change from baseline on day 29** |  |  |  |  |  |
| n (Missing) | 38 (1) | 40 (1) | 38 (1) | 40 (1) |  |
| Mean (SD) | -0.18 (0.20) | -0.17 (0.21) | -0.16 (0.13) | -0.12 (0.20) | 0.4773 |
| Median | -0.21 | -0.20 | -0.15 | -0.13 |  |
| Min, Max | -0.5, 0.4 | -0.7, 0.3 | -0.5, 0.1 | -0.5, 0.6 |  |
| 95% CI | -0.25 – -0.12 | -0.24 – -0.10 | -0.20 – -0.12 | -0.18 – -0.06 |  |
| p-value (vs. baseline) § | <0.0001 | <0.0001 | <0.0001 | 0.0005 |  |
| p-value (vs. placebo) † | 0.1644 | 0.281 | 0.2999 | NA |  |

* Baseline is defined as the last value before the first dose.

‡ Difference of cytokine’s level among the four groups was analyzed by the ANOVA.

† Difference of cytokine’s level compared with placebo group was analyzed by the independent samples t-test.

§ Difference of cytokine’s level compared with baseline in each group was analyzed by paired t-test.

Abbreviations: N = Number of patients in analysis set; n = Number of patients; SoC = Standard of Care; NA= Not Applicable.

Table S14.

Level of MCP-1 post administration (log10 pg/mL)

|  | **Meplazumab + SoC** | | | **Placebo + Soc  (N=41)** | **p-value** ‡ |
| --- | --- | --- | --- | --- | --- |
|  | **0.12 mg/kg (N=39)** | **0.2 mg/kg (N=41)** | **0.3 mg/kg (N=39)** |  |  |
| **Baseline*** |  |  |  |  |  |
| n (Missing) | 34 (5) | 38 (3) | 31 (8) | 36 (5) |  |
| Mean (SD) | 2.67 (0.28) | 2.90 (0.31) | 2.80 (0.30) | 2.87 (0.25) | 0.0062 |
| Median | 2.63 | 2.88 | 2.73 | 2.85 |  |
| Min, Max | 2.2, 3.2 | 2.4, 3.5 | 2.3, 3.4 | 2.3, 3.5 |  |
| 95% CI | 2.58 – 2.77 | 2.80 – 3.00 | 2.69 – 2.91 | 2.79 – 2.96 |  |
| p-value (vs. placebo) † | 0.0031 | 0.6911 | 0.2758 | NA |  |
| **Day 2** |  |  |  |  |  |
| n (Missing) | 38 (1) | 41 (0) | 35 (4) | 38 (3) |  |
| Mean (SD) | 2.68 (0.30) | 2.84 (0.32) | 2.78 (0.33) | 2.88 (0.32) | 0.0390 |
| Median | 2.69 | 2.82 | 2.85 | 2.88 |  |
| Min, Max | 2.2, 3.3 | 2.3, 3.5 | 2.1, 3.5 | 2.2, 3.9 |  |
| 95% CI | 2.59 – 2.78 | 2.74 – 2.94 | 2.67 – 2.89 | 2.78 – 2.99 |  |
| p-value (vs. placebo) † | 0.0063 | 0.5471 | 0.1913 | NA |  |
| **Change from baseline on day 2** |  |  |  |  |  |
| n (Missing) | 34 (5) | 38 (3) | 31 (8) | 36 (5) |  |
| Mean (SD) | 0.01 (0.19) | -0.07 (0.24) | -0.01 (0.20) | 0.01 (0.33) | 0.4689 |
| Median | -0.00 | -0.00 | -0.00 | -0.00 |  |
| Min, Max | -0.5, 0.6 | -0.6, 0.3 | -0.5, 0.5 | -0.9, 1.1 |  |
| 95% CI | -0.06 – 0.07 | -0.15 – 0.01 | -0.09 – 0.06 | -0.10 – 0.12 |  |
| p-value (vs. baseline) § | 0.8102 | 0.0808 | 0.7267 | 0.8342 |  |
| p-value (vs. placebo) † | 0.9535 | 0.2259 | 0.7211 | NA |  |
| **Day 8** |  |  |  |  |  |
| n (Missing) | 39 (0) | 41 (0) | 37 (2) | 40 (1) |  |
| Mean (SD) | 2.71 (0.35) | 2.75 (0.31) | 2.74 (0.34) | 2.80 (0.36) | 0.6712 |
| Median | 2.66 | 2.74 | 2.71 | 2.81 |  |
| Min, Max | 2.2, 4.0 | 2.1, 3.4 | 2.1, 3.5 | 2.2, 4.1 |  |
| 95% CI | 2.60 – 2.82 | 2.65 – 2.84 | 2.62 – 2.85 | 2.69 – 2.92 |  |
| p-value (vs. placebo) † | 0.2492 | 0.4435 | 0.4121 | NA |  |
| **Change from baseline on day 8** |  |  |  |  |  |
| n (Missing) | 34 (5) | 38 (3) | 31 (8) | 36 (5) |  |
| Mean (SD) | 0.02 (0.41) | -0.14 (0.32) | -0.06 (0.36) | -0.07 (0.39) | 0.3331 |
| Median | -0.09 | -0.09 | -0.05 | -0.06 |  |
| Min, Max | -0.4, 1.7 | -0.8, 0.8 | -0.9, 0.9 | -0.9, 1.2 |  |
| 95% CI | -0.12 – 0.17 | -0.24 – -0.04 | -0.20 – 0.07 | -0.20 – 0.06 |  |
| p-value (vs. baseline) § | 0.7491 | 0.0099 | 0.3413 | 0.3077 |  |
| p-value (vs. placebo) † | 0.3509 | 0.3837 | 0.9625 | NA |  |
| **Day 9** |  |  |  |  |  |
| n (Missing) | 39 (0) | 41 (0) | 37 (2) | 41 (0) |  |
| Mean (SD) | 2.74 (0.38) | 2.75 (0.25) | 2.68 (0.30) | 2.80 (0.29) | 0.4437 |
| Median | 2.68 | 2.76 | 2.71 | 2.81 |  |
| Min, Max | 2.2, 4.0 | 2.3, 3.3 | 2.0, 3.3 | 2.1, 3.6 |  |
| 95% CI | 2.61 – 2.86 | 2.67 – 2.83 | 2.59 – 2.78 | 2.71 – 2.89 |  |
| p-value (vs. placebo) † | 0.4198 | 0.4489 | 0.0925 | NA |  |
| **Change from baseline on day 9** |  |  |  |  |  |
| n (Missing) | 34 (5) | 38 (3) | 31 (8) | 36 (5) |  |
| Mean (SD) | 0.05 (0.43) | -0.15 (0.32) | -0.12 (0.29) | -0.08 (0.31) | 0.0802 |
| Median | -0.06 | -0.14 | -0.05 | -0.05 |  |
| Min, Max | -0.6, 1.7 | -0.7, 0.7 | -0.9, 0.4 | -0.8, 0.8 |  |
| 95% CI | -0.10 – 0.20 | -0.25 – -0.04 | -0.22 – -0.01 | -0.18 – 0.03 |  |
| p-value (vs. baseline) § | 0.4987 | 0.0064 | 0.0290 | 0.1517 |  |
| p-value (vs. placebo) † | 0.1621 | 0.3193 | 0.5595 | NA |  |
| **Day 29** |  |  |  |  |  |
| n (Missing) | 39 (0) | 41 (0) | 38 (1) | 41 (0) |  |
| Mean (SD) | 2.73 (0.21) | 2.81 (0.25) | 2.69 (0.23) | 2.76 (0.27) | 0.1646 |
| Median | 2.75 | 2.81 | 2.70 | 2.76 |  |
| Min, Max | 2.2, 3.2 | 2.4, 3.5 | 2.1, 3.3 | 2.1, 3.6 |  |
| 95% CI | 2.66 – 2.80 | 2.73 – 2.89 | 2.61 – 2.77 | 2.67 – 2.85 |  |
| p-value (vs. placebo) † | 0.6164 | 0.3674 | 0.2378 | NA |  |
| **Change from baseline on day 29** |  |  |  |  |  |
| n (Missing) | 34 (5) | 38 (3) | 31 (8) | 36 (5) |  |
| Mean (SD) | 0.05 (0.34) | -0.08 (0.33) | -0.14 (0.30) | -0.13 (0.29) | 0.0646 |
| Median | 0.03 | -0.02 | -0.08 | -0.11 |  |
| Min, Max | -0.6, 0.8 | -0.7, 0.7 | -0.9, 0.3 | -0.8, 0.5 |  |
| 95% CI | -0.07 – 0.17 | -0.19 – 0.03 | -0.25 – -0.03 | -0.23 – -0.03 |  |
| p-value (vs. baseline) § | 0.4230 | 0.1353 | 0.0149 | 0.0110 |  |
| p-value (vs. placebo) † | 0.0212 | 0.5125 | 0.9247 | NA |  |

* Baseline is defined as the last value before the first dose.

‡ Difference of cytokine’s level among the four groups was analyzed by the ANOVA.

† Difference of cytokine’s level compared with placebo group was analyzed by the independent samples t-test.

§ Difference of cytokine’s level compared with baseline in each group was analyzed by paired t-test.

Abbreviations: N = Number of patients in analysis set; n = Number of patients; SoC = Standard of Care; NA= Not Applicable.

Table S15.

Level of MIP-1β post administration (log10 pg/mL)

|  | **Meplazumab + SoC** | | | **Placebo + Soc  (N=41)** | **p-value** ‡ |
| --- | --- | --- | --- | --- | --- |
|  | **0.12 mg/kg (N=39)** | **0.2 mg/kg (N=41)** | **0.3 mg/kg (N=39)** |  |  |
| **Baseline*** |  |  |  |  |  |
| n (Missing) | 38 (1) | 41 (0) | 39 (0) | 40 (1) |  |
| Mean (SD) | 1.64 (0.34) | 1.72 (0.29) | 1.73 (0.33) | 1.62 (0.29) | 0.2898 |
| Median | 1.62 | 1.68 | 1.76 | 1.64 |  |
| Min, Max | 0.9, 2.4 | 0.9, 2.4 | 0.9, 2.3 | 0.9, 2.2 |  |
| 95% CI | 1.53 – 1.75 | 1.63 – 1.81 | 1.62 – 1.84 | 1.52 – 1.71 |  |
| p-value (vs. placebo) † | 0.7421 | 0.1257 | 0.111 | NA |  |
| **Day 2** |  |  |  |  |  |
| n (Missing) | 39 (0) | 41 (0) | 39 (0) | 41 (0) |  |
| Mean (SD) | 1.75 (0.33) | 1.79 (0.28) | 1.84 (0.34) | 1.69 (0.33) | 0.2074 |
| Median | 1.72 | 1.78 | 1.83 | 1.68 |  |
| Min, Max | 0.9, 2.8 | 0.9, 2.5 | 0.9, 2.6 | 0.9, 2.7 |  |
| 95% CI | 1.65 – 1.86 | 1.71 – 1.88 | 1.73 – 1.95 | 1.59 – 1.80 |  |
| p-value (vs. placebo) † | 0.4118 | 0.1423 | 0.0529 | NA |  |
| **Change from baseline on day 2** |  |  |  |  |  |
| n (Missing) | 38 (1) | 41 (0) | 39 (0) | 40 (1) |  |
| Mean (SD) | 0.14 (0.39) | 0.08 (0.33) | 0.11 (0.30) | 0.07 (0.30) | 0.8053 |
| Median | 0.14 | 0.09 | 0.04 | 0.00 |  |
| Min, Max | -0.7, 1.0 | -0.7, 0.6 | -0.4, 1.0 | -0.9, 1.0 |  |
| 95% CI | 0.01 – 0.26 | -0.03 – 0.18 | 0.01 – 0.21 | -0.02 – 0.17 |  |
| p-value (vs. baseline) § | 0.0370 | 0.1380 | 0.0250 | 0.1312 |  |
| p-value (vs. placebo) † | 0.4167 | 0.9467 | 0.5627 | NA |  |
| **Day 8** |  |  |  |  |  |
| n (Missing) | 39 (0) | 41 (0) | 39 (0) | 41 (0) |  |
| Mean (SD) | 1.90 (0.39) | 1.91 (0.23) | 1.88 (0.27) | 1.69 (0.32) | 0.0042 |
| Median | 1.78 | 1.94 | 1.83 | 1.70 |  |
| Min, Max | 1.3, 3.2 | 1.4, 2.4 | 1.3, 2.6 | 0.9, 2.2 |  |
| 95% CI | 1.77 – 2.03 | 1.83 – 1.98 | 1.79 – 1.97 | 1.59 – 1.79 |  |
| p-value (vs. placebo) † | 0.011 | 0.0007 | 0.0055 | NA |  |
| **Change from baseline on day 8** |  |  |  |  |  |
| n (Missing) | 38 (1) | 41 (0) | 39 (0) | 40 (1) |  |
| Mean (SD) | 0.27 (0.53) | 0.19 (0.31) | 0.15 (0.34) | 0.06 (0.34) | 0.1390 |
| Median | 0.23 | 0.24 | 0.16 | 0.00 |  |
| Min, Max | -0.9, 2.3 | -0.6, 0.8 | -0.7, 1.2 | -0.9, 1.2 |  |
| 95% CI | 0.09 – 0.44 | 0.10 – 0.29 | 0.04 – 0.26 | -0.04 – 0.17 |  |
| p-value (vs. baseline) § | 0.0035 | 0.0003 | 0.0100 | 0.2291 |  |
| p-value (vs. placebo) † | 0.0464 | 0.0783 | 0.2727 | NA |  |
| **Day 9** |  |  |  |  |  |
| n (Missing) | 39 (0) | 41 (0) | 39 (0) | 41 (0) |  |
| Mean (SD) | 1.90 (0.35) | 1.86 (0.26) | 1.89 (0.29) | 1.73 (0.37) | 0.0682 |
| Median | 1.79 | 1.87 | 1.90 | 1.72 |  |
| Min, Max | 1.4, 3.1 | 0.9, 2.2 | 1.3, 2.6 | 0.9, 2.6 |  |
| 95% CI | 1.78 – 2.01 | 1.78 – 1.94 | 1.80 – 1.98 | 1.61 – 1.85 |  |
| p-value (vs. placebo) † | 0.0427 | 0.0637 | 0.0326 | NA |  |
| **Change from baseline on day 9** |  |  |  |  |  |
| n (Missing) | 38 (1) | 41 (0) | 39 (0) | 40 (1) |  |
| Mean (SD) | 0.26 (0.49) | 0.14 (0.33) | 0.16 (0.34) | 0.11 (0.38) | 0.3365 |
| Median | 0.21 | 0.21 | 0.14 | 0.06 |  |
| Min, Max | -1.0, 1.8 | -1.3, 0.6 | -0.5, 1.1 | -0.9, 1.2 |  |
| 95% CI | 0.10 – 0.43 | 0.04 – 0.25 | 0.05 – 0.27 | -0.01 – 0.23 |  |
| p-value (vs. baseline) § | 0.0021 | 0.0082 | 0.0051 | 0.0833 |  |
| p-value (vs. placebo) † | 0.1204 | 0.6415 | 0.5095 | NA |  |
| **Day 29** |  |  |  |  |  |
| n (Missing) | 39 (0) | 41 (0) | 39 (0) | 41 (0) |  |
| Mean (SD) | 1.68 (0.33) | 1.68 (0.26) | 1.76 (0.35) | 1.62 (0.24) | 0.2290 |
| Median | 1.62 | 1.68 | 1.72 | 1.62 |  |
| Min, Max | 0.9, 2.7 | 0.9, 2.2 | 0.9, 3.0 | 1.2, 2.2 |  |
| 95% CI | 1.57 – 1.78 | 1.60 – 1.77 | 1.65 – 1.88 | 1.55 – 1.70 |  |
| p-value (vs. placebo) † | 0.4124 | 0.2907 | 0.0427 | NA |  |
| **Change from baseline on day 29** |  |  |  |  |  |
| n (Missing) | 38 (1) | 41 (0) | 39 (0) | 40 (1) |  |
| Mean (SD) | 0.04 (0.46) | -0.03 (0.41) | 0.03 (0.35) | -0.01 (0.31) | 0.7936 |
| Median | -0.01 | -0.02 | -0.00 | -0.05 |  |
| Min, Max | -1.0, 1.3 | -1.3, 0.6 | -0.5, 1.0 | -0.4, 1.2 |  |
| 95% CI | -0.11 – 0.20 | -0.16 – 0.09 | -0.08 – 0.14 | -0.11 – 0.09 |  |
| p-value (vs. baseline) § | 0.5607 | 0.5946 | 0.5666 | 0.8930 |  |
| p-value (vs. placebo) † | 0.5702 | 0.7342 | 0.6027 | NA |  |

* Baseline is defined as the last value before the first dose.

‡ Difference of cytokine’s level among the four groups was analyzed by the ANOVA.

† Difference of cytokine’s level compared with placebo group was analyzed by the independent samples t-test.

§ Difference of cytokine’s level compared with baseline in each group was analyzed by paired t-test.

Abbreviations: N = Number of patients in analysis set; n = Number of patients; SoC = Standard of Care; NA= Not Applicable.

Table S16.

Level of IP-10 post administration (log10 pg/mL)

|  | **Meplazumab + SoC** | | | **Placebo + Soc  (N=41)** | **p-value** ‡ |
| --- | --- | --- | --- | --- | --- |
|  | **0.12 mg/kg (N=39)** | **0.2 mg/kg (N=41)** | **0.3 mg/kg (N=39)** |  |  |
| **Baseline*** |  |  |  |  |  |
| n (Missing) | 39 (0) | 41 (0) | 39 (0) | 40 (1) |  |
| Mean (SD) | 2.94 (0.47) | 3.10 (0.41) | 3.08 (0.38) | 3.13 (0.32) | 0.1857 |
| Median | 2.91 | 3.08 | 3.01 | 3.08 |  |
| Min, Max | 1.9, 3.8 | 2.4, 3.9 | 2.3, 3.9 | 2.4, 3.7 |  |
| 95% CI | 2.79 – 3.10 | 2.97 – 3.22 | 2.96 – 3.20 | 3.03 – 3.23 |  |
| p-value (vs. placebo) † | 0.0463 | 0.707 | 0.5481 | NA |  |
| **Day 2** |  |  |  |  |  |
| n (Missing) | 39 (0) | 41 (0) | 39 (0) | 41 (0) |  |
| Mean (SD) | 2.78 (0.46) | 2.91 (0.44) | 2.99 (0.38) | 2.94 (0.29) | 0.0979 |
| Median | 2.71 | 2.89 | 2.97 | 2.92 |  |
| Min, Max | 2.0, 3.7 | 2.1, 3.9 | 2.1, 4.0 | 2.3, 3.6 |  |
| 95% CI | 2.63 – 2.93 | 2.78 – 3.05 | 2.87 – 3.12 | 2.85 – 3.03 |  |
| p-value (vs. placebo) † | 0.0618 | 0.7631 | 0.4593 | NA |  |
| **Change from baseline on day 2** |  |  |  |  |  |
| n (Missing) | 39 (0) | 41 (0) | 39 (0) | 40 (1) |  |
| Mean (SD) | -0.17 (0.34) | -0.18 (0.32) | -0.09 (0.25) | -0.19 (0.28) | 0.3967 |
| Median | -0.16 | -0.18 | -0.06 | -0.14 |  |
| Min, Max | -0.9, 0.8 | -0.9, 0.5 | -0.6, 0.6 | -0.8, 0.4 |  |
| 95% CI | -0.28 – -0.06 | -0.28 – -0.08 | -0.17 – -0.00 | -0.28 – -0.10 |  |
| p-value (vs. baseline) § | 0.0040 | 0.0008 | 0.0422 | 0.0001 |  |
| p-value (vs. placebo) † | 0.7646 | 0.9208 | 0.0887 | NA |  |
| **Day 8** |  |  |  |  |  |
| n (Missing) | 39 (0) | 41 (0) | 39 (0) | 41 (0) |  |
| Mean (SD) | 2.53 (0.48) | 2.46 (0.50) | 2.53 (0.36) | 2.65 (0.42) | 0.2778 |
| Median | 2.42 | 2.42 | 2.47 | 2.65 |  |
| Min, Max | 1.9, 3.7 | 1.5, 3.9 | 1.8, 3.4 | 1.9, 3.8 |  |
| 95% CI | 2.37 – 2.69 | 2.30 – 2.62 | 2.41 – 2.65 | 2.52 – 2.78 |  |
| p-value (vs. placebo) † | 0.2342 | 0.0658 | 0.1758 | NA |  |
| **Change from baseline on day 8** |  |  |  |  |  |
| n (Missing) | 39 (0) | 41 (0) | 39 (0) | 40 (1) |  |
| Mean (SD) | -0.41 (0.41) | -0.63 (0.49) | -0.55 (0.41) | -0.46 (0.42) | 0.1183 |
| Median | -0.34 | -0.67 | -0.55 | -0.38 |  |
| Min, Max | -1.5, 0.4 | -1.6, 0.6 | -1.5, 0.3 | -1.3, 0.6 |  |
| 95% CI | -0.55 – -0.28 | -0.79 – -0.48 | -0.68 – -0.41 | -0.60 – -0.33 |  |
| p-value (vs. baseline) § | <0.0001 | <0.0001 | <0.0001 | <0.0001 |  |
| p-value (vs. placebo) † | 0.5894 | 0.0989 | 0.3801 | NA |  |
| **Day 9** |  |  |  |  |  |
| n (Missing) | 39 (0) | 41 (0) | 39 (0) | 41 (0) |  |
| Mean (SD) | 2.47 (0.47) | 2.45 (0.51) | 2.51 (0.37) | 2.60 (0.38) | 0.4039 |
| Median | 2.42 | 2.34 | 2.46 | 2.53 |  |
| Min, Max | 1.6, 3.6 | 1.5, 3.9 | 1.7, 3.4 | 2.0, 3.5 |  |
| 95% CI | 2.31 – 2.62 | 2.29 – 2.61 | 2.38 – 2.63 | 2.48 – 2.72 |  |
| p-value (vs. placebo) † | 0.1632 | 0.1282 | 0.2647 | NA |  |
| **Change from baseline on day 9** |  |  |  |  |  |
| n (Missing) | 39 (0) | 41 (0) | 39 (0) | 40 (1) |  |
| Mean (SD) | -0.48 (0.43) | -0.65 (0.51) | -0.57 (0.43) | -0.52 (0.41) | 0.3545 |
| Median | -0.42 | -0.63 | -0.55 | -0.51 |  |
| Min, Max | -1.4, 0.4 | -1.6, 0.6 | -1.6, 0.3 | -1.4, 0.4 |  |
| 95% CI | -0.62 – -0.34 | -0.81 – -0.49 | -0.71 – -0.43 | -0.65 – -0.38 |  |
| p-value (vs. baseline) § | <0.0001 | <0.0001 | <0.0001 | <0.0001 |  |
| p-value (vs. placebo) † | 0.6963 | 0.205 | 0.5394 | NA |  |
| **Day 29** |  |  |  |  |  |
| n (Missing) | 39 (0) | 41 (0) | 39 (0) | 41 (0) |  |
| Mean (SD) | 2.37 (0.42) | 2.47 (0.48) | 2.46 (0.33) | 2.47 (0.33) | 0.6341 |
| Median | 2.35 | 2.38 | 2.38 | 2.42 |  |
| Min, Max | 1.6, 3.6 | 1.7, 3.9 | 2.0, 3.4 | 1.9, 3.5 |  |
| 95% CI | 2.23 – 2.51 | 2.32 – 2.62 | 2.36 – 2.57 | 2.37 – 2.57 |  |
| p-value (vs. placebo) † | 0.2513 | 0.988 | 0.9233 | NA |  |
| **Change from baseline on day 29** |  |  |  |  |  |
| n (Missing) | 39 (0) | 41 (0) | 39 (0) | 40 (1) |  |
| Mean (SD) | -0.57 (0.48) | -0.63 (0.53) | -0.62 (0.41) | -0.66 (0.48) | 0.8842 |
| Median | -0.50 | -0.70 | -0.63 | -0.60 |  |
| Min, Max | -1.5, 0.4 | -1.9, 0.6 | -1.5, 0.1 | -1.7, 0.4 |  |
| 95% CI | -0.73 – -0.42 | -0.80 – -0.46 | -0.75 – -0.49 | -0.81 – -0.50 |  |
| p-value (vs. baseline) § | <0.0001 | <0.0001 | <0.0001 | <0.0001 |  |
| p-value (vs. placebo) † | 0.4291 | 0.7873 | 0.6825 | NA |  |

* Baseline is defined as the last value before the first dose.

‡ Difference of cytokine’s level among the four groups was analyzed by the ANOVA.

† Difference of cytokine’s level compared with placebo group was analyzed by the independent samples t-test.

§ Difference of cytokine’s level compared with baseline in each group was analyzed by paired t-test.

Abbreviations: N = Number of patients in analysis set; n = Number of patients; SoC = Standard of Care; NA= Not Applicable.

Table S17.

Level of TNF-α post administration (log10 pg/mL)

|  | **Meplazumab + SoC** | | | **Placebo + Soc  (N=41)** | **p-value** ‡ |
| --- | --- | --- | --- | --- | --- |
|  | **0.12 mg/kg (N=39)** | **0.2 mg/kg (N=41)** | **0.3 mg/kg (N=39)** |  |  |
| **Baseline*** |  |  |  |  |  |
| n (Missing) | 39 (0) | 41 (0) | 39 (0) | 40 (1) |  |
| Mean (SD) | 1.41 (0.25) | 1.45 (0.30) | 1.37 (0.24) | 1.37 (0.22) | 0.3754 |
| Median | 1.43 | 1.49 | 1.39 | 1.41 |  |
| Min, Max | 0.8, 1.8 | 0.7, 2.3 | 0.7, 1.7 | 0.9, 1.8 |  |
| 95% CI | 1.33 – 1.49 | 1.36 – 1.55 | 1.29 – 1.45 | 1.30 – 1.44 |  |
| p-value (vs. placebo) † | 0.428 | 0.1425 | 0.962 | NA |  |
| **Day 2** |  |  |  |  |  |
| n (Missing) | 39 (0) | 41 (0) | 39 (0) | 41 (0) |  |
| Mean (SD) | 1.39 (0.22) | 1.47 (0.29) | 1.37 (0.27) | 1.38 (0.20) | 0.2367 |
| Median | 1.35 | 1.50 | 1.43 | 1.38 |  |
| Min, Max | 0.8, 1.8 | 0.9, 2.2 | 0.6, 1.8 | 0.9, 1.8 |  |
| 95% CI | 1.32 – 1.46 | 1.38 – 1.56 | 1.28 – 1.46 | 1.32 – 1.44 |  |
| p-value (vs. placebo) † | 0.8317 | 0.0971 | 0.8884 | NA |  |
| **Change from baseline on day 2** |  |  |  |  |  |
| n (Missing) | 39 (0) | 41 (0) | 39 (0) | 40 (1) |  |
| Mean (SD) | -0.02 (0.20) | 0.02 (0.24) | 0.00 (0.20) | 0.01 (0.14) | 0.8378 |
| Median | -0.03 | 0.05 | 0.00 | -0.00 |  |
| Min, Max | -0.5, 0.3 | -0.4, 0.6 | -0.6, 0.5 | -0.3, 0.3 |  |
| 95% CI | -0.08 – 0.05 | -0.06 – 0.09 | -0.06 – 0.07 | -0.03 – 0.06 |  |
| p-value (vs. baseline) § | 0.5596 | 0.6178 | 0.9278 | 0.5452 |  |
| p-value (vs. placebo) † | 0.4057 | 0.9067 | 0.7777 | NA |  |
| **Day 8** |  |  |  |  |  |
| n (Missing) | 39 (0) | 41 (0) | 39 (0) | 41 (0) |  |
| Mean (SD) | 1.47 (0.36) | 1.45 (0.30) | 1.36 (0.27) | 1.38 (0.22) | 0.2352 |
| Median | 1.42 | 1.45 | 1.42 | 1.38 |  |
| Min, Max | 0.8, 2.9 | 0.5, 2.0 | 0.6, 1.8 | 0.9, 2.1 |  |
| 95% CI | 1.36 – 1.59 | 1.36 – 1.55 | 1.27 – 1.44 | 1.31 – 1.45 |  |
| p-value (vs. placebo) † | 0.1733 | 0.2313 | 0.6583 | NA |  |
| **Change from baseline on day 8** |  |  |  |  |  |
| n (Missing) | 39 (0) | 41 (0) | 39 (0) | 40 (1) |  |
| Mean (SD) | 0.07 (0.40) | -0.00 (0.26) | -0.01 (0.20) | 0.02 (0.23) | 0.6293 |
| Median | 0.04 | 0.01 | -0.00 | 0.03 |  |
| Min, Max | -0.6, 1.9 | -0.7, 0.5 | -0.5, 0.4 | -0.4, 0.9 |  |
| 95% CI | -0.06 – 0.19 | -0.08 – 0.08 | -0.08 – 0.05 | -0.06 – 0.09 |  |
| p-value (vs. baseline) § | 0.3091 | 0.9673 | 0.7053 | 0.6206 |  |
| p-value (vs. placebo) † | 0.5211 | 0.7179 | 0.5335 | NA |  |
| **Day 9** |  |  |  |  |  |
| n (Missing) | 39 (0) | 41 (0) | 39 (0) | 41 (0) |  |
| Mean (SD) | 1.46 (0.28) | 1.46 (0.29) | 1.38 (0.25) | 1.36 (0.21) | 0.1889 |
| Median | 1.43 | 1.50 | 1.39 | 1.36 |  |
| Min, Max | 0.9, 2.3 | 0.5, 2.1 | 0.6, 1.9 | 0.8, 1.9 |  |
| 95% CI | 1.37 – 1.55 | 1.36 – 1.55 | 1.30 – 1.46 | 1.29 – 1.42 |  |
| p-value (vs. placebo) † | 0.0682 | 0.0787 | 0.6349 | NA |  |
| **Change from baseline on day 9** |  |  |  |  |  |
| n (Missing) | 39 (0) | 41 (0) | 39 (0) | 40 (1) |  |
| Mean (SD) | 0.05 (0.33) | 0.00 (0.22) | 0.01 (0.27) | -0.01 (0.21) | 0.7913 |
| Median | 0.06 | 0.02 | -0.00 | -0.02 |  |
| Min, Max | -0.7, 1.3 | -0.7, 0.5 | -0.5, 0.9 | -0.4, 0.6 |  |
| 95% CI | -0.06 – 0.16 | -0.07 – 0.07 | -0.08 – 0.10 | -0.07 – 0.06 |  |
| p-value (vs. baseline) § | 0.3493 | 0.9210 | 0.7811 | 0.8652 |  |
| p-value (vs. placebo) † | 0.3735 | 0.8488 | 0.7448 | NA |  |
| **Day 29** |  |  |  |  |  |
| n (Missing) | 39 (0) | 41 (0) | 39 (0) | 41 (0) |  |
| Mean (SD) | 1.40 (0.29) | 1.41 (0.27) | 1.37 (0.24) | 1.29 (0.31) | 0.2322 |
| Median | 1.39 | 1.46 | 1.31 | 1.31 |  |
| Min, Max | 0.9, 2.5 | 0.7, 2.1 | 0.9, 2.3 | 0.2, 1.9 |  |
| 95% CI | 1.30 – 1.49 | 1.33 – 1.50 | 1.29 – 1.45 | 1.19 – 1.39 |  |
| p-value (vs. placebo) † | 0.1236 | 0.0724 | 0.2309 | NA |  |
| **Change from baseline on day 29** |  |  |  |  |  |
| n (Missing) | 39 (0) | 41 (0) | 39 (0) | 40 (1) |  |
| Mean (SD) | -0.01 (0.33) | -0.04 (0.27) | 0.00 (0.25) | -0.08 (0.30) | 0.6416 |
| Median | -0.05 | -0.02 | -0.03 | -0.06 |  |
| Min, Max | -0.7, 0.9 | -0.7, 0.5 | -0.5, 0.7 | -1.0, 0.6 |  |
| 95% CI | -0.12 – 0.10 | -0.13 – 0.04 | -0.08 – 0.08 | -0.17 – 0.02 |  |
| p-value (vs. baseline) § | 0.8563 | 0.3318 | 0.9930 | 0.1145 |  |
| p-value (vs. placebo) † | 0.352 | 0.5894 | 0.2246 | NA |  |

* Baseline is defined as the last value before the first dose.

‡ Difference of cytokine’s level among the four groups was analyzed by the ANOVA.

† Difference of cytokine’s level compared with placebo group was analyzed by the independent samples t-test.

§ Difference of cytokine’s level compared with baseline in each group was analyzed by paired t-test.

Abbreviations: N = Number of patients in analysis set; n = Number of patients; SoC = Standard of Care; NA= Not Applicable.

Table S18.

Level of IFN-γ post administration (log10 pg/mL)

|  | **Meplazumab + SoC** | | | **Placebo + Soc  (N=41)** | **p-value** ‡ |
| --- | --- | --- | --- | --- | --- |
|  | **0.12 mg/kg (N=39)** | **0.2 mg/kg (N=41)** | **0.3 mg/kg (N=39)** |  |  |
| **Baseline*** |  |  |  |  |  |
| n (Missing) | 38 (1) | 38 (3) | 37 (2) | 38 (3) |  |
| Mean (SD) | 1.02 (0.65) | 1.31 (0.71) | 1.02 (0.61) | 1.10 (0.72) | 0.1990 |
| Median | 1.01 | 1.43 | 1.13 | 1.07 |  |
| Min, Max | 0.2, 2.3 | 0.2, 2.9 | 0.2, 2.3 | 0.2, 2.7 |  |
| 95% CI | 0.81 – 1.24 | 1.08 – 1.55 | 0.82 – 1.22 | 0.86 – 1.33 |  |
| p-value (vs. placebo) † | 0.6329 | 0.1968 | 0.613 | NA |  |
| **Day 2** |  |  |  |  |  |
| n (Missing) | 39 (0) | 40 (1) | 39 (0) | 41 (0) |  |
| Mean (SD) | 0.98 (0.63) | 1.18 (0.75) | 0.97 (0.53) | 1.02 (0.65) | 0.4356 |
| Median | 1.01 | 0.98 | 1.04 | 1.09 |  |
| Min, Max | 0.2, 2.3 | 0.2, 2.7 | 0.2, 2.2 | 0.2, 2.6 |  |
| 95% CI | 0.77 – 1.18 | 0.94 – 1.42 | 0.80 – 1.15 | 0.82 – 1.23 |  |
| p-value (vs. placebo) † | 0.7408 | 0.3157 | 0.7017 | NA |  |
| **Change from baseline on day 2** |  |  |  |  |  |
| n (Missing) | 38 (1) | 38 (3) | 37 (2) | 38 (3) |  |
| Mean (SD) | -0.04 (0.46) | -0.11 (0.36) | -0.03 (0.30) | -0.04 (0.41) | 0.7906 |
| Median | -0.00 | -0.00 | -0.00 | -0.00 |  |
| Min, Max | -1.0, 1.0 | -0.8, 0.8 | -0.8, 0.7 | -1.4, 0.9 |  |
| 95% CI | -0.19 – 0.11 | -0.23 – 0.01 | -0.13 – 0.07 | -0.18 – 0.09 |  |
| p-value (vs. baseline) § | 0.6043 | 0.0669 | 0.5525 | 0.5118 |  |
| p-value (vs. placebo) † | 0.966 | 0.4488 | 0.8621 | NA |  |
| **Day 8** |  |  |  |  |  |
| n (Missing) | 39 (0) | 41 (0) | 39 (0) | 41 (0) |  |
| Mean (SD) | 1.10 (0.63) | 1.04 (0.73) | 0.93 (0.63) | 0.98 (0.63) | 0.6663 |
| Median | 0.98 | 0.98 | 0.94 | 1.07 |  |
| Min, Max | 0.2, 2.2 | 0.2, 2.4 | 0.2, 2.1 | 0.2, 2.6 |  |
| 95% CI | 0.90 – 1.31 | 0.81 – 1.27 | 0.72 – 1.13 | 0.78 – 1.17 |  |
| p-value (vs. placebo) † | 0.3765 | 0.6654 | 0.7294 | NA |  |
| **Change from baseline on day 8** |  |  |  |  |  |
| n (Missing) | 38 (1) | 38 (3) | 37 (2) | 38 (3) |  |
| Mean (SD) | 0.10 (0.57) | -0.23 (0.58) | -0.09 (0.34) | -0.09 (0.52) | 0.0459 |
| Median | -0.00 | -0.06 | -0.00 | -0.00 |  |
| Min, Max | -1.3, 2.0 | -1.7, 0.9 | -1.1, 0.5 | -1.4, 0.9 |  |
| 95% CI | -0.09 – 0.29 | -0.42 – -0.04 | -0.20 – 0.02 | -0.26 – 0.08 |  |
| p-value (vs. baseline) § | 0.2817 | 0.0171 | 0.1188 | 0.2910 |  |
| p-value (vs. placebo) † | 0.1306 | 0.2577 | 0.998 | NA |  |
| **Day 9** |  |  |  |  |  |
| n (Missing) | 39 (0) | 41 (0) | 39 (0) | 41 (0) |  |
| Mean (SD) | 0.95 (0.62) | 1.06 (0.69) | 0.90 (0.63) | 0.96 (0.60) | 0.7323 |
| Median | 0.96 | 1.05 | 0.91 | 1.01 |  |
| Min, Max | 0.2, 2.3 | 0.2, 2.6 | 0.2, 2.3 | 0.2, 2.6 |  |
| 95% CI | 0.75 – 1.15 | 0.84 – 1.27 | 0.69 – 1.10 | 0.77 – 1.15 |  |
| p-value (vs. placebo) † | 0.9313 | 0.5202 | 0.6322 | NA |  |
| **Change from baseline on day 9** |  |  |  |  |  |
| n (Missing) | 38 (1) | 38 (3) | 37 (2) | 38 (3) |  |
| Mean (SD) | -0.07 (0.51) | -0.22 (0.52) | -0.13 (0.41) | -0.12 (0.54) | 0.6441 |
| Median | -0.00 | -0.08 | -0.00 | -0.03 |  |
| Min, Max | -1.5, 1.0 | -1.6, 0.7 | -1.5, 0.9 | -1.1, 1.1 |  |
| 95% CI | -0.24 – 0.10 | -0.39 – -0.05 | -0.26 – 0.01 | -0.30 – 0.05 |  |
| p-value (vs. baseline) § | 0.3923 | 0.0143 | 0.0715 | 0.1648 |  |
| p-value (vs. placebo) † | 0.6686 | 0.4435 | 0.982 | NA |  |
| **Day 29** |  |  |  |  |  |
| n (Missing) | 39 (0) | 41 (0) | 39 (0) | 41 (0) |  |
| Mean (SD) | 0.91 (0.64) | 1.05 (0.72) | 0.97 (0.65) | 0.99 (0.69) | 0.8411 |
| Median | 0.93 | 1.04 | 0.95 | 0.93 |  |
| Min, Max | 0.2, 2.4 | 0.2, 2.6 | 0.2, 2.3 | 0.2, 2.6 |  |
| 95% CI | 0.70 – 1.12 | 0.82 – 1.27 | 0.75 – 1.18 | 0.77 – 1.20 |  |
| p-value (vs. placebo) † | 0.6025 | 0.7058 | 0.8922 | NA |  |
| **Change from baseline on day 29** |  |  |  |  |  |
| n (Missing) | 38 (1) | 38 (3) | 37 (2) | 38 (3) |  |
| Mean (SD) | -0.10 (0.63) | -0.22 (0.54) | -0.06 (0.46) | -0.10 (0.63) | 0.6262 |
| Median | -0.10 | -0.11 | -0.00 | -0.00 |  |
| Min, Max | -1.2, 1.4 | -2.2, 0.8 | -1.1, 0.9 | -1.6, 1.3 |  |
| 95% CI | -0.30 – 0.11 | -0.40 – -0.04 | -0.21 – 0.10 | -0.31 – 0.11 |  |
| p-value (vs. baseline) § | 0.3554 | 0.0168 | 0.4728 | 0.3266 |  |
| p-value (vs. placebo) † | 0.9716 | 0.3802 | 0.7174 | NA |  |

* Baseline is defined as the last value before the first dose.

‡ Difference of cytokine’s level among the four groups was analyzed by the ANOVA.

† Difference of cytokine’s level compared with placebo group was analyzed by the independent samples t-test.

§ Difference of cytokine’s level compared with baseline in each group was analyzed by paired t-test.

Abbreviations: N = Number of patients in analysis set; n = Number of patients; SoC = Standard of Care; NA= Not Applicable.

Table S19.

Electrocardiograph examination in the safety analysis set

| **Change from baseline** | **Meplazumab + SoC** | | | **Placebo + SoC**  **(N=41)**  **n (%)** | **p-value**‡ |
| --- | --- | --- | --- | --- | --- |
|  | **0.12 mg/kg (N=40)**  **n (%)** | **0.2 mg/kg (N=41)**  **n (%)** | **0.3 mg/kg (N=40)**  **n (%)** |  |  |
| Unchanged | 15 (37.5) | 15 (36.6) | 9 (22.5) | 10 (24.4) | 0.5733 |
| Improved | 1 (2.5) | 3 (7.3) | 2 (5.0) | 2 (4.9) |  |
| Worsened | 0 | 4 (9.8) | 2 (5.0) | 1 (2.4) |  |
| p-value  (vs. placebo) † | 0.3597 | 0.6927 | 0.8245 | NA |  |
| ‡ Difference among the four groups was analyzed by the chi-square test.  † Difference compared with placebo group was analyzed by the chi-square test.  Abbreviations: N = Number of patients in analysis set; n = Number of patients; SoC = Standard of Care; NA= Not Applicable. | | | | | |

<insert page break then Fig. S1 here>

Figure. S1.

Change from baseline of IL-2 at each time point (log10 pg/mL, the baseline positive population)


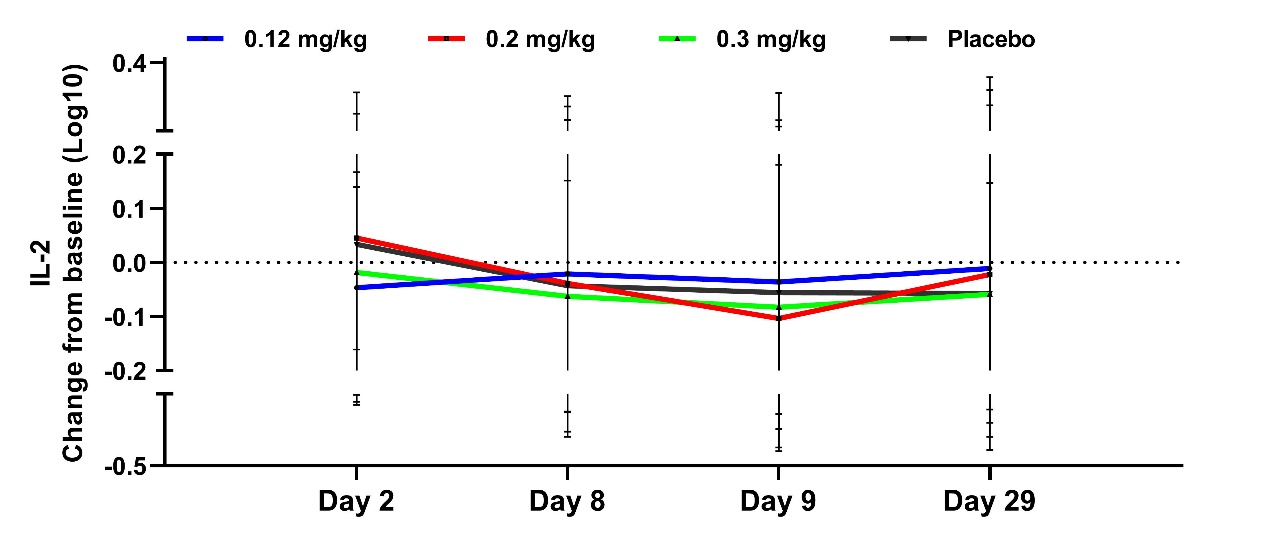


Figure. S2.

The difference of change from baseline between the meplazumab group and the placebo group of IL-2 (log10 pg/mL, the baseline positive population)

**
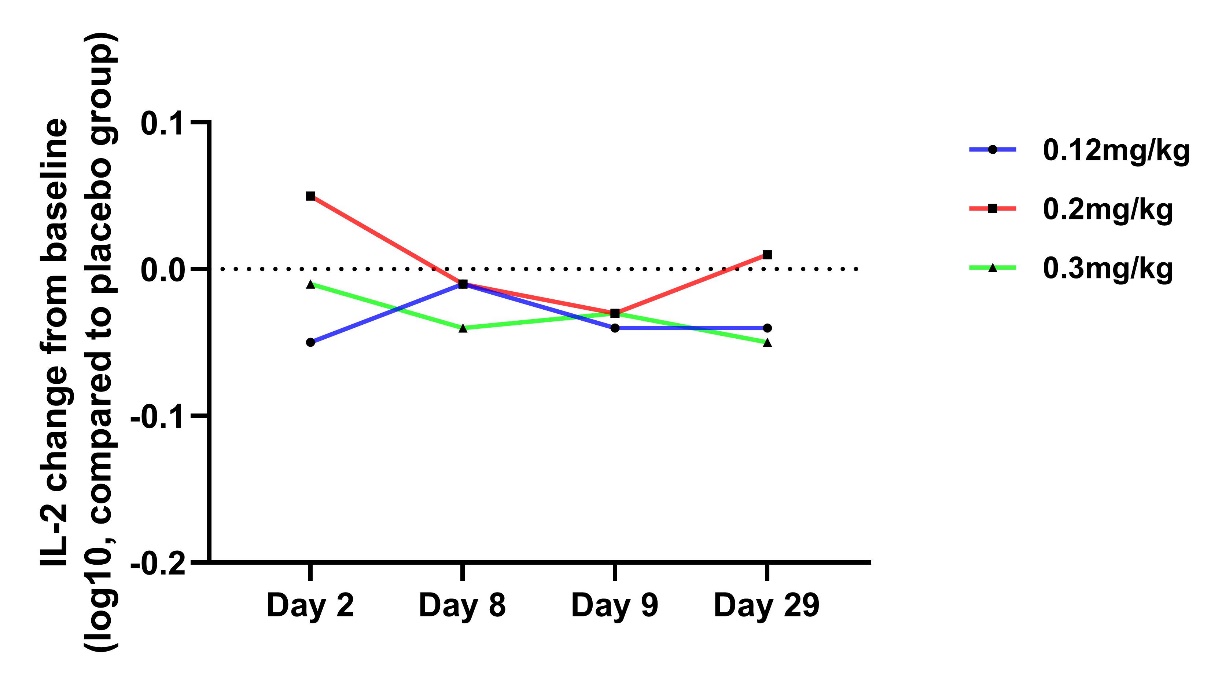
**

Figure. S3.

Change from baseline of IL-4 at each time point (log10 pg/mL, the baseline positive population)


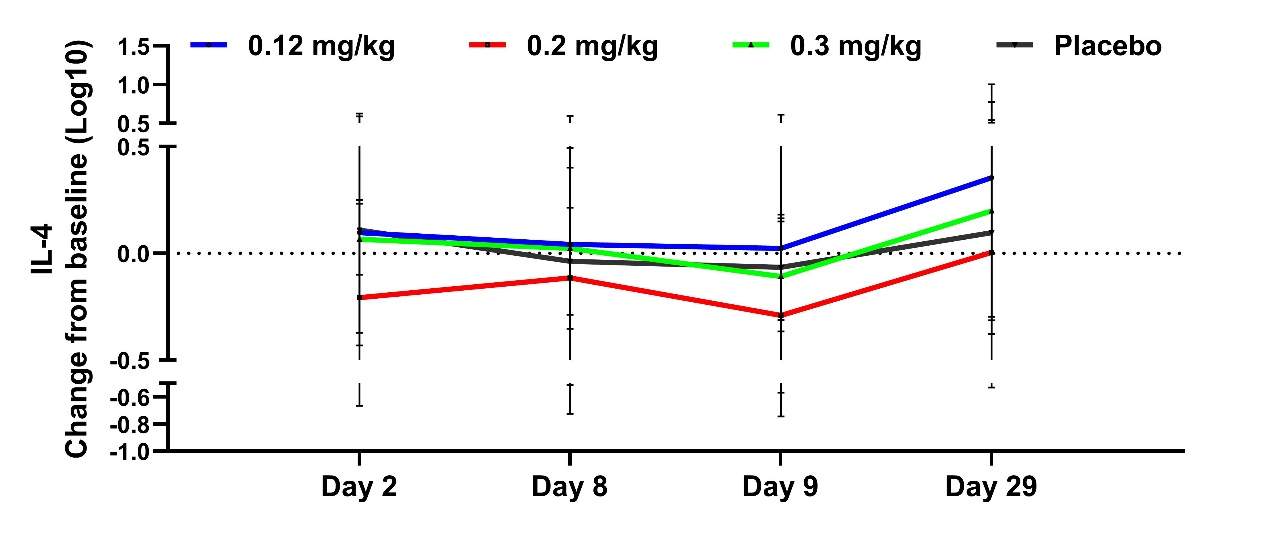


Figure. S4.

The difference of change from baseline between the meplazumab group and the placebo group of IL-4 (log10 pg/mL, the baseline positive population)

**
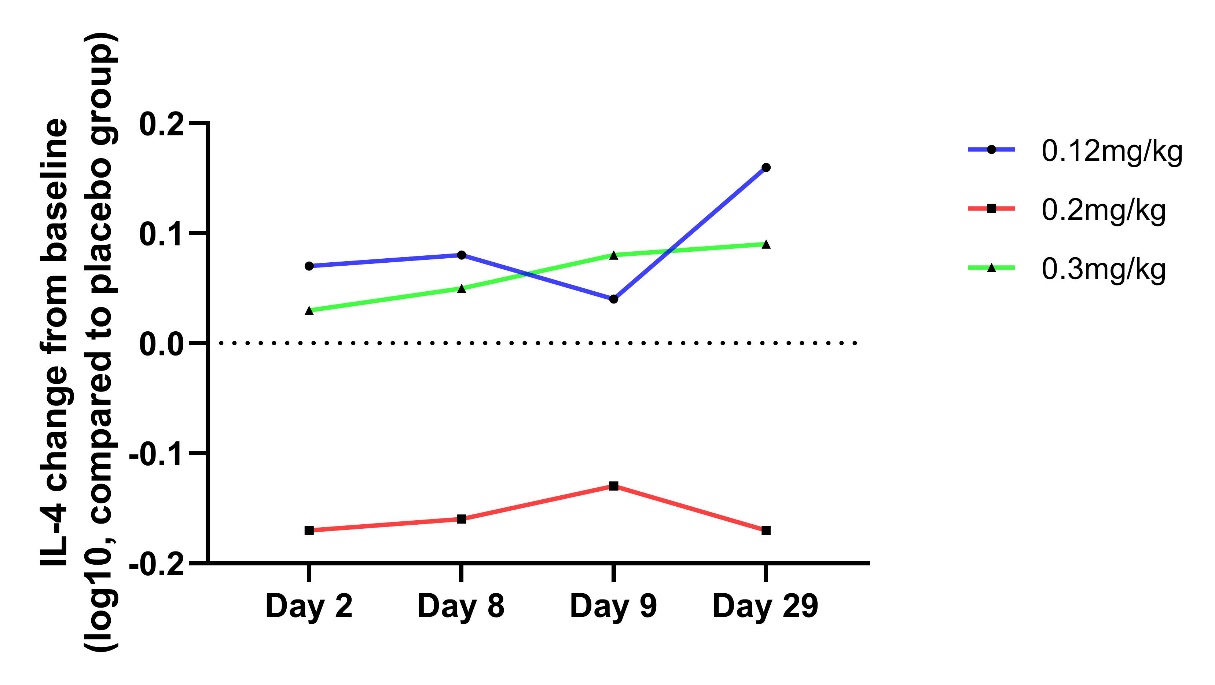
**

Figure. S5.

Change from baseline of IL-6 at each time point (log10 pg/mL, the baseline positive population)


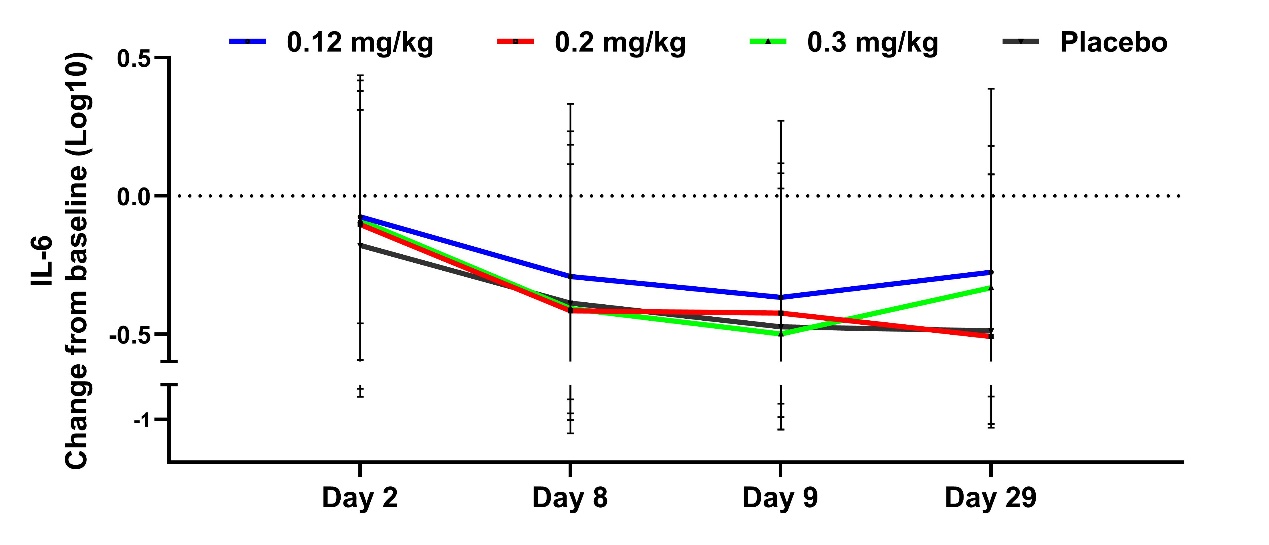


Figure. S6.

The difference of change from baseline between the meplazumab group and the placebo group of IL-6 (log10 pg/mL, the baseline positive population)

**
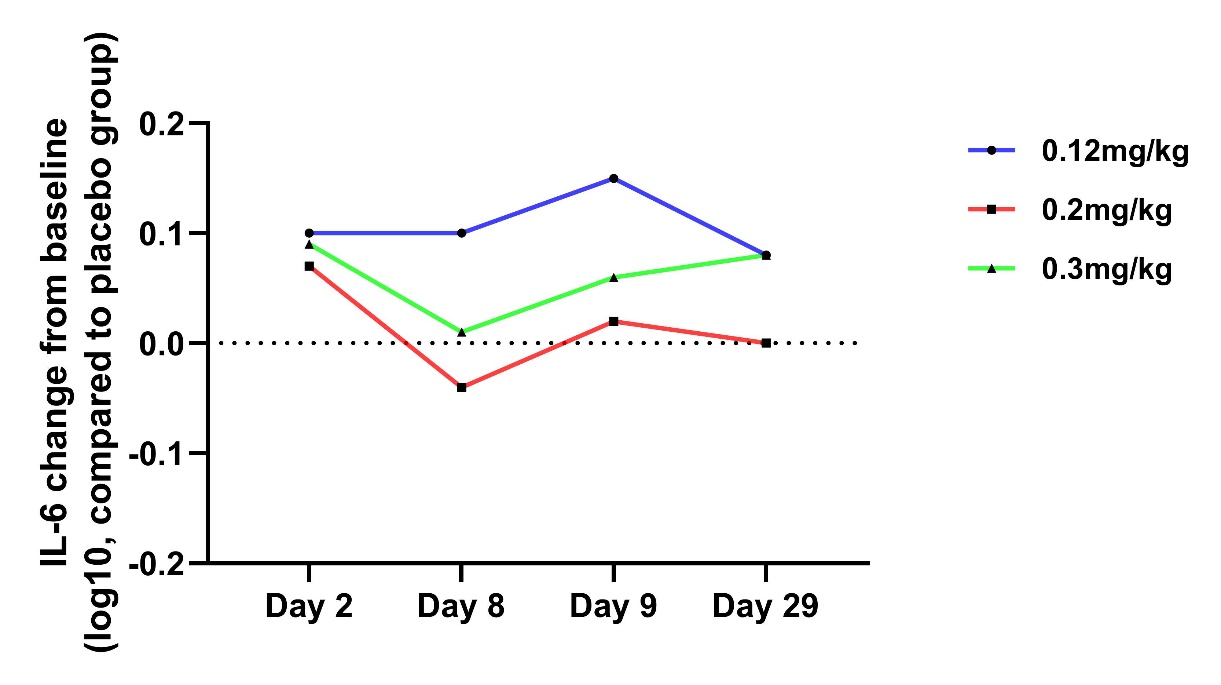
**

Figure. S7.

Change from baseline of IL-7 at each time point (log10 pg/mL, the baseline positive population)


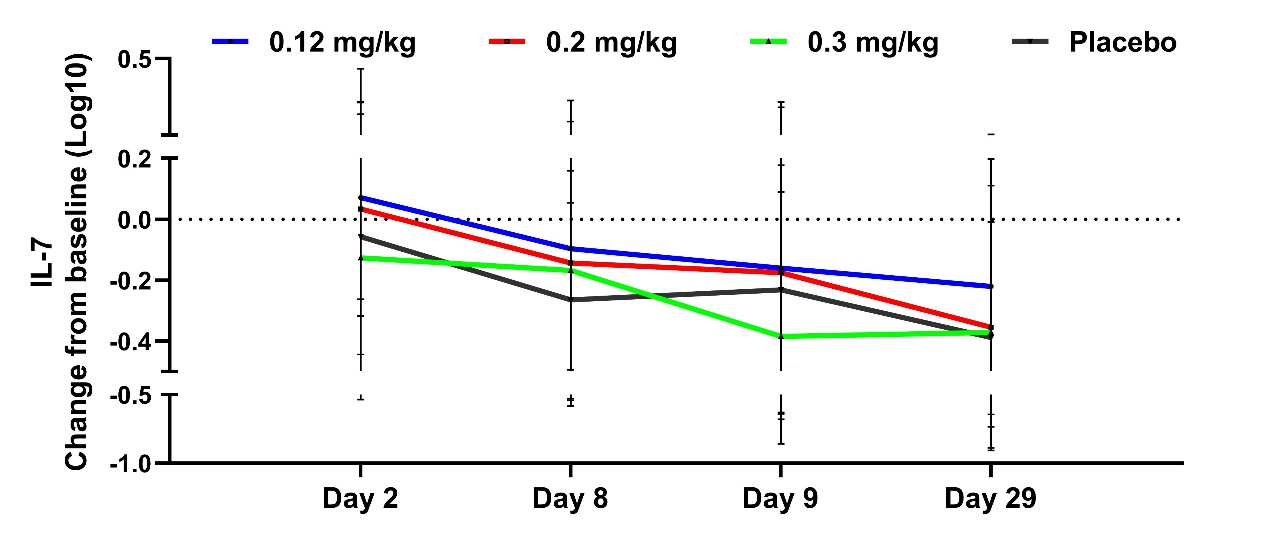


Figure. S8.

The difference of change from baseline between the meplazumab group and the placebo group of IL-7 (log10 pg/mL, the baseline positive population)


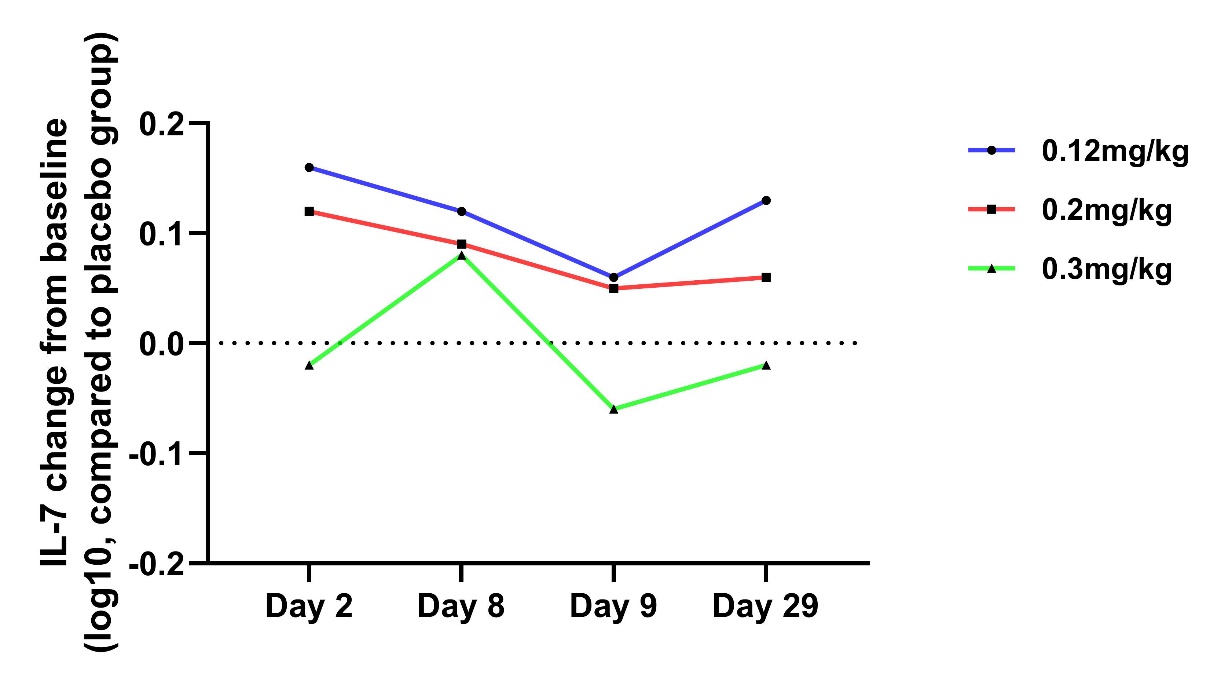


Figure. S9.

Change from baseline of IL-8 at each time point (log10 pg/mL, the baseline positive population)


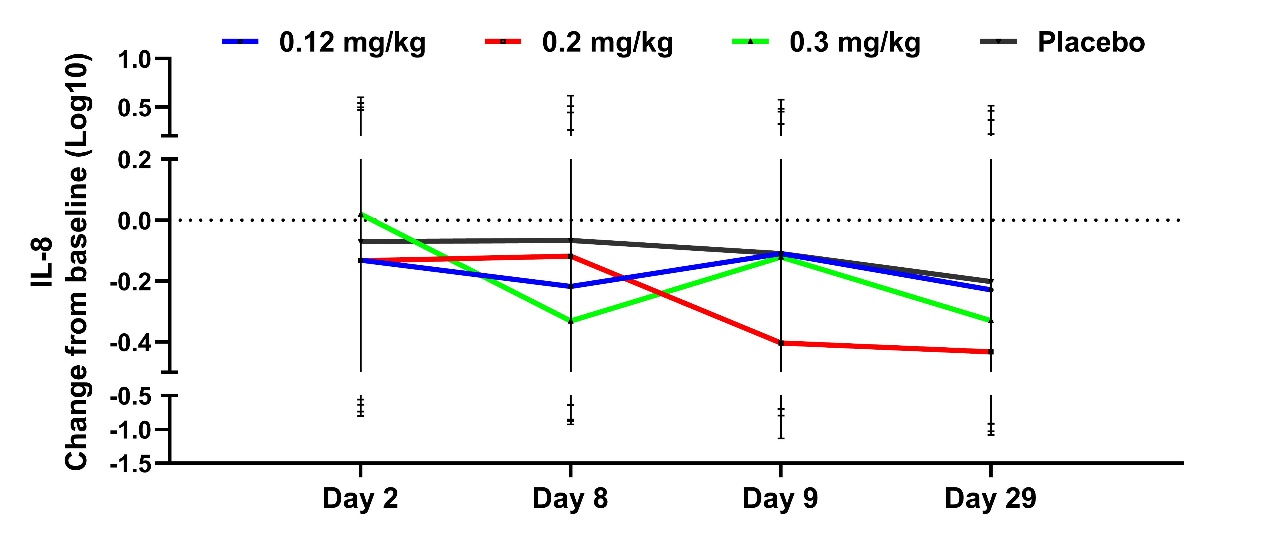


Figure. S10.

The difference of change from baseline between the meplazumab group and the placebo group of IL-8 (log10 pg/mL, the baseline positive population)


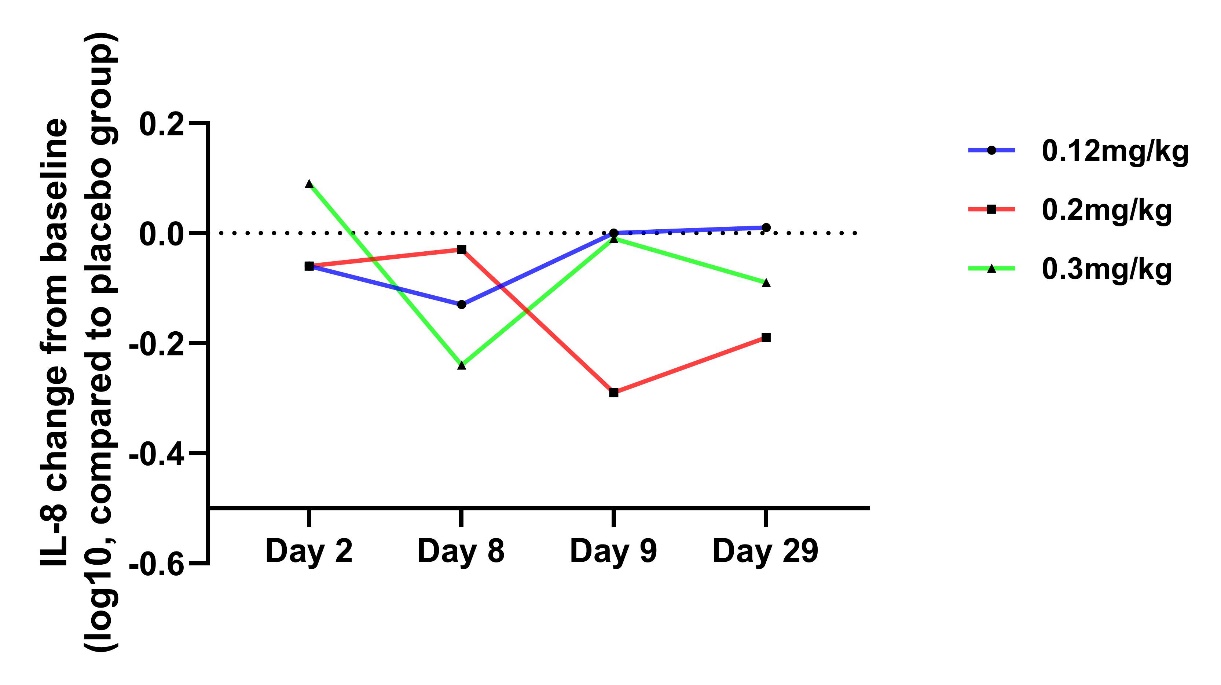


Figure. S11.

Change from baseline of IL-10 at each time point (log10 pg/mL, the baseline positive population)


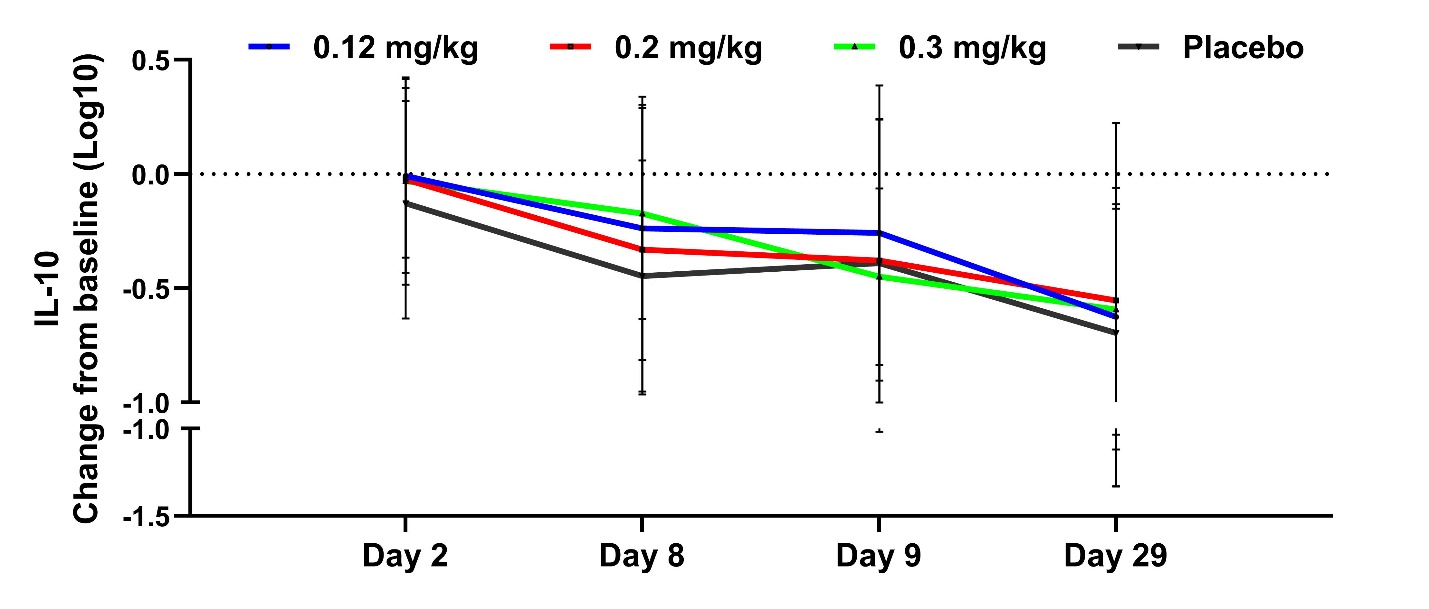


Figure. S12.

The difference of change from baseline between the meplazumab group and the placebo group of IL-10 (log10 pg/mL, the baseline positive population)


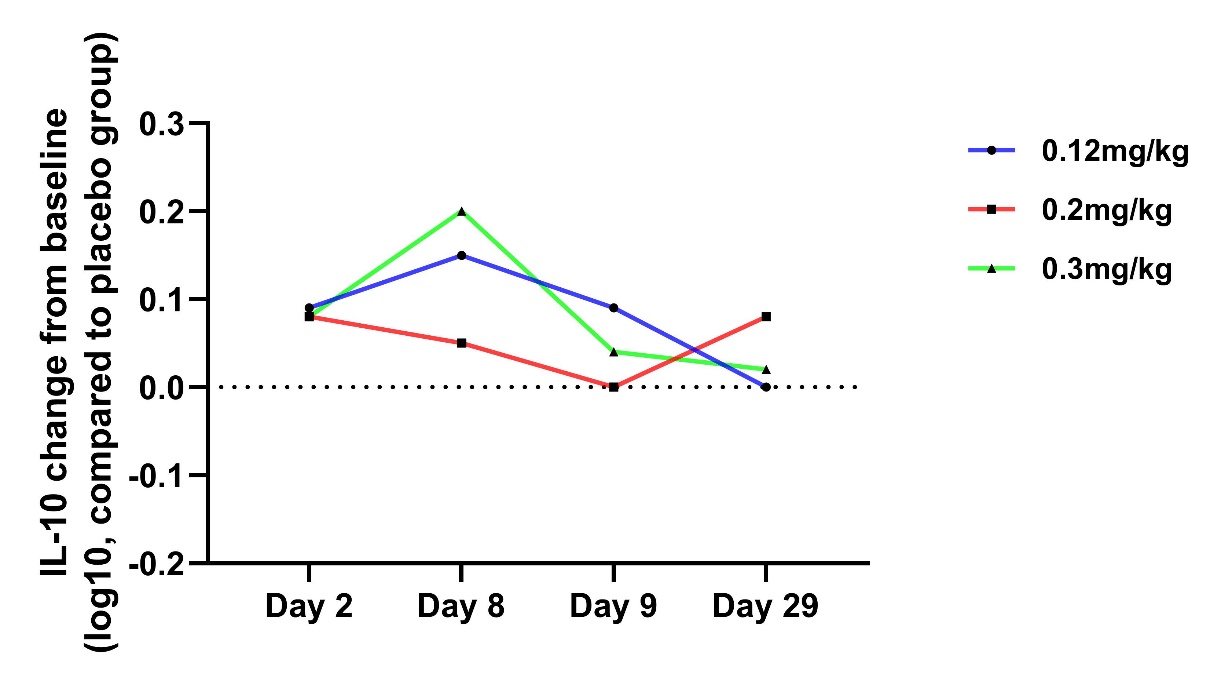


Figure. S13.

Change from baseline of IL-12p70 at each time point (log10 pg/mL, the baseline positive population)


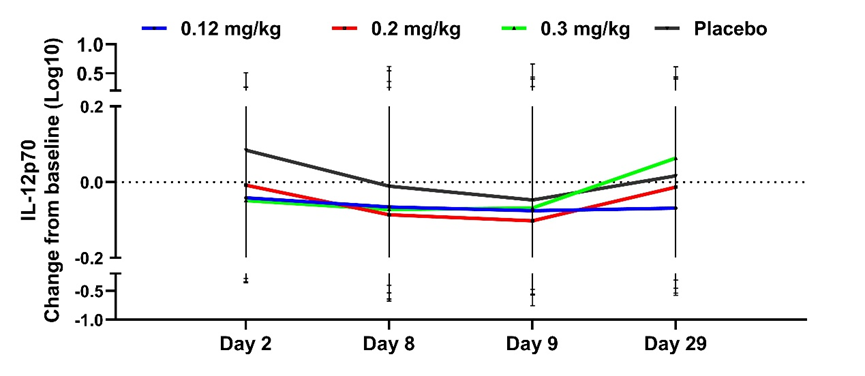


Figure. S14.

The difference of change from baseline between the meplazumab group and the placebo group of IL-12p70 (log10 pg/mL, the baseline positive population)


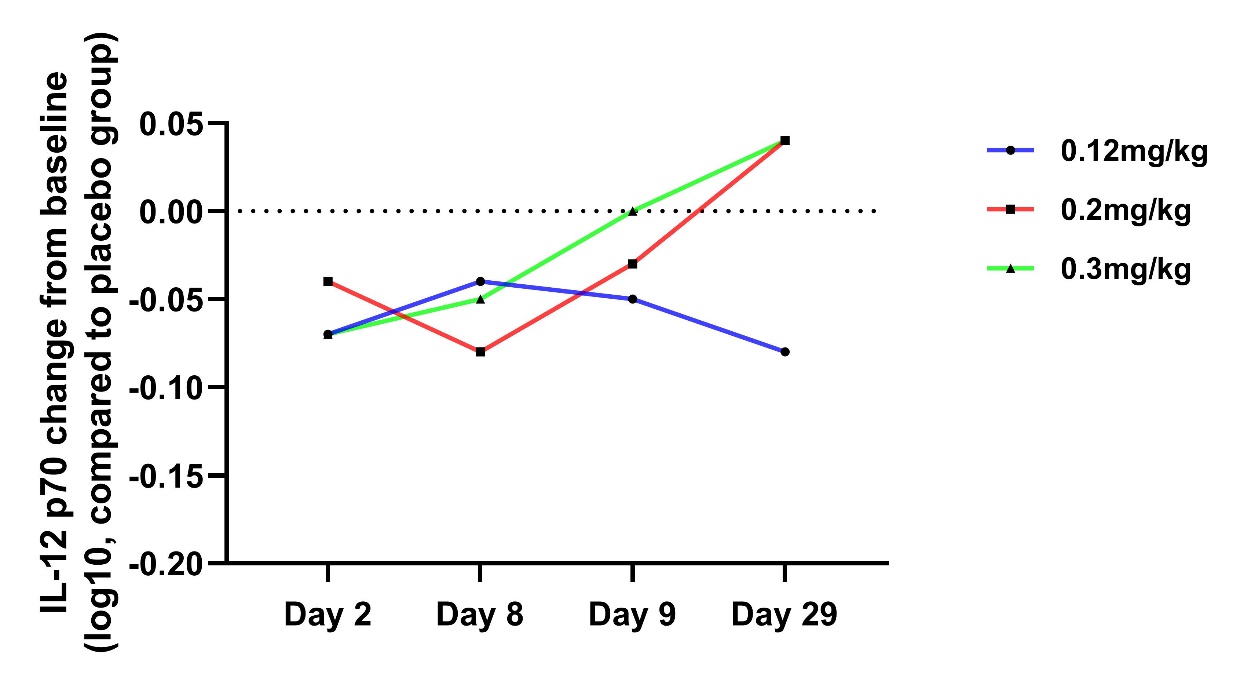


Figure. S15.

Change from baseline of IL-15 at each time point (log10 pg/mL, the baseline positive population)


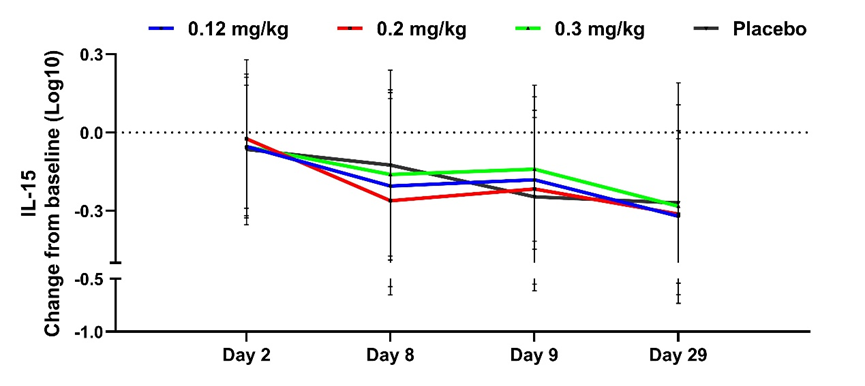


Figure. S16.

The difference of change from baseline between the meplazumab group and the placebo group of IL-15 (log10 pg/mL, the baseline positive population)


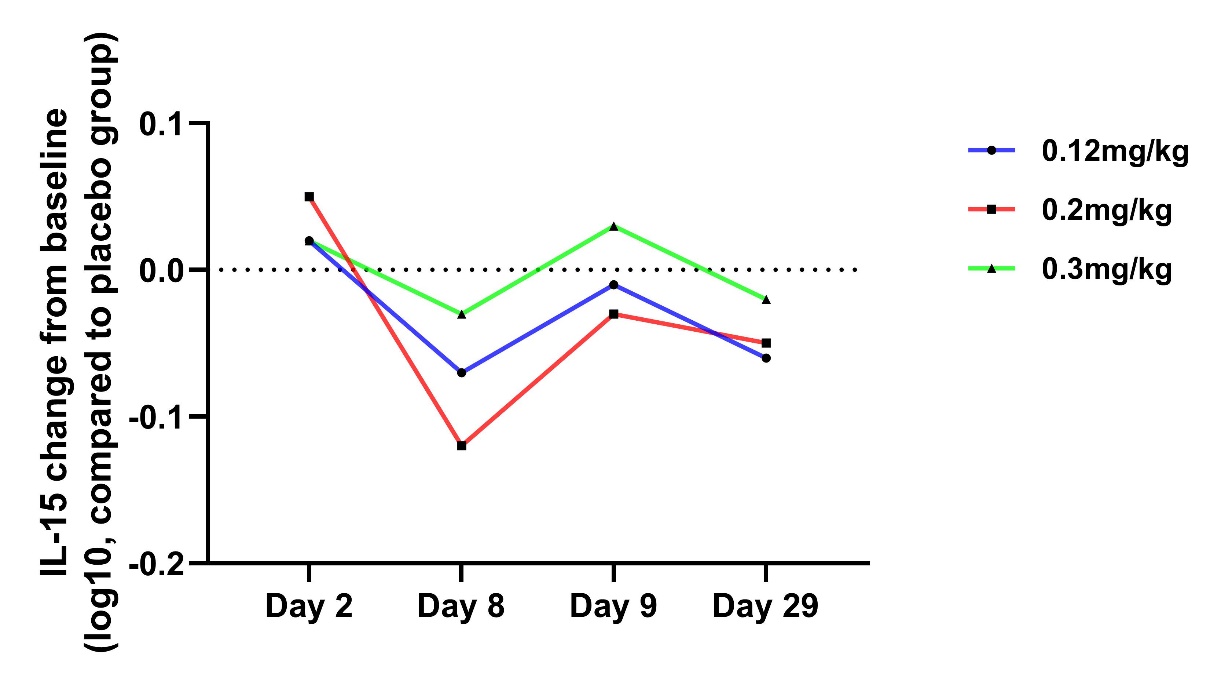


Figure. S17.

Change from baseline of IL-17A at each time point (log10 pg/mL, the baseline positive population)


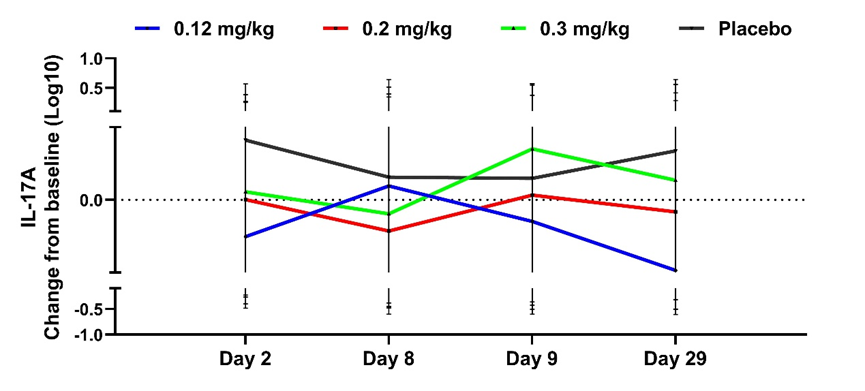


Figure. S18.

The difference of change from baseline between the meplazumab group and the placebo group of IL-17A (log10 pg/mL, the baseline positive population)


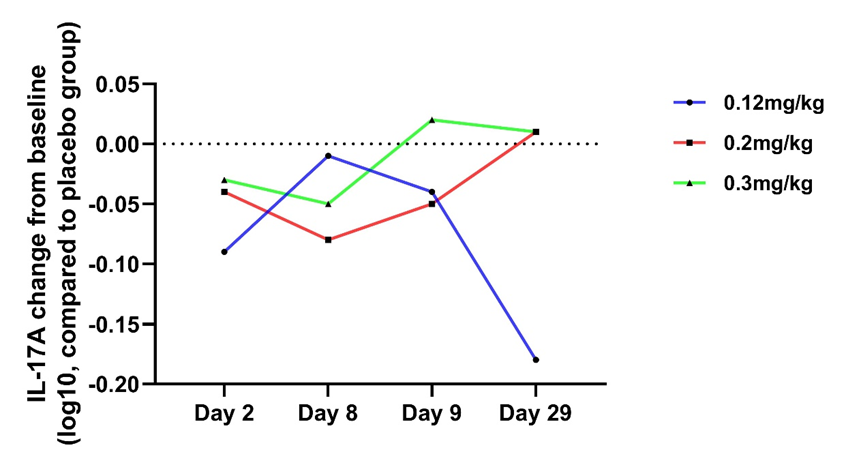


Figure. S19.

Change from baseline of IL-1ra at each time point (log10 pg/mL, the baseline positive population)


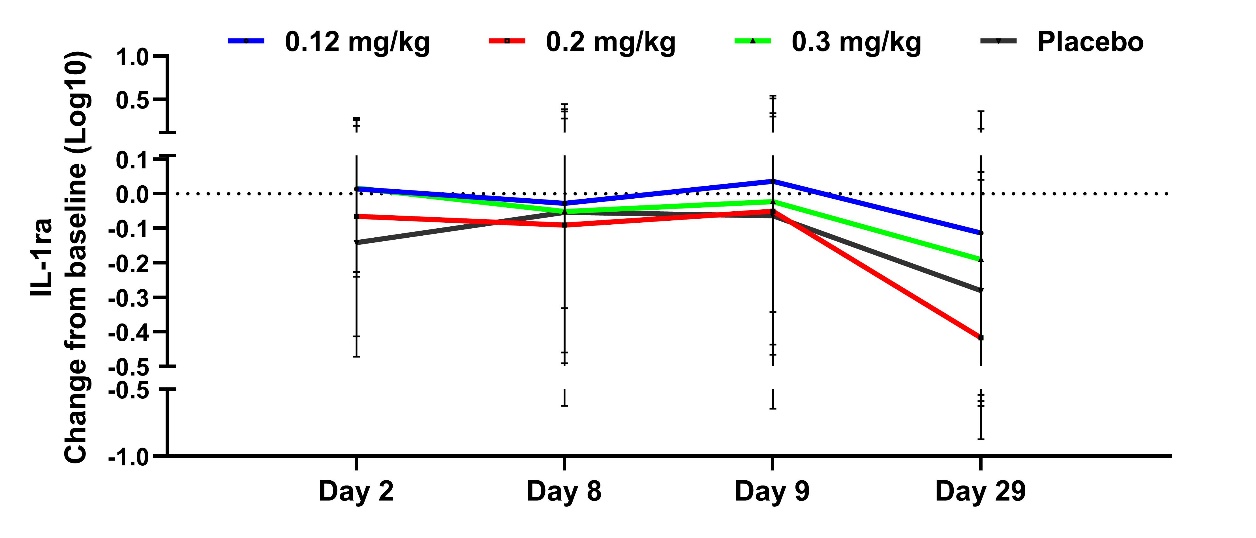


Figure. S20.

The difference of change from baseline between the meplazumab group and the placebo group of IL-1ra (log10 pg/mL, the baseline positive population)


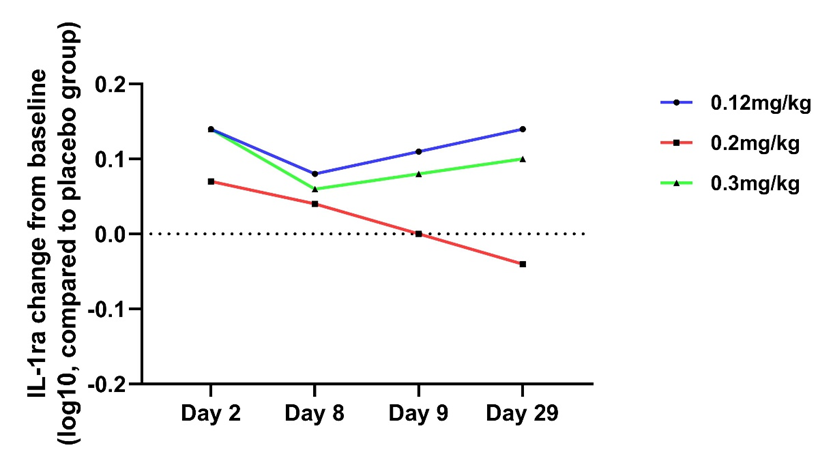


Figure. S21.

Change from baseline of IL-2Rα at each time point (log10 pg/mL, the baseline positive population)


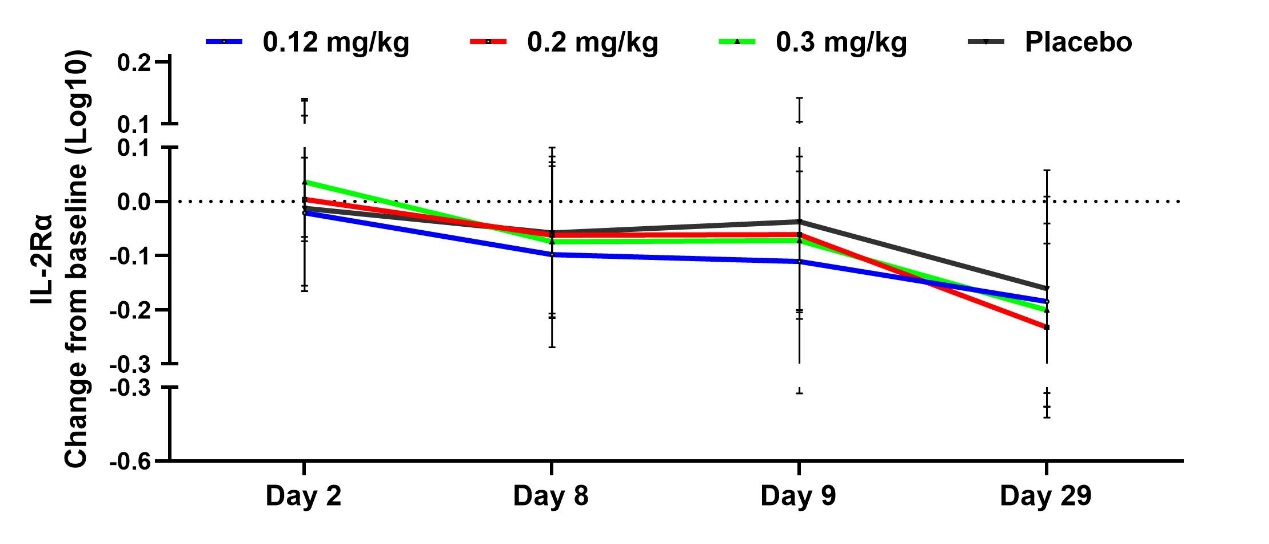


Figure. S22.

The difference of change from baseline between the meplazumab group and the placebo group of IL-2Rα (log10 pg/mL, the baseline positive population)


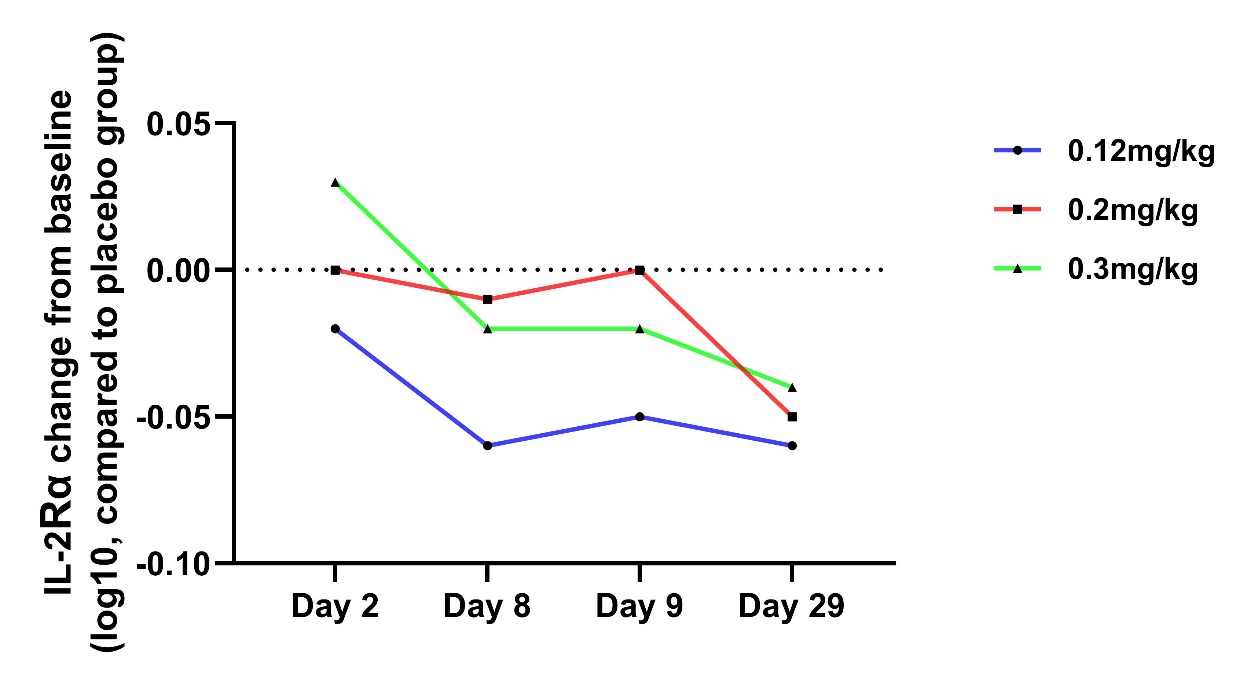


Figure. S23.

Change from baseline of MCP-1 at each time point (log10 pg/mL, the baseline positive population)


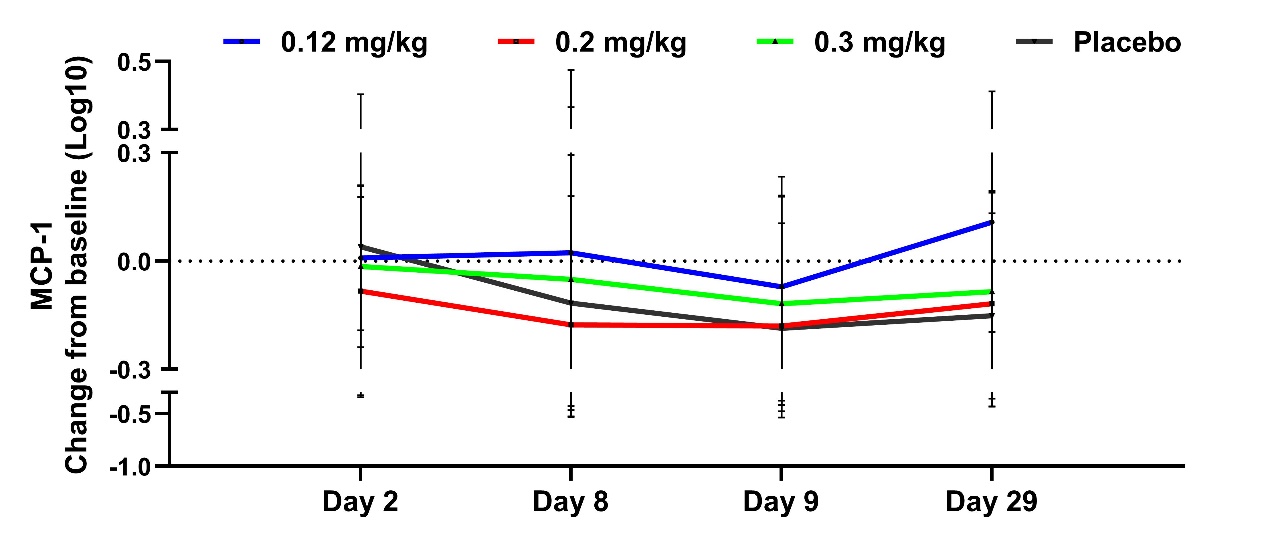


Figure. S24.

The difference of change from baseline between the meplazumab group and the placebo group of MCP-1 (log10 pg/mL, the baseline positive population)


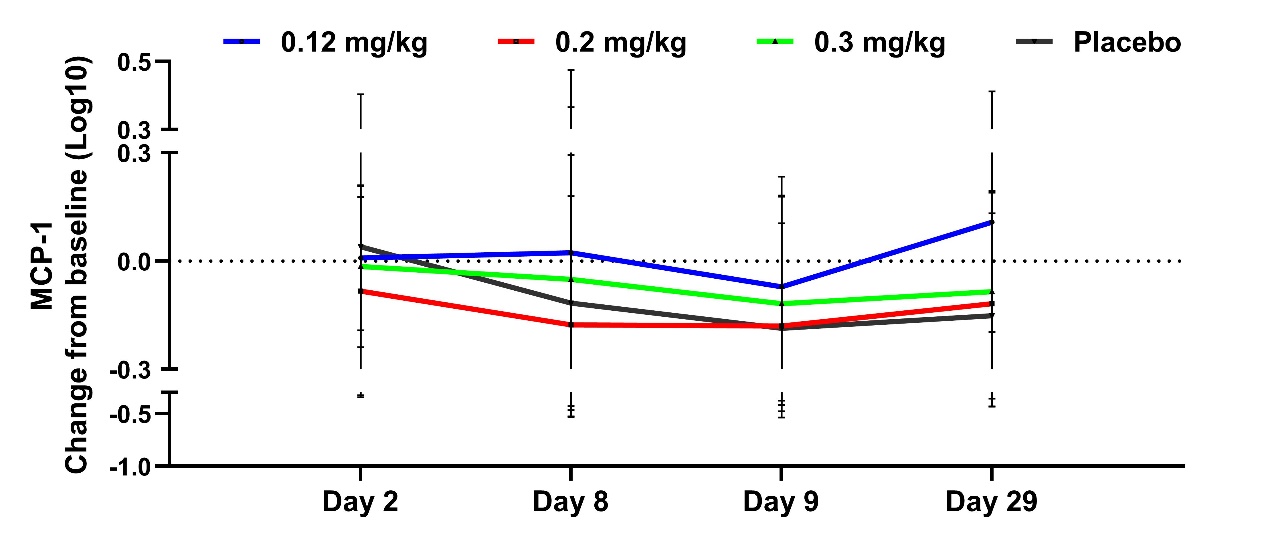


Figure. S25.

Change from baseline of MIP-1β at each time point (log10 pg/mL, the baseline positive population)


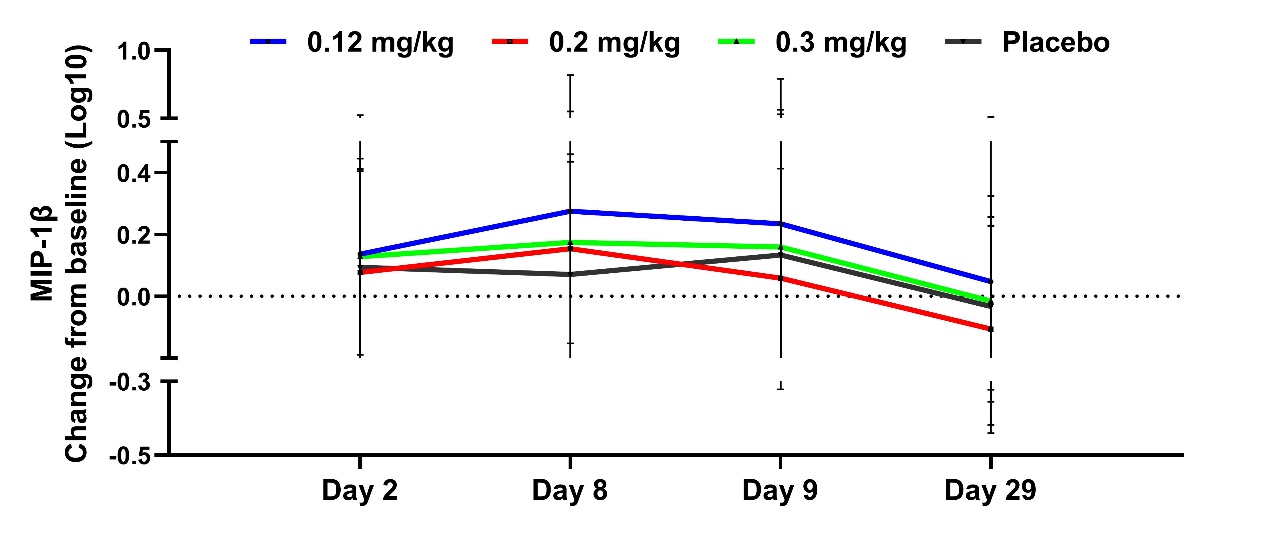


Figure. S26.

The difference of change from baseline between the meplazumab group and the placebo group of MIP-1β (log10 pg/mL, the baseline positive population)


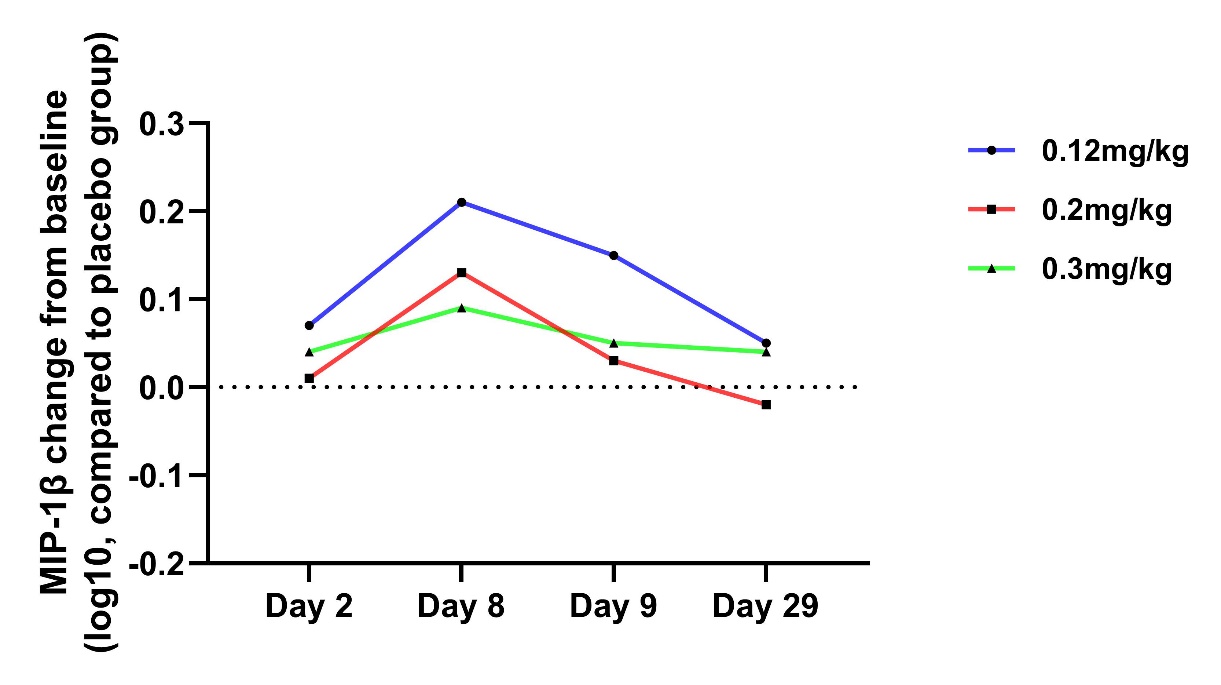


Figure. S27.

Change from baseline of IP-10 at each time point (log10 pg/mL, the baseline positive population)


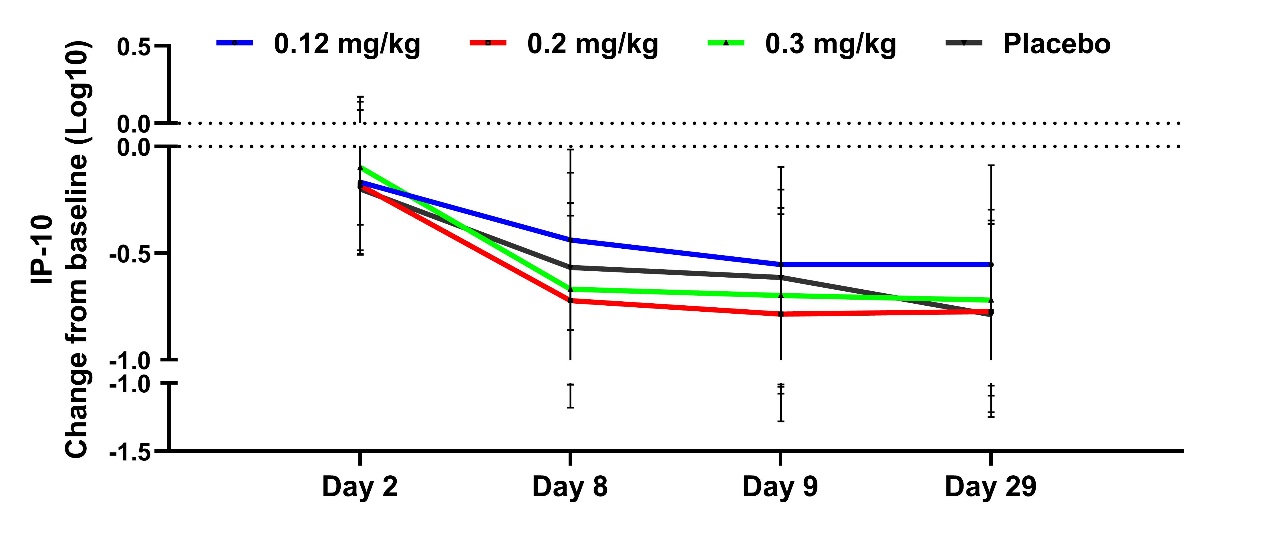


Figure. S28.

The difference of change from baseline between the meplazumab group and the placebo group of IP-10 (log10 pg/mL, the baseline positive population)


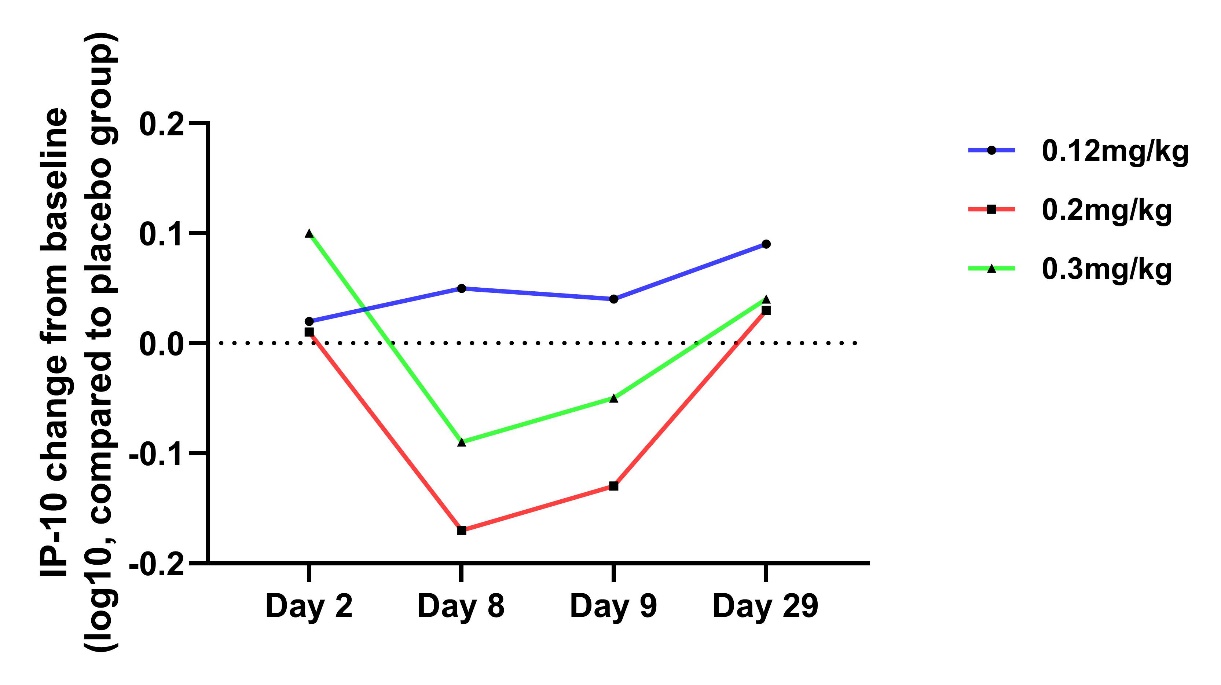


Figure. S29.

Change from baseline of TNF-α at each time point (log10 pg/mL, the baseline positive population)


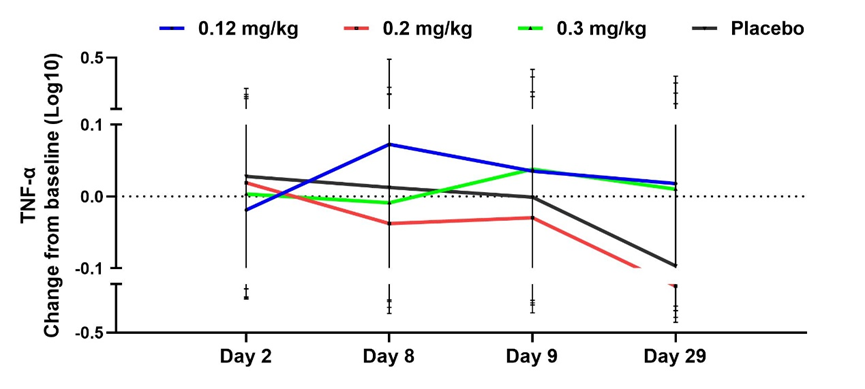


Figure. S30.

The difference of change from baseline between the meplazumab group and the placebo group of TNF-α (log10 pg/mL, the baseline positive population)


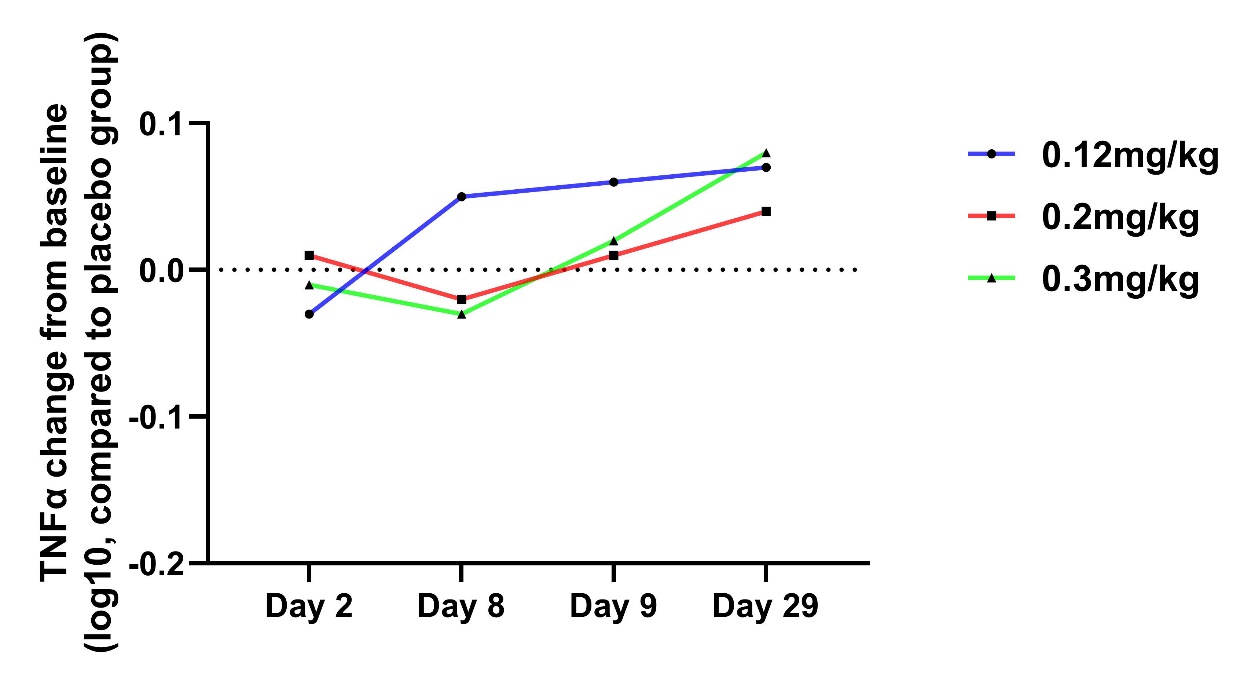


Figure. S31.

Change from baseline of IFN-γ at each time point (log10 pg/mL, the baseline positive population)


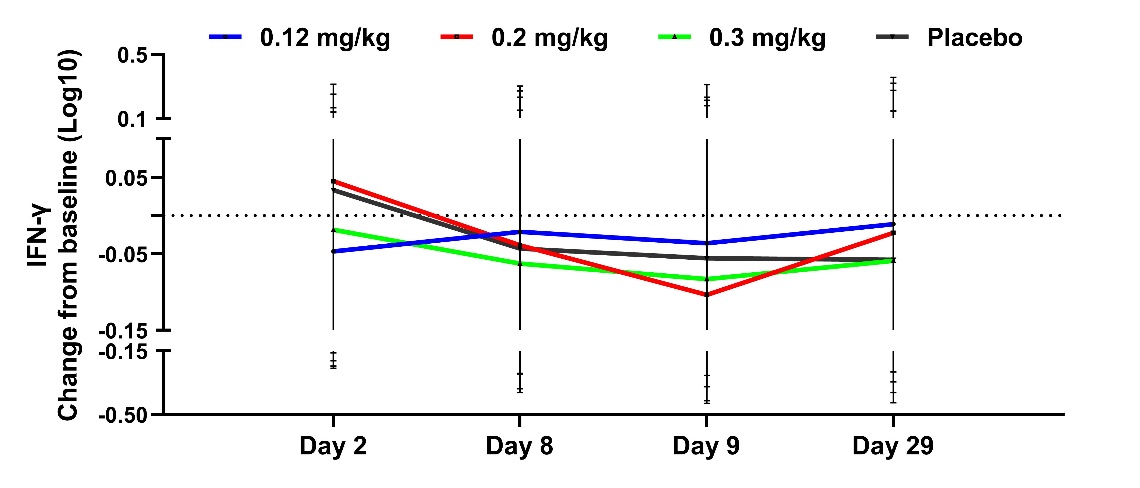


Figure. S32.

The difference of change from baseline between the meplazumab group and the placebo group of IFN-γ (log10 pg/mL, the baseline positive population)


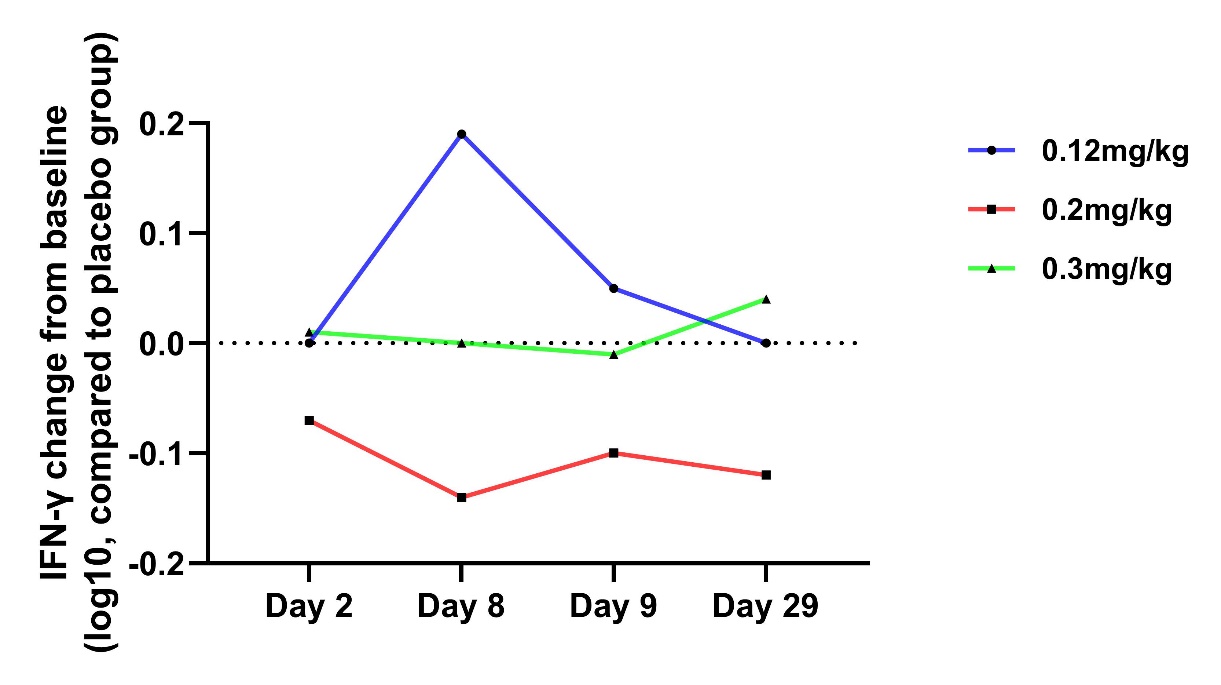

Supplement: Supplementary file 1 — Sigtrans_Supplementary_Materials [file 41392_2023_1323_MOESM1_ESM.docx]
