# Supplementary material for: Meplazumab in hospitalized adults with severe COVID-19 (DEFLECT): a multicenter, seamless phase 2/3, randomized, third-party double-blind clinical trial
Source: Signal Transduct Target Ther. 2023 Jan 30;8:46. doi: 10.1038/s41392-023-01323-9 (PMC9885411; doi:10.1038/s41392-023-01323-9)
Supplement: Supplementary file 2 — Sigtrans_Supplementary_Note_1 [file 41392_2023_1323_MOESM2_ESM.docx]

Title Page

Protocol Title: A Multicenter, Seamless, Randomized, Third-Party-Blind Clinical Trial to Evaluate the Safety and Efficacy of Meplazumab in Addition to Standard of Care for the Treatment of COVID‑19 in Hospitalized Adults

Protocol Number: MPZ-II-02

Amendment Number: 4

Product: Meplazumab for Injection

Short Title: Study to assess the effect of meplazumab on COVID‑19

Study Phase: Phase 2/3

Sponsor Name: Jiangsu Pacific Meinuoke Biopharmaceutical Co., Ltd. (PMBP)

**Legal Registered Address:** Jiangsu Pacific Meinuoke Biopharmaceutical Co., Ltd. (“PMBP”), having a place of business at No. 128 W Hehai Rd, Xinbei District, Changzhou, Jiangsu Province, China

Regulatory Agency Identifying Number(s): IND 149626, EudraCT 2020-003196-18

Date of Protocol: 15 November 2021

Sponsor Signatory:

Protocol Title: A Multicenter, Seamless, Randomized, Third-Party-Blind Clinical Trial to Evaluate the Safety and Efficacy of Meplazumab in Addition to Standard of Care for the Treatment of COVID‑19 in Hospitalized Adults

Protocol Number: MPZ-II-02

Date of Protocol: 15 November 2021

I have read this protocol in its entirety and agree to conduct the study accordingly:

| Xiaochun Chen  Chief Executive Officer |  | Date |
| --- | --- | --- |

Protocol Amendment Summary of Changes

Table 1 Document History

| Document | Date | Substantial | Region |
| --- | --- | --- | --- |
| Amendment 4 | 15-Nov-2021 | No | Global |
| Amendment 3 | 06-Sep-2021 | Yes | Global |
| Amendment 2 | 11-Jun-2021 | Yes | Global |
| Amendment 1 | 24-Nov-2020 | Yes | Global |
| Original Protocol | 23-Sep-2020 | - | - |

Amendment 4

The purpose of this prtocol amendment is to modify the sample size for Stage 1 to the protocol executed after protocol Amendment 3.

Table 2 Description of Changes in Amendment 4

| **Section # and Name** | **Description of Change** | **Brief Rationale** |
| --- | --- | --- |
| - 1. Synopsis   4.1 Overall Design  9.1 Sample Size Determination | The overall sample size for Stage 1 is changed from 216 subjects to about168 subjects, amongh which subjects will be allocated as 1:1:1:1 (42:42:42:42) into four arms to receive meplazumab or placebo. | Considering the global vaccination and the changing morbidity of COVID-19, especially the decline in Grade 3 and Grade 4 cases, sponsor determined to efficiently adjust sample size with reasonable power. |
| - 1. Schema | Data were corrected in Figure 1 (deleted text is in strikethrough and new text is set in boldface):  Stage 1: the subject number for each arm were changed to ~~n= 54~~ **n=42**;  Stage 2, interim analysis can be conducted when ~~216~~ **168** subjects reached on d29. | To update the data since the sample size was changed. |
| 9.1 Sample Size Determination | The following text is revised as follows (deleted text is in strikethrough and new text is set in boldface):  Using a step-down procedure for the comparisons between the dose groups and the placebo at a 2-sided alpha level of 0.05 each, ~~fifty-four~~ **forty-two** subjects per arm will power the study at ~~90~~ **81%** to distinguish the response rate at Day 29 of the dose groups from the placebo, assuming 80% response rate for the dose groups and 50% for the placebo. | As sample size is changed, the subject number for each arm will be forty-two.  As sample size is changed, the corresponding power will be 81%. |
| 12.6 Administrative Structure | CRO’s Project Manager was changed from Peipei Yang to Genevieve Liu. | For clarification. |

Amendment 3

The purpose of this protocol amendment is to modify the exclusion criterion which excludes the subject who received COVID-19 vaccines, and those who received kinase inhibitors or Janus kinase inhibitors.

Table 3 Description of Changes in Amendment 3

| **Section # and Name** | **Description of Change** | **Brief Rationale** |
| --- | --- | --- |
| 5.2 Exclusion Criteria | Exclusion criterion 7 is modified to remove the wording which excludes the subjects who received COVID-19 vaccines and kinase inhibitors or Janus kinase inhibitors. | Prohibition on COVID-19 vaccination was removed to enable study recruitment because COVID-19 vaccination has become mandatory in many countries/areas.  There’s still no clinical safety data on the combination use of meplazumab and COVID-19 vaccine; but from MoA perspective, there’s no safety concern for their use in combination.  Restrictions on Janus kinase inhibitors is removed because baricitinib has been approved for use in hospitalized patients with COVID‑19 in some countries/areas. |
| 6.5 Concomitant Therapy | The prohibition of kinase inhibitor and Janus kinase inhibitors is removed.  The prohibition of immunization with live or live-attenuated vaccine for 1 month following last study treatment administration is removed. | These are allowed now as they are removed from exclusion criterion 7  To allow the subjects to receive required immunization after end of the treatment. |
| 8.3 Adverse Event | Added a note that, any untoward condition resulting after receiving COVID-19 vaccine will be recorded as AE and followed up as per protocol | Since COVID-19 vaccine is allowed now, this note is added to clarify that all the untoward conditions resulting after receiving COVID-19 vaccine should be recorded as AEs. |
| 8.3.7 Pregnancy | Added a statement that pregnancies reported during the study should not be reported as AEs | To avoid pregnancy being reported as an AE. |

Amendment 2

Overall Rationale for the Amendment:

The purpose of this protocol amenment is to address FDA comments and to implement 4 protocol clarification notes to the protocol executed after protocol Amendment 1.

Table 4 Description of Changes in Amendment 2

| **Section # and Name** | **Description of Change** | **Brief Rationale** |
| --- | --- | --- |
| - 1. Synopsis   9.3.1. Efficacy Analyses | The following text is revised as follows (added text is in bold):  Time to event for subjects who die or withdraw from the study **during the evaluation period (Day 1 to Day 29)** for other reasons will be censored at the end of analysis period (i.e., Day 29). | As per FDA recommendation |
| 1.3. Schedule of Activities  8.0. Study Assessments and Procedures | Window period for Screening is increased to 2 days from 1 day. Now the Screening visit will be between Day -2 and Day 1. | Some local safety laboratories require longer turnaround time for test results. Screening period is extended to 2 days to allow all screening result to be available prior to randomization. |
| 1.3. Schedule of Activities | A new column for Day 8 is created and Day 8 procedures for non-hospitalized subjects are added.  The following note is added in footnote: Examinations every 6 hours thereafter until 24 hours after administration is not required for non-hospitalized patients) | In the current Schedule of Activities, the Day 8 procedures for non-hospitalized subjects is not clear. Hence created a new column for Day 8 to specify the procedures required. |
| 5.1. Inclusion Criteria | Inclusion criterion 1 is revised as follows (deleted text is in strikethrough and added text is in bold):  Adults (≥18 years) with laboratory-confirmed SARS-CoV-2 infection as determined by PCR or other commercial or public health assay, which is FDA cleared **or have use authorization in country that subject resides in** ~~approved for emergency use~~ (test results must be obtained within 72 hours of Day 1). | To clarify that local government authorized method is also acceptable. |
| 5.2. Exclusion Criteria | Exclusion criteria 2 and 3 are combined to read: Subject with evidence of critical COVID‑19 illness, defined by at least 1 of the following: requiring invasive mechanical ventilation or extracorporeal membrane oxygenation (ECMO); shock (defined by systolic blood pressure <90 mmHg, or diastolic blood pressure <60 mmHg, or requiring vasopressors); or multi-organ dysfunction/failure. | Current exclusion criterion 2 regarding respiratory failure is wrong, since any need for oxygen supplementation or ventilation defines it, and also contradicts the inclusion of grade 3 to 4 subjects. |
| 5.2. Exclusion Criteria | Exclusion criterion 8 (now 7) is revised as follows (deleted text is in strikethrough):  Use of anticancer, antitransplant rejection, immunomodulatory biological drug or kinase inhibitor (eg, tocilizumab, sarilumab), Janus kinase inhibitors (within 30 days of enrollment or 5 times the half-life [whichever is longer]), or COVID-19 vaccines ~~or COVID 19 non approved treatment~~. | The term “COVID-19 non-approved treatment” is vague and it implies off-label use of approved medication which is inconsistent with Section 6.5 regarding excluded medication. |
| 5.2. Exclusion Criteria | Exclusion criterion 9 (now 8) is revised as follows (added text is in bold):  Chronic glucocorticosteroid use equivalent to daily oral prednisone >10 mg per day **for more than 3 months** (10 mg oral prednisone every other day is allowed). | To provide a clear definition of chronic use of steroid. |
| 12.3. Clinical Laboratory Tests | Fasting glucose is removed from the clinical chemistry laboratory examination. | Fasting glucose is not mandatory and this is inconsistent with Section 8.2.4 where it states that fasting is not required before collection of blood samples for clinical laboratory evaluations. |

Amendment 1

Overall Rationale for the Amendment:

The protocol was amended primarily to add missing information and correct errors and inconsistencies.

Table 5 Description of Changes in Amendment 1

| **Section # and Name** | **Description of Change** | **Brief Rationale** |
| --- | --- | --- |
| Title Page  and Appendix 12.5. Signature of Investigator | The company name “Jiangsu Pacific Meinuoke Biopharmaceuticals (PMBP)” changed to “Jiangsu Pacific Meinuoke Biopharmaceutical Co., Ltd. (PMBP)” | For clarification. |
| - 1. Synopsis and throughout the document for consistency | The following text revised as follows (added text is set in boldface):  “This is a multicenter, seamless, randomized, third-party-blind study to evaluate the safety and efficacy of meplazumab for the treatment of COVID‑19 in hospitalized adults (≥18 years). Neither the subject nor the investigator shall be aware of ~~the study drug identity~~ **whether the subject receives the study drug or placebo**, as the study drug**/placebo** is ~~dispensed~~ **prepared** by a unblinded third party (eg, a pharmacist or nurse) **and administered by the authorized blinded site staff**. | To add clarification of the term “third‑party blind” study design. |
| - 1. Synopsis   9.3.1 Efficacy Analyses | The following text was updated (new text is set in boldface):  “The time to event endpoints, including time to sustained clinical improvement by Day 29 from treatment start date, will be compared between the treatment arms~~. stratifying for age group (age <65 years versus ≥65 years) and baseline severity grade, with death handled as competing risk, and the equivalence of cumulative incidence curve will be tested using the Gray’s test at the 2-sided alpha level of 0.05~~. **Time to event for subjects who die or withdraw from the study for other reasons will be censored at the end of analysis period (i.e., Day 29). The Kaplan Meier estimator will be used, Kaplan Meier curves will be plotted for each treatment arm, and the log-rank test will be used for comparing the treatment arms at the 2 -sided alpha level of 0.05**. ~~The unstratified cumulative incidence curve will be plotted for each treatment arm.~~ ~~Additionally, Cox regression model will be used to model the subdistribution hazard ratio (HR) between treatment arms under the log-rank test Fine and Gray’s competing risk framework, with death handed as competing risk;~~ **In addition, Cox regression model will be used by adjusting for age group (age <65 years versus ≥65 years) and baseline severity grade**; the HR and its 95% CI will be reported.” | To correct the information. |
| 1.1. Synopsis | In objectives of Stage 1, “2 doses” were changed to “3 doses” | Corrected the incorrect number of doses. |
|  | In the subsection “Interim Analysis”, “2 dose arms” was changed to “3 dose arms” |  |
| 1.3. Schedule of Activities | - “Urinalysis” was added to safety laboratory evaluations. - In the table footnote i), the reference to Section 8.1 was changed to Section 8.1.1 - The text in the row “Low flow oxygen supplementation days (<40%)” was changed to “Supplemental oxygen use days (low-flow nasal cannula, simple face mask). - The text in the row “High flow oxygen requirement days (>40%)” was changed to “High flow oxygen device use days (Venturi mask, high-flow nasal cannula).” - The text in the row “Ventilator requirement days” was changed to “Invasive or non-invasive mechanical ventilation use days.” - The rows “invasive and non-invasive mechanical ventilation days” were split into 2 separate rows. - The following change in the footnote l) was added (the new text is set in boldface):   “Complete hematology, serum chemistry, lactate hydrogenase (LDH), cardiac troponin, D-dimer, ferritin, **bicarbonate, haptoglobin** outlined in Appendix 12.3.” | Corrected the incorrect references, added missing information and added clarification. |
| 5.1. Inclusion Criteria | In inclusion criteria #5, reference to Appendix 12.3 was changed to Appendix 12.4 | Corrected the incorrect reference |
| 5.2. Exclusion Criteria | For exclusion criterion #8, added the following text (bold):  “Use of anticancer, antitransplant rejection, immunomodulatory biological drug or kinase inhibitor (eg, tocilizumab, sarilumab), Janus kinase inhibitors (within 30 days of enrollment or 5 times the half‑life [whichever is longer]), COVID‑19 vaccines or COVID-19 non approved treatment.” | To add clarification that COVID-19 vaccines or COVID‑19 non‑approved treatment will not be allowed. |
| 6.1. Study Treatments Administered | The following text:  “Meplazumab will be provided in the form of a 10-mg/vial freeze‑dried powder containing 1.60 g histidine, 3.08 g histidine hydrochloride, 50.0 g sucrose, 70.0 g mannitol, and 1.0 g polysorbate 80”  was changed to:  “Meplazumab will be provided in the form of a 10-mg/vial freeze-dried powder containing 1.60 mg histidine, 3.08 mg histidine hydrochloride, 50.0 mg sucrose, 70.0 mg mannitol, and 1.0 mg polysorbate 80.” | To correct the typo (correct “mg” – not “g”). |
| 6.3.3. Unblinding Procedure | The following text:  “If any site personnel are unblinded to a subject’s treatment, study treatment must be stopped immediately, and the subject must be withdrawn from the study.”  was changed to:  “If any site personnel are unblinded to a subject’s treatment, the subject must be discontinued from the study treatment. If any amount of study intervention was administered, follow procedures according to the SoA.” | For clarification. |
| 7.1. Discontinuation of Study Treatment | The following text:  “Subjects who discontinue study treatment will continue to be followed for safety in accordance with the SoA visit procedures up to End-of-Study Day 84.”  was changed to:  “Subjects who discontinue study treatment will remain in the study for follow-up and any further evaluations that need to be completed as described in the SoA.” | For clarification. |
| 8.2.3. Electrocardiogram | Added text:  “Centralized ECG data collection, analysis, and reporting will be used in this study, permitting secure central interpretation, storage, and retrieval of the ECG data. Instructions for the collection (eg, equipment), transmission, and archiving of ECG data will be agreed upon with the central laboratory and summarized.” | Added the missing information. |
| 8.3.1. Documentation and Reporting of AEs | The correction made as follows:  “Relevant AE data will be obtained at the study visits, based on information spontaneously provided by the subject and/or thorough questioning of the subject and through diary card data recorded by the subject.” | Removed the incorrect information, as no subject diary will be used for this study. |
| 9.4. Interim Analyses | The correction made as follows:  “Dose selection at the interim analysis will be made by monitoring both the efficacy data including response rates of 2 dose arms, time to event endpoints, ranked outcome trajectory endpoint, and safety data.” | Removed the incorrect information, as ranked outcome trajectory endpoint was viewed as an exploratory endpoint. |
| 12.2. Regulatory, Ethical, and Study Oversight Considerations | The following text was removed:  “The ICF will contain a separate section that addresses the use of remaining mandatory samples for optional exploratory research”; “A separate signature will be required to document a subject’s agreement to allow any remaining specimens to be used for exploratory research. Subjects who decline to participate in this optional research will not provide this separate signature.” | Removed the incorrect information, as there will be no optional exploratory research; all samples are mandatory. |
| 12.3. Clinical Laboratory Tests | Added text:   - “Urinalysis: specific gravity, pH, glucose, protein, blood, ketones, bilirubin, urobilinogen, nitrite, leukocyte esterase by dipstick, microscopic examination.” - “Bicarbonate” - “Haptoglobin” | Added missing laboratory tests |

Table of Contents

[Protocol Amendment Summary of Changes 3](#_Toc81833741)

[Table of T
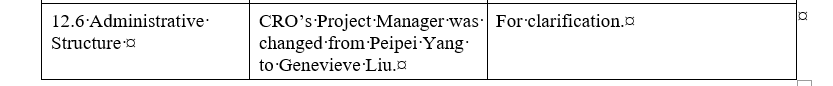
ables 15](#_Toc81833742)

[Table of Figures 16](#_Toc81833743)

[1.0 Protocol Summary 17](#_Toc81833744)

[1.1 Synopsis 17](#_Toc81833745)

[1.2 Schema 25](#_Toc81833746)

[1.3 Schedule of Activities 26](#_Toc81833747)

[2.0 Introduction 31](#_Toc81833748)

[2.1 Study Rationale 31](#_Toc81833749)

[2.2 Background 31](#_Toc81833750)

[2.3 Benefit/Risk Assessment 32](#_Toc81833751)

[3.0 Objectives and Endpoints 35](#_Toc81833752)

[4.0 Study Design 39](#_Toc81833753)

[4.1 Overall Design 39](#_Toc81833754)

[4.2 Scientific Rationale for Study Design 40](#_Toc81833755)

[4.3 Justification for Dose 41](#_Toc81833756)

[4.4 End of Study Definition 44](#_Toc81833757)

[5.0 Study Population 45](#_Toc81833758)

[5.1 Inclusion Criteria 45](#_Toc81833759)

[5.2 Exclusion Criteria 46](#_Toc81833760)

[5.3 Screen Failures 46](#_Toc81833761)

[6.0 Study Treatment 47](#_Toc81833762)

[6.1 Study Treatment(s) Administered 47](#_Toc81833763)

[6.2 Preparation/Handling/Storage/Accountability 48](#_Toc81833764)

[6.3 Measures to Minimize Bias: Randomization and Blinding 49](#_Toc81833765)

[6.3.1 Randomization Code Creation and Storage 49](#_Toc81833766)

[6.3.2 Investigational Drug Blind Maintenance 49](#_Toc81833767)

[6.3.3 Unblinding Procedure 50](#_Toc81833768)

[6.4 Study Treatment Compliance 50](#_Toc81833769)

[6.5 Concomitant Therapy 51](#_Toc81833770)

[6.6 Treatment after the End of the Study 52](#_Toc81833771)

[7.0 Discontinuation of Study Treatment and Subject Discontinuation/Withdrawal 53](#_Toc81833772)

[7.1 Discontinuation of Study Treatment 53](#_Toc81833773)

[7.2 Subject Discontinuation/Withdrawal from the Study 54](#_Toc81833774)

[7.3 Lost to Follow-up 54](#_Toc81833775)

[8.0 Study Assessments and Procedures 55](#_Toc81833776)

[8.1 Efficacy Assessments 56](#_Toc81833777)

[8.1.1 NEWS2 Score 56](#_Toc81833778)

[8.1.2 Clinical Assessment Scales 58](#_Toc81833779)

[8.2 Safety Assessments 58](#_Toc81833780)

[8.2.1 Physical Examinations 58](#_Toc81833781)

[8.2.2 Vital Signs 59](#_Toc81833782)

[8.2.3 Electrocardiogram 59](#_Toc81833783)

[8.2.4 Laboratory Assessments 59](#_Toc81833784)

[8.3 Adverse Events 59](#_Toc81833785)

[8.3.1 Documentation and Reporting of AEs 62](#_Toc81833786)

[8.3.2 Assessment of Intensity and Causality of Adverse Events 63](#_Toc81833787)

[8.3.3 Follow-up of AEs 64](#_Toc81833788)

[8.3.4 Serious Adverse Events 64](#_Toc81833789)

[8.3.5 Adverse Events of Special Interest 66](#_Toc81833790)

[8.3.6 Regulatory Reporting Requirements for SAEs 67](#_Toc81833791)

[8.3.7 Pregnancy 67](#_Toc81833792)

[8.4 Treatment of Overdose 68](#_Toc81833793)

[8.5 Pharmacokinetics 68](#_Toc81833794)

[8.6 Pharmacodynamics 70](#_Toc81833795)

[8.7 Biomarkers 71](#_Toc81833796)

[8.7.1 Immunogenicity Assessments 71](#_Toc81833797)

[8.7.2 Other Research 71](#_Toc81833798)

[8.8 Health Economics/Medical Resource Utilization and Health Economics 72](#_Toc81833799)

[9.0 Statistical Considerations 73](#_Toc81833800)

[9.1 Sample Size Determination 73](#_Toc81833801)

[9.2 Populations for Analyses 73](#_Toc81833802)

[9.3 Statistical Analyses 74](#_Toc81833803)

[9.3.1 Efficacy Analyses 74](#_Toc81833804)

[9.3.2 Safety Analyses 76](#_Toc81833805)

[9.3.3 Other Analyses 76](#_Toc81833806)

[9.3.4 Missing Data 77](#_Toc81833807)

[9.4 Interim Analyses 77](#_Toc81833808)

[9.5 Data Monitoring Committee 78](#_Toc81833809)

[10.0 DATA HANDLING, RECORDING and QUALITY ASSURANCE 79](#_Toc81833810)

[10.1 Data Handling and Record Keeping 79](#_Toc81833811)

[10.2 Source Documents 79](#_Toc81833812)

[10.3 Data Quality Assurance 79](#_Toc81833813)

[11.0 References 81](#_Toc81833814)

[12.0 Appendices 83](#_Toc81833815)

[12.1 Abbreviations 83](#_Toc81833816)

[12.2 Regulatory, Ethical, and Study Oversight Considerations 85](#_Toc81833817)

[Regulatory and Ethical Considerations 85](#_Toc81833818)

[Financial Disclosure 86](#_Toc81833819)

[Insurance 86](#_Toc81833820)

[Informed Consent Process 87](#_Toc81833821)

[Data Protection 87](#_Toc81833822)

[Dissemination of Clinical Study Data 88](#_Toc81833823)

[Study and Study Center Closure 88](#_Toc81833824)

[Publication Policy 88](#_Toc81833825)

[12.3 Clinical Laboratory Tests 89](#_Toc81833826)

[12.4 Contraceptive Guidance and Collection of Pregnancy Information 90](#_Toc81833827)

[12.5 Signature of Investigator 95](#_Toc81833828)

[12.6 Administrative Structure 96](#_Toc81833829)

Table of Tables

[Table 1 Document History 3](#_Toc87635123)

[Table 2 Description of Changes in Amendment 4 3](#_Toc87635124)

[Table 3 Description of Changes in Amendment 3 4](#_Toc87635125)

[Table 4 Description of Changes in Amendment 2 5](#_Toc87635126)

[Table 5 Description of Changes in Amendment 1 7](#_Toc87635127)

[Table 6 Study Treatment Details 48](#_Toc87635128)

[Table 7 The National Early Warning Score (NEWS) 2 57](#_Toc87635129)

Table of Figures

[Figure 1 Study Schema 25](#_Toc81833835)

# Protocol Summary

## Synopsis

Protocol Title: A Multicenter, Seamless, Randomized, Third-Party-Blind Clinical Trial to Evaluate the Safety and Efficacy of Meplazumab in Addition to Standard of Care for the Treatment of COVID‑19 in Hospitalized Adults

Short Title: Study to assess the effect of meplazumab on COVID‑19

Rationale:

Meplazumab is a humanized anti-CD147 IgG2 monoclonal antibody which is expected to block the binding of the severe acute respiratory syndrome coronavirus 2 (SARS‑CoV‑2) spike protein to the human host-cell-expressed CD147, thereby blocking entry of SARS‑CoV‑2 into human tissue. This expectation is based on in vitro functional studies using Vero E6 cells infected with SARS-CoV-2 that demonstrated effective meplazumab mediated virus gene copy number inhibition upwards of 90% as evaluated by quantitative polymerase chain reaction. Meplazumab may also inhibit COVID-19 associated cytokine storm syndrome based on inhibition of the pro‑inflammatory factor CyPA host-cell CD147 interaction.

| Objectives and Endpoints | |
| --- | --- |
| Objectives | Endpoints |
| Stage 1*:* *No primary endpoint will be defined for Stage 1.* | |
| To evaluate the efficacy of 3 selected doses of meplazumab plus Standard of Care (SoC) compared to control plus SoC in subjects hospitalized with coronavirus disease 2019 (COVID‑19) infection. | Dose selection endpoint: Determine an optimal dose based on response rate at Day 29 by sustained clinical improvement of 2 points (from randomization) on 6-point ordinal scale. |
| **Primary**: *Primary endpoint will be determined based on adaptation during the interim analysis of Stage 1 data.* |  |
| Stage 2: To evaluate the efficacy of the selected dose of meplazumab plus SoC (based on adaptation from Stage 1) compared to control plus SoC, in subjects hospitalized with COVID‑19 infection. | Primary endpoint will be determined at interim analysis.   - Time to sustained clinical improvement (days; Time frame: Day 1 through Day 29) of at least 2 points (from randomization) on a 6‑point ordinal scale, (where sustained improvement is improvement without subsequent worsening), or live discharge from the hospital, whichever comes first - Response rate, as defined by a sustained improvement of 2 points on a 6-point ordinal scale, at Day 29 - Mortality at Day 29 - Proportion of subjects alive and discharged without supplemental oxygen at Day 29 |
| **Secondary:** |  |
| - To evaluate response rate | - Response rate (number and %) by treatment arm at Day 2, 8, and 15, as defined by an improvement of 2 points on a 6-point ordinal scale |
| - To evaluate live discharge | - Proportion of subjects alive and discharge without supplemental oxygen at Day 15 and Day 57 |
| - To evaluate the safety of meplazumab as add-on therapy to SoC in subjects with COVID‑19 | - Physical examination - Clinical laboratory examinations - Vital signs (BP/HR/temperature/respiratory rate/saturation [finger oxygen SpO_2_]) - 12-lead electrocardiograms (ECGs) - Virologic load (quantitative PCR for COVID‑19 in nasopharyngeal [NP] swab) on Day  1 (predose), 3, 5, 8 (predose), and 9 or 10, 29 and 57(optional) - Antidrug antibody (ADA) titers (predose, end-of-treatment [Day 29], follow-up visit [Day 57] and end-of-study visit day) - Adverse events (AEs) of special interest: disease-related secondary infection complications, hemolysis, Grade 4 (Common Terminology Criteria for Adverse Events V5 [CTCAE]) neutropenia and lymphopenia, and anaphylactic reactions defined by Clinical Criteria for Diagnosing Anaphylaxis^(11)^; 20% decline in SpO_2_ between start and end of 1-hr study treatment infusion; ALT or AST >3 x ULN AND TBL >2 x ULN; Evidence of red blood cell (RBC) hemolysis as defined by 2 of the following 3 findings:   - Anemia that is not due to another obvious cause;   - Increased reticulocyte count that is not explained by an obvious cause;   - Signs of RBC destruction, such as increased lactate dehydrogenase (LDH), low haptoglobin ≤25 mg/dL, increased unconjugated bilirubin. |
| - To evaluate overall mortality | - Mortality at Days 15 and 57 - Time from treatment start date to death. |
| - To evaluate clinical recovery of COVID‑19 disease | - Time to sustained recovery (days; Time frame: Day 1 through Day 29) as defined by first day on which 1 of the following 2 categories is achieved using the 6-point ordinal scale:   1. Not hospitalized  2. Hospitalized, not requiring supplemental oxygen |
| - To evaluate the number of oxygen-free days | - Duration (days) of oxygen use and oxygen‑free days |
| - To evaluate ventilator-free days, incidence, and duration of new mechanical ventilation use | - Duration (days) of mechanical ventilation and mechanical ventilation-free days - Incidence of new mechanical ventilation use and duration (days) of new mechanical ventilation use |
| - To evaluate clinical relapse of COVID‑19 | - Incidence of new mechanical ventilation use after mechanical ventilation extubation period of 24 hours - Incidence of rehospitalization following hospital discharge |
| - To evaluate ICU and hospitalization length | - Duration (days) of ICU and hospitalization |
| Exploratory |  |
| - To evaluate pharmacokinetic (PK) exposure to meplazumab - To evaluate pharmacodynamic (PD) response to administration of meplazumab - To explore potential exposure-response relationships - To evaluate the efficacy of the selected dose using a composite ranked outcome trajectory score | - Meplazumab: serum and blood cell concentrations and derived PK parameters if calculable by noncompartmental analysis, PK parameters may include but are not limited to maximum observed concentration (C_max_), time to C_max_ (t_max_), area under the concentration-time curve (AUC) calculated to the last quantifiable concentration and to infinity [AUC_(0‑last)_, AUC_(0‑inf)_], elimination half-life (t½), volume of distribution (Vz, Vss), and systemic clearance (CL). - PD endpoints: changes from baseline in cytokine, and chemokines related to inflammatory and immune status including CyPA, IL‑6, IL‑8 MCP‑1, MIP‑1α, MIP-1β, TNF‑α, IFN‑γ, IL‑1RA, IL‑2, IL-2Rα, IL-4, IL‑7, IL‑10, IL-15, IL-17A, IL-12 p70, G‑CSF, M‑CSF, CXCL10, hsCRP. - Graphical (and statistical, if appropriate) display of exposure versus response for select endpoints. - Ranked outcome trajectory by Day 29 using the ordinal scale at Days 8, 15, and 29   For each subject, the below items are calculated and sorted,   1. The worst score over the 28 days; 2. The last recorded score; 3. The duration at the worst score; 4. The best score that occurs after the worst score; 5. The duration of (4) is observed.  - Rank by ascending order of 1, 2, 3, 4 then descending order of 5. |

Abbreviations: ALT = alanine aminotransferase; AST = aspartate aminotransferase; BP = blood pressure; CXCL10 = C–X–C motif chemokine 10; CyPA = cyclophilin A; G‑CSF = granulocyte colony‑stimulating factor; HR = heart rate; hsCRP = high-sensitivity C‑reactive protein; ICU = intensive care unit; IFN‑γ = interferon gamma; IL‑1 RA = interleukin‑1 Receptor Antagonist; IL‑2 = interleukin‑2; IL-2 Rα (Interleukin-2 Receptor alpha); IL‑4 = interleukin‑4; IL‑6 = interleukin‑6; IL‑7 = interleukin‑7; IL‑8 = interleukin‑8; IL‑10 = interleukin‑10; IL‑15 = interleukin‑15; IL‑17A = interleukin‑17A; IL-12p70 = interleukin12p70; MCP‑1 = monocyte chemoattractant protein‑1; M‑CSF = macrophage colony‑stimulating factor; MIP‑1α = macrophage inflammatory protein‑1 alpha; MIP-1β = macrophage inflammatory protein-1 beta; NP = nasopharyngeal; SpO_2 =_ peripheral capillary oxygen saturation; TBL = total bilirubin; TNF‑α = tumor necrosis factor alpha; ULN = upper limit of normal.

Overall Design:

This is a multicenter, seamless, randomized, third-party-blind study to evaluate the safety and efficacy of meplazumab for the treatment of COVID‑19 in hospitalized adults (≥18 years). Neither the subject nor the investigator shall be aware of whether the subject receives the study drug or placebo, as the study drug/placebo is prepared by an unblinded third party (eg, a pharmacist or nurse) and administered by an authorized blinded site staff.

Enrollment of subjects will be stopped once the total number of planned subjects have completed the Stage 1 Day 29 visit procedures. Once the interim analysis of Stage 1 study data is complete and the Independent Data Monitoring Committee (IDMC) has recommended the meplazumab dose that is safe and effective to carry forward into Stage 2, the study will resume subject enrollment. A summary of the key Stage 1 interim analysis results will be sent to the relevant Health Authorities involved, if requested.

Number of Investigators and Study Centers:

There will be 15 to 20 Investigators, at 12 to 20 study centers globally, participating in this study.

Number of Subjects:

Subjects will be screened 1 to 2 days before randomization.

Stage 1: Approximately 168 subjects will be randomized and allocated 1:1:1:1 (42:42:42:42) to receive meplazumab low dose, meplazumab medium dose, meplazumab high dose, or control. An interim analysis will be conducted to select the optimal dose of meplazumab compared with the control group based on response rates of clinical improvement at Day 29.

Stage 2: 240 more subjects will be randomized and allocated 2:1 (160:80) to receive either the optimal meplazumab dose determined after Stage 1, or control. At interim analysis, primary endpoint, sample size calculation for Stage 2 will be re‑evaluated based on the observed outcomes at Stage 1 and will be capped at 300 subjects total.

Treatment Groups and Duration:

Study duration for each subject will be 84±7 days from randomization in each stage.

**Treatment Administration**

| Study Treatment Name: | Meplazumab low dose | Meplazumab medium dose | Meplazumab high dose | Control |
| --- | --- | --- | --- | --- |
| Dosage Formulation: | Solution for IV infusion | Solution for IV infusion | Solution for IV infusion | Sterile normal saline (0.9%) for IV infusion |
| Unit Dose Strength(s)/Dosage Level(s): | First dose: 0.12 mg/kg – Day 1  second dose: control – Day 8* | First dose: 0.2 mg/kg – Day 1  second dose: 0.2 mg/kg – Day 8 | First dose: 0.3 mg/kg – Day 1  second dose: 0.3 mg/kg – Day 8 | First dose: control – Day 1  second dose: control – Day 8 |
| Route of Administration | IV | IV | IV | IV |
| Dosing Instructions: | 1-hour infusion, 100 mL | 1-hour infusion, 100 mL | 1-hour infusion, 100 mL | 1-hour infusion, 100 mL |
| Packaging and Labeling | Study treatment will be provided in kits that each contain 1, 10 mg vial of meplazumab. kits and vials will be labeled per country requirement. | Study treatment will be provided in kits that each contain 1, 10 mg vial of meplazumab. kits and vials will be labeled per country requirement. | Study treatment will be provided in kits that each contain 1, 10 mg vial of meplazumab. kits and vials will be labeled per country requirement. | Study treatment will be provided by site in standard local container, labeled per country requirement. |
| Manufacturer | Jiangsu Pacific Meinuoke Biopharmaceutical Co., Ltd.; control as described in the study‑specific Pharmacy Manual | Jiangsu Pacific Meinuoke Biopharmaceutical Co., Ltd. | Jiangsu Pacific Meinuoke Biopharmaceutical Co., Ltd. | As described in the study‑specific Pharmacy Manual |

Abbreviations: IV = intravenous; kg= kilogram; mg = milligram; mL = milliliter.
* Sterile normal saline (0.9%) for IV infusion

Statistical methods:

Efficacy

| Endpoint | Statistical Analysis Methods |
| --- | --- |
| Primary | Stage 1: There is no primary endpoint in Stage 1.  Dose selection endpoint: Response rate at Day 29 of the low, medium, and high dose groups will be compared against that of the placebo group using Chi-Square test. The comparison of the response rate between the dose groups and the placebo will be tested with a step-down procedure at the 2-sided alpha level of 0.05 each, and will proceed in the following order, high versus placebo, medium versus placebo, and low versus placebo. If one preceding testing fails to be rejected, all the following will not be tested.  Subjects in the Stage 1 intent-to-treat (ITT) set will be used.  Primary endpoints for Stage 2 will be determined based on Stage 1 results.  Stage 2:  The analysis of response rate at Day 29 will be conducted between the selected dose group and control using Cochran–Mantel–Haenszel (CMH) statistic, stratifying for age group (age <65 years versus ≥65 years), and baseline severity grade, and additional stratification factors if any as determined after evaluation of Stage 1 data. The p value associated with the CMH statistic will be compared at the 2-sided 0.05 alpha level.  Sensitivity analyses on the response rate at Day 29 will be to fit the response variable using logistic regression, including treatment, baseline, baseline and treatment interaction, age group (age <65 years versus ≥65 years), and age group and treatment interaction as fixed effects. Model-based point estimates for the treatment effects, 95% confidence intervals (CIs), and p-values will be calculated.  Other candidate binary endpoints, including mortality at Day 29 and proportion of subjects alive and discharged without supplemental oxygen at Day 29, will be analyzed using the same CMH test and logistic regression as above.  The time to event endpoints, including time to sustained clinical improvement by Day 29 from treatment start date, will be compared between the treatment arms. Time to event for subjects who die or withdraw from the study during the evaluation period (Day 1 to Day 29) for other reasons will be censored at the end of analysis period (i.e., Day 29). The Kaplan Meier estimator will be used, Kaplan Meier curves will be plotted for each treatment arm, and the log-rank test will be used for comparing the treatment arms at the 2-sided alpha level of 0.05.. In addition, Cox regression model will be used by adjusting for age group (age <65 years versus ≥65 years) and baseline severity grade; the HR and its 95% CI will be reported.  Other continuous endpoints will be summarized with descriptive statistics such as mean, standard deviation (SD), median, minimum, and maximum.  Subgroup analyses may be performed by age group, gender, and race, and concomitant antiviral agents.  Subjects in the Stage 2 ITT set will be used. |
| Secondary | Stage 1: There was no secondary endpoint in Stage 1.  Stage 2: Binary endpoints, including response rates at Days 2, 8, and 15 and mortality rates at Days 15, and 57, proportion of live discharges at Day 15 and 57, will be using the same CMH test and logistic regression model as in the primary endpoint.  Time to event endpoints, including time to clinical improvement, time to death, and time to live discharge, from treatment start date will use the same survival analysis model as in the primary analysis.  Other continuous endpoints will be summarized with descriptive statistics such as mean, SD, median, minimum, and maximum. |
| Exploratory | - To evaluate pharmacokinetic (PK) exposure to meplazumab - To evaluate pharmacodynamic (PD) response to administration of meplazumab - To explore potential exposure-response relationships - To evaluate the efficacy of the selected dose using a composite ranked outcome trajectory score |

**Safety Analysis**

All safety analyses will be performed on the Safety Analysis Set.

All AEs reported in this study will be coded using the currently available version of the Medical Dictionary for Regulatory Activities (MedDRA®). Coding will be to lowest level terms. The preferred term (PT), and the primary system organ class (SOC) will be listed.

Summaries of all treatment emergent AEs (TEAEs) by treatment group will include:

- The number (n) and percentage (%) of subjects with at least 1 TEAE by SOC and PT
- TEAEs by severity, presented by SOC and PT
- TEAEs by relationship to treatment (related, not related), presented by SOC and PT
- Treatment-emergent AEs of special interest (AESIs) (defined with a PT or a prespecified grouping)

Deaths and other serious AEs (SAEs) will be listed and summarized by treatment group.

Treatment-emergent adverse events leading to permanent treatment discontinuation will be listed and summarized by treatment group.

The number and proportion of subjects with normal/abnormal laboratory tests or different grades on Day 3, Day 8, Day 15, and Day 29 (or day of discharge) will be presented as shift tables by the baseline status (Day 1).

Potentially clinically significant abnormality (PCSA) values are defined as abnormal values considered medically important by the Sponsor according to predefined criteria/thresholds based on literature review. The incidence of PCSA vital signs on Day 8, Day 15, and Day 29 (or day of discharge) will be summarized overall and by their baseline normality status (normal/abnormal).

The number and proportion of subjects with confirmed positive ADA results and with confirmed positive neutralizing activity.

**Interim Analysis**

Dose selection at the interim analysis will be made by monitoring both the efficacy data including response rates of 3 dose arms, time to event endpoints, and safety data. The primary endpoints, and sample size evaluation will be re‑evaluated based on the data collected at Stage 1. Futility analysis comparing the dose arms to control will be conducted after Stage 1. Sample size for Stage 2 will be re‑evaluated based on Stage 1 results and the selected primary endpoint.

Meplazumab concentration (as available) will be listed and summarized by scheduled collection time and treatment for the pharmacokinetic analysis set (PKS). Similarly, meplazumab PK parameters (as available) will be listed and summarized by scheduled collection time and treatment for the pharmacokinetic parameter analysis set (PKPS). An interim PK analysis during Stage 2 is not planned. Pharmacodynamic/biomarker endpoints may be evaluated during the interim analysis at the end of Stage 1; however, if performed, the decision on dose selection will be based on the efficacy results.

Stage 2 interim analysis will be determined and described after the primary endpoint is selected.

Data Monitoring Committee:

An independent data monitoring committee (IDMC) will be responsible for safeguarding the safety of subjects and for general oversight of the study conduct. The IDMC will have the following responsibilities:

- Review results of the interim analysis
- Review of interim safety data at regular intervals while subjects remain on study treatment
- Review of individual safety issues as requested by the Medical Monitor during the course of the study with the goal of recommending an appropriate course of action.

The operating principles, roles and responsibilities of the IDMC will be fully described in the IDMC Charter*.*

## Schema

Figure 1 Study Schema


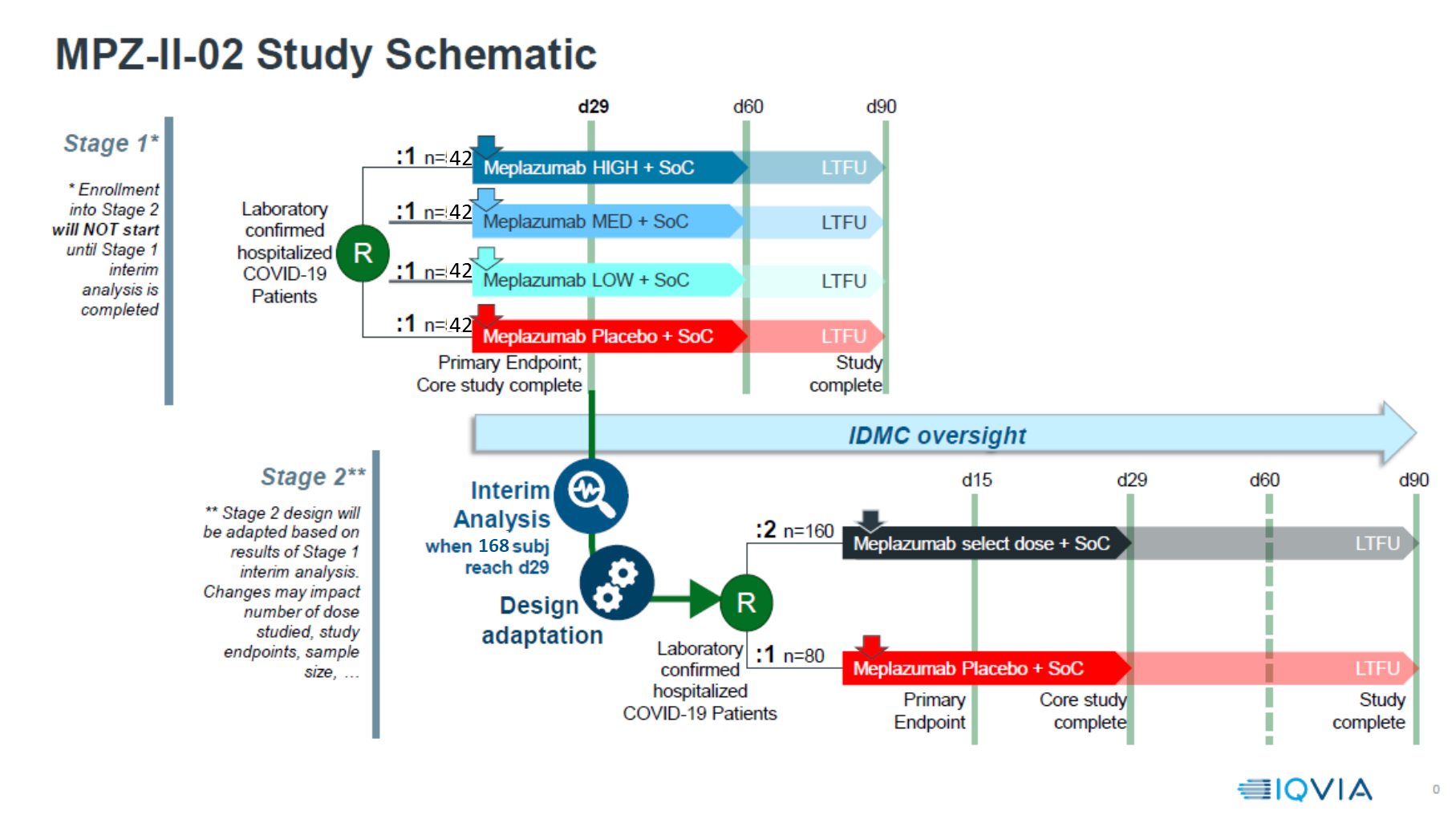
Abbreviations: d = day; IDMC = independent data monitoring committee; LTFU = long-term follow-up; n = number; SoC = standard of care; subj = subject.

## Schedule of Activities

**Schedule of Activities (Stage 1 and Stage 2)**

|  | ***Screen*** | ***Core Study*** | | | | | ***Follow-up^a^*** | ***End-of-study^a^*** |
| --- | --- | --- | --- | --- | --- | --- | --- | --- |
|  |  | ***Randomization*** |  | | | |  |  |
| **Day ± Window** | **Day -2 to 1**^c^ | **Day 1** | **Daily until hospital discharge** | **Day 8**  **(for non- hospitalized patients)** | **Day 15**^b^  **±3** | **Day 29**^b^  **±3** | **Day 57**  **±7** | **Day 84**  **±7** |
| **Time** |  |  |  |  |  |  |  |  |
| **Assessments/Procedures** |  |  |  |  |  |  |  |  |
| **ELIGIBILTY** |  |  |  |  |  |  |  |  |
| Informed consent | X |  |  |  |  |  |  |  |
| Demographics & Medical History ^d^  Disease | X |  |  |  |  |  |  |  |
| Date of Onset of Symptoms | X |  |  |  |  |  |  |  |
| Review SARS‑CoV‑2 results; confirm positive | X |  |  |  |  |  |  |  |
| Inclusion/exclusion Criteria | X |  |  |  |  |  |  |  |
| **STUDY INTERVENTION** |  |  |  |  |  |  |  |  |
| Randomization |  | X |  |  |  |  |  |  |
| Administration of meplazumab or control; weigh subject on each dosing day, before administration, to determine correct dose |  | X | X (Day 8) | X |  |  |  |  |
| Treatment with SoC until discharge according to treating physician’s discretion |  | X | | |  |  |  |  |
| **STUDY PROCEDURES** |  |  |  |  |  |  |  |  |
| Hospital admission & discharge date^e^ | X | X | X |  |  |  |  |  |
| COVID diagnosis date | X |  |  |  |  |  |  |  |
| Physical examination^f^ | X |  |  |  |  |  |  |  |
| Radiographic lung imaging (X-ray, CT scan)^g^ | X | X | X |  |  |  |  |  |
| Targeted physical examination^f^ |  |  | X | X |  |  |  |  |
| Vital signs including SpO_2_^h^ |  | X^j^ | X | X | X | X |  |  |
| Clinical and efficacy assessment scales^i^ |  | X^j^ | X |  | X | X |  |  |
| Targeted medication review |  | X^j^ | X |  | X | X | X | X |
| Adverse event evaluation | X | X | X | X | X | X | X | X |
| Infusion-related safety procedures |  | X | X^k^ | X |  |  |  |  |
| Disease-related co-infection complications |  | X | X |  |  | X |  |  |
| Survival (Death Yes/No) |  | X | X |  |  | X | X | X |
| Supplemental oxygen use days (low-flow nasal cannula, simple face mask) | X | X | X |  |  | X |  |  |
| High flow oxygen device use days (Venturi mask, high-flow nasal cannula) | X | X | X |  |  | X |  |  |
| Invasive mechanical ventilation use days |  | X | X |  |  | X |  |  |
| Non-invasive mechanical ventilation use days |  | X | X |  |  | X |  |  |
| **SAFETY LABORATORY** |  |  |  |  |  |  |  |  |
| Urinalysis | X |  |  |  |  |  |  |  |
| Safety hematology, chemistry and liver tests^l^ | X^m^ | X^j,n^ | Day 3, 5, 8, 11 (all ±1 day) if hospitalized |  |  | X |  |  |
| 12 lead ECG^o^ |  | X^j^ |  |  |  | X |  |  |
| Pregnancy test for females of childbearing potential | X^m^ |  |  |  |  |  |  |  |
| Hepatitis and HIV Virology screen (HIV, HBV, HCV) | X |  |  |  |  |  |  |  |
| **RESEARCH LABORATORY** |  |  |  |  |  |  |  |  |
| Blood for pharmacokinetic assessments^p^ |  | X | X^p^ | X^p^ |  | X | X |  |
| Blood for pharmacodynamic assessments^q^ |  | X | X^q^ | X^q^ |  | X |  |  |
| Antidrug-antibody assessment |  | X^j^ |  |  |  | X | X | X |
| Nasopharyngeal swab for viral load assessment |  | X^j^ | Day 3, 5, Day 8 (pre-dose) and Day 9 or Day 10 | X |  | X | X^r^ |  |

Abbreviations: CT = computer tomography; ECG = electrocardiogram; HBV = hepatitis B virus; HCV = hepatitis C virus; HIV = human immunodeficiency virus.

^a^ Visits that occur after discharge from the hospital may be conducted as a site visit, a telemedicine visit and/or home health nursing service, depending on individual study sites preferences and capabilities.

**^b^** If discharged prior to scheduled visit in person visits are preferred but recognizing quarantine and other factors may limit the subject’s ability to return to the clinic. In this case, these visits may be conducted as described above.

^c^ SARS‑CoV‑2 test results must be within 72 hours of Day 1 (randomization).

^d^ COVID‑19-specific history: shortness of breath, cough, fever, malaise, fatigue, myalgia; past medical history: chronic lung disease, including asthma, chronic renal disease, diabetes, heart disease, hypertension, autoimmune disease, women within 2 weeks postpartum and not breastfeeding, residents of long-term care facility, cancer, organ transplant

^e^ Dates to be captured include: date admitted to intensive care unit (ICU), date discharged from ICU, date readmitted to ICU, and date redischarged from ICU.

^f^ Physical examination (including weight, presenting symptoms; fever, respiratory symptoms, myalgias, malaise, etc). Subsequent visits will only include symptom directed (targeted) physical examination. Weight will be obtained on days when IMP is administered (eg, Day 8).

^g^ Radiographic lung image (X-ray, CT scan) will be recorded if performed/available at screen or Day 1, or during course of hospitalization.

^h^ Oral body temperature (Celsius scale with a thermometer), pulse rate (radial), respiratory rate, blood pressure (systolic and diastolic) and oxygen saturation (SpO_2_)

^i^ Clinical and efficacy data to be collected includes: measures from 6-point ordinal scale and NEWS2, as well as oxygen requirement, mechanical ventilator requirement, etc (see Section [8.1.1](#Appendix1)).

^j^ Baseline assessments should be performed prior to study treatment administration

^k^ Vital signs: including blood pressure (systolic and diastolic), pulse rate and saturation (finger oxygen SpO_2_), respiration and body temperature. Before each administration (within 30 min) and at 1 hour ±10 min after the end of administration during the treatment period, examinations should be performed every 6 hours thereafter until 24 hours after administration (Note: Examinations every 6 hours thereafter until 24 hours after administration is not required for non‑hospitalized patients).

^l^ Complete hematology, serum chemistry, lactate hydrogenase (LDH), cardiac troponin, D-dimer, ferritin, bicarbonate, haptoglobin outlined in [Appendix](#Appendix2) 12.3.

^m^ Laboratory tests performed in the 48 hours prior to enrollment will be accepted for determination of eligibility.

^n^ Any laboratory tests performed as part of routine clinical care within the specified visit window can be used for safety laboratory testing.

^o^ Recognizing that operational may limit the ability to record an ECG tracing, the procedure at a scheduled visit may be omitted and performed at the earliest time point when an ECG tracing can be performed.

^p^ Blood samples will be collected for pharmacokinetic assessments (when feasible) as follows while hospitalized or if subject returns for other blood collections after discharge: end of first infusion and between 2 and 4 hours relative to the end of the first infusion, within 20 to 52 hours following the infusion (on Day 2 or Day 3), prior to and at the end of second infusion (Day 8), within 20 to 52 hours following the second infusion (Day 9 or Day 10), Day 29 (at time of anti-drug antibodies [ADA] collection). This sampling schedule is specified for Stage 1. Stage 2 sampling times will be determined at the interim analysis based on emerging data. Testing will remain the same unless changes are warranted. For windows associated with the PK samples see Section 8.5.

^q^ Pharmacodynamic endpoints may include: CyPA, IL‑6, IL‑8, MCP‑1, MIP‑1α, MIP-1β, TNF‑α, IFN‑γ, IL‑1RA, IL‑2, IL-2Rα, IL-4, IL‑7, IL‑10, IL-15, IL‑17A, IL-12 p70, G‑CSF, M‑CSF, CXCL10, hsCRP. Collected when feasible at the following sampling times: predose, within 20 to 52 hours following the infusion (on Day 2 or Day 3), prior to second infusion (Day 8), within 20 to 52 hours following the second infusion (Day 9 or Day 10), Day 29 (optional). This sampling schedule is specified for Stage 1. Stage 2 sampling times will remain the same unless changes are warranted at the time of interim analysis. For windows associated with the PD samples see Section 8.6.

^r^ If viral RNA is still detected at Day 29, a follow-up sample for viral RNA testing at Day 57 will be collected.(Optional).

# Introduction

## Study Rationale

Meplazumab is a humanized anti-CD147 IgG2 monoclonal antibody which is expected to block the binding of the severe acute respiratory syndrome coronavirus 2 (SARS‑CoV‑2) spike protein to the human host-cell-expressed CD147, thereby blocking entry of SARS‑CoV‑2 into human tissue. This expectation is based on in vitro functional studies using Vero E6 cells infected with SARS-CoV-2 that demonstrated effective meplazumab mediated virus gene copy number inhibition upwards of 90% as evaluated by quantitative polymerase chain reaction. Meplazumab may also inhibit COVID-19 associated cytokine storm syndrome based on inhibition of the pro‑inflammatory factor CyPA host-cell CD147 interaction.

## Background

***Introduction of the disease***

Coronaviruses (CoV) are a large family of viruses that cause illness ranging from the common cold to more severe diseases such as Middle East respiratory syndrome (MERS-CoV) and severe acute respiratory syndrome (SARS-CoV). Coronavirus disease 2019 (COVID‑19) is caused by a new strain of the betacoronavirus that was discovered in 2019 and had not been previously identified in humans. The International Committee on Taxonomy of Viruses has proposed the virus be designated as SARS‑CoV‑2.

Most CoVs have a transmission history from animals to humans. The first reported cases of COVID‑19 were from Wuhan, a city in the Hubei Province of China, at the end of 2019. Investigation of the outbreak identified an initial association with a seafood market that sold live animals, where most subjects had worked or visited. This has subsequently progressed to a person-to-person transmission via droplets of respiratory secretions of an infected person^(1)^.

The incubation period for COVID‑19 is usually between 4 and 14 days from exposure. Common symptoms of COVID‑19 include respiratory symptoms, fever, cough, shortness of breath and breathing difficulties. In severe forms, infection can lead to pneumonia, severe acute respiratory syndrome, kidney failure and even death ^(2,3,4)^. Current management of COVID‑19 is supportive and acute respiratory distress syndrome (ARDS) is a major complication in subjects with severe disease. Respiratory failure from ARDS is the leading cause of mortality^(5)^.

Accumulating evidence suggests that a subgroup of subjects with severe COVID‑19 might have a cytokine release syndrome. Secondary hemophagocytic lymphohistiocytosis (sHLH) is a hyperinflammatory syndrome with fulminant and fatal hypercytokinemia resulting in multiorgan failure. Syndrome sHLH has been observed in 3.7% – 4.3% of sepsis cases commonly triggered by viral infections^(6,7)^; sHLH is characterized by unremitting fever, cytopenias, and hyperferritinemia with pulmonary involvement (including ARDS) in approximately 50% of subjects^(8)^.

Severe COVID‑19 presents a cytokine profile resembling sHLH. This includes increased interleukin (IL)-2, IL‑7, granulocyte-colony stimulating factor (G-CSF), interferon-γ inducible protein 10, monocyte chemoattractant protein 1, macrophage inflammatory protein 1-α, and tumor necrosis factor-α^(9)^. A study from Wuhan, China demonstrated high levels of ferritin and IL‑6 indicating that the fatality was a consequence of hyperinflammation^(5)^. Other studies have also revealed high levels of GM-CSF, along with a few other cytokines associated with severe complications in COVID‑19 subjects. High concentrations of GM-CSF were found in the plasma of severe and critically ill subjects requiring intensive care, that accounted for approximately 20%^(9,10)^.

Meplazumab for injection (hereafter referred to as meplazumab) is a humanized IgG_2_ monoclonal antibody that specifically binds CD147 molecule. The target for meplazumab is CD147 which has wide physiological and pathological significance and meplazumab was therefore explored as a possible treatment option for COVID‑19. CD147 is a highly glycosylated type I single transmembrane glycoprotein, belonging to the immunoglobulin (Ig) superfamily with its gene located on chromosome 19. It is expressed on the surface of red blood cells (RBCs) as well as on cells from multiple other tissue types. The mechanism of action of meplazumab involves binding to CD147 expressed on cells, to inhibit the interaction between CD147 and its ligand, SP expressed in viral envelope of SARS‑CoV‑2. This suppresses novel coronavirus invasion and replication amplification. Meplazumab also inhibits the interaction between CD147 and CyPA, and may inhibit the chemoattractant and inflammatory storm induced by SARS‑CoV‑2 infection as CyPA is one of the major pre-inflammatory cytokines which can recruit inflammatory cells and immune cells by release of cytokines, resulting in cytokine release syndrome. Information regarding nonclinical, pharmacokinetic, toxicology, and ongoing and completed clinical studies is available in the Investigators Brochure.

## Benefit/Risk Assessment

To date, meplazumab has previously been administered to 45 healthy volunteers in a single and multiple-dose tolerance trial (Study MPZ-I-01, N=42), or biodistribution study (Study MPZ‑I‑03; N=3), and 17 subjects with COVID‑19 disease, in the Study MPZ‑IIT‑01, a Phase 2 exploratory efficacy and safety, single and multiple dose tolerance study, in which no serious AEs (SAEs) were observed as defined by ICH Clinical Safety Data Management Definitions and Standards for Expedited Reporting. Abnormal laboratory examinations were observed in several subjects, including the abnormal hematology and biochemistry.

In Study MPZ‑IIT‑01, compared to control group, meplazumab treatment (10 mg infusions administered on 2 or 3 occasions over an approximately 1 week period) significantly improved the time-to-hospital discharge rate (p=0.005) in all subjects and case severity (p=0.021) in subjects in critical and severe condition. The time to virus negative in meplazumab group was reduced compared to that of the control group (median 3, 95% CI [1.5–4.5] versus 13, [6.5‑19.5]; p=0.045, HR=0.374, 95% CI [0.143–0.978]). The proportion of meplazumab-treated study subjects (%), with or without normal lymphocyte count and C-reactive protein concentration, was shown to be statistically significant compared to control-treated subjects from Day 7 through Day 29 (final study visit). No adverse effect was found in meplazumab-treated subjects.

However, due to limited clinical experience with meplazumab, all subjects will be closely monitored for any potential occurrence of SAEs, such as severe hypersensitivity or lung disorders, cardiac events and hepatic toxicity, as well as anemia suggestive of hemolysis. Because it may be medically difficult to discriminate between the progression of pre-existing lung disease (eg, asthma, chronic obstructive lung disease, lung fibrosis) and COVID‑19 lung disease, all worsening/progression of respiratory conditions in study subjects will be captured as AEs in the electronic case report form (eCRF). Furthermore, appropriate medical attention by the investigators and IQVIA’s medical monitors will be directed toward subjects thought to be at higher risk for these events and specific medical monitoring safety measures incorporated within the safety management plan.

The possibility of a severe allergic reaction, including anaphylaxis, which can manifest as life‑threatening bronchospasm or hypotension, should always be considered. A physician should be available on-site and supplies of epinephrine, antihistamine and corticosteroid readily accessible when a subject is being administered the study treatment.

For the duration of the hospitalization, all study subjects will receive SoC commensurate with critical care and hospitalization for COVID‑19, based on the medical judgment of the investigator. As such, antibiotics, antivirals, and corticosteroids will be accepted concomitant medications, as will investigational therapies that have been granted emergency use authorizations by regulatory health authorities. If the local SoC per written policies or guidelines (ie, not just an individual clinician decision) includes use of off-label medications, then use of these medications during the study is permitted. Otherwise, concomitant use of a therapy specifically for management of cytokine storm or for COVID‑19 infection is prohibited.

In view of the potential for interference with response to an investigational drug, restrictions on vaccine use are in place for clinical studies involving meplazumab. At present the effect of meplazumab on vaccine response is not known.

Live (live-attenuated) vaccines are not permitted within 2 weeks prior to randomization or during the study treatment and safety follow-up periods.

For this study, an independent safety and data monitoring committee (IDMC) will be established to provide safety oversight of study subjects. The IDMC will notify the Sponsor immediately should the safety of a subject(s) be at jeopardy due to participation in the study. The Sponsor will take all necessary safety precautions deemed necessary to protect the subject’s safety, including halting the study.

Based on the potential risks identified in association with meplazumab, a safety risk management plan/safety monitoring plan will be implemented to minimize risk to subjects participating in this study.

Given the nonclinical toxicology data and available safety information collected in 2 previous meplazumab clinical studies, and the drug’s mechanism of action, which is hypothesized to disrupt SARS‑CoV‑2 invasion into host cells as well as to dampen hyperimmune response triggered in severely ill COVID‑19 subjects, the benefit of meplazumab is thought to outweigh the risks of administration of the study treatment to subjects with COVID‑19.

More detailed information about the known and expected benefits and risks and reasonably expected AEs of meplazumab may be found in the Investigators Brochure.

# Objectives and Endpoints

|  | Objectives | Endpoints |
| --- | --- | --- |
| *No primary endpoint will be defined for Stage 1.* | - Stage 1: To evaluate the efficacy of 3 selected doses of meplazumab plus Standard of Care (SoC) compared to control plus SoC in subjects hospitalized with COVID‑19 infection. | - Dose selection endpoint: Determine an optimal dose based on response rate at Day 29 by sustained clinical improvement of 2 points (from randomization) on 6-point ordinal scale (see scale definition in Section [8.1.2](#Appendix1)) |
| Primary  *Primary endpoint will be determined based on adaptation during the interim analysis of Stage 1 data.* | - Stage 2: To evaluate the efficacy of the selected dose of meplazumab plus SoC (based on adaptation from Stage 1) compared to control plus SoC, in subjects hospitalized with COVID‑19 infection. | *Primary endpoint will be determined at interim analysis.*   - Time to sustained clinical improvement (days; Time frame: Day 1 through Day 29) of at least 2 points (from randomization) on a 6‑point ordinal scale, (where sustained improvement is improvement without subsequent worsening),or live discharge from the hospital, whichever comes first - Response rate, as defined by a sustained improvement of 2 points on a 6-point ordinal scale, at Day 29 - Mortality at Day 29 - Proportion of subjects alive and discharged without supplemental oxygen at Day 29 |
| **Secondary** | - To evaluate response rate | - Response rate (number and %) by treatment arm at Day 2, 8, and 15, as defined by a sustained improvement of 2 points on a 6-point ordinal scale |
|  | - To evaluate live discharge | - Proportion of subjects alive and discharge without supplemental oxygen at Day 15 and Day 57 |
|  | - To evaluate the safety of meplazumab as add-on therapy to SoC in subjects with COVID‑19 | - Physical examination - Clinical laboratory examinations - Vital signs (BP/HR/temperature/respiratory rate/ saturation [finger oxygen SpO_2_]) - 12-lead electrocardiogram (ECGs) - Virologic load (quantitative polymerase chain reaction [PCR] for COVID‑19 in NP swab) on Day 1(predose), 3, 5, 8 (pre dose) and 9 or 10, 29 and 57 (optional) - Antidrug antibody (ADA) titers (predose, end-of-treatment [Day 29], follow-up visit [Day 57] and end-of-study visit day) - Adverse events of special interest: disease-related secondary infection complications, hemolysis, Grade 4 (CTCAE V5) neutropenia and lymphopenia, and anaphylactic reactions defined by Clinical Criteria for Diagnosing Anaphylaxis^(11)^; 20% decline in SpO_2_ between start and end of 1-hr study treatment infusion; ALT or AST >3 x ULN AND TBL >2 x ULN; Evidence of red blood cell (RBC) hemolysis as defined by 2 of the following 3 findings:   - Anemia that is not due to another obvious cause;   - Increased reticulocyte count that is not explained by an obvious cause;   - Signs of RBC destruction, such as increased lactate dehydrogenase (LDH), low haptoglobin ≤25 mg/dL, increased unconjugated bilirubin. |
|  | - To evaluate overall mortality | - Mortality at Days 15 and 57 - Time from treatment start date to death. |
|  | - To evaluate clinical recovery of COVID‑19 disease | - Time to sustained recovery (days; Time frame: Day 1 through Day 29) as defined by first day on which 1 of the following 2 categories is achieved using the 6-point ordinal scale.   1. Not hospitalized   2. Hospitalized, not requiring supplemental oxygen |
|  | - To evaluate the number of oxygen-free days | - Duration (days) of oxygen use and oxygen-free days |
|  | - To evaluate ventilator-free days, incidence, and duration of new mechanical ventilation use | - Duration (days) of mechanical ventilation and mechanical ventilation-free days - Incidence of new mechanical ventilation use and duration (days) of new mechanical ventilation use |
|  | - To evaluate clinical relapse of COVID‑19 | - Incidence of new mechanical ventilation use after mechanical ventilation extubation period of 24 hours - Incidence of rehospitalization following hospital discharge |
|  | - To evaluate ICU and hospitalization length | - Duration (days) of ICU and hospitalization |
| **Exploratory** | - To evaluate pharmacokinetic (PK) exposure to meplazumab - To evaluate pharmacodynamic (PD) response to administration of meplazumab - To explore potential exposure-response relationships - To evaluate the efficacy of the selected dose using a composite ranked outcome trajectory score | - Meplazumab serum and blood cell concentrations and derived PK parameters: if calculable by noncompartmental analysis, PK parameters may include but are not limited to maximum observed concentration (C_max_), time to C_max_ (t_max_), area under the concentration-time curve (AUC) calculated to the last quantifiable concentration and to infinity [AUC_(0‑last)_, AUC_(0‑inf)_], elimination half-life (t½), volume of distribution (Vz, Vss), and systemic clearance (CL). - PD endpoints: Changes from baseline in cytokine, and chemokines related to inflammatory and immune status including but not limited to CyPA, IL‑6, IL‑8, MCP‑1, MIP‑1α, MIP-1β, TNF‑α, IFN‑γ, IL‑1 ra, IL‑2, IL-2Rα, IL-4, IL‑7, IL‑10, IL-15, IL‑17A, IL-12 p70, G‑CSF, M‑CSF, CXCL10, hsCRP. - Graphical (and statistical, if appropriate) display of exposure versus response for select endpoints. - Ranked outcome trajectory by Day 29 using the ordinal scale at Days 8, 15, and 29   For each subject, the below items are calculated and sorted,   1. The worst score over the 28 days; 2. The last recorded score; 3. The duration at the worst score; 4. The best score that occurs after the worst score; 5. The duration of (4) is observed.  - Rank by ascending order of 1, 2, 3, 4 then descending order of 5. |
| Abbreviations: ALT = alanine aminotransferase; AST = aspartate aminotransferase; BP = blood pressure; COVID‑19 = coronavirus disease 2019; CXCL10 = C–X–C motif chemokine 10; CyPA = cyclophilin A; G‑CSF = granulocyte colony‑stimulating factor; HR = heart rate; hsCRP = high‑sensitivity C‑reactive protein; ICU = intensive care unit; IFN‑γ = interferon gamma; IL‑1 RA = interleukin‑1 Receptor Antagonist; IL‑2 = interleukin‑2; IL-2 Rα (Interleukin-2 Receptor alpha); IL‑4 = interleukin‑4; IL‑6 = interleukin‑6; IL‑7 = interleukin‑7; IL‑8 = interleukin‑8; IL‑10 = interleukin‑10; IL‑15 = interleukin‑15; IL‑17A = interleukin‑17A; IL-12p70 = interleukin12p70; MCP‑1 = monocyte chemoattractant protein‑1; M‑CSF = macrophage colony‑stimulating factor; MIP‑1α = macrophage inflammatory protein‑1 alpha; MIP‑1β = macrophage inflammatory protein-1 beta; NP = nasopharyngeal; SpO_2_ = peripheral capillary oxygen saturation; TBL = total bilirubin; TNF‑α = tumor necrosis factor alpha; ULN = upper limit of normal. | | |

Some of the exploratory endpoints may be reported separately from the clinical study report (CSR).

# Study Design

## Overall Design

This is a multicenter, seamless, randomized, third-party-blind study to evaluate the safety and efficacy of meplazumab for the treatment of COVID‑19 in hospitalized adults (≥18 years). Neither the subject nor the investigator shall be aware of whether the subject is taking study drug or placebo, as the study drug/placebo is prepared by an unblinded third party (eg, a pharmacist or nurse) and administered by an authorized blinded site staff.

Enrollment of subjects will be stopped once the total number of planned subjects have completed the Stage 1 Day 29 visit procedures. Once the interim analysis of Stage 1 study data is complete and the IDMC has recommended the meplazumab dose that is safe and effective to carry forward into Stage 2, the study will resume subject enrollment. A summary of the key Stage 1 interim analysis results will be sent to the relevant Health Authorities involved, if requested.

For the duration of the hospitalization, all study subjects will receive SoC commensurate with critical care and hospitalization for COVID‑19, based on the medical judgment of the study investigator.

Stage 1, the dose-finding period of the study, will compare 3 meplazumab treatment arms to control, in addition to SoC, to determine the safe and efficacious dose to investigate in Stage 2, the confirmatory period of the study. Response rate (number and %) by treatment arm at Stage 1 on Day 29 will be used to select the dose to implement in Stage 2 of the study. Stage 1 data will additionally be used to determine optimal study endpoints, and the number of subjects to enroll into Stage 2 of the study. Stage 2 of the study is intended to provide a robust database on the selected dose to fully evaluate disease outcomes, including severe AEs, overall AEs, disease-related co-infection complications (eg, pneumonia, septic shock) and overall mortality.

Hospitalized subjects with laboratory-confirmed SARS‑CoV‑2 infection, with clinical status as defined as a Grade 3 (hospitalized, requiring supplemental oxygen) or Grade 4 (hospitalized, on non-invasive ventilation or high flow oxygen devices) on the 6-point ordinal scale (Section 8.1.2) will be enrolled for both Stage 1 and Stage 2. Randomization will be stratified by severity, and by age group (age <65 years or ≥65 years). Additional stratification factors such as region and/or receipt status of remdesivir at baseline may be considered for Stage 2 based on the data evaluated in Stage 1. Randomization sequence will be created separately for Stage 1 and Stage 2 with permuted block randomization.

For Stage 1, Approximately 168 subjects will be randomized and allocated 1:1:1:1 to receive either low dose, medium dose, or high dose of meplazumab, or control.

For Stage 2, 240 additional subjects will be randomized and allocated 2:1 to receive the optimal meplazumab dose or control.

In both stages, subjects will receive intravenous (IV) infusion of meplazumab or control as specified.

## Scientific Rationale for Study Design

Given that the SoC for COVID‑19 is rapidly evolving, the mainstay of treatment is largely supportive and based on the subject’s treating physician’s medical judgment. At present, few positive study results for COVID‑19 have been reported; however, based on early analysis of data from the [Adaptive COVID‑19 Treatment Trial](https://www.niaid.nih.gov/news-events/nih-clinical-trial-remdesivir-treat-covid-19-begins) (ACTT), remdesivir was granted Emergency Use Authorization by the Food and Drug Administration (FDA) for the treatment of COVID‑19 in subjects with suspected or laboratory-confirmed SARS‑CoV‑2 infection and severe COVID‑19 disease. The reported results showed that the remdesivir helped subjects with “moderate” disease recover more quickly when they received it for 5 days.

The UK RECOVERY trial assessed the mortality rate at Day 28 in hospitalized patients with COVID-19 who received low-dose dexamethasone 6 mg orally or IV daily for 10 days added to usual care. In the dexamethasone group, the incidence of death was lower than in the usual care group among patients receiving invasive mechanical ventilation (29.3% versus 41.4%) and among those receiving oxygen without invasive mechanical ventilation (23.3% versus 26.2%). These results from the RECOVERY trial, as well as several other similar studies, have led treating clinicians to consider when low-dose corticosteroids may be beneficial treatment for hospitalized patients with COVID-19.

After the SARS-CoV was identified in 2002 and caused a large global outbreak, there was an increased interest in the development of specific therapeutic agents. SARS-CoV case subjects were treated with corticosteroids, type 1 interferon (IFN) agents, convalescent plasma, ribavirin, and lopinavir or ritonavir, and, except for ribavirin, many of these agents have in vitro preclinical data that support their efficacy. Since the SARS outbreak, new therapeutic agents targeting viral entry proteins, proteases, polymerases, and methyltransferases have been tested, however, none of them has been shown to be efficacious in clinical trials.

This study utilizes an adaptive design that maximizes efficiency in identifying a safe and efficacious therapeutic agent for COVID‑19 during the current outbreak. As the study will be a multicenter, multinational randomized third-party-blind study, it will be possible to acquire rigorous data about the safety and efficacy of meplazumab for COVID‑19 in addition to well‑documented information about COVID‑19 disease in an important subpopulation that develop respiratory disease prior to the need for mechanical ventilation.

Randomization and the inclusion of a control arm are essential to control unintended introduction of bias into the study results necessary to establish the safety and efficacy of meplazumab in COVID‑19. The use of a 2:1, drug:control randomization ratio at Stage 2 of the study optimizes access to a potentially effective treatment while not significantly compromising control or statistical power. The collection of clinical and virologic data on enrolled subjects using a standardized timeline and collection instruments should provide valuable information about the clinical course of and morbidities associated with severe COVID‑19 in a diverse group of hospitalized adult subjects.

The study is third party-blinded for all investigational staff who make determinations related to the study. The third party-blinded design is important, to make objective assessments of the pharmacological effect of the study treatment and to minimize the effect of assessment bias.

## Justification for Dose

The proposed doses for Stage 1 of the planned Phase 2/3 study are 0.12 mg/kg (administered once, on Day 1), 0.4 mg/kg (administered as 0.2 mg/kg on Day 1 and Day 8) and 0.6 mg/kg (administered as 0.3 mg/kg on Day 1 and Day 8). The dose selection and dosing frequency for this Phase 2/3 clinical trial are based on the safety, pharmacokinetic (PK), and receptor occupancy (RO) results of a Phase 1 single and multiple-dose safety/tolerance study (Study MPZ-I-01), a Phase 1 ^131^I‑meplazumab biodistribution study (Study MPZ-I-03), an open label pilot Phase 2 investigator-initiated trial (Study MPZ-IIT-01) and supporting nonclinical data.

In Study MPZ-I-01, which tested single IV doses of 0.06, 0.12, 0.2, 0.3, 0.42, and 0.56 mg/kg, or the 0.3 mg/kg repeat-dose administered on 2 occasions, 7 days apart (Section 2.3), no deaths, serious TEAEs, TEAEs of Grade ≥ 3, TEAEs leading to dose adjustment or temporary discontinuation, or TEAEs leading to permanent discontinuation of the study were reported. Meplazumab concentrations in serum were low and rapidly decreased below the assay limit of quantitation (0.15 ng/mL). This suggests that free circulating meplazumab concentrations are rapidly depleted from the serum via binding to CD147 on blood cells and target tissues. Sustained meplazumab exposure and RO on human peripheral blood cells were observed in this study with half-life well exceeding 20 days. Meplazumab bound to peripheral blood cells was quantifiable through the Day 56 cutoff for Study MPZ-I-01, with residual RO ranging from approximately 4% to 16.5%. The End-of-Study Day 84 PK and RO results are not yet available. Dose-limiting exposure was observed with increasing meplazumab dose. The dose-limiting blood-cell PK is thought to be due to saturation of CD147 binding on the blood cells. The highest mean blood-cell bound meplazumab concentrations were observed in the 0.42 mg/kg dose group with a mean maximum concentration of 4.72 µg/mL. At this dose, meplazumab concentrations bound to blood cells were approximately 100-fold higher than the maximum concentration observed in serum.

Supporting nonclinical data indicate that meplazumab binding to blood cells is reversible (see Invesitgator’s Brochure for more information). Meplazumab dissociation from RBCs (into supernatant) was demonstrated over a 120 hour incubation period. An equilibrium between cell-bound and free (in supernatant) meplazumab was observed within 24 to 48 hours under the assay conditions. This study also demonstrated that released meplazumab is able to subsequently bind to other CD147 carrying tissues; human normal lung epithelial cells (BEAS-2B) were utilized in this study. Binding of meplazumab (contained in the supernatant) to BEAS-2B appeared to follow the time-course of meplazumab release. It is hypothesized that circulating blood cells serve as reservoirs for meplazumab and continuously distribute/release meplazumab to target tissues with CD147 expression, such as the lung. This hypothesis is supported by the results of the biodistribution Study MPZ-I-03 in which IV-administered ^131^I‑meplazumab rapidly distributed throughout the body to the evaluated tissues. Tissue uptake of ^131^I‑meplazumab was calculated as tissue to cardiac pool (T/C) radiation ratio. In the lung, one of the key target organs of interest for COVID-19, the T/C radiation ratio of lung relative to cardiac blood-pool ranged from 0.41 to 0.32 over the 1 to 336 post-administration evaluation period. These results indicate that meplazumab can reach its intended targets (such as lung tissue) for COVID-19 treatment and target binding is sustained for at least 14 days.

Meplazumab was found to block interaction between CD147 and its ligands, SP and CyPA, in an ELISA-based competitive binding assay with half-maximal inhibitory concentration (IC_50_) values of 16.44 and 1.28 µg/mL, respectively. In vitro functional studies using Vero E6 cells infected with SARS-CoV-2 demonstrated effective meplazumab mediated virus gene copy number inhibition as evaluated by quantitative PCR. The extent of inhibition was concentration dependent with an IC_50_ of 17.58 μg/mL. A concentration-dependent RO rate was determined in Vero E6 to establish a correlation between meplazumab RO on the CD147 receptors in Vero E6 cell. Receptor occupancy percentage on intact Vero E6 cells at 17.58 μg/mL was modeled to be 47.19% while meplazumab concentrations resulting in approximately 90% viral inhibition in infected Vero E6 cells were calculated to yield a RO of approximately 88%. The RO at 200 μg/mL (the highest tested concentration in Study MPZ‑CPE‑2020‑02) was calculated as 88.92%.

The observed RO in blood-cells and corresponding blood-cell PK results observed in the Phase 1 clinical study (Study No. MPZ-I-01) may be correlated to the RO-response results obtained in Vero E6 cells under the assumption that RO in human peripheral blood cells is directly correlated with meplazumab binding to and occupancy on CD147 on target tissues. In healthy volunteers the mean maximum RO% on peripheral blood cells were 15.89%, 30.65%, 59.33%, 83.92%, 97.61%, and 111.00% after single dose administration of 0.06, 0.12, 0.2, 0.3, 0.42 and 0.56 mg/kg, respectively. The maximum RO% after repeat dose (0.3 mg/kg) administration were 85.30% (first dose) and 99.12% (second dose), respectively. Based on the above information:

- A single 0.12 mg/kg meplazumab dose has been selected as minimum effective dose. The maximum RO associated with the dose (0.12 mg/kg) in Study No. MPZ-I-01 was 30.65%. This dose is expected to have low risk of both on-target and off-target related AEs. In exploratory clinical Study MPZ‑IIT‑01, meplazumab was administered as 10 mg doses on up to 3 occasions, (Days 1 and 2, and also on Day 5 in some subjects). The dosage of meplazumab for the subjects was 10 mg and the body weights were 45 to 81 kg, which was about 0.12 to 0.22 mg/kg for each dose. This dose was well tolerated in COVID-19 patients and demonstrated a promising clinical response compared to controls in Study MPZ-IIT-01. The planned dose of 0.12 mg/kg is expected to result in an initial clinical response in COVID‑19 patients.
- A 0.4 mg/kg meplazumab dose, administered as 2 doses of 0.2 mg/kg 7 days apart, has been selected as the midrange dose. Given the observed nonlinear meplazumab PK in blood cells, the divided dose is expected to provide a superior extended meplazumab exposure compared to a single 0.4 m/kg dose. Re-administration after 7 days will boost both blood-cell and tissue-bound meplazumab concentrations. This dose is the lowest single IV meplazumab dose that reaches a maximum RO of 50%. The 50% RO number is based on the consideration that it is mechanistically expected one would need 50% RO to minimally reduce viral entry into human tissue. When evaluating the RO% versus time profiles, mean RO% in the 0.2 mg/kg group remained above 50% for 7 days postdose but fell below 50% within 10 days following dose administration (43.33%). Within 2 weeks, mean RO% had decreased to 38.23%. The additional exposure to meplazumab in the second week from onset of illness is the critical window to control the progress of COVID-19 disease. In the exploratory clinical Study MPZ‑IIT‑01, meplazumab was administered as 10 mg doses on up to 3 occasions, (Days 1 and 2, and also on Day 5 in some subjects). This dose regimen, which translates into an approximately 0.3 to 0.4 mg/kg total dose, was well tolerated in COVID-19 patients and demonstrated a promising clinical response compared to controls in Study MPZ‑IIT‑01.
- A 0.6 mg/kg meplazumab dose, administered as 2 doses of 0.3 mg/kg 7 days apart, has been selected as the high dose. Distribution of ^131^I-meplazumab associated radioactivity to intended targets (such as lung tissue) was demonstrated following a single 0.3 mg/kg IV administration in Study MPZ-I-03. In healthy subjects, mean RO% on peripheral blood cells ranged from greater than 80% on Day 1 through greater than 60% for the first 7 days after the first 0.3 mg/kg meplazumab dose. Following re-administration of the 0.3 mg/kg dose after 7 days, mean RO% ranged from approximately 99% to 74% over the subsequent 7-day sampling period Study MPZ-I-01. The second dose of meplazumab is expected to boost both blood-cell and tissue-bound meplazumab concentrations for continued high RO through at least 2 weeks following treatment imitation.

The selected doses of 0.12 mg/kg single dose, 0.4 and 0.6 mg/kg (administered as 2 injections on Days 1 and 8) for repeat-dose are 1/33.33, 1/10 and 1/6.67 times the no-observed-adverse-effect‑level (NOAEL) in rhesus monkeys (4 mg/kg/week), respectively. Although a 10-fold lower binding affinity of meplazumab for monkey CD147 was observed in vitro compared to human CD147, the safety of the proposed doses is supported by the results of the single and multiple-dose safety and tolerance study (Study MPZ-I-01) in healthy volunteers where no SAEs were reported at single doses up to 0.56 mg/kg and multiple doses of 0.3 mg/kg.

## End of Study Definition

A subject is considered to have completed the study if he/she has completed all phases of the study including the last visit or the last scheduled procedure shown in the Schedule of Activities (SoA) (Section 1.3).

The end of the study is defined as the date of the last visit of the last subject in the study or last scheduled procedure shown in the SoA (Section 1.3) for the last subject in the study globally.

# Study Population

Prospective approval of protocol deviations to recruitment and enrollment criteria, also known as protocol waivers or exemptions, is not permitted.

## Inclusion Criteria

Subjects are eligible to be included in the study only if all of the following criteria apply:

1. Adults (≥18 years) with laboratory-confirmed SARS‑CoV‑2 infection as determined by PCR or other commercial or public health assay, which is FDA cleared or have use authorization in country that subject resides in (test results must be obtained within 72 hours of Day 1).
2. A score of Grade 3 (hospitalized, requiring supplemental oxygen) or Grade 4 (hospitalized, on non-invasive ventilation or high flow oxygen devices) on the 6-point ordinal scale.
3. Willingness and ability to comply with study-related procedures and assessments.
4. Ability to provide informed consent signed by study subject or legally authorized representative.
5. Male and/or female
   1. Male subjects:

- A male subject must agree to use contraception as detailed in [Appendix](#Appendix6) [12.4](#Appendix124) of this protocol during the treatment period and for at least 6 months, corresponding to time needed to eliminate study treatment for both genotoxic and teratogenic study treatments, after the last dose of study treatment.
  1. Female subjects:
- A female subject is eligible to participate if she is not pregnant (see [Appendix](#Appendix6) 12.4), not planning to get pregnant in the next 6 months, not breastfeeding, and at least 1 of the following conditions applies:
  - 1. Not a woman of childbearing potential (WOCBP) as defined in [Appendix](#Appendix6) 12.4.

OR

- - 1. A WOCBP who agrees to follow the contraceptive guidance in [Appendix](#Appendix6) 12.4 during the treatment period and for at least 130 days, (5 terminal half‑lives and, for genotoxic products, an additional 30 days, corresponding to time needed to eliminate study treatment plus 30 days for study treatments with genotoxic potential) after the last dose of study treatment.

## Exclusion Criteria

Subjects are excluded from the study if any of the following criteria apply:

1. Any physical examination findings, laboratory abnormality, and/or history of any illness, that in the study Investigator’s judgment, could jeopardize the safety of the subject by their participation in the study.
2. Subject with evidence of critical COVID-19 illness, defined by at least 1 of the following: requiring invasive mechanical ventilation or extracorporeal membrane oxygenation (ECMO); shock (defined by systolic blood pressure <90 mmHg, or diastolic blood pressure <60 mmHg, or requiring vasopressors); or multi-organ dysfunction/failure.
3. Stage 4 severe chronic kidney disease or requiring dialysis (ie, estimated glomerular filtration rate [eGFR] <30 mL/min/1.73 m^2^).
4. Pregnant or breast feeding.
5. Anticipated transfer to another hospital which is not a study site within 72 hours.
6. Allergy to any study medication.
7. Use of anticancer, antitransplant rejection, or immunomodulatory biological drug (within 30 days of enrollment or 5 times the half-life [whichever is longer]).
8. Chronic glucocorticosteroid use equivalent to daily oral prednisone >10 mg per day for more than 3 months (10 mg oral prednisone every other day is allowed).
9. Live (live-attenuated) vaccines are not permitted within 2 weeks prior to randomization or during the study treatment and safety follow-up periods.
10. Subjects participating in another clinical study. There will be a need for washout with 5 half‑lives depending on the study treatment or 30 days since any previous study, whichever is longer.
11. Total bilirubin (TBL) >2 × upper limit of normal (ULN), or alanine aminotransferase (ALT) >5 × ULN, or aspartate aminotransferase (AST) >5 × ULN, or alkaline phosphatase >5 × ULN.
12. Platelet <50×10^9^/L, or hemoglobin <60 g/L.
13. Glomerular filtration rate <30 mL/min/1.73 m^2^, or serum creatinine increased by 0.5 mg/dL within 7 days, or oliguria (<400 mL/24 hour), or anuria (<100 mL/24 hour).

## Screen Failures

Screen failures are defined as subjects who consent to participate in the clinical study but are not subsequently randomly assigned to study intervention.

After the screening evaluations have been completed, the investigator or designee will review the inclusion/exclusion criteria and determine the subject’s eligibility for the study.

Only the reason for ineligibility will be collected on screen failures. Subjects who are found to be ineligible will be told the reason for ineligibility.

# Study Treatment

Study treatment is defined as any study treatment(s), marketed product(s), placebo, control, or medical device(s) intended to be administered to a subject according to the study protocol.

## Study Treatment(s) Administered

Meplazumab will be provided in the form of a 10-mg/vial freeze-dried powder containing 1.60 mg histidine, 3.08 mg histidine hydrochloride, 50.0 mg sucrose, 70.0 mg mannitol, and 1.0 mg polysorbate 80.

Matching control will be sterile normal saline (0.9%) for IV infusion.

Meplazumab will be provided by the Sponsor. Control will be provided by the clinical study site (Table 6).

Table 6 Study Treatment Details

| Study Treatment Name: | Meplazumab Low Dose | Meplazumab Medium Dose | Meplazumab High Dose | Control |
| --- | --- | --- | --- | --- |
| Dosage Formulation: | Solution for IV infusion | Solution for IV infusion | Solution for IV infusion | Sterile normal saline (0.9%) for IV infusion |
| Unit Dose Strength(s)/Dosage Level(s): | First dose: 0.12 mg/kg – Day 1  second dose: control – Day 8* | First dose: 0.2 mg/kg – Day 1  second dose: 0.2 mg/kg – Day 8 | First dose: 0.3 mg/kg – Day 1  second dose: 0.3 mg/kg – Day 8 | First dose: control – Day 1  second dose: control – Day 8 |
| Route of Administration | IV | IV | IV | IV |
| Dosing Instructions: | 1-hour infusion, 100 mL | 1-hour infusion, 100 mL | 1-hour infusion, 100 mL | 1-hour infusion, 100 mL |
| Packaging and Labeling | Study treatment will be provided in kits that each contain 1, 10 mg vial of meplazumab. Kits and vials will be labeled per country requirement. | Study treatment will be provided in kits that each contain 1, 10 mg vial of meplazumab. Kits and vials will be labeled per country requirement. | Study treatment will be provided in kits that each contain 1, 10 mg vial of meplazumab. Kits and vials will be labeled per country requirement. | Study treatment will be provided by site in standard local container, labeled as required per country requirement. |
| Manufacturer | Jiangsu Pacific Meinuoke Biopharmaceutical Co., Ltd. | Jiangsu Pacific Meinuoke Biopharmaceutical Co., Ltd. | Jiangsu Pacific Meinuoke Biopharmaceutical Co., Ltd. | As detailed in the study‑specific Pharmacy Manual |

Abbreviations: IV = intravenous; kg= kilogram; mg = milligram; mL = milliliter.
* Sterile normal saline (0.9%) for IV infusion

## Preparation/Handling/Storage/Accountability

1. The investigator or designee must confirm appropriate temperature conditions have been maintained during transit for all study treatment received and any discrepancies are reported and resolved before use of the study treatment.
2. Only subjects enrolled in the study may receive study treatment and only authorized study center staff may supply or administer study treatment. All study treatments must be stored in a secure, environmentally controlled, and monitored (manual or automated) area in accordance with the labeled storage conditions with access limited to the investigator and authorized study center staff.
3. The investigator, institution, or the head of the medical institution (where applicable) is responsible for study treatment accountability, reconciliation, and record maintenance (ie, receipt, reconciliation, and final disposition records).
4. Further guidance and information for the final disposition of unused study treatment are provided in the Study Reference Manual.

The investigator, a member of the study center staff, or a hospital pharmacist must maintain an adequate record of the receipt and distribution of all study medication using the Drug Accountability Form. These forms must be available for inspection at any time.

## Measures to Minimize Bias: Randomization and Blinding

Study treatment will be provided in a blinded fashion and dispensed in accordance with randomization code. Randomization will be performed by interactive web response system (IWRS). Before the study is initiated, the log-in information and directions for the IWRS will be provided to each study site. Study treatment will be dispensed at the study visits as summarized in the SoA (Section 1.3).

The investigator or investigator’s designee will access the IWRS at Screening to obtain the subject study number. The investigator or the investigator’s designee will utilize the IWRS to randomize the subject into the study. During this contact, the investigator or designee will provide the necessary subject-identifying information, including the subject number assigned at screening. The MED ID number of the study medication to be dispensed will then be provided by the IWRS. If the MED ID number is lost or damaged, the site can request a replacement from the IWRS. (Refer to the IWRS manual provided separately). The MED ID number will be entered onto the eCRF. At all drug-dispensing visits, the investigator or designee will again contact the IWRS to request additional investigational drug for a subject.

### Randomization Code Creation and Storage

The randomization schedule will be created by the Sponsor or designee and stored in a secure area, accessible only to authorized personnel. Block sizes will be specified in the randomization specifications.

### Investigational Drug Blind Maintenance

The study treatment will be provided with an unblinded label, randomization will be unblinded and dispensing and preparation of the solution for IV infusion will be completed by an unblinded pharmacist. Detailed instructions are laid out in the Pharmacy Manual.

The study treatment blind will be maintained using the IWRS, which can be accessed and broken by the investigator or designee in an emergency for unblinding of study medication assignments to ensure the safety of the subject.

In order to maintain this blind, the randomization schedule is to be kept strictly confidential, accessible only to authorized persons (eg, randomization statistician, pharmacists, and the bioanalytical laboratories that prepare and analyze relevant samples), until the time of unblinding at Stage 1 and Stage 2.

### Unblinding Procedure

The study treatment blind shall not be broken by the investigator unless information concerning the study treatment is necessary for the medical treatment of the subject. In the event of a medical emergency, if possible (but not required), the Medical Monitor should be contacted before the study treatment blind is broken to discuss the need for unblinding and if unblinding is required.

For unblinding a subject, the study treatment blind can be obtained and broken by the investigator at any time, by accessing the IWRS.

The Sponsor must be notified as soon as possible if the study treatment blind is broken. The date, time and reason for the blind being broken must be recorded in the eCRF.

If any site personnel are unblinded to a subject’s treatment, the subject must be discontinued from the study treatment. If any amount of study intervention was administered, follow procedures according to the SoA. The investigator or other site personnel should not reveal the specific treatment assignment of the unblinded subject to the Medical Monitor or other contract research organization (CRO) or Sponsor staff. The subject should continue to the End-of-Study/Early Termination (ET) Visit.

## Study Treatment Compliance

Study treatment supplies will be counted and reconciled at the site before being returned to the Sponsor or designee, or before being destroyed at the site (if approved by the Sponsor for destruction at the site).

The investigator or designee must ensure that the study treatment is used in accordance with the approved protocol and is dispensed only to subjects enrolled in the study. To document appropriate use of the study treatment, the investigator must maintain records of all Sponsor-supplied drug delivery to the site, site inventory, dispensation and use by each subject, and return to the Sponsor or designee.

Upon receipt of the study treatment, the investigator or designee must verify the contents of the shipments against the packing list. The verifier should ensure that the quantity is correct, and that the study treatment is received within the labeled storage conditions in good condition. If there are any discrepancies between the packing list versus the actual product received, the Sponsor must be contacted to resolve the issue. The packing list should be filed in the investigator’s essential document file.

The investigator must maintain 100% accountability for all study treatment received and dispensed during his or her entire participation in the study. Proper drug accountability includes, but is not limited to:

- Frequently verifying that actual inventory matches IWRS inventory.
- Verifying that the IWRS is completed for the MED ID used to prepare each dose.
- Verifying that all containers used are documented accurately in the IWRS.
- Verifying that required IWRS fields are completed accurately.

If any dispensing errors or discrepancies are discovered, the Sponsor must be notified immediately.

The current inventory of all study treatment at sites will be tracked through the IWRS.

The IWRS will include all required information as a separate entry for each subject to whom study treatment is dispensed.

The investigator will be notified of any expiration date extension for study treatment during the study conduct. On expiration date notification from the Sponsor or designee, the site must complete all instructions outlined in the notification including segregation of expired study treatment for return to the Sponsor or its designee for destruction.

In the event of expiration date extension of supplies already at the study site, supplies may be relabeled with the new expiration date. In such cases, the Sponsor or its designee will prepare additional labels and all necessary documentation for completion of the procedure.

## Concomitant Therapy

Concomitant medications in a hospitalized population change daily and are difficult to collect and attribute to success and failure therapy and impact on safety. Concomitant medications will be recorded in this study. Any medication, killed vaccine, or COVID-19 vaccine that the subject is receiving at the time of enrollment (within 7 days before the time of enrollment) or receives during the study must be recorded on the eCRF along with:

- Reason for use
- Dates of administration including start and end dates
- Dosage information including dose and frequency

The Medical Monitor should be contacted if there are any questions regarding concomitant or prior therapy.

**Excluded Medications**

- Anticancer, antitransplant rejection or immunomodulatory biological drug within 30 days or 5 times the half-life (whichever is longer) prior to enrollment.
- Chronic glucocorticosteroid use equivalent to daily oral prednisone >10 mg per day (10 mg oral prednisone every other day is allowed) within 30 days of enrollment.
- Immunization with a live or live-attenuated vaccine within 2 weeks prior to randomization and at any time during the study .
- Therapy (experimental or off-label use of approved therapy) specifically for prevention or treatment of COVID‑19 or COVID‑19 associated cytokine storm is excluded. If the local SoC per written policies or guidelines (ie, not just an individual clinician decision) includes use of off-label medications, then use of these medications during the study is permitted but may require additional safety monitoring by the site. Otherwise, concomitant use of a therapy specifically for management of cytokine storm or for COVID‑19 infection is prohibited due to lack of evidence on additive or synergistic effects when administered in combination with the agent under study in this protocol. Immunomodulatory agents taken for conditions, other than COVID‑19, identified in medical history are not prohibited.

## Treatment after the End of the Study

The Sponsor will not provide any additional care to subjects after they leave the study because such care should not differ from what is normally expected for subjects with COVID‑19.

# Discontinuation of Study Treatment and Subject Discontinuation/Withdrawal

## Discontinuation of Study Treatment

Subjects may be discontinued from study treatment for the following reasons:

1. The subject has experienced a pretreatment event or a treatment-emergent AE that requires early termination because continued participation imposes an unacceptable risk to the subject’s health, or the subject is unwilling to continue because of the pretreatment event or AE.
   - Liver Function Test Abnormalities
   - Study medication must be discontinued immediately with appropriate clinical follow‑up (including repeat laboratory tests) until the subject’s laboratory profile has returned to normal/baseline status, if the following circumstances occur at any time during study medication treatment:
     - ALT or AST >8 × ULN, or
     - ALT or AST >5 × ULN and persists for more than 2 weeks, or
     - ALT or AST >3 × ULN in conjunction with elevated total bilirubin >2 × ULN or international normalized ratio (INR) >1.5, without any anticoagulant treatment, or
     - ALT or AST >3 × ULN with appearance of fatigue, nausea, vomiting, right upper quadrant pain or tenderness, fever, rash and/or eosinophilia (>5%).
2. Major protocol deviation. The discovery postrandomization that the subject failed to meet protocol entry criteria or did not adhere to protocol requirements, and continued participation poses an unacceptable risk to the subject’s health.
3. Lost to follow-up. The subject did not return to the clinic and attempts to contact the subject were unsuccessful. Attempts to contact the subject must be documented.
4. Pregnancy. The subject is found to be pregnant.

Subjects who discontinue study treatment will remain in the study for follow-up and any further evaluations that need to be completed as described in the SoA.

Subjects who discontinue study treatment will not be replaced.

See the SoA (Section 1.3) for data to be collected at the time of treatment discontinuation and follow-up and for any further evaluations that need to be completed.

## Subject Discontinuation/Withdrawal from the Study

A subject may withdraw from the study at any time at his/her own request or may be withdrawn at any time at the discretion of the investigator for safety, behavioral, compliance or administrative reasons.

If the subject withdraws consent for disclosure of future information, the Sponsor may retain and continue to use any data collected before such a withdrawal of consent.

If a subject withdraws from the study, he/she may request destruction of any samples taken and not tested, and the investigator must document this in the study center study records.

## Lost to Follow-up

A subject will be considered lost to follow-up if he or she repeatedly fails to return for scheduled visits and is unable to be contacted by the study center.

The following actions must be taken if a subject fails to return to the clinic for a required study visit:

- The study center must attempt to contact the subject and reschedule the missed visit as soon as possible and counsel the subject on the importance of maintaining the assigned visit schedule and ascertain whether or not the subject wishes to and/or should continue in the study.
- Before a subject is deemed lost to follow‑up, the investigator or designee must make every effort to regain contact with the subject (where possible, 3 telephone calls and, if necessary, a certified letter to the subject’s last known mailing address or local equivalent methods). These contact attempts should be documented in the subject’s medical record.
- Should the subject continue to be unreachable, he/she will be considered to have withdrawn from the study.

# Study Assessments and Procedures

Study procedures and their timing are summarized in the SoA (Section 1.3).

Protocol waivers or exemptions are not allowed.

Immediate safety concerns should be discussed with the Sponsor immediately upon occurrence or awareness to determine if the subject should continue or discontinue study treatment.

Adherence to the study design requirements, including those specified in the SoA (Section 1.3), is essential and required for study conduct.

All screening evaluations must be completed and reviewed to confirm that potential subjects meet all eligibility criteria. The investigator will maintain a screening log to record details of all subjects screened and to confirm eligibility or record reasons for screening failure, as applicable.

Procedures conducted as part of the subject’s routine clinical management (eg, blood count) and obtained before signing of the informed consent form (ICF) may be utilized for screening or baseline purposes provided the procedures met the protocol-specified criteria and were performed within the time frame defined in the SoA (Section 1.3).

**Screening**

Subjects will be assessed for eligibility criteria between Day -2 and Day 1 including a laboratory confirmed diagnosis of SARS‑CoV‑2 infection. The diagnostic test could be either IgG/IgM Rapid test kits or RT-PCR, and results confirming diagnosis must be within 72 hours of Day 1 (randomization).

Clinical screening laboratory evaluations will be performed locally by the site laboratory. The overall eligibility of the subject to participate in the study will be assessed once all screening values are available. Blood for screening laboratory evaluations should be repeated if not done in the preceding 72 hours.

**Demography and Medical History**

The following demographical details will be taken and recorded in the case report form (CRF): gender, year of birth, race and ethnicity.

Wherever possible, relevant medical history within the past 5 years will be recorded in the CRF for past and ongoing conditions. Other details include:

- Day of onset of COVID‑19 symptoms.
- History of chronic medical conditions related to inclusion and exclusion criteria.
- COVID-19-specific history: shortness of breath, cough, fever, malaise, fatigue, myalgia; Past medical history: chronic lung disease, including asthma, chronic renal disease, diabetes, heart disease, hypertension, autoimmune disease, women within 2 weeks postpartum and not breastfeeding, residents of long-term care facility, cancer, organ transplant
- Radiographic lung image (including X-ray, computerized tomography [CT] scan) if performed/available at Screening, Day 1, or during course of hospitalization will be recorded.

**Hospitalization and Diagnosis**

Details will be captured for all subjects regarding:

- Date of hospitalization;
- Date of COVID‑19 diagnosis;
- Date of intensive care unit (ICU) admission;
- Date of discharge from ICU;
- Any readmission dates and redischarge dates.

## Efficacy Assessments

### NEWS2 Score

The National Early Warning score 2 (NEWS2) (Table 7) has demonstrated an ability to discriminate subjects at risk of poor outcomes^(12)^. This score is based on 7 clinical parameters. The NEWS2 Score is being used as an exploratory measure of clinical outcomes.

This should be evaluated at the first assessment of a given study day. These parameters can be obtained from the hospital chart using the last measurement prior to the time of assessment. This is recorded for the day obtained, ie, on Day 3, Day 3 score is obtained and recorded as Day 3.

Table 7 The National Early Warning Score (NEWS) 2

Abbreviations: C = confusion; P = arousable to pain; SpO_2_ = peripheral capillary oxygen saturation; U = unresponsive; V = arousable to voice.
Reproduced from: Royal College of Physicians. National Early Warning Score (NEWS) 2: Standardising the assessment of acute-illness severity in the NHS. Updated report of a working party. London: RCP, 2017 ^(12)^

### Clinical Assessment Scales

**Measures of clinical support**

At each study day while hospitalized, the following measure of clinical support should be assessed:

- Hospitalization
- Survival
- Oxygen requirement - low flow oxygen supplementation days (<40%)/high flow oxygen requirement days (>40%)
- Noninvasive mechanical ventilation (via mask)
- Mechanical ventilator requirement (via endotracheal tube or tracheostomy tube)
- Extracorporeal membrane oxygenation (ECMO) requirement

**Six-point Ordinal Scale**

The ordinal scale is an assessment of the clinical status at the first assessment of a given study day. Each day, the worse score for the previous day will be recorded, ie on Day 3, Day 2 score is obtained and recorded as Day 2. The scale is as follows:

1. Not hospitalized;

2. Hospitalized, not requiring supplemental oxygen;

3. Hospitalized, requiring supplemental oxygen;

4. Hospitalized, on non-invasive ventilation or high flow oxygen devices;

5. Hospitalized, on invasive mechanical ventilation or ECMO;

6. Death.

## Safety Assessments

Planned time points for all safety assessments are provided in the SoA (Section 1.3).

### Physical Examinations

At Screening physical examination will include weight, presenting symptoms; fever, respiratory symptoms, myalgia, malaise, etc.

At subsequent visits a symptom-directed (targeted) physical examination will be performed to evaluate for any possible AE. No physical examination is needed for routine visits.

### Vital Signs

Oral body temperature (Celsius scale with a thermometer), pulse rate (radial), respiratory rate, blood pressure (systolic and diastolic) and peripheral capillary oxygen saturation (SpO_2_).

Blood pressure will be monitored using equipment appropriate to the site of collection, whether it be hospital ICU, hospital ward, or outpatient location. A mercury column sphygmomanometer/digital are considered appropriate medical devices to measure blood pressure. Oxygen saturation will be monitored using a fingertip pulse oximeter or similar device used at the study site.

### Electrocardiogram

Centralized ECG data collection, analysis, and reporting will be used in this study, permitting secure central interpretation, storage, and retrieval of the ECG data. Instructions for the collection (eg, equipment), transmission, and archiving of ECG data will be agreed upon with the central laboratory and summarized.

A 12‑lead electrocardiogram (ECG) will be obtained as outlined in the [SoA](#_Schedule_of_Activities) (Section 1.3) at Days 1 and 29 using an ECG machine that automatically calculates the heart rate and measures PR, QRS, QT, and QTc intervals. Additional ECGs, if performed, will be captured on other hospitalized days and visits.

### Laboratory Assessments

Clinical laboratory evaluations: Fasting is not required before collection of laboratory samples.

- Blood will be collected at the time points indicated in the [SoA](#_Schedule_of_Activities) (Section 1.3).
- Clinical laboratory parameters include complete hematology, serum chemistry, lactate dehydrogenase (LDH), cardiac troponin, D-dimer, and ferritin as outlined in [Appendix 12.3](#Appendix123).
- At screening subjects will be tested for hepatitis B, hepatitis C, and human immunodeficiency virus (HIV).
- This testing will be performed at each clinical trial site in real time.

## Adverse Events

All AEs reported in this study will be coded using the currently available version of the Medical Dictionary for Regulatory Activities (MedDRA). Coding will be to lowest level terms. The preferred term (PT) and the primary system organ class (SOC) will be listed.

Summaries of all treatment emergent AEs (TEAEs) by treatment group will include:

- The number (n) and percentage (%) of subjects with at least 1 TEAE by SOC and PT;
- Treatment emergent AEs by severity, presented by SOC and PT;
- Treatment emergent AEs by relationship to treatment (related, not related), presented by SOC and PT;
- Treatment-emergent AEs of special interest (AESIs) (defined with a PT or a prespecified grouping);
- Deaths and other SAEs will be listed and summarized by treatment group;
- Treatment-emergent AEs leading to permanent treatment discontinuation will be listed and summarized by treatment group.
- The number and proportion of subjects with normal/abnormal laboratory tests or different grades on Day 3, Day 8, Day 15, and Day 29 (or day of discharge) will be presented as shift tables by the baseline status (Day 1).
- Potentially clinically significant abnormality (PCSA) values are defined as abnormal values considered medically important by the Sponsor according to predefined criteria/thresholds based on literature review. The incidence of PCSA vital signs on Day 8, Day 15, and Day 29 (or day of discharge) will be summarized overall and by their baseline normality status (normal/abnormal).
- The number and proportion of subjects with confirmed positive ADA results and with confirmed positive neutralizing activity.

Definition (ICH‑Good Clinical Practice): An AE is any untoward medical occurrence in a subject or clinical investigation subject administered a pharmaceutical product and which does not necessarily have a causal relationship with this treatment.

An AE can therefore be any unfavorable and unintended sign (including an abnormal laboratory finding), symptom, or disease temporarily associated with the use of a medicinal (investigational) product, whether or not related to the medical (investigational) product.

Events Meeting the AE definition:

- Any abnormal laboratory test results (hematology, clinical chemistry, or urinalysis) or other safety assessments (eg, ECG, radiological scans, vital signs measurements), including those that worsen from baseline, considered clinically significant in the medical and scientific judgment of the investigator (ie, not related to progression of underlying disease).
- Exacerbation of a chronic or intermittent pre-existing condition including either an increase in frequency and/or intensity of the condition.
- New conditions detected or diagnosed after study treatment administration even though it may have been present before the start of the study.
- Signs, symptoms or the clinical sequelae of a suspected drug-drug interaction.
- Signs, symptoms or the clinical sequelae of a suspected overdose of either study treatment or a concomitant medication. Overdose per se will not be reported as an AE/SAE unless it is an intentional overdose taken with possible suicidal/self-harming intent. Such overdoses should be reported regardless of sequelae.
- "Lack of efficacy" or "failure of expected pharmacological action" per se will not be reported as an AE or SAE. Such instances will be captured in the efficacy assessments. However, the signs, symptoms and/or clinical sequelae resulting from lack of efficacy will be reported as AE or SAE if they fulfill the definition of an AE or SAE.

Events NOT Meeting the AE definition:

- Any clinically significant abnormal laboratory findings or other abnormal safety assessments which are associated with the underlying disease, unless judged by the investigator to be more severe than expected for the subject’s condition.
- The disease/disorder being studied or expected progression, signs or symptoms of the disease/disorder being studied, unless more severe than expected for the subject’s condition.
- Medical or surgical procedure (eg, endoscopy, appendectomy): the condition that leads to the procedure is the AE.
- Situations in which an untoward medical occurrence did not occur (social and/or convenience admission to a hospital).
- Anticipated day-to-day fluctuations of pre-existing disease(s) or condition(s) present or detected at the start of the study that do not worsen.

Note: Any untoward condition resulting after receiving COVID-19 vaccine will be recorded as an AE and followed up as per protocol.

Adverse events will be reported by the subject (or, when appropriate, by a caregiver, surrogate, or the subject's legally authorized representative).

The investigator and any designees are responsible for detecting, documenting, and recording events that meet the definition of an AE or SAE and remain responsible for following up AEs that are serious, considered related to the study treatment or study procedures, or that caused the subject to discontinue the study treatment (see Section 7.0).

### Documentation and Reporting of AEs

The responsibility of the investigator to record any AEs starts when the ICF is signed through the end of study.

The investigator must record all AEs on standard AE reporting form (IQVIA Biotech template). This form will be included in the CRF booklet.

When an AE occurs, it is the responsibility of the investigator to review all documentation (eg, hospital progress notes, laboratory reports, and diagnostics reports) related to the event.

The Investigator will be required to describe the AE, onset and stop date, severity, the course of action taken, if any, as well as any pertinent data necessary to allow a complete evaluation of the AE. Relevant AE data will be obtained at the study visits, based on information spontaneously provided by the subject and/or thorough questioning of the subject.

If a physician not involved with the study sees a subject in relation to an AE, the investigator should make every effort to contact the treating physician in a timely manner in order to obtain all information necessary to appropriate reporting of the event. As the quality and precision of acquired AE data are critical, Investigators should use the AE definitions provided below and should observe the following guidelines when completing the AE pages of the CRFs.

- Whenever possible, recognized medical terms should be used to describe AEs rather than colloquialisms (for example, ‘influenza’ rather than ‘flu’), and abbreviations should be avoided.
- AEs should be described using specific clinical diagnosis, if this is available, rather than a list of component signs or symptoms (eg, ‘congestive heart failure’ rather than ‘dyspnea, rales and cyanosis’).
- Signs and symptoms that are not linked (as co‑manifestations) to an identified disease or syndrome, or for which an overall diagnosis is not available, should be reported as individual AEs.
- Provisional diagnosis (eg, ‘suspected myocardial infarction’) are acceptable but should be followed up to a definite diagnosis when finally available.
- AEs describing an infection must be as specific as possible, ie, type and location of infection must be provided.

### Assessment of Intensity and Causality of Adverse Events

Investigators need to evaluate the severity of AEs using the following definitions:

- Mild: The subject is aware of the event or symptom, but the event or symptom is easily tolerated causing minimal discomfort and not interfering with everyday activities.
- Moderate: The subject experiences sufficient discomfort to interfere with or reduce his or her usual level of activity.
- Severe: Significant impairment of functioning: the subject is unable to carry out usual activities and therapeutic intervention is required.

An AE that is assessed as severe should not be confused with a SAE. Severe is a category utilized for rating the intensity of an event; and both AEs and SAEs can be assessed as severe.

Investigator is obligated to assess the relationship between study treatment and each occurrence of each AE

- Definitely Related: A clinical event, including laboratory test abnormality, occurring in a plausible time relationship to the medication administration, and which cannot be explained by concurrent disease or other drugs or chemicals. The response to the withdrawal of the drug should be clinically plausible.
- Probably Related: A clinical event, including laboratory test abnormality, with a reasonable time sequence to administration of the drug, unlikely to be attributed to concurrent disease or other drugs or chemicals, and which follows a clinically reasonable response on withdrawal (de-challenge). Re-challenge information is not required to fulfil this definition.
- Possibly Related: A clinical event, including laboratory test abnormality, with a reasonable time sequence to the medication administration, but which could also be explained by concurrent disease or other drugs or chemicals. Information on the drug withdrawal may be lacking or unclear.
- Unlikely Related: A clinical event, including laboratory test abnormality, with little or no temporal relationship to medication administration, and for which other drugs, chemicals or underlying disease provide a plausible explanation.
- Not Related: A clinical event, including laboratory test abnormality that has no temporal relationship to the medication or has more likely the alternative etiology.
- “Not Related” and “Unlikely Related” will be defined as unrelated to the investigational product; “Possibly Related”, “Probably Related” and “Definitely Related” will be defined as related to the investigational product. The investigator will use clinical judgment to determine the relationship.
- Alternative causes, such as underlying disease(s), concomitant therapy, and other risk factors, as well as the temporal relationship of the event to study treatment administration will be considered and investigated.
- The investigator will also consult the Investigator’s Brochure and/or Product Information, for marketed products, in his/her assessment.
- For each AE, the investigator must document in the medical notes that he/she has reviewed the AE and has provided an assessment of causality.
- The causality assessment is one of the criteria used when determining regulatory reporting requirements.

### Follow-up of AEs

The investigator is obligated to perform or arrange for the conduct of supplemental measurements and/or evaluations as medically indicated or as requested by the CRO to elucidate the nature and/or causality of the AE or SAE as fully as possible. This may include additional laboratory tests or investigations, histopathological examinations, or consultation with other health care professionals.

If a subject dies during participation in the study or during a recognized follow-up period, the investigator will provide the CRO with a copy of any postmortem findings including histopathology.

New or updated information will be recorded in the originally completed CRF.

The investigator will submit any updated SAE data to the Sponsor within 24 hours of receipt of the information.

### Serious Adverse Events

If an event is not an AE per the definition above, then it cannot be an SAE even if serious conditions are met (eg, hospitalization for signs/symptoms of the disease under study, death due to progression of disease).

An SAE is defined as any untoward medical occurrence that, at any dose:

1. Results in death
2. Is life-threatening

The term ‘life-threatening’ in the definition of “serious” refers to an event in which the subject was at risk of death at the time of the event. It does not refer to an event, which hypothetically might have caused death, if it were more severe.

1. Requires inpatient hospitalization or prolongation of existing hospitalization

In general, hospitalization signifies that the subject has been detained (usually involving at least an overnight stay) at the hospital or emergency ward for observation and/or treatment that would not have been appropriate in the physician’s office or outpatient setting. Complications that occur during hospitalization are AEs. If a complication prolongs hospitalization or fulfills any other serious criteria, the event is serious. When in doubt as to whether “hospitalization” occurred or was necessary, the AE should be considered serious.

Hospitalization for elective treatment of a pre-existing condition that did not worsen from baseline is not considered an AE.

1. Results in persistent disability/incapacity

The term disability means a substantial disruption of a person’s ability to conduct normal life functions. This definition is not intended to include experiences of relatively minor medical significance such as uncomplicated headache, nausea, vomiting, diarrhea, influenza, and accidental trauma (eg, sprained ankle) which may interfere with or prevent everyday life functions but do not constitute a substantial disruption.

1. Is a congenital anomaly/birth defect
2. Other situations:

Medical or scientific judgment should be exercised in deciding whether SAE reporting is appropriate in other situations such as important medical events that may not be immediately life-threatening or result in death or hospitalization but may jeopardize the subject or may require medical or surgical intervention to prevent 1 of the other outcomes listed in the above definition. These events should usually be considered serious.

Examples of such events include invasive or malignant cancers, intensive treatment in an emergency room or at home for allergic bronchospasm, blood dyscrasias or convulsions that do not result in hospitalization, or development of drug dependency or drug abuse.

#### SAE Reporting and Follow-up

Serious AE Reporting to the Medical Monitor via an Electronic Data Collection Tool

- The primary mechanism for reporting an SAE to the Medical Monitor will be the electronic data collection tool.
- If the electronic system is unavailable for more than 24 hours, then the study center will use the paper SAE data collection tool (see next section).
- The study center will enter the SAE data into the electronic system as soon as it becomes available.
- After the study is completed at a given study center, the electronic data collection tool will be taken off-line to prevent the entry of new data or changes to existing data.
- If a study center receives a report of a new SAE from a subject or receives updated data on a previously reported SAE after the electronic data collection tool has been taken off-line, then the study center can report this information on a paper SAE form (see next section) or to the Medical Monitor/designated CRO Safety Team by telephone/fax or email.
- Contacts for SAE reporting can be found in the safety paper forms and the safety management plan.

SAE Reporting to the Medical Monitor/designated CRO Safety Team via paper SAE form

- The secondary (backup) mechanism for reporting an SAE to the Medical Monitor/designated CRO Safety Team will be via a paper SAE form.
- E-mail/Facsimile transmission of the paper SAE form is the preferred method to transmit this information to the Medical Monitor/designated CRO Safety Team.
- In rare circumstances and in the absence of facsimile equipment, notification by telephone is acceptable with a copy of the SAE data collection tool sent by overnight mail or courier service.
- Initial notification via telephone does not replace the need for the investigator to complete and sign the SAE CRF pages within the designated reporting time frames.
- Contacts for SAE reporting can be found in the SAE paper form and the safety management plan.

Any ongoing SAEs at the time of the subject’s participation in the study will be followed up thereafter until an outcome is known, eg, resolution is reached.

#### Assessment of Intensity and Causality of Serious Adverse Events

Refer to [Section 8.3.2](#_Assessment_of_Intensity).

### Adverse Events of Special Interest

Adverse events of special interest (AESIs) must be recorded in the eCRF. If serious criteria are met, then follow the SAE reporting and follow-up process. Adverse events of special interest include:

- Disease related secondary infection complications
- Grade 4 (Common Terminology Criteria for Adverse Events V5) neutropenia and lymphopenia
- Anaphylactic reactions defined by Clinical Criteria for Diagnosing Anaphylaxis^(11)^
- 20% decline in SpO_2_ between start and end of 1-hour study treatment infusion
- ALT or AST >3 x ULN AND TBL >2 x ULN
- Evidence of RBC hemolysis as defined by 2 of the following 3 findings:
  - Anemia that is not due to another obvious cause;
  - Increased reticulocyte count that is not explained by an obvious cause,
  - Signs of RBC destruction, such as increased LDH, low haptoglobin ≤25 mg/dL, increased unconjugated bilirubin.

### Regulatory Reporting Requirements for SAEs

- Prompt notification by the Investigator to the Sponsor of an SAE is essential so that legal obligations and ethical responsibilities toward the safety of subjects and the safety of a study treatment under clinical investigation are met.
- The Sponsor has a legal responsibility to notify both the local regulatory authority and other regulatory agencies about the safety of a study treatment under clinical investigation. The Sponsor will comply with country-specific regulatory requirements relating to safety reporting to the regulatory authority, Institutional Review Boards (IRB)/Independent Ethics Committees (IEC), and Investigators.
- Investigator safety reports must be prepared for suspected unexpected serious adverse reactions (SUSAR) according to local regulatory requirements and Sponsor policy and forwarded to Investigators as necessary.
- An Investigator who receives an Investigator safety report describing an SAE or other specific safety information (eg, summary or listing of SAEs) from the Sponsor will review and then file it along with the Investigator’s Brochure and will notify the IRB/IEC, if appropriate according to local requirements.

### Pregnancy

Details of all pregnancies in female subjects and, if indicated, female partners of male subjects, will be collected after the start of study treatment until term or outcome.

If a pregnancy is reported, the investigator should inform the Sponsor within 24 hours of learning of the pregnancy; however pregnancies should not be reported as AEs.

Abnormal pregnancy outcomes (eg, spontaneous abortion, fetal death, stillbirth, congenital anomalies, ectopic pregnancy) are considered SAEs.

## Treatment of Overdose

An overdose is defined as a known deliberate or accidental administration of investigational drug, to or by a study subject, at a dose above that which is assigned to that individual subject according to the study protocol.

The Sponsor does not recommend specific treatment for an overdose. In the event of an overdose, the investigator should:

1. Contact the Medical Monitor immediately.
2. Closely monitor the subject for any AE/SAE and laboratory abnormalities until meplazumab can no longer be detected systemically (at least 125 days).
3. Document the quantity of the excess dose as well as the duration of the overdose in the eCRF.

Decisions regarding dose interruptions or modifications will be made by the investigator in consultation with the Medical Monitor based on the clinical evaluation of the subject.

No specific antidote is currently available for meplazumab. In case of overdose, it is recommended that the appropriate supportive medical care is provided. In case of allergic reaction, withdrawal of study medication is advised. For treatment of allergic reactions, current guidelines for treatment (eg, with corticosteroids) should be applied.

## Pharmacokinetics

Venous blood samples will be collected to evaluate the PK of meplazumab in serum and whole blood cells (when feasible); however, a missed blood collection will not be considered a protocol deviation. Blood samples will be taken either by direct venipuncture (any suitable vein) or an indwelling cannula inserted in a forearm vein. On the day of meplazumab administration, samples should be collected in the contralateral (opposite) arm from the one being used for drug infusion. The actual date and time (24‑hour clock time) of each sample collection will be recorded in the eCRF. Any sampling problems will be documented in the eCRF. The start and end date/time of each infusion as well as any infusion interruptions will also be documented in the eCRF.

Each sample will be divided into 2 aliquots (a primary PK and a back-up sample). Samples collected for analyses of meplazumab concentration may also be used to evaluate safety or efficacy aspects related to concerns arising during or after the study. Instructions for the collection and handling of biological samples will be provided by the Sponsor in a separate laboratory manual.

The time points and windows for PK sample collection are specified for Stage 1 as follows while the subject is hospitalized, or if the subject returns for other blood collections after discharge:

- at the end of first infusion (+10 minutes) and 2 (+2) hours relative to the end of the first infusion,
- either on Day 2 or on Day 3 (within approximately 20 to 52 hours relative to end of infusion);
- prior to and at the end of the second infusion (+10 minutes) on Day 8,
- either on Day 9 or on Day 10 (within approximately 20 to 52 hours relative to end of second infusion);
- Day 29 (±3 days at time of ADA collection); and
- Day 57 (±7 days at time of ADA collection).

Sample should be collected when operationally feasible.

Stage 2 sampling times will be determined at the interim analysis based on emerging data. Sample collections will remain the same unless changes are warranted. At a minimum, in all Stage 2 subjects, PK samples will continue to be collected at the time of ADA collection. However, the number of subjects from whom additional blood samples for PK will be collected as well as the schedule for the additional samples may be reduced; this will depend on the number of Stage 2 subjects in the 2 meplazumab dose arms who are able to provide samples and the extent of sample collection in these subjects.

Drug concentration information that would unblind the study will not be reported to investigative sites or blinded personnel until the study has been unblinded.

Samples for the determination of meplazumab in serum and blood cells will be analyzed using appropriate validated bioanalytical methods. Full details of the bioanalytical methods will be described in a separate Bioanalytical Report. All samples in subjects who received meplazumab still within the known stability of the analyte of interest at the time of receipt by the bioanalytical laboratory will be analyzed. Samples collected from subjects who received control will not be analyzed.

In subjects with sufficient serum and/or blood cell meplazumab data, an attempt will be made to calculate PK parameters following the infusion regimen. Parameters will be calculated by noncompartmental analysis using actual elapsed time from dose relative to the start of infusion on Day 1 (and separately for each infusion, if appropriate) with Phoenix® WinNonlin® Version 8.0 or higher (Certara, LP Princeton, New Jersey, United States) and/or SAS® Version 9.4 or higher (SAS Institute, Inc, Cary, North Carolina, United States). Parameters, if calculable, will include but are not limited to:

- Maximum observed concentration (C_max_) following the first infusion and overall
- Time to C_max_ (t_max_) following the first infusion and overall
- Area under the concentration versus time curve to the last quantifiable concentration AUC_(0‑last)_
- Area under the concentration versus time curve from time zero extrapolated to infinity AUC_(0-inf)_
- Elimination half-life (t½)
- Systemic clearance (CL)
- Volume of distribution (Vz)
- Steady-state volume of distribution (Vss)

Other PK parameters may also be calculated as appropriate. Further details will be provided in the statistical analysis plan (SAP).

## Pharmacodynamics

Venous blood samples will be collected to assess changes from baseline in cytokine, and chemokines related to inflammatory and immune status. Pharmacodynamic endpoints may include:C–X–C motif chemokine 10 (CXCL10); cyclophilin A (CyPA); granulocyte colony‑stimulating factor (G‑CSF); Tumor Necrosis Factor alpha (TNFα); interferon gamma (IFN‑γ); interleukin‑1 Receptor Antagonist (IL‑1 RA); interleukin‑2 (IL-2); interleukin-2 Receptor alpha (IL-2 Rα); interleukin‑4 (IL-4); interleukin‑6 (IL‑6);  interleukin‑7 (IL-7); interleukin‑8 (IL‑8); interleukin‑10 (IL‑10); interleukin‑15 (IL‑15);  interleukin‑17A (IL‑17A); interleukin12p70 (IL-12p70); monocyte chemoattractant protein‑1 (MCP‑1); macrophage colony‑stimulating factor (M‑CSF); macrophage inflammatory protein‑1 alpha (MIP‑1α); macrophage inflammatory protein-1 beta (MIP-1β); and high-sensitivity C-reactive protein (hsCRP). Blood samples will be collected when operationally feasible at the following sampling times:

- prior to start of the first infusion (baseline);
- either on Day 2 or Day 3 (within approximately 20 to 52 hours relative to end of infusion);
- prior to second infusion on Day 8;
- either on Day 9 or on Day 10 (within approximately 20 to 52 hours relative to end of second infusion);
- Day 29 (±3 days at time of ADA collection).

When scheduled at the same day as the PK samples on Day 2 and subsequent study days, both pharmacodynamic (PD) and PK samples should be collected at the same time. A missed blood collection will not be considered a protocol deviation.

This sampling schedule is specified for Stage 1. Sample collections will remain the same unless changes are warranted. Stage 2 sampling times will be determined at the interim analysis based on emerging data. Instructions for the collection and handling of biological samples will be provided by the Sponsor in a separate laboratory manual. The actual date and time (24‑hour clock time) of each sample will be recorded.

## Biomarkers

### Immunogenicity Assessments

Antibodies to meplazumab will be evaluated in serum samples collected from all subjects according to the Schedule of Assessments (Section 1.3). Collection of samples for analysis of ADAs is mandatory and they should be collected whenever feasible. These samples will be tested by the Sponsor or Sponsor’s designee.

Serum samples will be screened for antibodies binding to meplazumab and the titer of confirmed positive samples will be reported. Antibodies will be further characterized and/or evaluated for their ability to neutralize the activity of meplazumab. Other analyses may be performed to verify the stability of antibodies to meplazumab and/or further characterize the immunogenicity of meplazumab. The detection and characterization of antibodies to meplazumab will be performed using a validated assay method by or under the supervision of the Sponsor. Samples will be collected, labeled, stored, and shipped as detailed in the Laboratory Manual.

Samples may be stored for a maximum of 5 years (or according to local regulations) following the last subject’s last visit for the study at a facility selected by the Sponsor to enable further analysis of immune responses to meplazumab.

### Other Research

If feasible, nasopharyngeal (NP) swabs for viral load assessments (quantitative PCR for COVID‑19) will be collected, at baseline (Day 1 prior to infusion), Day 3, Day 5, Day 8 (prior to the second infusion), and on either Day 9 or Day 10 at the time of PD sample collection, and on Day 29. If viral RNA is still detected at Day 29, a follow-up sample for viral RNA testing and Day 57 (optional). A missed sample collection will not be considered a protocol deviation. Viral load assessment will be performed using an appropriate assay method by or under the supervision of the Sponsor. Samples will be collected, labeled, stored, and shipped as detailed in the Laboratory Manual.

## Health Economics/Medical Resource Utilization and Health Economics

Health Economics/Medical Resource Utilization and Health Economics parameters are not evaluated in this study.

# Statistical Considerations

## Sample Size Determination

Stage 1: Approximately 168 subjects will be randomized and allocated 1:1:1:1 (42:42:42:42) to receive meplazumab low dose, meplazumab medium dose, meplazumab high dose or control. Following a 29 day treatment period after completion of randomization, an interim analysis will be conducted to select the optimal meplazumab dose based on the response rates of clinical improvement at Day 29 between 3 dose levels, and safety data, details will be found in IDMC charter. Using a step-down procedure for the comparisons between the dose groups and the placebo at a 2-sided alpha level of 0.05 each, forty-two subjects per arm will power the study at 81% to distinguish the response rate at Day 29 of the dose groups from the placebo, assuming 80% response rate for the dose groups and 50% for the placebo.

Stage 2: Sample size for Stage 2 will be re‑evaluated based on Stage 1 results and the selected primary endpoint; 240 more subjects will be randomized and allocated 2:1 (160:80) to receive the optimal meplazumab dose determined after Stage 1 or control. A total sample size of 240 on optimal meplazumab dose and control, has at least 95% power to detect an actual 30% difference on the response rate of clinical improvement (50% versus 80%) between meplazumab and control at 2‑sided alpha level 0.05.

At interim analysis, primary endpoint, sample size calculation for Stage 2 will be re‑evaluated based on the observed outcomes at Stage 1 and will be capped at 300 subjects in total.

Data collected at Stage 1 and Stage 2 will be analyzed separately.

## Populations for Analyses

For purposes of analysis, the following populations are defined.

Enrolled population: All subjects who sign the ICF; this definition will be applied to the Stage 1 enrolled set and Stage 2 enrolled set.

Intention-to-treat (ITT) population: all subjects who are randomized. This definition will be applied to the Stage 1 ITT set and Stage 2 ITT set.

Safety population: All subjects who are randomized and take at least 1 dose of study medication (meplazumab or control) will be included in the safety set. This definition will be applied to the Stage 1 safety set and Stage 2 safety set.

Pharmacokinetic analysis set (PKS): All subjects who receive meplazumab and have quantifiable meplazumab concentrations postdose without protocol deviations or events affecting the PK results will be included in the PKS. This definition will be applied to the Stage 1 PKS set and Stage 2 PKS set.

Pharmacokinetic parameter analysis set (PKPS): All subjects who receive meplazumab and have at least 1 PK parameter result without deviation or events affecting the results will be included in the PKPS. This definition will be applied to the Stage 1 PKPS set and Stage 2 PKPS set, as appropriate.

Pharmacodynamic analysis set (PDS): All subjects who receive study medication (drug or control) and have evaluable results for at least 1 PD endpoint postdose. The above definition of PDS set will be applied to the Stage 1 PDS set and Stage 2 PDS set.

## Statistical Analyses

The SAP will be developed and finalized before database lock and will describe the subject analysis sets to be included in the analyses, and procedures for accounting for missing, unused, and spurious data. This section is a summary of the planned statistical analyses of the primary and secondary endpoints.

### Efficacy Analyses

| **Endpoint** | **Statistical Analysis Methods** |
| --- | --- |
| Primary | Stage 1:There is no primary endpoint in Stage 1.  Dose selection endpoint: Response rate at Day 29 of the low, medium, and high dose groups will be compared against that of the placebo group using Chi-Square test. The comparison of the response rate between the dose groups and the placebo will be tested with a step-down procedure at the 2-sided alpha level of 0.05 each, and will proceed in the following order, high versus placebo, medium versus placebo, and low versus placebo. If one preceding testing fails to be rejected, all the following will not be tested.  Subjects in the Stage 1 ITT set will be used.  Primary endpoints for Stage 2 will be determined based on Stage 1 results.  Stage 2:  The analysis of response rate at Day 29 will be conducted between the selected dose group and control using Cochran–Mantel–Haenszel (CMH) statistic, stratifying for age group (age <65 years versus ≥65 years), and baseline severity grade, and additional stratification factors if any as determined after evaluation of Stage 1 data. The p value associated with the CMH statistic will be compared at the 2-sided 0.05 alpha level.  Sensitivity analyses on the response rate at Day 29 will be to fit the response variable using logistic regression, including treatment, baseline, baseline and treatment interaction, age group (age <65 years versus ≥65 years), and age group and treatment interaction as fixed effects. Model-based point estimates for the treatment effects, 95% confidence intervals (CIs), and p-values will be calculated.  Other candidate binary endpoints, including mortality at Day 29 and proportion of subjects alive and discharged without supplemental oxygen at Day 29, will be analyzed using the same CMH test and logistic regression as above.  The time to event endpoints, including time to sustained clinical improvement by Day 29 from treatment start date, will be compared between the treatment arms. Time to event for subjects who die or withdraw from the study during the evaluation period (Day 1 to Day 29) for other reasons will be censored at the end of analysis period (i.e., Day 29). The Kaplan Meier estimator will be used, Kaplan Meier curves will be plotted for each treatment arm, and the log-rank test will be used for comparing the treatment arms at the 2 sided alpha level of 0.05. In addition, Cox regression model will be used to model the subdistribution hazard ratio (HR) between treatment arms under the Fine and Gray’s competing risk framework, with death handed as competing risk; the HR and its 95% CI will be reported.  Other continuous endpoints will be summarized with descriptive statistics such as mean, standard deviation (SD), median, min, and max.  Subgroup analyses may be performed by age group, gender, and race, and concomitant antiviral agents.  Subjects in the Stage 2 ITT set will be used. |
| Secondary | Stage 1: There is no secondary endpoint in Stage 1.  Stage 2: Binary endpoints, including response rates at Day 2, 8, and 15 and mortality rates at Days 15, and 57, proportion of live discharges at Day 15 and 57, will be using the same CMH test and logistic regression model as in the primary endpoint.  Time to event endpoints, including time to clinical improvement, time to death, and time to live discharge, from treatment start date will be using the same survival analysis model as in the primary analysis.  Other continuous endpoints will be summarized with descriptive statistics such as mean, SD, median, minimum and maximum. |
| Exploratory | - To evaluate PK exposure to meplazumab - To evaluate PD response to administration of meplazumab - To explore potential exposure-response relationships - To evaluate the efficacy of the selected dose using a composite ranked outcome trajectory score |

### Safety Analyses

All safety analyses will be performed on the Safety Analysis Set.

All AEs reported in this study will be coded using the currently available version of the MedDRA. Coding will be to lowest level terms. The PT, and the primary system organ class (SOC) will be listed.

Summaries of all TEAEs by treatment group will include:

- The number (n) and percentage (%) of subjects with at least 1 TEAE by SOC and PT
- Treatment emergent AEs by severity, presented by SOC and PT
- Treatment emergent AEs by relationship to treatment (related, not related), presented by SOC and PT
- Treatment-emergent AESIs (defined with a PT or a prespecified grouping)
- Deaths and other SAEs will be listed and summarized by treatment group.
- Treatment-emergent AEs leading to permanent treatment discontinuation will be listed and summarized by treatment group.

The number and proportion of subjects with normal/abnormal laboratory tests or different grades on Day 3, Day 8, Day 15, and Day 29 (or day of discharge) will be presented as shift tables by the baseline status (Day 1).

Potentially clinically significant abnormality (PCSA) values are defined as abnormal values considered medically important by the Sponsor according to predefined criteria/thresholds based on literature review. The incidence of PCSA vital signs on Day 8, Day 15, and Day 29 (or day of discharge) will be summarized overall and by their baseline normality status (normal/abnormal).

The number and proportion of subjects with confirmed positive ADA results and with confirmed positive neutralizing activity.

### Other Analyses

Pharmacokinetic results (meplazumab concentration and PK parameters, as appropriate), PD, and biomarker exploratory analyses will be described in the SAP finalized before database lock. Pharmacodynamic and biomarker exploratory analyses may be presented separately from the main CSR. Meplazumab exposure versus response variables may be graphically and statistically displayed for select endpoints. Exposure-response data obtained from this study may be modeled and/or combined with data from other studies and used for modeling and simulations. If modeling and/or simulations are performed, a separate data analysis plan will be prepared and the results will be reported separately from the main CSR.

### Missing Data

All subjects recruited into the study will be accounted for, including those who did not complete the study. Subjects who withdraw from the study will have the reasons of withdrawal collected in the CRF. A noncompleter in the endpoints of the response rate analyses will be treated as nonresponder. More details will be described in the SAP before data base lock.

## Interim Analyses

Dose selection at the interim analysis will be made by monitoring both the efficacy data including response rates of 3 dose arms, time to event endpoints, and safety data. The primary efficacy endpoint selection will be determined by the IDMC at the end of Stage 1 based on the data collected, which could depend on their clinical relevance and strength to detect the treatment difference between optimal dose and control. Sample size for Stage 2 will be re‑evaluated based on Stage 1 results and the selected primary endpoint. Futility analysis comparing the efficacy of the dose arms to control will be conducted after Stage 1.

As part of the interim analysis at the end of Stage 1, meplazumab concentration (as available) will be listed and summarized by scheduled collection time and treatment for the PKS. Similarly, meplazumab PK parameters (as available) will be listed and summarized by scheduled collection time and treatment for the PKPS. An interim PK analysis during Stage 2 is not planned. Pharmacodynamic/biomarker endpoints may be evaluated during the interim analysis at the end of Stage 1; however, if performed, the decision on dose selection will be based on the efficacy results.

For Stage 1, efficacy and safety data collected up to Day 29 from the last subject randomization date will be included in the interim analysis report. Pharmacokinetic and PD data will be included in the report only if available by the cutoff date.

Stage 2 interim analysis for efficacy will be determined and described after the primary endpoint is selected.

The SAP will describe the planned interim analyses in greater detail.

## Data Monitoring Committee

An IDMC will be responsible for safeguarding the safety of subjects and for general oversight of the study conduct. The IDMC will have the following responsibilities:

- Review results of the interim analysis
- Review of interim safety data at regular intervals while subjects remain on study treatment
- Review of individual safety issues as requested by the Medical Monitor during the course of the study with the goal of recommending an appropriate course of action.

The operating principles, roles and responsibilities of the IDMC will be fully described in the IDMC Charter*.*

# DATA HANDLING, RECORDING and QUALITY ASSURANCE

## Data Handling and Record Keeping

The investigator must ensure that proper source documentation for all activities performed in relation to this study are sufficiently maintained and securely kept. The investigator will transfer all relevant data from the source documents to the CRF as stipulated in this study protocol and his/her signature on the CRF guarantees the completeness and integrity of these data. It is the investigator’s responsibility to retain study essential documents for at least 15 years since the formal discontinuation of the study. These documents should be retained for a longer period if required by an agreement with the Sponsor. In such an instance, it is the responsibility of the Sponsor to inform the investigator/institution as to when these documents no longer need to be retained.

## Source Documents

The investigator/institution should maintain adequate and accurate source documents and study records that include all pertinent observations on each of the study center’s subjects. Source data should be attributable, legible, contemporaneous, original, accurate and complete. Changes to source data should be traceable, should not obscure the original entry, and should be explained if necessary (eg, via an audit trail).

Source documents provide evidence for the existence of the subject and substantiate the integrity of the data collected. Source documents are filed at the investigator’s study center.

Data reported on the CRF or entered in the eCRF that are transcribed from source documents must be consistent with the source documents or the discrepancies must be explained. The investigator may need to request previous medical records or transfer records, depending on the study. Also, current medical records must be available.

## Data Quality Assurance

All subject data relating to the study will be recorded on eCRFs unless transmitted to the Sponsor or designee electronically (eg, laboratory data). The investigator is responsible for verifying that data entries are accurate and correct by physically or electronically signing the eCRF.

The investigator must maintain accurate documentation (source data) that supports the information entered in the eCRF. The investigator must permit study-related monitoring, audits, IRB/IEC review and regulatory agency inspections and provide direct access to source data documents. The Sponsor or designee is responsible for the data management of this study including quality checking of the data.

Study monitors will perform ongoing remote data review and remote data verification, where available, to confirm that data entered into the eCRF by authorized study center personnel are accurate, complete, and wherever possible verifiable from source documents; that the safety and rights of subjects are being protected; and that the study is being conducted in accordance with the currently approved protocol and any other study agreements, ICH GCP, and all applicable regulatory requirements.

Records and documents, including signed ICFs, pertaining to the conduct of this study must be retained by the investigator for 15 years after study completion unless local regulations or institutional policies require a longer retention period. No records may be destroyed during the retention period without the written approval of the Sponsor. No records may be transferred to another location or party without written notification to the Sponsor.

# References

1. Tang X, Wu C, Li X, et al. On the origin and continuing evolution of SARS‑CoV‑2. *Natl Sci Rev.* 2020;0:1-12.
2. Chan J, Yuan S, Kok K, To K, Chu H, Yang J, et al. A familial cluster of pneumonia associated with the 2019 novel coronavirus indicating person-to-person transmission: a study of a family cluster. *Lancet.* 2020;395(10223):514-523.
3. Guan W, Ni Z, Hu Y, Liang W, Ou C, He J, et al. Clinical Characteristics of Coronavirus Disease 2019 in China. *N Engl J Med.* 2020;382:1708-1720.
4. Li Q, Guan X, Wu P, Wang X, Zhou L, Tong Y, et al. Early Transmission Dynamics in Wuhan, China, of Novel Coronavirus–Infected Pneumonia. *N Engl J Med.* 2020;382:1199‑1207.
5. Ruan Q, Yang K, Wang W, Jiang L, Song J. Clinical predictors of mortality due to COVID‑19 based on an analysis of data of 150 patients from Wuhan, China. *Intensive Care Med*. 2020:46;846–848.
6. Karakike E, Giamarellos-Bourboulis E. Macrophage Activation-Like Syndrome: A Distinct Entity Leading to Early Death in Sepsis. *Frontiers in Immunology.* 2019;10(55):1‑10
7. Ramos-Casals M, Brito-Zerón P, López-Guillermo A, Khamashta M, Bosch X. Adult haemophagocytic syndrome. *Lancet*, 2014:383(9927);1503-1516.
8. Seguin A, Galicier L, Boutboul D, Lemiale V, Azoulay E. Pulmonary Involvement in Patients With Hemophagocytic Lymphohistiocytosis. *Chest.* 2016;149(5):1294-1301.
9. Huang C, Wang Y, Li X, Ren L, Zhao J, Hu Y, et al. Clinical features of patients infected with 2019 novel coronavirus in Wuhan, China. *Lancet.* 2020;395(10223):497-506.
10. Wu Z, McGoogan J. Characteristics of and Important Lessons From the Coronavirus Disease 2019 (COVID‑19) Outbreak in China. *JAMA.* 2020;323(13):1239-1242.
11. Sampson HA, Muñoz-Furlong A, Campbell RL, Adkinson Jr NF, Bock SA, Branum A, et al. Second Symposium on the Definition and Management of Anaphylaxis: Summary report--Second National Institute of Allergy and Infectious Disease/Food Allergy and Anaphylaxis Network Symposium. *J Allergy Clin Immunol*. 2006;117(2):391-397.
12. Royal College of Physicians. National Early Warning Score (NEWS) 2 standardising the assessment of acute-illness severity in the NHS. 2017. https://www.rcplondon.ac.uk/projects/outputs/national-early-warning-score-news-2

# Appendices

## Abbreviations

| Abbreviation | Definition |
| --- | --- |
| ADA | Antidrug antibody |
| AE | Adverse event |
| AESI | Adverse event of special interest |
| ALT | Alanine aminotransferase |
| ARDS | Acute respiratory distress syndrome |
| AST | Aspartate aminotransferase |
| AUC_(0-inf)_ | Area under the concentration versus time curve from time zero extrapolated to infinity |
| AUC_(0-last)_ | Area under the concentration versus time curve from time zero to the last quantifiable concentration |
| CD147 | Cluster of differentiation 147 |
| CL | Systemic clearance |
| C_max_ | Maximum observed concentration |
| CMH | Cochran–Mantel–Haenszel |
| COVID‑19 | Coronavirus disease 2019 |
| CPK | Creatine phosphokinase |
| CRF | Case report form |
| CRO | Contract research organization |
| CSR | Clinical study report |
| CXCL10 | C–X–C motif chemokine 10 |
| CyPA | Cyclophilin A |
| ECG | Electrocardiogram |
| ECMO | Extracorporeal membrane oxygenation |
| eCRF | Electronic case report form |
| FDA | Food and Drug Administration |
| FSH | Follicle stimulating hormone |
| G-CSF | Granulocyte colony-stimulating factor |
| HRT | Hormonal replacement therapy |
| IC50 | Half-maximal inhibitory concentration |
| ICF | Informed consent form |
| ICH | International Council for Harmonisation |
| ICU | Intensive care unit |
| IEC | Independent Ethics Committee |
| IFN | Interferon |
| IFN-γ | Interferon gamma |
| Ig | Immunoglobulin |
| IL-1RA | Interleukin-1 Receptor Antagonist |
| IL-2 | Interleukin-2 |
| IL-2Rα | Interleukin-2 Receptor alpha |
| IL-4 | Interleukin-4 |
| IL-6 | Interleukin-6 |
| IL-7 | Interleukin-7 |
| IL-8 | Interleukin-8 |
| IL-10 | Interleukin-10 |
| IL-15 | Interleukin-15 |
| IL-17A | Interleukin-17A |
| IL-12p70 | Interleukin-12p70 |
| IDMC | Independent Data Monitoring Committee |
| IRB | Institutional Review Board |
| ITT | Intent-to-treat |
| IV | Intravenous |
| IWRS | Interactive web response system |
| KM | Kaplan-Meier |
| LDH | Lactate dehydrogenase |
| MCP-1 | Monocyte chemoattractant protein-1 |
| MedDRA | Medical Dictionary for Regulatory Activities |
| MIP-1α | Macrophage inflammatory protein-1 alpha |
| MIP-1β | Macrophage inflammatory protein-1 beta |
| NEWS2 | National Early Warning score 2 |
| NP | Nasopharyngeal |
| PCR | Polymerase chain reaction |
| PCSA | Potentially clinically significant abnormality |
| PD | Pharmacodynamic |
| PDS | Pharmacodynamic analysis set |
| PK | Pharmacokinetic |
| PKS | Pharmacokinetic analysis set |
| PKPS | Pharmacokinetic parameter analysis set |
| PT | Preferred term |
| RBC | Red blood cell |
| RO | Receptor occupancy |
| SAE | Serious adverse event |
| SAP | Statistical analysis plan |
| SARS-CoV-2 | Severe acute respiratory syndrome coronavirus 2 |
| SD | Standard deviation |
| sHLH | Secondary hemophagocytic lymphohistiocytosis |
| SoA | Schedule of Activities |
| SoC | Standard of Care |
| SOC | System organ class |
| SpO_2_ | Peripheral capillary oxygen saturation |
| t½ | Elimination half-life |
| TBL | Total bilirubin |
| TEAE | Treatment emergent adverse event |
| t_max_ | Time to maximum concentration |
| ULN | Upper limit of normal |
| Vss | Steady-state volume of distribution |
| Vz | Volume of distribution |
| WOCBP | Woman of childbearing potential |

## Regulatory, Ethical, and Study Oversight Considerations

Regulatory and Ethical Considerations

- This study will be conducted in accordance with the protocol and with the following:
  - Consensus ethical principles derived from international guidelines including the Declaration of Helsinki and Council for International Organizations of Medical Sciences (CIOMS) International Ethical Guidelines.
  - Applicable ICH Good Clinical Practice (GCP) Guidelines.
  - Applicable laws and regulations.
- The protocol, protocol amendments, ICF, Investigator Brochure, and other relevant documents (eg, advertisements) must be submitted to an IRB/IEC by the Investigator and reviewed and approved by the IRB/IEC before the study is initiated.
- Any amendments to the protocol will require IRB/IEC and regulatory authority approval, when applicable, before implementation of changes made to the study design, except for changes necessary to eliminate an immediate hazard to subjects.
- The Investigator will be responsible for the following:
  - Providing written summaries of the status of the study to the IRB/IEC annually or more frequently in accordance with the requirements, policies, and procedures established by the IRB/IEC.
  - Notifying the IRB/IEC of SAEs or other significant safety findings as required by IRB/IEC procedures.
  - Providing oversight of the conduct of the study at the study center and adherence to requirements of 21 CFR, ICH guidelines, the IRB/IEC, European regulation 536/2014 for clinical studies (if applicable), and all other applicable local regulations.
- After reading the protocol, each Investigator will sign the protocol signature page and send a copy of the signed page to the Sponsor or representative (Appendix 12.5). The study will not start at any study center at which the Investigator has not signed the protocol.

Financial Disclosure

Investigators and sub-Investigators will provide the Sponsor with sufficient, accurate financial information as requested to allow the Sponsor to submit complete and accurate financial certification or disclosure statements to the appropriate regulatory authorities. Investigators are responsible for providing information on financial interests during the course of the study and for 1 year after completion of the study.

Insurance

Sponsor will provide insurance in accordance with local guidelines and requirements as a minimum for the subjects in this study. The terms of the insurance will be kept in the study files.

Informed Consent Process

- The Investigator or his/her representative will explain the nature of the study to the subject or his/her legally authorized representative and answer all questions regarding the study.
- Subjects must be informed that their participation is voluntary. Subjects or their legally authorized representative will be required to sign a statement of informed consent that meets the requirements of 21 CFR 50, local regulations, ICH guidelines, Health Insurance Portability and Accountability Act (HIPAA) requirements, where applicable, and the IRB/IEC or study center.
- The medical record must include a statement that written informed consent was obtained before the subject was entered in the study and the date the written consent was obtained. The authorized person obtaining the informed consent must also sign the ICF.
- Subjects must be reconsented to the most current version of the ICF(s) during their participation in the study.
- A copy of the ICF(s) must be provided to the subject or the subject’s legally authorized representative.

The Investigator or authorized designee will explain to each subject the objectives of the exploratory research. Subjects will be told that they are free to refuse to participate and may withdraw their consent at any time and for any reason during the storage period.

Data Protection

- Subjects will be assigned a unique identifier by the Sponsor. Any subject records or datasets that are transferred to the Sponsor will contain the identifier only; subject names or any information which would make the subject identifiable will not be transferred.
- The subject must be informed that his/her personal study-related data will be used by the Sponsor in accordance with local data protection law. The level of disclosure must also be explained to the subject.
- The subject must be informed that his/her medical records may be examined by Clinical Quality Assurance auditors or other authorized personnel appointed by the Sponsor, by appropriate IRB/IEC members, and by inspectors from regulatory authorities.
- The ICF will incorporate (or, in some cases, be accompanied by a separate document incorporating) wording that complies with relevant data protection and privacy legislation.
- The Sponsor or its representative will not provide individual genotype results to subjects, any insurance company, any employer, their family members, general physician, or any other third party, unless required to do so by law.
- Extra precautions are taken to preserve confidentiality and prevent genetic data being linked to the identity of the subject. In exceptional circumstances, however, certain individuals might see both the genetic data and the personal identifiers of a subject. For example, in the case of a medical emergency, the Sponsor or representative physician or an Investigator might know a subject’s identity and also have access to his or her genetic data. Also regulatory authorities may require access to the relevant files.

**Medical Monitor**

IQVIA

Dissemination of Clinical Study Data

The results of the study should be reported within 1 year from the end of the clinical study. Irrespective of the outcome, the Sponsor will submit to the EU database a summary of the results of the clinical study within 1 year from the end of the clinical study. It shall be accompanied by a summary written in a manner that is understandable to laypersons.

Study and Study Center Closure

The Sponsor designee reserves the right to close the study center or terminate the study at any time for any reason at the sole discretion of the Sponsor. Study centers will be closed upon study completion. A study center is considered closed when all required documents and study supplies have been collected and a study center closure visit has been performed.

The Investigator may initiate study center closure at any time, provided there is reasonable cause and sufficient notice is given in advance of the intended termination.

Reasons for the early closure of a study center by the Sponsor or Investigator may include but are not limited to:

- Failure of the Investigator to comply with the protocol, the requirements of the IRB/IEC or local health authorities, the Sponsor’s procedures, or GCP guidelines.
- Inadequate recruitment of subjects by the Investigator.
- Discontinuation of further study treatment development.

Publication Policy

The data generated by this study are confidential information of the Sponsor. The Sponsor will make the results of the study publicly available. The publication policy with respect to the Investigator and study center will be set forth in the Clinical Trial Agreement.

- The results of this study may be published or presented at scientific meetings. If this is foreseen, the Investigator agrees to submit all manuscripts or abstracts to the Sponsor before submission. This allows the Sponsor to protect proprietary information and to provide comments.
- The Sponsor will comply with the requirements for publication of study results. In accordance with standard editorial and ethical practice, the Sponsor will generally support publication of multicenter studies only in their entirety and not as individual study center data. In this case, a Coordinating Investigator will be designated by mutual agreement.
- Authorship will be determined by mutual agreement and in line with International Committee of Medical Journal Editors authorship requirements.

## Clinical Laboratory Tests

The tests detailed below will be performed by the local laboratory.

Additional tests may be performed at any time during the study as determined necessary by the investigator or required by local regulations.

- Hematology (hemoglobin, hematocrit, RBC indices)
- Thrombocyte count (platelets)
- White blood cell counts with differential (including neutrophils, eosinophils, basophils, lymphocytes and monocytes)
- Coagulation (international normalized ratio, activated partial thromboplastin time)
- LDH, Cardiac troponin, D-dimer and Ferritin
- Serum Chemistry:
- Electrolytes (sodium, potassium, chloride)
- Non-fasting glucose
- Blood urea nitrogen
- Creatinine
- Uric acid
- Phosphate
- Total calcium
- Cholesterol
- Triglycerides
- Albumin
- Total bilirubin
- Total protein
- γ-glutamyl transferase
- ALT
- AST
- Alkaline phosphatase
- Bicarbonate
- Haptoglobin
- Serology at screening: Hepatitis B, Hepatitis C, and HIV testing is to be completed at screening only.
- Urinalysis: specific gravity, pH, glucose, protein, blood, ketones, bilirubin, urobilinogen, nitrite, leukocyte esterase by dipstick, microscopic examination.

Unscheduled safety laboratory assessments may be conducted at any time during the study if clinically indicated.

## Contraceptive Guidance and Collection of Pregnancy Information

Definitions:

*Woman of Childbearing Potential (WOCBP)*

A woman is considered fertile following menarche and until becoming postmenopausal unless permanently sterile (see below).

*Women in the following categories are not considered WOCBP*

1. Premenarchal
2. Premenopausal female with 1 of the following:
   1. Documented hysterectomy.
   2. Documented bilateral salpingectomy.
   3. Documented bilateral oophorectomy.
   4. Note: Documentation can come from the study center personnel’s: review of the subject’s medical records, medical examination, or medical history interview.
3. Postmenopausal female:
   1. A postmenopausal state is defined as no menses for 12 months without an alternative medical cause. A high follicle stimulating hormone (FSH) level in the postmenopausal range may be used to confirm a postmenopausal state in women not using hormonal contraception or hormonal replacement therapy (HRT). However, in the absence of 12 months of amenorrhea, a single FSH measurement is insufficient.
   2. Females on HRT and whose menopausal status is in doubt will be required to use 1 of the nonestrogen hormonal highly effective contraception methods if they wish to continue their HRT during the study. Otherwise, they must discontinue HRT to allow confirmation of postmenopausal status before study enrollment.

Contraception Guidance

*Male* *subjects*

- Male subjects with female partners of childbearing potential are eligible to participate if they agree to ONE of the following during the protocol-defined time frame in Section 5.1:
  - Are abstinent from penile-vaginal intercourse as their usual and preferred lifestyle (abstinent on a long-term and persistent basis) and agree to remain abstinent.
  - Agree to use a male condom plus partner use of a contraceptive method with a failure rate of <1% per year when having penile-vaginal intercourse with a WOCBP who is not currently pregnant.
- In addition, male subjects must refrain from donating sperm for the duration of the study and for 6 months after the last dose of study treatment.
- Male subjects with a pregnant or breastfeeding partner must agree to remain abstinent from penile‑vaginal intercourse or use a male condom during each episode of penile penetration during the protocol-defined time frame.

*Female subjects*

Female subjects of childbearing potential are eligible to participate if they agree to use a highly effective method of contraception consistently and correctly as described in the table below.

Highly Effective Contraceptive Methods

| Highly Effective Contraceptive Methods That Are User Dependent ^a^  *Failure rate of <1% per year when used consistently and correctly.* |
| --- |
| Combined (estrogen and progestogen containing) hormonal contraception associated with inhibition of ovulation^b^   - Oral. - Intravaginal. - Transdermal. |
| Progestogen only hormonal contraception associated with inhibition of ovulation   - Oral. - Injectable. |
| Highly Effective Methods That Are User Independent ^a^ |
| Implantable progestogen only hormonal contraception associated with inhibition of ovulation^b^   - Intrauterine device (IUD). - Intrauterine hormone-releasing system (IUS).   Bilateral tubal occlusion. |
| **Vasectomized partner**  *A vasectomized partner is a highly effective birth control method provided that the partner is the sole male sexual partner of the WOCBP and the absence of sperm has been confirmed. If not, an additional highly effective method of contraception should be used.* |
| **Sexual abstinence**  *Sexual abstinence is considered a highly effective method only if defined as refraining from heterosexual intercourse during the entire period of risk associated with the study treatment. The reliability of sexual abstinence needs to be evaluated in relation to the duration of the study and the preferred and usual lifestyle of the subject.* |
| NOTES:  ^a^ Typical use failure rates may differ from those when used consistently and correctly. Use should be consistent with local regulations regarding the use of contraceptive methods for subjects participating in clinical studies.  ^b^ Hormonal contraception may be susceptible to interaction with the study treatment, which may reduce the efficacy of the contraceptive method. In this case, 2 highly effective methods of contraception should be utilized during the treatment period and for at least 130 days, for genotoxic products, an additional 30 days, corresponding to time needed to eliminate study treatment plus 30 days for study treatments with genotoxic potential, after the last dose of study treatment. |

Pregnancy Testing:

- A WOCBP should only be included after a confirmed menstrual period and a negative highly sensitive serum pregnancy test.
- Additional pregnancy testing is not required.

Collection of Pregnancy Information

*Male subjects with partners who become pregnant*

- The investigator will attempt to collect pregnancy information on any male subject’s female partner who becomes pregnant while the male subject is in this study. This applies only to male subjects who receive meplazumab.
- After obtaining the necessary signed informed consent from the pregnant female partner directly, the investigator will record pregnancy information on the appropriate form and submit it to the Sponsor within 24 hours of learning of the partner’s pregnancy. The female partner will also be followed to determine the outcome of the pregnancy. Information on the status of the mother and child will be forwarded to the Sponsor. Generally, the follow-up will be no longer than 6 to 8 weeks following the estimated delivery date. Any termination of the pregnancy will be reported regardless of fetal status (presence or absence of anomalies) or indication for the procedure.

*Female Subjects who become pregnant*

- The investigator will collect pregnancy information on any female subject who becomes pregnant while participating in this study. Information will be recorded on the appropriate form and submitted to the Sponsor within 24 hours of learning of a subject’s pregnancy. The subject will be followed to determine the outcome of the pregnancy. The investigator will collect follow-up information on the subject and the neonate and the information will be forwarded to the Sponsor. Generally, follow-up will not be required for longer than 6 to 8 weeks beyond the estimated delivery date. Any termination of pregnancy will be reported, regardless of fetal status (presence or absence of anomalies) or indication for the procedure.
- While pregnancy itself is not considered to be an AE or SAE, any pregnancy complication or elective termination of a pregnancy will be reported as an AE or SAE. A spontaneous abortion is always considered to be an SAE and will be reported as such.
- Any poststudy pregnancy related SAE considered reasonably related to the study treatment by the investigator will be reported to the Sponsor as described in Section 8.3.6. While the investigator is not obligated to actively seek this information in former subjects, he or she may learn of an SAE through spontaneous reporting.
- Any female subject who becomes pregnant while participating in the study will discontinue study treatment or be withdrawn from the study.

Continuation of study treatment may only be allowed if either of the following criteria is met:

The study treatment has an approved label that indicates it can be used safely in pregnant females.

OR

All of the following apply:

- The subject has a high mortality disease.
- The investigator determines the subject is benefitting from study participation and there is no other reasonable treatment for her.
- The Sponsor and the relevant IRB/IEC give written approval.
- The subject gives signed informed consent.
- The investigator agrees to monitor the outcome of the pregnancy and the status of the subject and her offspring.
- The protocol is amended to allow such participation on a case-by-case basis, if such participation is not already addressed in the protocol.

## Signature of Investigator

PROTOCOL TITLE: A Multicenter, Seamless, Randomized, Third-Party-Blind Clinical Trial to Evaluate the Safety and Efficacy of Meplazumab in Addition to Standard of Care for the Treatment of COVID‑19 in Hospitalized Adults

PROTOCOL NO: MPZ-II-02

VERSION: Amendment 3

This protocol is a confidential communication of Jiangsu Pacific Meinuoke Biopharmaceutical Co., Ltd. (PMBP). I confirm that I have read this protocol, I understand it, and I will work according to this protocol. I will also work consistently with the ethical principles that have their origin in the Declaration of Helsinki and that are consistent with Good Clinical Practices and the applicable laws and regulations. Acceptance of this document constitutes my agreement that no unpublished information contained herein will be published or disclosed without prior written approval from the Sponsor.

Instructions to the investigator: Please SIGN and DATE this signature page. PRINT your name, title, and the name of the study center in which the study will be conducted. Return the signed copy to the CRO for forwarding to Jiangsu Pacific Meinuoke Biopharmaceutical Co., Ltd. (PMBP).

I have read this protocol in its entirety and agree to conduct the study accordingly:

Signature of Investigator: __________________________ Date: ________

Printed Name: __________________________

Investigator Title: __________________________

Name/Address of Center: __________________________

___________________________

___________________________

## Administrative Structure

| **Sponsor** | Jiangsu Pacific Meinuoke Biopharmaceutical Co., Ltd. (“PMBP”), having a place of business at No. 128 W Hehai Rd, Xinbei District, Changzhou, Jiangsu Province, China |  |
| --- | --- | --- |
| **Sponsor’s Authorized Representative** | Xiaochun Chen, Chief Executive Officer |  |
| **Contract Research Organization (CRO)** | | IQVIA |
| **CRO’s Project Manager** | Peipei Yang | |
| **Protocol Author** | IQVIA | |
