# Supplementary material for: Meplazumab in hospitalized adults with severe COVID-19 (DEFLECT): a multicenter, seamless phase 2/3, randomized, third-party double-blind clinical trial
Source: Signal Transduct Target Ther. 2023 Jan 30;8:46. doi: 10.1038/s41392-023-01323-9 (PMC9885411; doi:10.1038/s41392-023-01323-9)
Supplement: Supplementary file 3 — Sigtrans_Supplementary_Note_2 [file 41392_2023_1323_MOESM3_ESM.docx]

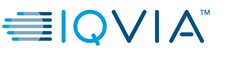
Jiangsu Pacific Meinuoke Biopharmaceutical Co., Ltd. (PMBP)

Protocol MPZ-II-02

Statistical Analysis Plan Page 1 of 82

**STATISTICAL ANALYSIS PLAN**

## MPZ-II-02

**A MULTICENTER, SEAMLESS, RANDOMIZED, THIRD-PARTY-BLIND, CLINICAL TRIAL TO EVALUATE THE SAFETY AND EFFICACY OF MEPLAZUMAB IN ADDITION TO STANDARD OF CARE FOR THE TREATMENT OF COVID-19 IN HOSPITALIZED ADULTS**

**AUTHOR: YUEH WANG, YANG TENG**

**VERSION NUMBER AND DATE: V2.0, 13DEC2021**

Document: \\ieedc-vnasc01\BIOSdata\Jiangsu_PMBP\Meplazumab\SZA62090\Biostatistics\Documentation\SAP\

| Author: Yueh Wang, Yang Teng | Version Number: Version Date: | 2.0  13-Dec-2021 |
| --- | --- | --- |
| Template No.: CS_TP_BS016 Revision 6 Effective Date: 02Dec2019 | Reference: | CS_WI_BS005 |

Copyright © 2009, 2010, 2012, 2016, 2018, 2019 IQVIA. All rights reserved. The contents of this document are confidential and proprietary to IQVIA Holdings Inc. and its subsidiaries. Unauthorized use, disclosure or reproduction is strictly prohibited.


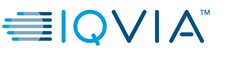
Jiangsu Pacific Meinuoke Biopharmaceutical Co., Ltd. (PMBP)

Protocol MPZ-II-02

Statistical Analysis Plan Page 4 of 82

**MODIFICATION HISTORY**

| **Unique Identifier for this Version** | **Date of the Document Version** | **Author** | **Significant Changes from Previous Version** |
| --- | --- | --- | --- |
| 0.1 | 25Sep2020 | Ivy Yeh | Not Applicable – First Version |
| 0.2 | 03Dec2020 | Ivy Yeh, Andrew Ralya | Changes after EMA/IND Submission:   1. PK and PD analysis are included, and a separate PK SAP will not be generated. 2. Clarify the secondary endpoints for Stage 1. 3. Section 6.4: Add the visit window rule for response rate analysis. 4. Section 7.1: Add the sample size calculation method. 5. Section 7.8: Add unblinding data handlings. 6. Section 16.1.3: Update censoring rule for time to sustained clinical improvement or live discharge on Day 29 7. Section 16.4: Add more details for 1st IA 8. Update per protocol amendment |
| 0.3 | 26Mar2021 | Summer Hsieh | Change while preparing dry run outputs:   1. Section 6.2: Add more details for the definition of baseline. 2. Section 10: Height is not collected in CRF, delete ‘Height’ for demographic. 3. Update MedDRA and WHO Drug version |

Document: \\ieedc-vnasc01\BIOSdata\Jiangsu_PMBP\Meplazumab\SZA62090\Biostatistics\Documentation\SAP\

| Author: Yueh Wang, Yang Teng | Version Number: Version Date: | 2.0  13-Dec-2021 |
| --- | --- | --- |
| Template No.: CS_TP_BS016 Revision 6 Effective Date: 02Dec2019 | Reference: | CS_WI_BS005 |

Copyright © 2009, 2010, 2012, 2016, 2018, 2019 IQVIA. All rights reserved. The contents of this document are confidential and proprietary to IQVIA Holdings Inc. and its subsidiaries. Unauthorized use, disclosure or reproduction is strictly prohibited.


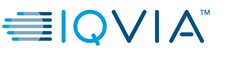
Jiangsu Pacific Meinuoke Biopharmaceutical Co., Ltd. (PMBP)

Protocol MPZ-II-02

Statistical Analysis Plan Page 5 of 82

| 1.0 | 30Jun2021 | Summer Hsieh | Update the protocol version to Amendment 2, date 11Jun2021. |
| --- | --- | --- | --- |
| 2.0 | 13Dec2021 | Yueh Wang | 1. Section 1: Updated the protocol version to Amendment 4, date 12Nov2021. 2. Section 3.1 and Figure A: Updated stage 1 sample size from 216 to approximately 168. 3. Section 7.1: Updated stage 1 sample size from 216 to approximately 168, and corresponding power from 90% to 81%. 4. Section 7.8: Per FDA suggestion, updated unblinding handling from removing accidental unblinded subjects from the ITT analysis set to keeping accidental unblinded subjects in the ITT analysis set. |

Document: \\ieedc-vnasc01\BIOSdata\Jiangsu_PMBP\Meplazumab\SZA62090\Biostatistics\Documentation\SAP\

| Author: Yueh Wang, Yang Teng | Version Number: Version Date: | 2.0  13-Dec-2021 |
| --- | --- | --- |
| Template No.: CS_TP_BS016 Revision 6 Effective Date: 02Dec2019 | Reference: | CS_WI_BS005 |

Copyright © 2009, 2010, 2012, 2016, 2018, 2019 IQVIA. All rights reserved. The contents of this document are confidential and proprietary to IQVIA Holdings Inc. and its subsidiaries. Unauthorized use, disclosure or reproduction is strictly prohibited.


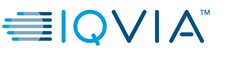
Jiangsu Pacific Meinuoke Biopharmaceutical Co., Ltd. (PMBP)

Protocol MPZ-II-02

Statistical Analysis Plan Page 6 of 82

**TABLE OF CONTENTS**

## INTRODUCTION 12

## STUDY OBJECTIVES 12

###### Primary Objective 12

###### Secondary Objectives 12

###### Exploratory Objectives 13

## STUDY DESIGN 13

###### General Description 13

###### Schedule of Activities 17

###### Changes to Analyses from Protocol 17

## PLANNED ANALYSES 18

###### Interim Analyses 18

###### Final Analysis 19

###### Independent Data Monitoring Committee 19

## ANALYSIS SETS 20

###### Enrolled Analysis Set (ENR) 20

###### Intent-to-Treat Analysis Set (ITT) 20

###### Safety Analysis Set (SAF) 20

###### Pharmacokinetic Analysis Set (PKS) 20

###### Pharmacokinetic parameter analysis set (PKPS) 21

###### Pharmacodynamic Analysis Set (PDS) 21

Document: \\ieedc-vnasc01\BIOSdata\Jiangsu_PMBP\Meplazumab\SZA62090\Biostatistics\Documentation\SAP\

| Author: Yueh Wang, Yang Teng | Version Number: Version Date: | 2.0  13-Dec-2021 |
| --- | --- | --- |
| Template No.: CS_TP_BS016 Revision 6 Effective Date: 02Dec2019 | Reference: | CS_WI_BS005 |

Copyright © 2009, 2010, 2012, 2016, 2018, 2019 IQVIA. All rights reserved. The contents of this document are confidential and proprietary to IQVIA Holdings Inc. and its subsidiaries. Unauthorized use, disclosure or reproduction is strictly prohibited.


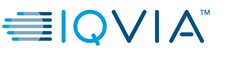
Jiangsu Pacific Meinuoke Biopharmaceutical Co., Ltd. (PMBP)

Protocol MPZ-II-02

Statistical Analysis Plan Page 7 of 82

###### Process for Analysis Set Assignment 21

## GENERAL CONSIDERATIONS 22

###### Reference Start Date and Study Day 22

###### Baseline 22

###### Unscheduled Visits, Retests, and Early Termination Data 23

###### Windowing Conventions 23

###### Common Calculations 24

## STATISTICAL CONSIDERATIONS 24

###### Sample Size Calculation 24

###### Missing data 26

###### Statistical Tests 26

###### Multiple Comparisons/ Multiplicity 27

###### Multicenter Studies 27

###### Adjustments for Covariates and Factors to be Included in Analyses 27

###### Examination of Subgroups 28

###### Unblinding Handling 28

###### Software Version 29

## OUTPUT PRESENTATIONS 29

## DISPOSITION AND WITHDRAWALS 29

###### Disposition 29

###### Protocol Deviations 30

Document: \\ieedc-vnasc01\BIOSdata\Jiangsu_PMBP\Meplazumab\SZA62090\Biostatistics\Documentation\SAP\

| Author: Yueh Wang, Yang Teng | Version Number: Version Date: | 2.0  13-Dec-2021 |
| --- | --- | --- |
| Template No.: CS_TP_BS016 Revision 6 Effective Date: 02Dec2019 | Reference: | CS_WI_BS005 |

Copyright © 2009, 2010, 2012, 2016, 2018, 2019 IQVIA. All rights reserved. The contents of this document are confidential and proprietary to IQVIA Holdings Inc. and its subsidiaries. Unauthorized use, disclosure or reproduction is strictly prohibited.


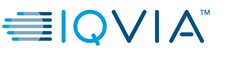
Jiangsu Pacific Meinuoke Biopharmaceutical Co., Ltd. (PMBP)

Protocol MPZ-II-02

Statistical Analysis Plan Page 8 of 82

## DEMOGRAPHIC AND OTHER BASELINE CHARACTERISTICS 30

###### Derivations 31

## MEDICAL HISTORY 31

## DISEASE HISTORY 32

###### Derivations 33

## MEDICATIONS AND PROCEDURES 33

## EXPOSURE TO STUDY DRUG 34

###### Derivations 35

## COMPLIANCE WITH STUDY DRUG 35

## EFFICACY ENDPOINTS 35

###### Primary Efficacy 35

- - 1. Primary Efficacy Endpoint 35
    2. Missing Data Imputation Method for Primary Efficacy Endpoint 36
    3. Primary Analysis of Primary Efficacy Endpoint 37
    4. Sensitivity Analyses for Primary Efficacy Endpoint 39
    5. Supplementary Analyses for Primary Efficacy Endpoint 40

###### Secondary Efficacy 41

- - 1. Secondary Efficacy Endpoints & Derivations 41
       1. Response rate at Day 2, 8, 15 41
       2. Proportion of subjects alive and discharge without supplemental oxygen at Day 15 and Day 57 ...41 16.2.1.3. Mortality at Days 15 and 57 41
       3. Time from treatment start date to death 42
       4. Time to sustained recovery (Days) 42
       5. Duration (days) of oxygen use and oxygen-free days 42
       6. Duration (days) of mechanical ventilation and mechanical ventilation-free days 43
       7. Incidence of new mechanical ventilation use and duration (days) of new mechanical ventilation use 43
       8. Incidence of new mechanical ventilation use after mechanical ventilation extubation period of 24 hours 44
       9. Incidence of rehospitalization following hospital discharge 44

Document: \\ieedc-vnasc01\BIOSdata\Jiangsu_PMBP\Meplazumab\SZA62090\Biostatistics\Documentation\SAP\

| Author: Yueh Wang, Yang Teng | Version Number: Version Date: | 2.0  13-Dec-2021 |
| --- | --- | --- |
| Template No.: CS_TP_BS016 Revision 6 Effective Date: 02Dec2019 | Reference: | CS_WI_BS005 |

Copyright © 2009, 2010, 2012, 2016, 2018, 2019 IQVIA. All rights reserved. The contents of this document are confidential and proprietary to IQVIA Holdings Inc. and its subsidiaries. Unauthorized use, disclosure or reproduction is strictly prohibited.


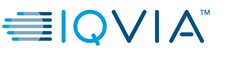
Jiangsu Pacific Meinuoke Biopharmaceutical Co., Ltd. (PMBP)

Protocol MPZ-II-02

Statistical Analysis Plan Page 9 of 82

- - - 1. Duration (days) of ICU and hospitalization 44
    1. Missing Data Imputation Method for Secondary Efficacy Endpoints 44
    2. Primary Analysis of Secondary Efficacy Endpoints 45

Considering no inferential interpretation will be made by secondary efficacy endpoints, statistical testing will not performed for all the secondary efficacy endpoints 45

- - - 1. ‘Time-to-event’ Secondary Efficacy Endpoints 45
      2. ‘Binary endpoints’ Secondary Efficacy Endpoints= 45
      3. Other Secondary Efficacy Endpoints 45
    1. Sensitivity Analyses for Secondary Efficacy Endpoints 45
    2. Supplementary Analyses for Secondary Efficacy Endpoints 45

###### Exploratory Efficacy 46

- - 1. Exploratory efficacy endpoints & Derivations 46
       1. Ranked trajectory over 29 days 46
    2. Analysis of Exploratory Efficacy Endpoints 46
       1. Analysis of ranked trajectory over 29 days 46

###### Interim Analysis 46

- - 1. Interim Analyis for Sample Size Re-Assessment 47
    2. Interm Analysis for dose selection and efficacy endpoint selection (Stage 1) 47

## SAFETY ENDPOINTS 49

###### Adverse Events 49

- - 1. All TEAEs 49
       1. Severity 49
       2. Relationship to Study Drug 50
       3. Relationship to Non-Study Treatment 50
       4. Relationship to Study Procedure 50
       5. Action Taken with Study Treatment 51
       6. Outcome 51
    2. Adverse Events with an Outcome of Death 51
    3. Serious Adverse Events 51
    4. TEAEs Leading to Permanent Discontinuation of Study Drug 51
    5. Adverse Events of Special Interest 52

###### Deaths 52

###### Laboratory Evaluations 53

- - 1. CTCAE Toxicity Grades 54
    2. Laboratory Normal Ranges 54
    3. Serology and Urinalysis at Screening 54

###### Covid-19 Virological Load 54

###### Vital Signs 55

Document: \\ieedc-vnasc01\BIOSdata\Jiangsu_PMBP\Meplazumab\SZA62090\Biostatistics\Documentation\SAP\

| Author: Yueh Wang, Yang Teng | Version Number: Version Date: | 2.0  13-Dec-2021 |
| --- | --- | --- |
| Template No.: CS_TP_BS016 Revision 6 Effective Date: 02Dec2019 | Reference: | CS_WI_BS005 |

Copyright © 2009, 2010, 2012, 2016, 2018, 2019 IQVIA. All rights reserved. The contents of this document are confidential and proprietary to IQVIA Holdings Inc. and its subsidiaries. Unauthorized use, disclosure or reproduction is strictly prohibited.


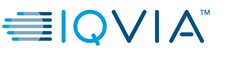
Jiangsu Pacific Meinuoke Biopharmaceutical Co., Ltd. (PMBP)

Protocol MPZ-II-02

Statistical Analysis Plan Page 10 of 82

- - 1. Vital Signs Markedly Abnormal Criteria 56

###### ECG Evaluations 56

- - 1. ECG Markedly Abnormal Criteria 57

###### Other Safety Assessments 58

- - 1. General Physical Examination 58
    2. Radiographic lung image 58
    3. Antidrug - Antibodies 58
    4. The National Early Warning score (NEWS2) 59
    5. Pregnancy Test 59
    6. COVID Symptom Assessment 60

## PHARMACOKINETIC AND PHARMACODYNAMIC ENDPOINTS 60

###### Pharmacokinetics 61

- - 1. Meplazumab Serum and BLood Cell Concentration Data 62
    2. Meplazumab Serum and Blood Pharmacokinetic Parameters 63

###### Pharmacodynamics 66

###### Exposure Response Correlations 67

## DATA NOT SUMMARIZED OR PRESENTED 67

## REFERENCES 68

## APPENDIX 1. PARTIAL DATE CONVENTIONS 70

###### Algorithm for Treatment Emergence of Adverse Events 70

###### Algorithm for Prior / Concomitant Medications 70

## APPENDIX 2. PROGRAMMING CONVENTIONS FOR OUTPUTS 72

###### Dates & Times 72

###### Spelling Format 72

###### Paper Size, Orientation, and Margins 72

###### Fonts 72

Document: \\ieedc-vnasc01\BIOSdata\Jiangsu_PMBP\Meplazumab\SZA62090\Biostatistics\Documentation\SAP\

| Author: Yueh Wang, Yang Teng | Version Number: Version Date: | 2.0  13-Dec-2021 |
| --- | --- | --- |
| Template No.: CS_TP_BS016 Revision 6 Effective Date: 02Dec2019 | Reference: | CS_WI_BS005 |

Copyright © 2009, 2010, 2012, 2016, 2018, 2019 IQVIA. All rights reserved. The contents of this document are confidential and proprietary to IQVIA Holdings Inc. and its subsidiaries. Unauthorized use, disclosure or reproduction is strictly prohibited.


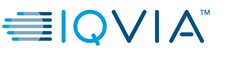
Jiangsu Pacific Meinuoke Biopharmaceutical Co., Ltd. (PMBP)

Protocol MPZ-II-02

Statistical Analysis Plan Page 11 of 82

###### Presentation of Treatment Groups 72

###### Presentation of Nominal Visits 73

###### Descriptive Statistics 73

###### Percentages 74

###### p-values 74

###### Listings 74

## APPENDIX 3. CTCAE TOXICITY GRADE, VERSION 5.0 75

Document: \\ieedc-vnasc01\BIOSdata\Jiangsu_PMBP\Meplazumab\SZA62090\Biostatistics\Documentation\SAP\

| Author: Yueh Wang, Yang Teng | Version Number: Version Date: | 2.0  13-Dec-2021 |
| --- | --- | --- |
| Template No.: CS_TP_BS016 Revision 6 Effective Date: 02Dec2019 | Reference: | CS_WI_BS005 |

Copyright © 2009, 2010, 2012, 2016, 2018, 2019 IQVIA. All rights reserved. The contents of this document are confidential and proprietary to IQVIA Holdings Inc. and its subsidiaries. Unauthorized use, disclosure or reproduction is strictly prohibited.


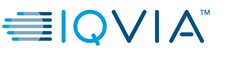
Jiangsu Pacific Meinuoke Biopharmaceutical Co., Ltd. (PMBP)

Protocol MPZ-II-02

Statistical Analysis Plan Page 12 of 82

1. **INTRODUCTION**

This statistical analysis plan (SAP) describes the rules and conventions to be used in the presentation and analysis of efficacy and safety data for protocol MPZ-II-02. It describes the data to be summarized and analyzed, including specifics of the statistical analyses to be performed. The detailed analyses for the pharmacokinetic (PK), and pharmacodynamic (PD) data will also be described in the analysis plan, while some of the exploratory endpoints may be reported separately from the clinical study report per sponsor’s agreement.

This SAP is based on protocol amendment 4 version, dated 12Nov2021.

1. **STUDY OBJECTIVES**
   1. **PRIMARY OBJECTIVE**

**Stage 1:**

To evaluate the efficacy of 3 selected doses of meplazumab plus Standard of Care (SoC) compared to control plus SoC in subjects hospitalized with coronavirus disease 2019 (COVID-19) infection.

**Stage 2:**

To evaluate the efficacy of the selected dose of meplazumab plus SoC (based on adaptation from Stage 1) compared to control plus SoC, in subjects hospitalized with COVID-19 infection.

- 1. **SECONDARY OBJECTIVES**

**Stage 1:**

- To evaluate the safety of meplazumab as add-on therapy to SoC in subjects hospitalized with COVID-19
- To assess other potential primary efficacy endpoints for Stage 2, including time to sustained clinical improvement or live discharge from hospital by Day 29, mortality at Day 29, proportion of subjects alive and discharged without supplemental oxygen at Day 29

**Stage 2:**

Document: \\ieedc-vnasc01\BIOSdata\Jiangsu_PMBP\Meplazumab\SZA62090\Biostatistics\Documentation\SAP\

| Author: Yueh Wang, Yang Teng | Version Number: Version Date: | 2.0  13-Dec-2021 |
| --- | --- | --- |
| Template No.: CS_TP_BS016 Revision 6 Effective Date: 02Dec2019 | Reference: | CS_WI_BS005 |

Copyright © 2009, 2010, 2012, 2016, 2018, 2019 IQVIA. All rights reserved. The contents of this document are confidential and proprietary to IQVIA Holdings Inc. and its subsidiaries. Unauthorized use, disclosure or reproduction is strictly prohibited.


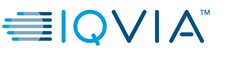
Jiangsu Pacific Meinuoke Biopharmaceutical Co., Ltd. (PMBP)

Protocol MPZ-II-02

Statistical Analysis Plan Page 13 of 82

The secondary objectives are:

- To evaluate response rate
- To evaluate live discharge
- To evaluate the safety of meplazumab as add-on therapy to SoC in subjects with COVID-19
- To evaluate overall mortality
- To evaluate clinical recovery of COVID-19 disease
- To evaluate the number of oxygen-free days
- To evaluate ventilator-free days, incidence, and duration of new mechanical ventilation use
- To evaluate clinical relapse of COVID-19
- To evaluate ICU and hospitalization length
  1. **EXPLORATORY OBJECTIVES**

The exploratory objectives for both Stage 1 and Stage 2 are:

- To evaluate pharmacokinetic (PK) exposure to meplazumab
- To evaluate pharmacodynamic (PD) response to administration of meplazumab
- To explore potential exposure-response relationships The exploratory objectives for Stage 2 only is:
- To evaluate the efficacy of the selected dose using a composite ranked outcome trajectory score

1. **STUDY DESIGN**
   1. **GENERAL DESCRIPTION**

This is a multi-center, seamless, randomized, third-party-blind, study to evaluate the safety and efficacy of

Document: \\ieedc-vnasc01\BIOSdata\Jiangsu_PMBP\Meplazumab\SZA62090\Biostatistics\Documentation\SAP\

| Author: Yueh Wang, Yang Teng | Version Number: Version Date: | 2.0  13-Dec-2021 |
| --- | --- | --- |
| Template No.: CS_TP_BS016 Revision 6 Effective Date: 02Dec2019 | Reference: | CS_WI_BS005 |

Copyright © 2009, 2010, 2012, 2016, 2018, 2019 IQVIA. All rights reserved. The contents of this document are confidential and proprietary to IQVIA Holdings Inc. and its subsidiaries. Unauthorized use, disclosure or reproduction is strictly prohibited.


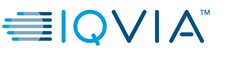
Jiangsu Pacific Meinuoke Biopharmaceutical Co., Ltd. (PMBP)

Protocol MPZ-II-02

Statistical Analysis Plan Page 14 of 82

meplazumab for the treatment of COVID-19 in hospitalized adults (≥18 years). Neither the subject nor the investigator shall be aware of whether the subject receives the study drug or placebo, as the study drug/placebo is prepared by an unblinded third party (eg, a pharmacist or nurse) and administered by an authorized blinded site staff.

Enrollment of subjects will be stopped once the total number of planned subjects have completed the Stage 1 Day 29 visit procedures. Once the interim analysis of Stage 1 study data is complete and the Independent Data Monitoring Committee (IDMC) has recommended the meplazumab dose that is safe and effective to carry forward into Stage 2, the study will resume subject enrollment. A summary of the key Stage 1 interim analysis results will be sent to the relevant Health Authorities involved, if requested.

For the duration of the hospitalization, all study subjects will receive SoC commensurate with critical care and hospitalization for COVID-19, based on the medical judgment of the study investigator.

Stage 1, the dose-finding period of the study, will compare 3 meplazumab treatment arms to control, in addition to SoC, to determine the safe and efficacious dose to investigate in Stage 2, the confirmatory period of the study.

Response rate (number and %) by treatment arm at Stage 1 on Day 29 will be used to select the dose to implement in Stage 2 of the study. Stage 1 data will additionally be used to determine optimal study endpoints, and the number of subjects to enroll into Stage 2 of the study. Stage 2 of the study is intended to provide a robust database on the selected dose to fully evaluate disease outcomes, including severe AEs, overall AEs, disease-related co-infection complications (eg, pneumonia, septic shock) and overall mortality.

Hospitalized subjects with laboratory-confirmed SARS-CoV-2 infection, with clinical status as defined as a Grade 3 (hospitalized, requiring supplemental oxygen) or Grade 4 (hospitalized, on non-invasive ventilation or high flow oxygen devices) on the 6-Point Ordinal Scale will be enrolled for both Stage 1 and Stage 2. Randomization will be stratified by severity, and by age group (age <65 years or ≥65 years). Additional stratification factors such as region and/or receipt status of remdesivir at baseline may be considered for Stage 2 based on the data evaluated in Stage 1. Randomization sequence will be created separately for Stage 1 and Stage 2 with permuted block randomization.

For Stage 1, approximately 168 subjects will be randomized and allocated 1:1:1:1 to receive either low dose, medium dose or high dose of meplazumab, or control. An interim analysis will be conducted to select the optimal dose of meplazumab compared with the control group based on response rates of clinical improvement at Day 29.

For Stage 2, 240 more subjects will be randomized and allocated 2:1 (160:80) to receive the optimal meplazumab dose determined after Stage 1, or control. At interim analysis, primary endpoint, sample size calculation for Stage 2 will be re-evaluated based on the observed outcomes at Stage 1 and will be capped at 300 subjects total.

Study duration for each subject will be 84±7 days from randomization in each Stage, including screening period

Document: \\ieedc-vnasc01\BIOSdata\Jiangsu_PMBP\Meplazumab\SZA62090\Biostatistics\Documentation\SAP\

| Author: Yueh Wang, Yang Teng | Version Number: Version Date: | 2.0  13-Dec-2021 |
| --- | --- | --- |
| Template No.: CS_TP_BS016 Revision 6 Effective Date: 02Dec2019 | Reference: | CS_WI_BS005 |

Copyright © 2009, 2010, 2012, 2016, 2018, 2019 IQVIA. All rights reserved. The contents of this document are confidential and proprietary to IQVIA Holdings Inc. and its subsidiaries. Unauthorized use, disclosure or reproduction is strictly prohibited.


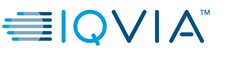
Jiangsu Pacific Meinuoke Biopharmaceutical Co., Ltd. (PMBP)

Protocol MPZ-II-02

Statistical Analysis Plan Page 15 of 82

follow by 28 days core study period, a follow-up visit at Day 57 and the end of study visit at Day 84. The study schema can be found in Figure A below:

Document: \\ieedc-vnasc01\BIOSdata\Jiangsu_PMBP\Meplazumab\SZA62090\Biostatistics\Documentation\SAP\

| Author: Yueh Wang, Yang Teng | Version Number: Version Date: | 2.0  13-Dec-2021 |
| --- | --- | --- |
| Template No.: CS_TP_BS016 Revision 6 Effective Date: 02Dec2019 | Reference: | CS_WI_BS005 |

Copyright © 2009, 2010, 2012, 2016, 2018, 2019 IQVIA. All rights reserved. The contents of this document are confidential and proprietary to IQVIA Holdings Inc. and its subsidiaries. Unauthorized use, disclosure or reproduction is strictly prohibited.


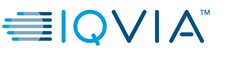
Jiangsu Pacific Meinuoke Biopharmaceutical Co., Ltd. (PMBP)

Protocol MPZ-II-02

Statistical Analysis Plan Page 16 of 82

**Figure A. Study Schema**


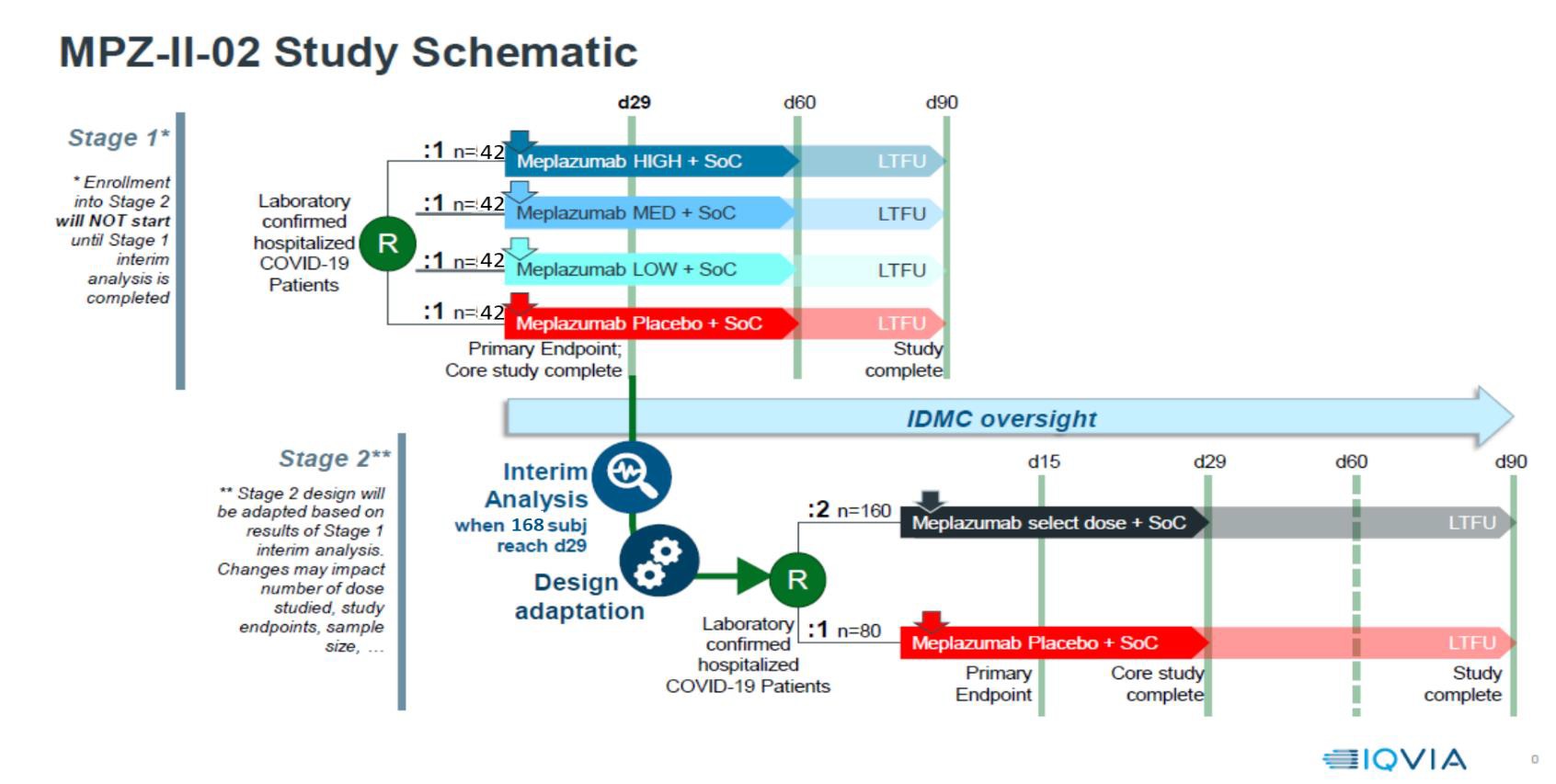


Abbreviations: d = day; IDMC = independent data monitoring committee; LTFU = long-term follow-up; n = number; SoC = standard of care; subj = subject.

Document: \\ieedc-vnasc01\BIOSdata\Jiangsu_PMBP\Meplazumab\SZA62090\Biostatistics\Documentation\SAP\

| Author: Yueh Wang, Yang Teng | Version Number: Version Date: | 2.0  13-Dec-2021 |
| --- | --- | --- |
| Template No.: CS_TP_BS016 Revision 6 Effective Date: 02Dec2019 | Reference: | CS_WI_BS005 |

Copyright © 2009, 2010, 2012, 2016, 2018, 2019 IQVIA. All rights reserved. The contents of this document are confidential and proprietary to IQVIA Holdings Inc. and its subsidiaries. Unauthorized use, disclosure or reproduction is strictly prohibited.


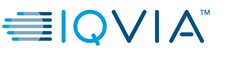
Jiangsu Pacific Meinuoke Biopharmaceutical Co., Ltd. (PMBP)

Protocol MPZ-II-02

Statistical Analysis Plan Page 17 of 82

In both Stages, subjects will receive IV infusion of meplazumab or control as specified, and the details can be found in Table 1 below:

**Table 1. Study Treatment Details**

| **Study Treatment Name:** | Meplazumab Low- Dose | Meplazumab Medium Dose | Meplazumab High- Dose | Control |
| --- | --- | --- | --- | --- |
| **Dosage Formulation:** | Solution for  intravenous infusion | Solution for  intravenous infusion | Solution for  intravenous infusion | Sterile normal saline (0.9%) for IV infusion |
| **Unit Dose** | 1st dose: 0.12 | 1st dose: 0.2 mg/kg | 1st dose: 0.3 mg/kg | 1st dose: control – |
| **Strength(s)/Dosage**  **Level(s):** | mg/kg – Day 1 | – Day 1 | – Day 1 | Day 1 |
|  | 2nd dose: control – | 2nd dose: 0.2 mg/kg | 2nd dose: 0.3 mg/kg | 2nd dose: control – |
|  | Day 8* | – Day 8 | – Day 8 | Day 8 |
| **Route of Administration** | Intravenous | Intravenous | Intravenous | Intravenous |
| **Dosing Instructions:** | 1-hour infusion,  100 mL | 1-hour infusion, 100 mL | 1-hour infusion,  100 mL | 1-hour infusion,  100 mL |

Abbreviations: kg= kilogram; mg = milligram; mL = milliliter.

* Sterile normal saline (0.9%) for IV infusion

Within this SAP, meplazumab placebo +SoC is referred to as the control.

- 1. **SCHEDULE OF ACTIVITIES**

Schedule of activities can be found in Section 1.3 of the protocol.

- 1. **CHANGES TO ANALYSES FROM PROTOCOL**

Not applicable.

Document: \\ieedc-vnasc01\BIOSdata\Jiangsu_PMBP\Meplazumab\SZA62090\Biostatistics\Documentation\SAP\

| Author: Yueh Wang, Yang Teng | Version Number: Version Date: | 2.0  13-Dec-2021 |
| --- | --- | --- |
| Template No.: CS_TP_BS016 Revision 6 Effective Date: 02Dec2019 | Reference: | CS_WI_BS005 |

Copyright © 2009, 2010, 2012, 2016, 2018, 2019 IQVIA. All rights reserved. The contents of this document are confidential and proprietary to IQVIA Holdings Inc. and its subsidiaries. Unauthorized use, disclosure or reproduction is strictly prohibited.


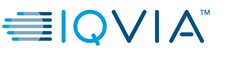
Jiangsu Pacific Meinuoke Biopharmaceutical Co., Ltd. (PMBP)

Protocol MPZ-II-02

Statistical Analysis Plan Page 18 of 82

1. **PLANNED ANALYSES**

The following analyses will be performed for the study:

- Interim analysis of Stage 1 data (will be reviewed by an Independent Data Monitoring Committee (IDMC))
- Final analysis

Efficacy for the interim analysis of Stage 2 will be determined and described in a later addition of this SAP. This will be described after the primary endpoint is selected after the interim analysis of Stage 1.

- 1. **INTERIM ANALYSES**

For Stage 1, efficacy and safety data collected up to Day 29 from the last subject randomization date will be included in the interim analysis (IA) report. The primary efficacy endpoint selection for Stage 2 will be determined by the IDMC (see Section 4.3) at the end of Stage 1 based on the data collected, which could depend on their clinical relevance and strength to detect the treatment difference between optimal dose and control. Pharmacokinetic and PD data will be included in the report only if available by the cutoff date.

The sample size for Stage 2 will be re-evaluated based on the data collected at Stage 1 and the selected primary endpoint. A futility analysis comparing the efficacy of the dose arms to control will be conducted after Stage 1 and will also be based on the Stage 1 data.

As part of the interim analysis of Stage 1, meplazumab concentrations and parameters (as available) will be listed and summarized by scheduled collection time and treatment for the Pharmacokinetic Analysis Set (PKS). Similarly, meplazumab PK parameters (as available) will be listed and summarized by scheduled collection time and treatment for the Pharmacokinetic Parameter Analysis Set (PKPS).

Stage 2 interim analysis for efficacy will be determined and described after the primary endpoint is selected at the end of Stage 1. An interim PK analysis during Stage 2 is not planned.

Derivations and definitions for the IAs of Stage 1 and Stage 2 reviewed by the IDMC will be based on those required for the final analysis contained in this analysis plan, unless deviations are stated within this SAP. The rules and conventions to be used in the analysis of efficacy data for the IA are described in Section 16.4, including specifics of the statistical analyses to be performed. The list of outputs provided with the set of output templates planned for the final analysis will highlight which of these outputs will also be provided for the IAs of Stage 1 and Stage 2.

Document: \\ieedc-vnasc01\BIOSdata\Jiangsu_PMBP\Meplazumab\SZA62090\Biostatistics\Documentation\SAP\

| Author: Yueh Wang, Yang Teng | Version Number: Version Date: | 2.0  13-Dec-2021 |
| --- | --- | --- |
| Template No.: CS_TP_BS016 Revision 6 Effective Date: 02Dec2019 | Reference: | CS_WI_BS005 |

Copyright © 2009, 2010, 2012, 2016, 2018, 2019 IQVIA. All rights reserved. The contents of this document are confidential and proprietary to IQVIA Holdings Inc. and its subsidiaries. Unauthorized use, disclosure or reproduction is strictly prohibited.


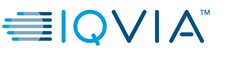
Jiangsu Pacific Meinuoke Biopharmaceutical Co., Ltd. (PMBP)

Protocol MPZ-II-02

Statistical Analysis Plan Page 19 of 82

- 1. **FINAL ANALYSIS**

All final, planned analyses identified in this SAP will be performed by IQVIA Biostatistics following sponsor authorization of this SAP, sponsor authorization of the analysis sets, database lock (DBL), and general study unblinding. Data collected at Stage 1 and Stage 2 will be both included in final analysis, while data of Stage 1 and Stage 2 won’t be combined but will be analyzed separately. Pharmacokinetic data for Stage 1 and Stage 2 may be combined for select analyses (see Section 18).

- 1. **INDEPENDENT DATA MONITORING COMMITTEE**

An Independent Data Monitoring Committee (IDMC) will be responsible for safeguarding the safety of subjects and for general oversight of the study conduct. The IDMC will have the following responsibilities:

- Review results of the interim analysis
- Review of interim safety data at regular intervals while subjects remain on study treatment
- Review of individual safety issues as requested by the Medical Monitor during the course of the study with the goal of recommending an appropriate course of action.

The operating principles, roles and responsibilities of the IDMC will be fully described in the IDMC Charter.

The data cut-off for each IDMC data review meeting will be determined based on the date of the IDMC data review meeting once these dates are established. All subjects who completed, discontinued or are ongoing in the study at the time of the data cut-off will be accounted for in the IDMC outputs. Data will be as clean as possible, but may not be fully cleaned and/or coded.

The IQVIA study team, including those responsible for creating the programs to produce the outputs for the IDMC data review meetings, will remain blinded. Once the programs have been produced by the IQVIA study team, these programs will be sent to an independent statistician, who will apply the randomization schedule and provide the IDMC members with a set of unblinded outputs, which will be described more in Section 16.4.

The interim PK analysis will be performed by an independent PK scientist with support of an independent statistical programmer responsible for the preparation of the analysis dataset used for the PK analysis.

The list of the unblinded personnel will be documented in the Unblinding Plan, which will be finalized before the data cut-off for the first IDMC data review meeting.

Document: \\ieedc-vnasc01\BIOSdata\Jiangsu_PMBP\Meplazumab\SZA62090\Biostatistics\Documentation\SAP\

| Author: Yueh Wang, Yang Teng | Version Number: Version Date: | 2.0  13-Dec-2021 |
| --- | --- | --- |
| Template No.: CS_TP_BS016 Revision 6 Effective Date: 02Dec2019 | Reference: | CS_WI_BS005 |

Copyright © 2009, 2010, 2012, 2016, 2018, 2019 IQVIA. All rights reserved. The contents of this document are confidential and proprietary to IQVIA Holdings Inc. and its subsidiaries. Unauthorized use, disclosure or reproduction is strictly prohibited.


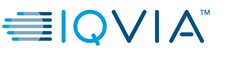
Jiangsu Pacific Meinuoke Biopharmaceutical Co., Ltd. (PMBP)

Protocol MPZ-II-02

Statistical Analysis Plan Page 20 of 82

1. **ANALYSIS SETS**

The definition of each analysis set described below will be applied to both Stage 1 and Stage 2 subjects.

- 1. **ENROLLED ANALYSIS SET (ENR)**

The enrolled analysis set will contain all subjects who sign the informed consent form (ICF) for this study.

- 1. **INTENT-TO-TREAT ANALYSIS SET (ITT)**

All subjects who are randomized will be included in the ITT. All analysis of ITT will be based on each subject’s randomized assigned treatment (not actual treatment received).

- 1. **SAFETY ANALYSIS SET (SAF)**

All subjects who are randomized and take at least 1 dose of study medication (meplazumab or placebo) will be included in the safety set.

If there is any doubt whether a subject started the treatment period, he/she will be assumed treated for the purposes of analysis.

For analyses and displays based on SAF analysis set, subjects will be classified according to the actual treatment received, i.e., if subjects randomized to the control group receive the study drug with low dose, they will be classified under the low-dose group. If subjects randomized to the study drug with low dose do not receive the study drug they will be classified under the control group.

- 1. **PHARMACOKINETIC ANALYSIS SET (PKS)**

Subjects who receive at least 1 dose of meplazumab and have at least 1 quantifiable meplazumab concentration post- dose without protocol deviations or events affecting the PK results will be part of this population.

For analyses and displays based on the PKS analysis set, subjects will be classified according to the actual treatment received.

Document: \\ieedc-vnasc01\BIOSdata\Jiangsu_PMBP\Meplazumab\SZA62090\Biostatistics\Documentation\SAP\

| Author: Yueh Wang, Yang Teng | Version Number: Version Date: | 2.0  13-Dec-2021 |
| --- | --- | --- |
| Template No.: CS_TP_BS016 Revision 6 Effective Date: 02Dec2019 | Reference: | CS_WI_BS005 |

Copyright © 2009, 2010, 2012, 2016, 2018, 2019 IQVIA. All rights reserved. The contents of this document are confidential and proprietary to IQVIA Holdings Inc. and its subsidiaries. Unauthorized use, disclosure or reproduction is strictly prohibited.


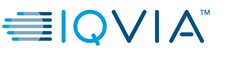
Jiangsu Pacific Meinuoke Biopharmaceutical Co., Ltd. (PMBP)

Protocol MPZ-II-02

Statistical Analysis Plan Page 21 of 82

- 1. **PHARMACOKINETIC PARAMETER ANALYSIS SET (PKPS)**

All subjects who receive meplazumab and have at least 1 PK parameter result without deviation or events affecting the results will be included in the PKPS.

Subjects who receive at least 1 dose of meplazumab will be evaluated on a case-by-case basis if sufficient meplazumab concentration-time data are available to allow calculation of at least 1 PK parameter.

For analyses and displays based on the PKPS analysis set, subjects will be classified according to the actual treatment received.

- 1. **PHARMACODYNAMIC ANALYSIS SET (PDS)**

The PDS will contain all subjects who receive study medication (drug or control) and have evaluable results for at least 1 PD endpoint post dose without deviation or events affecting the results.

For analyses and displays based on the PDS, subjects will be classified according to the actual treatment received.

- 1. **PROCESS FOR ANALYSIS SET ASSIGNMENT**

The analysis sets that will be used to summarize, analyze, and list the data collected during the course of this study are all defined in Section 5 and have agreement with PMBP before DBL. The ITT analysis set, and the SAF analysis set do not contain subjective criteria (e.g., protocol deviations). Hence, the authorization of this SAP will also stand as the agreement and authorization of the inclusion/exclusion of each subject in each of these analysis sets.

For the PKS, PKPS, and the PDS, the identification and agreement of protocol deviations or events which affect PK and/or PD results will be performed between the PK analyst, biostatistician, and the sponsor, prior to DBL, with sponsor authorization of any excluded subjects or their data.

For the exploratory PK results (and PD results, as appropriate) a 2-stage process will be followed:

- First stage (before unblinding): no concentration data will be included in the review. Data will be evaluated on a case-by-case basis to determine whether the subject should be excluded from the PK (and/or PD) analysis sets based on deviations/events that could affect PK (and/or PD) concentrations and/or PK parameters before the concentration results are disclosed.

Document: \\ieedc-vnasc01\BIOSdata\Jiangsu_PMBP\Meplazumab\SZA62090\Biostatistics\Documentation\SAP\

| Author: Yueh Wang, Yang Teng | Version Number: Version Date: | 2.0  13-Dec-2021 |
| --- | --- | --- |
| Template No.: CS_TP_BS016 Revision 6 Effective Date: 02Dec2019 | Reference: | CS_WI_BS005 |

Copyright © 2009, 2010, 2012, 2016, 2018, 2019 IQVIA. All rights reserved. The contents of this document are confidential and proprietary to IQVIA Holdings Inc. and its subsidiaries. Unauthorized use, disclosure or reproduction is strictly prohibited.


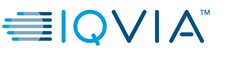
Jiangsu Pacific Meinuoke Biopharmaceutical Co., Ltd. (PMBP)

Protocol MPZ-II-02

Statistical Analysis Plan Page 22 of 82

- Second stage (after unblinding): data with concentration results will be reviewed. The PK (and PD) analysis sets defined prior to concentration results disclosure will be confirmed or updated.

The decisions made during the 2-stage process will be documented. Changes to the procedures (eg, deviations) or events which may impact the quality of the PK (and/or PD) data are outlined in the Section 18. Collection of PK and PD samples is optional in the study protocol. While missed sample collections are not considered a protocol deviation, the absence of available concentration-time data may lead to subject exclusion from the PK and/or PD analysis sets.

1. **GENERAL CONSIDERATIONS**
   1. **REFERENCE START DATE AND STUDY DAY**

Meplazumab is given in addition to SoC, and for SoC there will not necessarily be an obvious start date. It is expected that the study treatment will begin at the same day of randomization and therefore for this study, for efficacy and safety analyses, unless otherwise stated, the reference start date will be the date of randomization, i.e. Day 1.

Study Day will be calculated from the reference start date and will be used to show start/stop day of assessments and events.

Study Day will be computed as follows:

- Study Day = (Date of event –Date of randomization) + 1 if the date of the event is on or after the date of randomization;
- Study Day = (Date of event – Date of randomization) if the date of the event is prior to the date of randomization.

In the situation where the event date is partial or missing, Study Day and any corresponding durations will appear missing in the listings, and dates will be presented as partial or missing.

- 1. **BASELINE**

Unless otherwise specified, Baseline is defined as the last non-missing measurement taken prior to the reference start date (including unscheduled assessments). For patients who are dosed after the day of randomization which Document: \\ieedc-vnasc01\BIOSdata\Jiangsu_PMBP\Meplazumab\SZA62090\Biostatistics\Documentation\SAP\

| Author: Yueh Wang, Yang Teng | Version Number: Version Date: | 2.0  13-Dec-2021 |
| --- | --- | --- |
| Template No.: CS_TP_BS016 Revision 6 Effective Date: 02Dec2019 | Reference: | CS_WI_BS005 |

Copyright © 2009, 2010, 2012, 2016, 2018, 2019 IQVIA. All rights reserved. The contents of this document are confidential and proprietary to IQVIA Holdings Inc. and its subsidiaries. Unauthorized use, disclosure or reproduction is strictly prohibited.


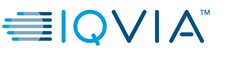
Jiangsu Pacific Meinuoke Biopharmaceutical Co., Ltd. (PMBP)

Protocol MPZ-II-02

Statistical Analysis Plan Page 23 of 82

would cause patients to have a missing baseline, the day of first dose will be used for baseline. In the case where the last non-missing measurement and the reference start date coincide and time of measurement is not available, that measurement will be considered pre-Baseline unless otherwise specified. For AEs commencing on the reference start date, the event will be defined as pre-Baseline or post-Baseline by the clinical team and collected on eCRF. Medications commencing on the date of randomization will be considered post-Baseline.

- 1. **UNSCHEDULED VISITS, RETESTS, AND EARLY TERMINATION DATA**

In the case of a retest, the measurement results will be recorded on an unscheduled visit form.

For by-visit summaries, data recorded at the nominal visit, per the schedule of activities, will be presented. That is, unscheduled data will not be included in by-visit summaries but might contribute to the Baseline timepoint and/or worst-case value, where required (e.g. shift tables or summaries involving the worst-case values for post-Baseline assessments).

Early termination data will be mapped to the next available visit number for by-visit summaries.

Listings will include scheduled, unscheduled and early discontinuation data. By-subject meplazumab concentration- time profiles will include all reported concentration results (scheduled and unscheduled) associated with the profile, if appropriate.

- 1. **WINDOWING CONVENTIONS**

Visit windowing will be performed for this study only for the below situation:

- The early termination data described in Section 6.3.
- The response rate at Day 29 for discharged subjects only. A 3 day visit window is allowed per protocol schedule of activities for subjects who are discharged before Day 29, and the 6-point ordinal score collected within the 3 days can be used for analysis, i.e. for discharged subjects, data collected between days 26 and 32 will be used for the day 29 analysis. In case there are multiple results within the 3 day window, the result with the Study Day closest to 29 will be used; if still multiple results collected at different Study Days with the same distance to Day 29, the worst score will be taken for analysis. The visit windowing rule will be applied for both Stage 1 and Stage 2 response rate analysis.

Document: \\ieedc-vnasc01\BIOSdata\Jiangsu_PMBP\Meplazumab\SZA62090\Biostatistics\Documentation\SAP\

| Author: Yueh Wang, Yang Teng | Version Number: Version Date: | 2.0  13-Dec-2021 |
| --- | --- | --- |
| Template No.: CS_TP_BS016 Revision 6 Effective Date: 02Dec2019 | Reference: | CS_WI_BS005 |

Copyright © 2009, 2010, 2012, 2016, 2018, 2019 IQVIA. All rights reserved. The contents of this document are confidential and proprietary to IQVIA Holdings Inc. and its subsidiaries. Unauthorized use, disclosure or reproduction is strictly prohibited.


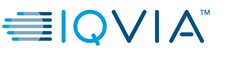
Jiangsu Pacific Meinuoke Biopharmaceutical Co., Ltd. (PMBP)

Protocol MPZ-II-02

Statistical Analysis Plan Page 24 of 82

- 1. **COMMON CALCULATIONS**

Change from baseline will be calculated as:

- Change from baseline = Test value at post-Baseline visit – Baseline value Percent change from baseline will be calculated as:
- Percent change from Baseline (%) = (Change from Baseline at post-Baseline visit / Baseline value) * 100%

1. **STATISTICAL CONSIDERATIONS**

For continuous non PK/PD data, descriptive statistics (i.e., n [number of subjects with available data], mean, standard deviation [SD], median, minimum, and maximum values) will be presented by treatment arms and visit, when applicable.

For categorical data, the number and percentages of subjects in each category will be presented by treatment arms and visit, when applicable.

The statistical parameters and presentations for PK and PD endpoints are described in Section 18.

- 1. **SAMPLE SIZE CALCULATION**

**Stage 1:**

Approximately 168 subjects will be randomized and allocated 1:1:1:1 (42:42:42:42) to receive meplazumab low dose, meplazumab medium dose, meplazumab high dose or control. Following a 29 days treatment period after completion of randomization, an interim analysis will be conducted to select the optimal meplazumab dose based on the response rates of clinical improvement at Day 29 between 3 dose levels, and safety data (see Section 16.4).

Using a step-down procedure for the comparisons between the dose groups and the control at a 2-sided alpha level of 0.05 each, fifty-four subjects per arm will power the study at 81% to distinguish the response rate at Day 29 of the dose groups from the control, assuming 80% response rate for the dose groups and 50% for the control (Bian et al. 2021 figure 4a).

The sample size is calculated based on the hypothesis:

*H01*: pdose = pControl

Document: \\ieedc-vnasc01\BIOSdata\Jiangsu_PMBP\Meplazumab\SZA62090\Biostatistics\Documentation\SAP\

| Author: Yueh Wang, Yang Teng | Version Number: Version Date: | 2.0  13-Dec-2021 |
| --- | --- | --- |
| Template No.: CS_TP_BS016 Revision 6 Effective Date: 02Dec2019 | Reference: | CS_WI_BS005 |

Copyright © 2009, 2010, 2012, 2016, 2018, 2019 IQVIA. All rights reserved. The contents of this document are confidential and proprietary to IQVIA Holdings Inc. and its subsidiaries. Unauthorized use, disclosure or reproduction is strictly prohibited.


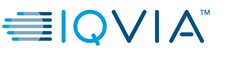
Jiangsu Pacific Meinuoke Biopharmaceutical Co., Ltd. (PMBP)

Protocol MPZ-II-02

Statistical Analysis Plan Page 25 of 82

*H11*: pdose ≠ pControl

where:

- Pdose is the proportion of subjects with response on Day 29 for dose group;
- PControl is the proportion of subjects with response on Day 29 for control group.

The sample size can be re-calculated by below formula:

𝑁1 = 𝐾 ∗ 𝑁2

Where K in the Stage 1 is 1, and N1 and N2 represent 2 different treatment arms

𝑁2 =

(𝑍�⁄2+𝑍�)2

𝑃 ����

2

[

(1−𝑃� ���)

+ 𝑃������𝑙(1 − 𝑃������𝑙)]

(𝑃����−𝑃������𝑙) 𝐾

Where α=0.05, β=0.1, which means this has at least 81% power to detect an actual difference on the response rate of

Day 29 between the dose group and control group at 2-sided alpha level 0.05.

**Stage 2:**

After the interim analysis for Stage 1, the primary endpoint and sample size calculation for Stage 2 will be re- evaluated based on the observed outcomes at Stage 1 and will be capped at 300 subjects in total. However, for Stage 2 it is initially planned that 240 more subjects will be randomized and allocated 2:1 (160:80) to receive the optimal meplazumab dose determined after Stage 1 or control. A total sample size of 240 on optimal meplazumab dose and control, has at least 95% power to detect an actual 30% difference on the response rate of clinical improvement (50% vs. 80%) between meplazumab and control at 2-sided alpha level 0.05.

If the selected primary endpoint after stage 1 is not response rate but belongs to the proportion type of endpoint, the sample size will be re-calculated based on the hypothesis and formula described in stage 1. If the selected primary endpoint belongs to time to event type of endpoint, the sample size will be re-calculated based on the hypothesis:

*H01*: λdose = λControl

*H11*: λdose ≠ λControl

where:

o λdose is the hazard rate of dose group;

Document: \\ieedc-vnasc01\BIOSdata\Jiangsu_PMBP\Meplazumab\SZA62090\Biostatistics\Documentation\SAP\

| Author: Yueh Wang, Yang Teng | Version Number: Version Date: | 2.0  13-Dec-2021 |
| --- | --- | --- |
| Template No.: CS_TP_BS016 Revision 6 Effective Date: 02Dec2019 | Reference: | CS_WI_BS005 |

Copyright © 2009, 2010, 2012, 2016, 2018, 2019 IQVIA. All rights reserved. The contents of this document are confidential and proprietary to IQVIA Holdings Inc. and its subsidiaries. Unauthorized use, disclosure or reproduction is strictly prohibited.


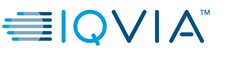
Jiangsu Pacific Meinuoke Biopharmaceutical Co., Ltd. (PMBP)

Protocol MPZ-II-02

Statistical Analysis Plan Page 26 of 82

o λControl is the hazard rate of control group. The sample size can be re-calculated by below formula:

𝑁1 = 𝐾 ∗ 𝑁2

Where K in the stage 1 is 1, and N1 and N2 represent 2 different treatment arms.

𝑁2 =

(𝑍�⁄2+𝑍�)2

𝜎2(𝜆�𝑜𝑠�)

2

[

+ 𝜎2(𝜆������𝑙) ]

(𝜆����−𝜆������𝑙) �

Where α=0.05, β=0.1, which means this has at least 90% power to detect an actual difference on the hazard rate between the dose group and control group at 2-sided alpha level 0.05.

All the above sample size will be calculated by statistical software where applicable (nQuery version 8.6, SAS version 9.4 or above), based on 2-sided Type I error rate=0.05, and 90% power.

Data collected at Stage 1 and Stage 2 will be analyzed separately.

- 1. **MISSING DATA**

All subjects recruited into the study will be accounted for, including those who did not complete the study. Subjects who withdraw from the study will have the reasons of withdrawal collected in the CRF.

Missing efficacy data will be handled as described in Sections 16.1.2 and 16.2.2.

Missing safety data will not be imputed, except for missing AE severity and relationship data (refer to Section 17.1.1). Partial or completely missing AE and medication dates will be judged by the rules as described in APPENDIX 1.

Missing PK concentrations will be handled as described in Section **Error! Reference source not found.**.

# STATISTICAL TESTS

All statistical tests will be conducted at the two-sided 5% significant level for the efficacy endpoints, unless otherwise specified in the description of the analyses. Confidence Intervals (CIs) will be two-sided at the 95% level. No statistical testing will be performed for the safety endpoints. Exploratory analyses for PK and/or PD endpoints

Document: \\ieedc-vnasc01\BIOSdata\Jiangsu_PMBP\Meplazumab\SZA62090\Biostatistics\Documentation\SAP\

| Author: Yueh Wang, Yang Teng | Version Number: Version Date: | 2.0  13-Dec-2021 |
| --- | --- | --- |
| Template No.: CS_TP_BS016 Revision 6 Effective Date: 02Dec2019 | Reference: | CS_WI_BS005 |

Copyright © 2009, 2010, 2012, 2016, 2018, 2019 IQVIA. All rights reserved. The contents of this document are confidential and proprietary to IQVIA Holdings Inc. and its subsidiaries. Unauthorized use, disclosure or reproduction is strictly prohibited.


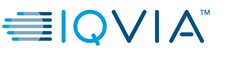
Jiangsu Pacific Meinuoke Biopharmaceutical Co., Ltd. (PMBP)

Protocol MPZ-II-02

Statistical Analysis Plan Page 27 of 82

are discussed in Section 18.

- 1. **MULTIPLE COMPARISONS/ MULTIPLICITY**

A step-down testing procedure using a fixed-sequence method will be used at Stage 1 interim for dose selection to adjust for multiple comparisons between the three dose groups and the control. The details can be found in Section

16.1.3. No further multiplicity control will be applied for the study.

- 1. **MULTICENTER STUDIES**

This study will be conducted by multiple (15 to 20) investigators at multiple (12 to 20) centers internationally, but may be changed based on actual recruitment. For the primary efficacy endpoint, centers or pooled centers will be included as a factor in the supplementary analyses.

If all study centers have >14 subjects for this study, study center will not be pooled. If any study center has <15 subjects for this study, center will be pooled preserving category (Teaching hospital vs. district general hospital). If the total number of subjects in a pooled category is <15, the smallest pooled category will be pooled with the next smallest center. The step will be repeated until all the centers or pooled centers with ≥15 subjects. The pooled rule will be discussed and approved by sponsor before final DBL.

- 1. **ADJUSTMENTS FOR COVARIATES AND FACTORS TO BE**

**INCLUDED IN ANALYSES**

The analyses for efficacy will be adjusted for the following covariates and factors. For details of their inclusion in the models, refer the Sections 16.1.3, and 16.2.3.

- Age (years) group at informed consent (2 levels: < 65 or ≥ 65);
- Baseline severity grade (2 levels: Grade 3 or Grade 4)
- Additional stratification factors if any as determined after evaluation of Stage 1 data
- Study center or pooled study center

If data are too sparse for one level of the above factors (i.e., less than 5% of ITT subjects with age more or equal to

Document: \\ieedc-vnasc01\BIOSdata\Jiangsu_PMBP\Meplazumab\SZA62090\Biostatistics\Documentation\SAP\

| Author: Yueh Wang, Yang Teng | Version Number: Version Date: | 2.0  13-Dec-2021 |
| --- | --- | --- |
| Template No.: CS_TP_BS016 Revision 6 Effective Date: 02Dec2019 | Reference: | CS_WI_BS005 |

Copyright © 2009, 2010, 2012, 2016, 2018, 2019 IQVIA. All rights reserved. The contents of this document are confidential and proprietary to IQVIA Holdings Inc. and its subsidiaries. Unauthorized use, disclosure or reproduction is strictly prohibited.


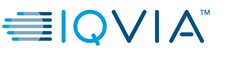
Jiangsu Pacific Meinuoke Biopharmaceutical Co., Ltd. (PMBP)

Protocol MPZ-II-02

Statistical Analysis Plan Page 28 of 82

65 years old), the factor will not be included in the statistical model/test to avoid convergence issue. Determination of the factor(s) to be included into the statistical models/tests will be finalized before the study DBL in a blinded fashion.

- 1. **EXAMINATION OF SUBGROUPS**

Subgroup analyses will be conducted as stated in Sections 16.1.5. It should be noted that the study was not designed to detect treatment differences with high statistical power within subgroups.

The subgroups are:

- Age (years) group at informed consent (2 levels: <65 and ≥65);
- Gender (2 levels: Female and Male);
- Race (5 levels: American Indian or Alaska Native, Asian, Black or African American, Native Hawaiian or Other Pacific Islanders, and White)
- Concomitant antiviral agents used (3 levels: none, single antiviral, or multiple antivirals)
- Prior Remdesivir use (2 levels: Yes and No)

If data are too sparse for one of the other subgroups, neither descriptive statistics nor statistical inferences will be provided for that subgroup, as well as for its complementary subgroup. For example, if only 3% of ITT subjects are more than or equal to 65 years old, no subgroup analysis (statistical inferences) will be provided for the subjects based on age category.

Determination of the subgroups to be summarized/analyzed will be finalized before the study DBL in a blinded fashion.

Exploratory subgroup analyses for PK endpoints are discussed in Section 18.

- 1. **UNBLINDING HANDLING**

Emergency unblinding for safety purposes is expected though, and those subjects will be kept in both the ITT analysis set and SAF analysis set.

For subjects that are unexpected (accidentally) unblinding, the subjects will remain in the SAF analysis set for safety

Document: \\ieedc-vnasc01\BIOSdata\Jiangsu_PMBP\Meplazumab\SZA62090\Biostatistics\Documentation\SAP\

| Author: Yueh Wang, Yang Teng | Version Number: Version Date: | 2.0  13-Dec-2021 |
| --- | --- | --- |
| Template No.: CS_TP_BS016 Revision 6 Effective Date: 02Dec2019 | Reference: | CS_WI_BS005 |

Copyright © 2009, 2010, 2012, 2016, 2018, 2019 IQVIA. All rights reserved. The contents of this document are confidential and proprietary to IQVIA Holdings Inc. and its subsidiaries. Unauthorized use, disclosure or reproduction is strictly prohibited.


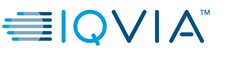
Jiangsu Pacific Meinuoke Biopharmaceutical Co., Ltd. (PMBP)

Protocol MPZ-II-02

Statistical Analysis Plan Page 29 of 82

data analysis and in the ITT analysis set, and all information, including efficacy information obtained after unblinding, will be used in the analyses. A footnote for related outputs will be included to explain why few subjects are excluded. No sensitivity analyses on efficacy endpoints that include the accidentally unblinded subjects will be performed, as such efficacy data will not be used inform any decision making.

- 1. **SOFTWARE VERSION**

All statistical analyses and production of listings, tables, and figures will be conducted using SAS Version 9.4 or higher. Flagging of PK/PD data and derivation of PK parameters will be performed using Phoenix® WinNonlin® V8.3 or higher (Certara, Princeton, New Jersey, USA).

1. **OUTPUT PRESENTATIONS**

APPENDIX 2 shows conventions for presentation of data in outputs.

The templates provided with this SAP describe the presentations for this study and therefore, the format and content of the summary tables, figures, and listings to be provided by IQVIA Biostatistics. Some minor modifications of the outputs may be necessary to accommodate actual data collected during the study.

1. **DISPOSITION AND WITHDRAWALS**

All subjects who provide informed consent and are screened will be accounted for in this study.

- 1. **DISPOSITION**

Number of subjects screened, subjects with screen failure and reason for screen failure will also be presented overall based on the ENR analysis set. Number of subjects randomized will be presented overall and by treatment arms for the ENR analysis set.

Number and percentages of subjects treated, ongoing on treatment (for IAs only), who completed/discontinued early from treatment (including reason for withdrawal), ongoing in study (for IAs only), and who completed/discontinued early from the study (including reason for withdrawal) will be provided overall and by treatment arm based on the ITT analysis set.

Document: \\ieedc-vnasc01\BIOSdata\Jiangsu_PMBP\Meplazumab\SZA62090\Biostatistics\Documentation\SAP\

| Author: Yueh Wang, Yang Teng | Version Number: Version Date: | 2.0  13-Dec-2021 |
| --- | --- | --- |
| Template No.: CS_TP_BS016 Revision 6 Effective Date: 02Dec2019 | Reference: | CS_WI_BS005 |

Copyright © 2009, 2010, 2012, 2016, 2018, 2019 IQVIA. All rights reserved. The contents of this document are confidential and proprietary to IQVIA Holdings Inc. and its subsidiaries. Unauthorized use, disclosure or reproduction is strictly prohibited.


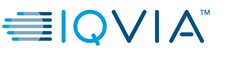
Jiangsu Pacific Meinuoke Biopharmaceutical Co., Ltd. (PMBP)

Protocol MPZ-II-02

Statistical Analysis Plan Page 30 of 82

Similarly, number of subjects included and excluded from each analysis set (including reason for exclusion) will be summarized overall and by treatment arms based on the ITT analysis set. A listing showing inclusion and exclusion of each subject from each analysis set, including reason for exclusion, will be provided.

- 1. **PROTOCOL DEVIATIONS**

Number and percentage of subject with important protocol deviations, as identified by the study team as being major or critical in a blinded fashion before the DBL, will be provided overall and by treatment arm based on the ITT analysis set for each category of protocol deviations specified in the Protocol Deviations Management Plan.

A listing of protocol deviations identified by the study team (important or not) will be provided.

Protocol deviations or events that have the potential to affect the reliability of PK and/or PD endpoints are discussed in Section 18.

1. **DEMOGRAPHIC AND OTHER BASELINE CHARACTERISTICS**

The following demographic and other baseline characteristics will be reported for this study:

- Age (years) – calculated relative to date of consent
- Age groups: <65 and ≥65
- Sex (Male, Female)
- Childbearing potential for female subjects only (Yes, No)
- Reason for not being of childbearing potential (Post-Menopausal, Premenarchal, Surgically Sterile, Other)
- Ethnicity (Hispanic or Latino, Not Hispanic or Latino, Not Reported, Unknown)
- Race (Multiple, American Indian or Alaska Native, Asian, Black or African American, Native Hawaiian or Other Pacific Islanders, White)
- Baseline weight (kg)
- Concomitant antiviral agents used (none, single antiviral, multiple antivirals)
- Prior Remdesivir use (Yes, No)

Document: \\ieedc-vnasc01\BIOSdata\Jiangsu_PMBP\Meplazumab\SZA62090\Biostatistics\Documentation\SAP\

| Author: Yueh Wang, Yang Teng | Version Number: Version Date: | 2.0  13-Dec-2021 |
| --- | --- | --- |
| Template No.: CS_TP_BS016 Revision 6 Effective Date: 02Dec2019 | Reference: | CS_WI_BS005 |

Copyright © 2009, 2010, 2012, 2016, 2018, 2019 IQVIA. All rights reserved. The contents of this document are confidential and proprietary to IQVIA Holdings Inc. and its subsidiaries. Unauthorized use, disclosure or reproduction is strictly prohibited.


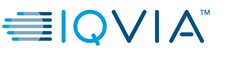
Jiangsu Pacific Meinuoke Biopharmaceutical Co., Ltd. (PMBP)

Protocol MPZ-II-02

Statistical Analysis Plan Page 31 of 82

- Concomitant Remdesivir use (Yes, No)
- Number of subjects by study center and pooled center
- Baseline ordinal scale for clinical severity (Grade 3, Grade 4)
- Any conditions that meet the risk factors of COVID-19 (Yes, No)

The risk factors included: chronic lung disease, including asthma, chronic renal disease, diabetes, heart disease, hypertension, autoimmune disease, women within 2 weeks postpartum and not breastfeeding, residents of long- term care facility, cancer, organ transplant

- Chest Imaging performed (Yes, No) and results (Normal, Abnormal)

Continuous demographic and other baseline characteristics will be summarized using descriptive statistics overall and by treatment arm based on the ITT analysis set. For categorical demographic and other baseline characteristics, number and percentage of subjects in each category will be provided overall and by treatment arm based on the ITT analysis set. No statistical testing will be carried out for demographic or other baseline characteristics.

- 1. **DERIVATIONS**

BMI, in kg/m2, will be calculated as follows:

- BMI (kg/ m2) = weight (kg)/ [height (m)2]

1. **MEDICAL HISTORY**

Wherever possible, relevant medical history within the past 5 years will be recorded in the CRF for past and ongoing conditions, including any risk factors of COVID-19 as defined in protocol. Medical history is defined as any medical conditions/diseases that started and stopped before randomization.

Medical history will be coded using the Medical Dictionary for Regulatory Activities (MedDRA), Version 23.1 or higher, and will be summarized by System Organ Class (SOC) and Preferred Term (PT) overall and by treatment arm based on the SAF analysis set. A subject having more than one medical condition/disease within the same SOC/PT will be counted only once for that SOC or PT. Number of subjects and percentage to the analysis set will be presented in frequency tables, ordered by SOC and PT in descending order of the frequency by total subjects. For SOCs or PTs with the same frequency, categories will be sorted alphabetically.

Document: \\ieedc-vnasc01\BIOSdata\Jiangsu_PMBP\Meplazumab\SZA62090\Biostatistics\Documentation\SAP\

| Author: Yueh Wang, Yang Teng | Version Number: Version Date: | 2.0  13-Dec-2021 |
| --- | --- | --- |
| Template No.: CS_TP_BS016 Revision 6 Effective Date: 02Dec2019 | Reference: | CS_WI_BS005 |

Copyright © 2009, 2010, 2012, 2016, 2018, 2019 IQVIA. All rights reserved. The contents of this document are confidential and proprietary to IQVIA Holdings Inc. and its subsidiaries. Unauthorized use, disclosure or reproduction is strictly prohibited.


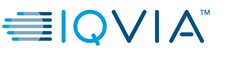
Jiangsu Pacific Meinuoke Biopharmaceutical Co., Ltd. (PMBP)

Protocol MPZ-II-02

Statistical Analysis Plan Page 32 of 82

All medical history will be listed.

1. **DISEASE HISTORY**

The following disease history characteristics will be summarized overall and by treatment arm based on the SAF analysis set and derived from the COVID Symptom Assessment eCRF page:

- Time since COVID-19 symptoms onset (days) – calculated relative to the date of randomization;
- Time since COVID-19 diagnosis (days) – calculated relative to date of randomization;
- Presence of each of the following COVID-19 symptoms at study entry:
  - Shortness of breath;
  - Cough;
  - Fever;
  - Fatigue/malaise;
  - Myalgia;
- Time since hospital admission (days)– calculated relative to date of randomization;
- In intensive care unit (ICU)/high dependency unit (HDU) at study entry (Yes, No);
- Time since ICU/HDU admission (days) for subjects in ICU/HDU at study entry – calculated relative to date of randomization;
- On supplemental oxygen at study entry (Yes, No);
- Type of supplemental oxygen for subjects on supplemental oxygen at study entry (Invasive Mechanical Ventilation, Non-Invasive Mechanical Ventilation, ECMO);
- Time since start of supplemental oxygen (days) for subjects on supplemental oxygen at study entry - calculated relative to date of randomization;
- On invasive mechanical ventilation at study entry;
- Type of invasive mechanical ventilation for subjects on invasive mechanical ventilation at study entry (Via

Document: \\ieedc-vnasc01\BIOSdata\Jiangsu_PMBP\Meplazumab\SZA62090\Biostatistics\Documentation\SAP\

| Author: Yueh Wang, Yang Teng | Version Number: Version Date: | 2.0  13-Dec-2021 |
| --- | --- | --- |
| Template No.: CS_TP_BS016 Revision 6 Effective Date: 02Dec2019 | Reference: | CS_WI_BS005 |

Copyright © 2009, 2010, 2012, 2016, 2018, 2019 IQVIA. All rights reserved. The contents of this document are confidential and proprietary to IQVIA Holdings Inc. and its subsidiaries. Unauthorized use, disclosure or reproduction is strictly prohibited.


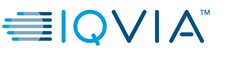
Jiangsu Pacific Meinuoke Biopharmaceutical Co., Ltd. (PMBP)

Protocol MPZ-II-02

Statistical Analysis Plan Page 33 of 82

Endotracheal Tube, Via Tracheostomy Tube, Via ECMO);

- Time since start of invasive mechanical ventilation (days) for subjects on invasive mechanical ventilation at study entry - calculated relative to date of randomization;

All disease history characteristics will be listed.

- 1. **DERIVATIONS**

‘Time since’ disease history characteristics, in days, will be calculated as follows:

- Time since COVID-19 symptoms onset (days) = (Date of randomization – Date of COVID-19 symptoms onset);
- Time since COVID-19 diagnosis (days) = (Date of randomization - Date of COVID-19 diagnosis);
- Time since hospital admission (days) = (Date of randomization – Date of hospitalization admission due to COVID-19)
- Time since ICU/HDU admission (days) = (Date of randomization – Date of ICU/HDU admission due to COVID-19) if subject is already in ICU/HDU at study entry;
- Time since start of supplemental oxygen (days) = (Date of randomization – Date of start of supplemental oxygen) if subject is already on supplemental oxygen at study entry;
- Time since start of invasive mechanical ventilation (days) = (Date of randomization – Date of start of invasive mechanical ventilation) if subject is already on invasive mechanical ventilation at study entry.

1. **MEDICATIONS AND PROCEDURES**

- Prior medications/procedures are defined as any medication/procedures that started and stopped prior to the date of randomization.
- Concomitant medications/procedures are defined as:
  - Any medication/procedures that started before randomization AND was ongoing at the date of randomization or ended on the date of randomization;

Document: \\ieedc-vnasc01\BIOSdata\Jiangsu_PMBP\Meplazumab\SZA62090\Biostatistics\Documentation\SAP\

| Author: Yueh Wang, Yang Teng | Version Number: Version Date: | 2.0  13-Dec-2021 |
| --- | --- | --- |
| Template No.: CS_TP_BS016 Revision 6 Effective Date: 02Dec2019 | Reference: | CS_WI_BS005 |

Copyright © 2009, 2010, 2012, 2016, 2018, 2019 IQVIA. All rights reserved. The contents of this document are confidential and proprietary to IQVIA Holdings Inc. and its subsidiaries. Unauthorized use, disclosure or reproduction is strictly prohibited.


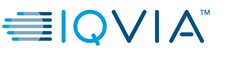
Jiangsu Pacific Meinuoke Biopharmaceutical Co., Ltd. (PMBP)

Protocol MPZ-II-02

Statistical Analysis Plan Page 34 of 82

o Any medication/procedures that started on or after the date of randomization.

Partially or completely missing medication start and stop dates will be handled as described in APPENDIX 1.

All medications will be coded using the World Health Organization (WHO) Drug Global dictionary, Version B3 September 2020 or higher. All procedures will be coded using the MedDRA dictionary, version 23.1 or higher.

Prior and concomitant medications will be summarized by Anatomical Therapeutic Class (ATC) level 2 and preferred drug name overall and by treatment arm based on the SAF analysis set. A subject having more than one medication within the same ATC Level 2 or preferred drug name will be counted only once for that ATC Level 2 or preferred drug name. Number of subjects and percentage to the analysis set will be presented in frequency tables, ordered by ATC and PT in descending order of the frequency by total subjects. For ATCs or PTs with the same frequency, categories will be sorted alphabetically.

All medications/procedures (prior, and concomitant) will be listed.

1. **EXPOSURE TO STUDY DRUG**

The study drug will be administered as 2 injections on Day 1 and 8. Exposure to study drug will be summarized by each treatment arm based on the SAF analysis set categorically for each dose as below:

- Exposure to first dose (Yes, No)
- Exposure to second dose (Yes, No)

The following additional information for exposure will also be summarized for each dose and overall, by treatment arm as below:

- Infusion period (minutes) (For each dose only)
- Dose adjustment (Dose not changed, Dose increased, Dose reduced, Drug interrupted, Drug withdrawal, Not applicable, Unknown)
- Dose interrupted
  - Interruption times
  - Duration (subject level) for those subjects with dose interruption(s) (Minutes)

Document: \\ieedc-vnasc01\BIOSdata\Jiangsu_PMBP\Meplazumab\SZA62090\Biostatistics\Documentation\SAP\

| Author: Yueh Wang, Yang Teng | Version Number: Version Date: | 2.0  13-Dec-2021 |
| --- | --- | --- |
| Template No.: CS_TP_BS016 Revision 6 Effective Date: 02Dec2019 | Reference: | CS_WI_BS005 |

Copyright © 2009, 2010, 2012, 2016, 2018, 2019 IQVIA. All rights reserved. The contents of this document are confidential and proprietary to IQVIA Holdings Inc. and its subsidiaries. Unauthorized use, disclosure or reproduction is strictly prohibited.


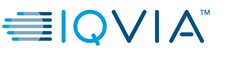
Jiangsu Pacific Meinuoke Biopharmaceutical Co., Ltd. (PMBP)

Protocol MPZ-II-02

Statistical Analysis Plan Page 35 of 82

- Main reason for dose adjustment (Adverse event, Other)
- Overdose (Yes, No)
- Quantity of overdose (mg) (For each dose only)
  1. **DERIVATIONS**
- Infusion period (minutes) = (End date and time of study drug infusion – Start date and time of study drug infusion) +1
- Dose interrupted duration (subject level) = Sum of all the interrupted duration occurred for one exposure

1. **COMPLIANCE WITH STUDY DRUG**

Not applicable. As treatments are administered only twice on Day 1 and Day 8, compliance is not calculated for the study.

1. **EFFICACY ENDPOINTS**

Unless otherwise indicated, all efficacy summaries and figures will be presented by treatment arm and visit, when appropriate, based on the ITT analysis set for both Stage 1 and Stage 2.

- 1. **PRIMARY EFFICACY**
     1. **PRIMARY EFFICACY ENDPOINT**

**Stage 1:**

There is no primary endpoint in Stage 1.

Dose selection endpoint: Determine an optimal dose based on response rate at Day 29 by sustained clinical improvement of 2 points (from randomization) on 6-point ordinal scale.

The 6-point ordinal scale, which is used to assess subject’s clinical status is as below:

Document: \\ieedc-vnasc01\BIOSdata\Jiangsu_PMBP\Meplazumab\SZA62090\Biostatistics\Documentation\SAP\

| Author: Yueh Wang, Yang Teng | Version Number: Version Date: | 2.0  13-Dec-2021 |
| --- | --- | --- |
| Template No.: CS_TP_BS016 Revision 6 Effective Date: 02Dec2019 | Reference: | CS_WI_BS005 |

Copyright © 2009, 2010, 2012, 2016, 2018, 2019 IQVIA. All rights reserved. The contents of this document are confidential and proprietary to IQVIA Holdings Inc. and its subsidiaries. Unauthorized use, disclosure or reproduction is strictly prohibited.


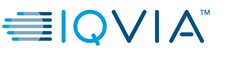
Jiangsu Pacific Meinuoke Biopharmaceutical Co., Ltd. (PMBP)

Protocol MPZ-II-02

Statistical Analysis Plan Page 36 of 82

**Stage 2:**

1. Not hospitalized;
2. Hospitalized, not requiring supplemental oxygen;
3. Hospitalized, requiring supplemental oxygen;
4. Hospitalized, on non-invasive ventilation or high flow oxygen devices;
5. Hospitalized, on invasive mechanical ventilation or ECMO;
6. Death.

Primary endpoints for Stage 2 will be determined based on evaluation of Stage 1 results at the interim analysis. Candidate primary efficacy endpoints are listed as follows:

- - Time to sustained clinical improvement (days; Time frame: Day 1 through Day 29) of at least 2 points (from randomization) on a 6-point ordinal scale, where sustained improvement is improvement without subsequent worsening, or live discharge from the hospital, whichever comes first
  - Response rate, as defined by a sustained improvement of 2 points on a 6-point ordinal scale, at Day 29
  - Mortality at Day 29
  - Proportion of subjects alive and discharged without supplemental oxygen at Day 29

For both stage 1 and stage 2, the sustained clinical improvement is defined as improvement without subsequent worsening, e.g. if a patient improves from an ordinal scale grade of 5 at Baseline to a grade of 3, but subsequently has a grade greater than 3 this would be considered as subsequent worsening and the patient would be a non- responder at that timepoint.

- - 1. **MISSING DATA IMPUTATION METHOD FOR PRIMARY EFFICACY ENDPOINT**

The dose selection endpoint at Stage 1 and candidate primary endpoint at Stage 2 includes binary endpoints and time to event endpoints.

The handling rules for binary endpoints if they are missing at Day 29 are as below:

- - Response rate at Day 29:

Subjects with no data at Day 29 will be counted as a non-responder.

Document: \\ieedc-vnasc01\BIOSdata\Jiangsu_PMBP\Meplazumab\SZA62090\Biostatistics\Documentation\SAP\

| Author: Yueh Wang, Yang Teng | Version Number: Version Date: | 2.0  13-Dec-2021 |
| --- | --- | --- |
| Template No.: CS_TP_BS016 Revision 6 Effective Date: 02Dec2019 | Reference: | CS_WI_BS005 |

Copyright © 2009, 2010, 2012, 2016, 2018, 2019 IQVIA. All rights reserved. The contents of this document are confidential and proprietary to IQVIA Holdings Inc. and its subsidiaries. Unauthorized use, disclosure or reproduction is strictly prohibited.


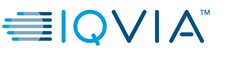
Jiangsu Pacific Meinuoke Biopharmaceutical Co., Ltd. (PMBP)

Protocol MPZ-II-02

Statistical Analysis Plan Page 37 of 82

- - Mortality at Day 29:

We do not expect missing data for mortality rate as death must be collected and reported after study withdrawal, except for those who lost to follow-up. Subjects who continue to be unreachable after at least 3 attempts and there’s no information available to say they are alive will be viewed as death.

- - Proportion of subjects alive and discharged without supplemental oxygen at Day 29: Subjects with no data by Day 29 will be treated as a non-responder.
    1. **PRIMARY ANALYSIS OF PRIMARY EFFICACY ENDPOINT**

**Stage 1:** There is no primary endpoint in Stage 1.

For dose selection purpose: The difference of response rates between each dose group and control group will be calculated and displayed. This will be calculated as follows:

Difference in proportions = the proportion of dose group - the proportion of control group.

In addition, response rate at Day 29 of the low, medium, and high dose groups will be compared against that of the control group using a Chi-Square test. The comparison of the response rate between the dose groups and the control will be tested with a step-down procedure, using a fixed-sequence method, at the 2-sided alpha level of 0.05 each, and will proceed in the following order, high versus control, medium versus control, and low versus control. If one preceding testing fails to be rejected, all the following will not be tested. The hypotheses and testing order are as follows:

First step is to test 𝐻01: 𝑃𝐻𝑖𝑔ℎ = 𝑃𝐶���𝑟�� 𝑣� 𝐻𝐴1: 𝑃𝐻𝑖𝑔ℎ ≠ 𝑃𝐶���𝑟�� ,

If the null hypothesis in the last step is rejected, test 𝐻02: 𝑃���𝑖�� = 𝑃𝐶���𝑟�� 𝑣� 𝐻𝐴2 : 𝑃���𝑖�� ≠ 𝑃𝐶���𝑟�� , Similarly, if the null hypothesis in the last step is rejected, test 𝐻03: 𝑃��𝑤 = 𝑃𝐶���𝑟�� 𝑣� 𝐻𝐴3: 𝑃��𝑤 ≠ 𝑃𝐶���𝑟�� *.*

where:

- PDose is the proportion of subjects with response on Day 29 for MPZ-II-02 high dose, medium dose or low dose;
- pcontrol is the proportion of subjects with response on Day 29 for control group.

**Stage 2:** The primary endpoint will be determined during the interim analysis of Stage 1 data*.*

Document: \\ieedc-vnasc01\BIOSdata\Jiangsu_PMBP\Meplazumab\SZA62090\Biostatistics\Documentation\SAP\

| Author: Yueh Wang, Yang Teng | Version Number: Version Date: | 2.0  13-Dec-2021 |
| --- | --- | --- |
| Template No.: CS_TP_BS016 Revision 6 Effective Date: 02Dec2019 | Reference: | CS_WI_BS005 |

Copyright © 2009, 2010, 2012, 2016, 2018, 2019 IQVIA. All rights reserved. The contents of this document are confidential and proprietary to IQVIA Holdings Inc. and its subsidiaries. Unauthorized use, disclosure or reproduction is strictly prohibited.


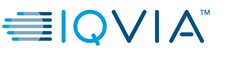
Jiangsu Pacific Meinuoke Biopharmaceutical Co., Ltd. (PMBP)

Protocol MPZ-II-02

Statistical Analysis Plan Page 38 of 82

- - Time to sustained clinical improvement or live discharge from hospital by Day 29

Number and percentage of subjects with and without sustained clinical improvement or live discharge from hospital by Day 29 from randomization will be presented by treatment arm based on the ITT analysis set. To be considered an event in the analysis, a subject needs to show a sustained improvement of at least 2 points (from randomization) on the 6-point ordinal scale without subsequent worsening, or live discharge from hospital. Subjects who require re- hospitalization, and discharge again before Day 29, will still be considered an event in the analysis, based on the second hospitalization to be evaluated in the same manner as those subjects in the first hospitalization.

For subjects without the event, the main reason for censoring will be summarized. The censoring convention for time to event endpoint by Day 29 is as below:

- - Subjects alive but did not have sustained 2-point improvement
  - Subjects who did not discharge from hospital
  - Subjects who are initially discharged but then subsequently transferred to hospital care of medical care facilities
  - Subjects who die
  - Subjects who live discharge but withdraws from the study before Day 29

The above censoring conditions will all have their time to sustained clinical improvement censored at Day 29. The time to sustained clinical improvement will be compared between the treatment arms. The Kaplan Meier estimator will be used, Kaplan Meier curves will be plotted for each treatment arm, and the log-rank test will be used for comparing the treatment arms at the 2-sided alpha level of 0.05. In addition, Cox regression model will be used by adjusting for age group (age <65years versus ≥65 years) and baseline severity grade; the HR and its 95% CI, and the p-value will be reported.

- - Response rate at Day 29

Difference in proportions will be presented and calculated as per Stage 1. The analysis of response rate at Day 29 will be conducted between the selected dose group and control using Cochran–Mantel–Haenszel (CMH) statistic, stratifying for age group (age <65 years versus ≥65 years), and baseline severity grade (grade 3 versus grade 4), and additional stratification factors if any as determined after evaluation of Stage 1 data. The p value associated with the CMH statistic will be compared at the 2-sided 0.05 alpha level. The hypothesis to be tested is:

*H0*: R = 1

Document: \\ieedc-vnasc01\BIOSdata\Jiangsu_PMBP\Meplazumab\SZA62090\Biostatistics\Documentation\SAP\

| Author: Yueh Wang, Yang Teng | Version Number: Version Date: | 2.0  13-Dec-2021 |
| --- | --- | --- |
| Template No.: CS_TP_BS016 Revision 6 Effective Date: 02Dec2019 | Reference: | CS_WI_BS005 |

Copyright © 2009, 2010, 2012, 2016, 2018, 2019 IQVIA. All rights reserved. The contents of this document are confidential and proprietary to IQVIA Holdings Inc. and its subsidiaries. Unauthorized use, disclosure or reproduction is strictly prohibited.


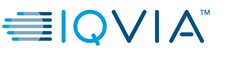
Jiangsu Pacific Meinuoke Biopharmaceutical Co., Ltd. (PMBP)

Protocol MPZ-II-02

Statistical Analysis Plan Page 39 of 82

*H1*: R ≠ 1

where:

o R is the odds ratio. The null hypothesis is that there’s no association between the treatment groups and response.

- - Mortality at Day 29

The analysis of mortality rate at Day 29 will be conducted between the selected dose group and control using the same methodology as described above of response rate. The hypothesis to be tested is:

*H0*: R = 1

*H1*: R ≠ 1

where:

o R is the odds ratio. The null hypothesis is that there’s no association between the treatment groups and death.

- - Proportion of subjects alive and discharged without supplemental oxygen at Day 29

Subjects alive and discharged without supplemental oxygen will be captured in the eCRF “Death Details” forms without death date, “Hospitalization” form with stop date before Day 29, and “Supplemental Oxygen and Mechanical Ventilation” form without any type of supplemental oxygen recorded after discharge. The analysis of proportion of subjects alive and discharged without supplemental oxygen at Day 29 will be conducted between the selected dose group and control using the same methodology as described above of response rate. The null hypothesis to be tested is there’s no association between the treatment groups for subjects alive and discharged without supplemental oxygen at Day 29.

- - 1. **SENSITIVITY ANALYSES FOR PRIMARY EFFICACY ENDPOINT**

Sensitivity analyses on the binary endpoints, including response rate at Day 29, mortality at Day 29, and proportion of subjects alive and discharged without supplemental oxygen at Day 29, will be provided by fitting the response variable using logistic regression, including treatment, baseline severity, baseline severity and treatment interaction, age group (age <65 years versus ≥65 years), and age group and treatment interaction as fixed effects for Stage 2.

Model-based point estimates for the treatment effects, 95% CIs, and p-values will be calculated.

Document: \\ieedc-vnasc01\BIOSdata\Jiangsu_PMBP\Meplazumab\SZA62090\Biostatistics\Documentation\SAP\

| Author: Yueh Wang, Yang Teng | Version Number: Version Date: | 2.0  13-Dec-2021 |
| --- | --- | --- |
| Template No.: CS_TP_BS016 Revision 6 Effective Date: 02Dec2019 | Reference: | CS_WI_BS005 |

Copyright © 2009, 2010, 2012, 2016, 2018, 2019 IQVIA. All rights reserved. The contents of this document are confidential and proprietary to IQVIA Holdings Inc. and its subsidiaries. Unauthorized use, disclosure or reproduction is strictly prohibited.


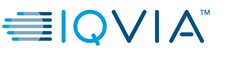
Jiangsu Pacific Meinuoke Biopharmaceutical Co., Ltd. (PMBP)

Protocol MPZ-II-02

Statistical Analysis Plan Page 40 of 82

Sensitivity analysis on time to sustained clinical improvement by Day 29 will be provided by viewing subjects who require re-hospitalization, and who were discharged again before Day 29 as not meeting the “sustained clinical improvement” event in the analysis. These subjects will be censored at Day 29.

- - 1. **SUPPLEMENTARY ANALYSES FOR PRIMARY EFFICACY ENDPOINT**

The following supplementary analyses will be performed for the primary efficacy endpoint of Stage 2:

- - For Time to sustained clinical improvement
    - The proportional hazard assumption will be examined as suggested by Therneau and Grambsch (2000) and Lawless (1982), as follows:

For each categorical factor (lets say X), a plot of log negative log survival distribution function of the lifetimes (i.e. log [-log S0 (t|xi)]) versus the log of the lifetime (i.e., log [t]) within each level of xi should produce roughly parallel lines if a Cox proportional hazard is appropriate.

This visual inspection is done for informative purposes only. Analyses of the primary efficacy endpoint will not be adjusted based on these plots.

- - - A forest plot will be produced for the hazard ratio and its 95% CI within each of the subgroups described in Section 7.7 and for the primary analysis.
    - The same analysis as Section 16.1.3 will be repeated but the model will include center/pooled center as a fixed effect.
  - For binary endpoints:
    - For each subgroup defined in Section 7.7, number and proportion of subjects with and without outcome will be provided by treatment group based on the ITT analysis set. The same analysis as for the primary endpoints (refer to section 16.1.3) will be repeated for each subgroup, with the following exception: for subgroups corresponding to one of the factor levels stratified by CMH statistic, the corresponding factor will be excluded from the CMH statistic. For example, the age group factor will not be stratified in the CMH statistic for the analysis of age<65 years subgroup and analysis of age ≥65 years subgroup.

Document: \\ieedc-vnasc01\BIOSdata\Jiangsu_PMBP\Meplazumab\SZA62090\Biostatistics\Documentation\SAP\

| Author: Yueh Wang, Yang Teng | Version Number: Version Date: | 2.0  13-Dec-2021 |
| --- | --- | --- |
| Template No.: CS_TP_BS016 Revision 6 Effective Date: 02Dec2019 | Reference: | CS_WI_BS005 |

Copyright © 2009, 2010, 2012, 2016, 2018, 2019 IQVIA. All rights reserved. The contents of this document are confidential and proprietary to IQVIA Holdings Inc. and its subsidiaries. Unauthorized use, disclosure or reproduction is strictly prohibited.


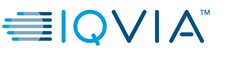
Jiangsu Pacific Meinuoke Biopharmaceutical Co., Ltd. (PMBP)

Protocol MPZ-II-02

Statistical Analysis Plan Page 41 of 82

**16.2. SECONDARY EFFICACY**

To assess the potential primary efficacy endpoints for Stage 2, the below endpoints will also be performed by using Stage 1 data:

- - Time to sustained clinical improvement or live discharge from hospital by Day 29
  - Mortality at Day 29
  - Proportion of subjects alive and discharged without supplemental oxygen at Day 29 The detail on the analysis methods are the same as described in Section 16.1.3.

For Stage 2, the secondary efficacy analyses will be performed on the ITT analysis set, and details can be found in the following sections.

- - 1. **SECONDARY EFFICACY ENDPOINTS & DERIVATIONS**
       1. **Response rate at Day 2, 8, 15**

The definition of responder is provided in Section 16.1.1. Subjects who have no ordinal scale assessment on a particular study day, (including subjects who have died prior to that study day) will be considered as non - responders.

- - - 1. **Proportion of subjects alive and discharge without supplemental oxygen at Day 15 and Day 57**

The definition of responder to live discharge is provided in Section 16.1.3. The proportion will be based on subjects alive and discharge without supplemental oxygen by each treatment arm at Day 15 and 57 separately.

- - - 1. **Mortality at Days 15 and 57**

For subjects who have died, date of death will be taken from the “Death Details” form of the eCRF. Day of death will be derived as (Date of death - date of randomization) +1.

Subjects will be counted as having died at each of Days 15, and 57 if they died on or prior to each of those respective Study Days. Subjects who died after each of Day 15, or Day 57 will be counted as alive at each of these

Document: \\ieedc-vnasc01\BIOSdata\Jiangsu_PMBP\Meplazumab\SZA62090\Biostatistics\Documentation\SAP\

| Author: Yueh Wang, Yang Teng | Version Number: Version Date: | 2.0  13-Dec-2021 |
| --- | --- | --- |
| Template No.: CS_TP_BS016 Revision 6 Effective Date: 02Dec2019 | Reference: | CS_WI_BS005 |

Copyright © 2009, 2010, 2012, 2016, 2018, 2019 IQVIA. All rights reserved. The contents of this document are confidential and proprietary to IQVIA Holdings Inc. and its subsidiaries. Unauthorized use, disclosure or reproduction is strictly prohibited.


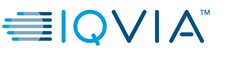
Jiangsu Pacific Meinuoke Biopharmaceutical Co., Ltd. (PMBP)

Protocol MPZ-II-02

Statistical Analysis Plan Page 42 of 82

respective Study Days.

For each subject who has not died at the time of analysis, the Study Day that they were last known date alive will be derived, using the rules for censored observations of time to death. If that Study Day is after each of Day 15, or Day 57 the subject will be counted as alive at each of these respective Study Days. If that Study Day is on or prior to Day 15, or Day 57 the subject will be classified as a censored for the respective Study Day.

- - - 1. **Time from treatment start date to death**

Date of death will be taken from the “Death Details” form of the eCRF. Time from treatments start date to death will be defined as: (date of death – date of treatment start date) +1.

Subjects who have not died or are permanently lost to follow-up at the time of analysis will have censored observations, and will all have their time to death censored at the last known date alive, which will be derived from “Date of completion/discontinuation” of “Disposition” form in the eCRF

- - - 1. **Time to sustained recovery (Days)**

Time to sustained recovery is defined from randomization to the first day on which one of the following 2 categories is achieved without subsequent worsening using the 6-point ordinal scale during the core study period (Day1 through Day 29):

1. Not hospitalized
2. Hospitalized, not requiring supplemental oxygen Calculated as: (the first date of recovery – date of randomization) +1

The definition of sustained recovery is recovery without subsequent worsening, e.g. if a patient achieves grade 2 from an ordinal scale grade, but subsequently has a grade greater than 2 during the study period, this would be considered as subsequent worsening and the patient would be a non-responder.

- - - 1. **Duration (days) of oxygen use and oxygen-free days**

The duration of each occurrence of oxygen use will be derived based on the start date and time, and end date and time of the use of any type of supplemental oxygen (Invasive Mechanical Ventilation, Non-Invasive Mechanical Ventilation, ECMO) as captured on the eCRF “Supplemental Oxygen and Mechanical Ventilation” form. For each

Document: \\ieedc-vnasc01\BIOSdata\Jiangsu_PMBP\Meplazumab\SZA62090\Biostatistics\Documentation\SAP\

| Author: Yueh Wang, Yang Teng | Version Number: Version Date: | 2.0  13-Dec-2021 |
| --- | --- | --- |
| Template No.: CS_TP_BS016 Revision 6 Effective Date: 02Dec2019 | Reference: | CS_WI_BS005 |

Copyright © 2009, 2010, 2012, 2016, 2018, 2019 IQVIA. All rights reserved. The contents of this document are confidential and proprietary to IQVIA Holdings Inc. and its subsidiaries. Unauthorized use, disclosure or reproduction is strictly prohibited.


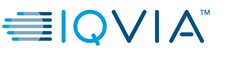
Jiangsu Pacific Meinuoke Biopharmaceutical Co., Ltd. (PMBP)

Protocol MPZ-II-02

Statistical Analysis Plan Page 43 of 82

subject, the duration in days of oxygen use will be derived as the sum of the duration (in minutes) of each occurrence of oxygen use, divided by 1440 (=24*60).

The number of oxygen-free days will be derived as the number of unique 24-hour days for which no oxygen was used.

To account for the different durations of the study for subjects, including those who die and may therefore have less oxygen use recorded than other subjects, the duration of oxygen use and the number of oxygen-free days will also be converted into percentages based on the total time in hospital for a subject, from randomization to the “Stop date” of admission as captured on the eCRF “Hospitalization” form. If a subject dies and the stop date of admission is missing, the date of death will be used instead.

- - - 1. **Duration (days) of mechanical ventilation and mechanical ventilation-free days**

The duration of ventilation will be derived based on the start date and time, and end date and time of either Invasive Mechanical Ventilation or Non-Invasive Mechanical Ventilation as the “Type of Supplemental Oxygen” captured on the eCRF “Supplemental Oxygen and Mechanical Ventilation”.

The number of ventilation-free days will be derived as the number of unique 24-hour days for which no ventilation was provided.

To account for the different durations of the study for subjects, including those who die and may therefore have less ventilation recorded than other subjects, the duration of ventilation and the number of ventilation-free days will also be converted into percentages based on the total time in hospital for a subject, from randomization to the Stop date of admission as captured on the eCRF “Hospitalization” form. If a subject dies and the stop date of admission is missing, the date of death will be used instead.

- - - 1. **Incidence of new mechanical ventilation use and duration (days) of new mechanical ventilation use**

New mechanical ventilation use is defined as mechanical ventilation other than that occurring at the time of randomization, i.e. for subjects who are on ventilation (either invasive or non-invasive) at the time of randomization, new ventilation use would be ventilation after the end of the ventilation at the time of randomization. For subjects who are not on ventilation at the time of randomization, any ventilation use after randomization would be considered as new ventilation use.

The duration in days, of new ventilation use would be derived in a similar manner as for duration of ventilation in

Document: \\ieedc-vnasc01\BIOSdata\Jiangsu_PMBP\Meplazumab\SZA62090\Biostatistics\Documentation\SAP\

| Author: Yueh Wang, Yang Teng | Version Number: Version Date: | 2.0  13-Dec-2021 |
| --- | --- | --- |
| Template No.: CS_TP_BS016 Revision 6 Effective Date: 02Dec2019 | Reference: | CS_WI_BS005 |

Copyright © 2009, 2010, 2012, 2016, 2018, 2019 IQVIA. All rights reserved. The contents of this document are confidential and proprietary to IQVIA Holdings Inc. and its subsidiaries. Unauthorized use, disclosure or reproduction is strictly prohibited.


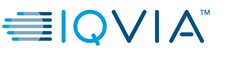
Jiangsu Pacific Meinuoke Biopharmaceutical Co., Ltd. (PMBP)

Protocol MPZ-II-02

Statistical Analysis Plan Page 44 of 82

Section 16.2.1.7 but excluding the initial period of ventilation for subjects who were being ventilated at the time of randomization.

- - - 1. **Incidence of new mechanical ventilation use after mechanical ventilation extubation period of 24 hours**

New mechanical ventilation use after mechanical ventilation extubation period of 24 hours is defined as mechanical ventilation use other than that had be extubated, and start date and time are 24 hours later than the end date and time of previous mechanical ventilation. The number of subjects meeting the definition will be displayed, and the percentage will be calculated based on the subjects with mechanical ventilation extubation by each treatment arm.

- - - 1. **Incidence of rehospitalization following hospital discharge**

The incidence will be calculated for the re-hospitalized subjects based on subjects with hospital discharge during the whole study period by each treatment arm. The hospitalization information can be captured from eCRF “Hospitalization” form.

- - - 1. **Duration (days) of ICU and hospitalization**

The duration of ICU and hospitalization will be derived based on the start date, and end date of “Hospitalization Status” selected as “ICU” and all hospitalization records captured on eCRF “Hospitalization” separately.

- - 1. **MISSING DATA IMPUTATION METHOD FOR SECONDARY EFFICACY ENDPOINTS**

Non-completer in binary endpoints (including response rate at Day 2, 8, and 15, proportion of subjects alive and discharge without supplemental oxygen at Day 15, and 57) will be treated as non-responder, same as the primary analysis mentioned in Section 16.1.2. There will no missing data imputation for secondary efficacy endpoints.

Document: \\ieedc-vnasc01\BIOSdata\Jiangsu_PMBP\Meplazumab\SZA62090\Biostatistics\Documentation\SAP\

| Author: Yueh Wang, Yang Teng | Version Number: Version Date: | 2.0  13-Dec-2021 |
| --- | --- | --- |
| Template No.: CS_TP_BS016 Revision 6 Effective Date: 02Dec2019 | Reference: | CS_WI_BS005 |

Copyright © 2009, 2010, 2012, 2016, 2018, 2019 IQVIA. All rights reserved. The contents of this document are confidential and proprietary to IQVIA Holdings Inc. and its subsidiaries. Unauthorized use, disclosure or reproduction is strictly prohibited.


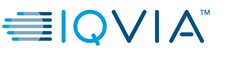
Jiangsu Pacific Meinuoke Biopharmaceutical Co., Ltd. (PMBP)

Protocol MPZ-II-02

Statistical Analysis Plan Page 45 of 82

- - 1. **PRIMARY ANALYSIS OF SECONDARY EFFICACY ENDPOINTS**

Considering no inferential interpretation will be made by secondary efficacy endpoints, statistical testing will not performed for all the secondary efficacy endpoints.

- - - 1. **‘Time-to-event’ Secondary Efficacy Endpoints**

Time-to-event secondary efficacy endpoints, including “Time from treatment start date to death” (Section 16.2.1.4), “Time to sustained recovery” (Section 16.2.1.5), will be analyzed using the same survival analysis model as in the primary analysis of the “Time-to-event” primary efficacy endpoint (refer to Section 16.1.3).

- - - 1. **‘Binary endpoints’ Secondary Efficacy Endpoints=**

‘Binary endpoints’ secondary efficacy endpoints, including “Response rate at Day 2, 8, 15” (Section 16.2.1.1), “Proportion of subjects alive and discharge without supplemental oxygen at Day 15 and Day 57” (Section 16.2.1.2), “Mortality at Day 15 and 57” (Section 16.2.1.3), “Incidence of new mechanical ventilation use” (Section 16.2.1.8), “Incidence of new mechanical ventilation use after mechanical ventilation extubation period of 24 hours” (Section 16.2.1.9), “Incidence of rehospitalization following hospital discharge” (Section 16.2.1.10) will be analyzed using the same CMH test and logistic regression model as in the primary analysis of the primary efficacy endpoint (refer to Section 16.1.3).

- - - 1. **Other Secondary Efficacy Endpoints**

Other secondary efficacy endpoints type not mentioned in Sections 16.2.3.1 and 16.2.3.2, including durations, will be summarized with descriptive statistics including mean, SD, median, min and max for continuous variables, and be displayed the number of subjects, and percentage, for categorical variables.

- - 1. **SENSITIVITY ANALYSES FOR SECONDARY EFFICACY ENDPOINTS**

No sensitivity analysis will be performed for secondary efficacy endpoints.

- - 1. **SUPPLEMENTARY ANALYSES FOR SECONDARY EFFICACY ENDPOINTS**

No supplementary analyses will be performed for secondary efficacy endpoints.

Document: \\ieedc-vnasc01\BIOSdata\Jiangsu_PMBP\Meplazumab\SZA62090\Biostatistics\Documentation\SAP\

| Author: Yueh Wang, Yang Teng | Version Number: Version Date: | 2.0  13-Dec-2021 |
| --- | --- | --- |
| Template No.: CS_TP_BS016 Revision 6 Effective Date: 02Dec2019 | Reference: | CS_WI_BS005 |

Copyright © 2009, 2010, 2012, 2016, 2018, 2019 IQVIA. All rights reserved. The contents of this document are confidential and proprietary to IQVIA Holdings Inc. and its subsidiaries. Unauthorized use, disclosure or reproduction is strictly prohibited.


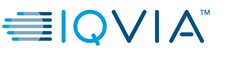
Jiangsu Pacific Meinuoke Biopharmaceutical Co., Ltd. (PMBP)

Protocol MPZ-II-02

Statistical Analysis Plan Page 46 of 82

**16.3. EXPLORATORY EFFICACY**

- - 1. **EXPLORATORY EFFICACY ENDPOINTS & DERIVATIONS**

Exploratory efficacy will only be applied to stage 2 subjects using the ITT analysis set.

- - - 1. **Ranked trajectory over 29 days**

Ranked outcome trajectory by Day 29 using the ordinal scale at Days 8, 15, and 29. For each subject across different groups, the below items are calculated and sorted,

- - - - 1. [Ascending order] The worst score over the 28 days and including Day 29;
        2. [Ascending order] The last recorded score;
        3. [Ascending order] The duration at the worst score
        4. [Ascending order] The best score that occurs after the worst score (this will equal to the worst score if the worst is the lowest).
        5. [Descending order] The duration of (4) is observed (the duration will be 0 if the worst score is the lowest one).

Rank by ascending order of 1, 2, 3, 4 then descending order of 5. Each of the orderings performed at steps 2, 3, 4 and 5 above are used to resolve any tied ranks resulting from the previous step, and each subject will have one overall rank for their trajectory compared to all other subjects (across both treatment arms). Lower ranks will represent a better trajectory.

- - 1. **ANALYSIS OF EXPLORATORY EFFICACY ENDPOINTS**
       1. **Analysis of ranked trajectory over 29 days**

The mean and SE, which is estimated using Rubin’s rules will be displayed for the ordinal scale ranking. The ranks of all subjects will be compared between different groups using the stratified Wilcoxon (van Elteren) test (using “Row mean scores differ” test in SAS PROC FREQ with the CMH option, with scores=modridit), stratified by, age group (age <65 years, versus age ≥65 years), and baseline severity grade.

**16.4. INTERIM ANALYSIS**

Dose selection at the interim analysis will be made by monitoring both the efficacy data including response rates of 3 dose arms, time to event endpoints, and safety data. The primary efficacy endpoint selection will be determined by

Document: \\ieedc-vnasc01\BIOSdata\Jiangsu_PMBP\Meplazumab\SZA62090\Biostatistics\Documentation\SAP\

| Author: Yueh Wang, Yang Teng | Version Number: Version Date: | 2.0  13-Dec-2021 |
| --- | --- | --- |
| Template No.: CS_TP_BS016 Revision 6 Effective Date: 02Dec2019 | Reference: | CS_WI_BS005 |

Copyright © 2009, 2010, 2012, 2016, 2018, 2019 IQVIA. All rights reserved. The contents of this document are confidential and proprietary to IQVIA Holdings Inc. and its subsidiaries. Unauthorized use, disclosure or reproduction is strictly prohibited.


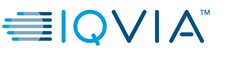
Jiangsu Pacific Meinuoke Biopharmaceutical Co., Ltd. (PMBP)

Protocol MPZ-II-02

Statistical Analysis Plan Page 47 of 82

the IDMC at the end of Stage 1 based on the data collected, which could depend on their clinical relevance and strength to detect the treatment difference between optimal dose and control. Interim analysis is the analysis of Stage 1 of the seamless two-stage design, and data collected at Stage 1 and Stage 2 will be analyzed separately so no alpha adjustment will be applied at each Stage.

- - 1. **INTERIM ANALYIS FOR SAMPLE SIZE RE-ASSESSMENT**

Sample size for Stage 2 will be re-evaluated based on Stage 1 results and the selected primary endpoint. The sample size estimation hypothesis and formula based on different endpoint types are described in Section 7.1.

- - 1. **INTERM ANALYSIS FOR DOSE SELECTION AND EFFICACY ENDPOINT SELECTION**

**(STAGE 1)**

Dose selection will be performed by comparing the three dose groups with the control group with Stage 1 results on efficacy and safety data, and PK data if available at Stage 1 cut-off. The details of the analyses can be found in Section 16.1.3. Pharmacodynamic/biomarker endpoints may be evaluated during the interim analysis at the end of Stage 1; however, if performed, the decision on dose selection will be based on the efficacy and safety results. Stage 2 interim analysis for efficacy will be determined and described after the primary endpoint is selected at Stage 1 interim analysis.

Primary efficacy endpoint selection for Stage 2 will be performed with Stage 1 results by evaluating both the clinical relevance of benefit/risk profile and statistical sensitivity of detecting treatment difference. The candidate efficacy endpoints are described in Section 16.1.1.

In addition to efficacy and safety data, few baseline information will be first displayed to better understand the characteristics of study subjects:

- - Subject disposition
  - Protocol deviations
  - Demographic and other baseline characteristics
  - Disease history
  - Study drug exposure

The efficacy data that will be summarized in the interim analysis include:

Document: \\ieedc-vnasc01\BIOSdata\Jiangsu_PMBP\Meplazumab\SZA62090\Biostatistics\Documentation\SAP\

| Author: Yueh Wang, Yang Teng | Version Number: Version Date: | 2.0  13-Dec-2021 |
| --- | --- | --- |
| Template No.: CS_TP_BS016 Revision 6 Effective Date: 02Dec2019 | Reference: | CS_WI_BS005 |

Copyright © 2009, 2010, 2012, 2016, 2018, 2019 IQVIA. All rights reserved. The contents of this document are confidential and proprietary to IQVIA Holdings Inc. and its subsidiaries. Unauthorized use, disclosure or reproduction is strictly prohibited.


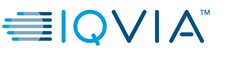
Jiangsu Pacific Meinuoke Biopharmaceutical Co., Ltd. (PMBP)

Protocol MPZ-II-02

Statistical Analysis Plan Page 48 of 82

- - Time to sustained clinical improvement or live discharge from hospital by Day 29
  - Response rate at Day 29 (also the dose selection endpoint)
  - Mortality at Day 29
  - Proportion of subjects alive and discharged without supplemental oxygen at Day 29 The safety data that will be summarized in the interim analysis include:
  - Treatment emergent adverse events (TEAEs) serious adverse event (SAE), and adverse events of special interest (AESIs)
    - Summary table and listing for TEAEs o Summary table and listing for SAEs o Summary table and listing for AESIs
  - Selected laboratory tests (hematology, chemistry, coagulation)
    - Observed and change from baseline tables by treatment arm of selected lab tests will be provided
    - Box and whisker plots over time by treatment arm in SI units for selected lab tests
  - Vital signs
    - Observed and change from baseline tables by treatment arm of vital signs parameters will be provided
    - Box and whisker plots over time by treatment group for vital signs parameters (see Section 17.5)
    - Listing for the vital signs abnormal values defined markedly abnormal criteria
  - Electrocardiogram (ECG)
    - Observed and change from baseline tables by treatment arm of ECG parameters will be provided
    - Box and whisker plots over time by treatment group of ECG parameters (see Section 17.6)
    - Listing for ECG parameters abnormal values defined markedly abnormal criteria

Document: \\ieedc-vnasc01\BIOSdata\Jiangsu_PMBP\Meplazumab\SZA62090\Biostatistics\Documentation\SAP\

| Author: Yueh Wang, Yang Teng | Version Number: Version Date: | 2.0  13-Dec-2021 |
| --- | --- | --- |
| Template No.: CS_TP_BS016 Revision 6 Effective Date: 02Dec2019 | Reference: | CS_WI_BS005 |

Copyright © 2009, 2010, 2012, 2016, 2018, 2019 IQVIA. All rights reserved. The contents of this document are confidential and proprietary to IQVIA Holdings Inc. and its subsidiaries. Unauthorized use, disclosure or reproduction is strictly prohibited.


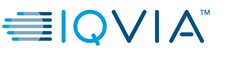
Jiangsu Pacific Meinuoke Biopharmaceutical Co., Ltd. (PMBP)

Protocol MPZ-II-02

Statistical Analysis Plan Page 49 of 82

1. **SAFETY ENDPOINTS**

All safety summaries will be presented by treatment arm based on the SAF analysis set for both Stage 1 and Stage 2 subjects. There will be no statistical comparisons between the treatment groups for safety data.

- 1. **ADVERSE EVENTS**
  - Prior AEs are defined as any AE that started or worsened in severity on or after the date of signed informed consent but before the date of randomization.
  - Treatment emergent AEs (TEAEs) are defined as any AE that started or worsened in severity on or after the date of randomization.

See APPENDIX 1 for judging the TEAE for AEs with partial dates. In the case where it is not possible to define an AE as treatment emergent or not, the AE will be classified by the worst case; i.e., treatment emergent.

AEs will be coded using the MedDRA dictionary, Version 23.1 or higher.

Worsening of an already recorded Adverse Event will be collected as a new event in eCRF. An overall summary of number and percentage of subjects within each of the categories described in the sub-sections below will be provided by treatment group based on the SAF analysis set. Should a subject experience multiple event within a category, the subject will be counted only once for that category. The frequency tables will be ordered by SOC and PT in descending order of the frequency by total subjects. For SOCs or PTs with the same frequency, categories will be sorted alphabetically.

All AEs (prior and TEAE) will be listed.

- - 1. **ALL TEAES**

Number and percentage of subjects with at least one TEAE will be presented by SOC and PT, and will be broken down further by maximum severity (Minor, Moderate and Severe) and relationship to study drug (related or not related).

- - - 1. **Severity**

Severity will be classified as mild, moderate and severe. TEAEs with a missing severity will be classified as severe.

Document: \\ieedc-vnasc01\BIOSdata\Jiangsu_PMBP\Meplazumab\SZA62090\Biostatistics\Documentation\SAP\

| Author: Yueh Wang, Yang Teng | Version Number: Version Date: | 2.0  13-Dec-2021 |
| --- | --- | --- |
| Template No.: CS_TP_BS016 Revision 6 Effective Date: 02Dec2019 | Reference: | CS_WI_BS005 |

Copyright © 2009, 2010, 2012, 2016, 2018, 2019 IQVIA. All rights reserved. The contents of this document are confidential and proprietary to IQVIA Holdings Inc. and its subsidiaries. Unauthorized use, disclosure or reproduction is strictly prohibited.


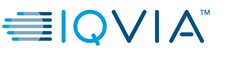
Jiangsu Pacific Meinuoke Biopharmaceutical Co., Ltd. (PMBP)

Protocol MPZ-II-02

Statistical Analysis Plan Page 50 of 82

Should a subject experience multiple events within a SOC or PT, only the subject’s worst severity will be counted for that SOC or PT. Number and percentage of subjects with at least one severe TEAE will further be presented by SOC and PT.

- - - 1. **Relationship to Study Drug**

Relationship to study drug, as indicated by the Investigator, will be classified as not related, unlikely related, possibly related, probably related or definitely related (increasing severity of relationship).

A “Related” TEAE is defined as a TEAE with a relationship to study drug of possibly related, probably related or definitely related while a “Non-related” TEAE is defined as a TEAE with a relationship to study drug of not related or unlikely related. TEAEs with a missing relationship to study drug will be regarded as related to study drug.

Should a subject experience multiple events within a SOC or PT, only the subject’s worst relationship will be counted for that SOC or PT. Number and percentage of subjects with at least one TEAE related to study drug will further be presented by SOC and PT.

- - - 1. **Relationship to Non-Study Treatment**

Relationship to non-study treatment, as indicated by the Investigator, will be classified as not related, unlikely related, possibly related, probably related or definitely related (increasing severity of relationship). “Related” is defined as a TEAE with a relationship to non-study drug of possibly related, probably related or definitely related while “Non-related” is defined as a TEAE with a relationship to non-study treatment of not related or unlikely related. TEAEs with a missing relationship to non-study treatment will be regarded as related to non-study treatment. The information will be displayed in the listing only.

- - - 1. **Relationship to Study Procedure**

Relationship to study procedure, as indicated by the Investigator, will be classified as not related, unlikely related, possibly related, probably related or definitely related (increasing severity of relationship). “Related” is defined as a TEAE with a relationship to study procedure of possibly related, probably related or definitely related while “Non- related” is defined as a TEAE with a relationship to study procedure of not related or unlikely related. TEAEs with a missing relationship to study procedure will be regarded as related to study procedure. The information will be displayed in the listing only.

Document: \\ieedc-vnasc01\BIOSdata\Jiangsu_PMBP\Meplazumab\SZA62090\Biostatistics\Documentation\SAP\

| Author: Yueh Wang, Yang Teng | Version Number: Version Date: | 2.0  13-Dec-2021 |
| --- | --- | --- |
| Template No.: CS_TP_BS016 Revision 6 Effective Date: 02Dec2019 | Reference: | CS_WI_BS005 |

Copyright © 2009, 2010, 2012, 2016, 2018, 2019 IQVIA. All rights reserved. The contents of this document are confidential and proprietary to IQVIA Holdings Inc. and its subsidiaries. Unauthorized use, disclosure or reproduction is strictly prohibited.


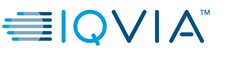
Jiangsu Pacific Meinuoke Biopharmaceutical Co., Ltd. (PMBP)

Protocol MPZ-II-02

Statistical Analysis Plan Page 51 of 82

- - - 1. **Action Taken with Study Treatment**

Action taken with study treatment will be collected in eCRF as “Dose Increase”, “Dose not changed”, “Dose reduced”, “Dose rate reduced”, “Drug interrupted”, “Drug withdrawal”, “Not applicable” or “Unknown” for each adverse event. The details information of each adverse event will be displayed in the listing.

- - - 1. **Outcome**

Outcome of each adverse event will be collected in eCRF as “Not recovered / not resolved”, “Recovered / resolved”, “Recovered / resolved with sequelae”, “Recovering / resolving”, “Unknown”, or “Fatal”. The details information of each adverse event will be displayed in the listing.

- - 1. **ADVERSE EVENTS WITH AN OUTCOME OF DEATH**

TEAEs with an outcome of death are those events which are recorded as “Fatal” outcome on the “Adverse Events” form of the eCRF. A summary of TEAEs with an outcome of death by SOC and PT will be prepared.

A listing of all AEs with an outcome of death will be provided.

- - 1. **SERIOUS ADVERSE EVENTS**

Serious adverse events (SAEs) are those events recorded as “Serious” on the “Adverse Events” form of the eCRF. A summary of serious TEAEs by SOC and PT will be prepared. Should a subject experience multiple events within a SOC or PT, the subject will be counted only once for that SOC or PT.

A listing of all SAEs will be provided.

- - 1. **TEAES LEADING TO PERMANENT DISCONTINUATION OF STUDY DRUG**

TEAEs leading to permanent discontinuation of study drug are those events recorded as “Drug withdrawal” for “Action Taken with study treatment” on the “Adverse Events” form of the eCRF. A summary of TEAEs leading to permanent discontinuation of study drug by SOC and PT will be prepared. Should a subject experience multiple events within a SOC or PT, the subject will be counted only once for that SOC or PT.

Document: \\ieedc-vnasc01\BIOSdata\Jiangsu_PMBP\Meplazumab\SZA62090\Biostatistics\Documentation\SAP\

| Author: Yueh Wang, Yang Teng | Version Number: Version Date: | 2.0  13-Dec-2021 |
| --- | --- | --- |
| Template No.: CS_TP_BS016 Revision 6 Effective Date: 02Dec2019 | Reference: | CS_WI_BS005 |

Copyright © 2009, 2010, 2012, 2016, 2018, 2019 IQVIA. All rights reserved. The contents of this document are confidential and proprietary to IQVIA Holdings Inc. and its subsidiaries. Unauthorized use, disclosure or reproduction is strictly prohibited.


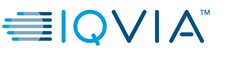
Jiangsu Pacific Meinuoke Biopharmaceutical Co., Ltd. (PMBP)

Protocol MPZ-II-02

Statistical Analysis Plan Page 52 of 82

- - 1. **ADVERSE EVENTS OF SPECIAL INTEREST**

AEs of special interest (AESIs) are:

- - Disease related secondary infection complications
  - Grade 4 (CTCAE V5) neutropenia and lymphopenia
  - Anaphylactic reactions defined by Clinical Criteria for Diagnosing Anaphylaxis
  - 20% decline in oxygen saturation (SpO2) between start and end of 1-hour study drug infusion
  - ALT or AST >3x ULN AND TBL>2x ULN
  - Evidence of RBC hemolysis as defined by 2 of the following 3 findings:
    - Anemia that is not due to another obvious cause;
    - Increased reticulocyte count that is not explained by an obvious cause,
    - Signs of RBC destruction, such as increased LDH, low haptoglobin ≤25 mg/dL, increased unconjugated bilirubin.

AESIs will be summarized by category of AESIs and PT.

- 1. **DEATHS**

If any subjects die during the study as recorded on the “Death Details” form of the eCRF, the number and percentage of subjects who died due to COVID-19 as primary cause of death and those who died due to any other primary cause will be summarized by treatment group based on the SAF analysis set. Similarly, the number and percentage of subjects who died due to COVID-19 as secondary cause of death and those who died due to any other secondary cause will be summarized by treatment group based on the SAF analysis set. Death due to COVID-19 will be identified by the study TMA in a blinded fashion before the study DBL.

A listing of all deaths will be provided.

Document: \\ieedc-vnasc01\BIOSdata\Jiangsu_PMBP\Meplazumab\SZA62090\Biostatistics\Documentation\SAP\

| Author: Yueh Wang, Yang Teng | Version Number: Version Date: | 2.0  13-Dec-2021 |
| --- | --- | --- |
| Template No.: CS_TP_BS016 Revision 6 Effective Date: 02Dec2019 | Reference: | CS_WI_BS005 |

Copyright © 2009, 2010, 2012, 2016, 2018, 2019 IQVIA. All rights reserved. The contents of this document are confidential and proprietary to IQVIA Holdings Inc. and its subsidiaries. Unauthorized use, disclosure or reproduction is strictly prohibited.


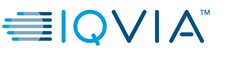
Jiangsu Pacific Meinuoke Biopharmaceutical Co., Ltd. (PMBP)

Protocol MPZ-II-02

Statistical Analysis Plan Page 53 of 82

- 1. **LABORATORY EVALUATIONS**

Fasting is not required before collection of laboratory samples, blood will be collected at the time points indicated in Section 3.2, and the testing will be performed at each clinical trial site in real time.

- - Clinical laboratory parameters include those from hematology, serum chemistry, lactate dehydrogenase (LDH), cardiac troponin, D -dimer, and ferritin as listed up in eCRF “Local Laboratory Results: Hematology”, “Local Laboratory Results: Chemistry”, “Local Laboratory Results: Coagulation” forms.
  - At screening subjects will be tested for Hepatitis B, Hepatitis C and Human Immunodeficiency Virus (HIV), and urinalysis.

Quantitative laboratory parameters reported as “< X”, i.e. below the lower limit of quantification (BLQ) or “> X”, i.e., above the upper limit of quantification (ULQ), will be converted to X for the purpose of quantitative summaries, but will be presented as recorded, i.e. as “< X” or “> X” in the listings.

The following summaries will be provided by treatment group based on the SAF analysis set for each of chemistry, hematology, and coagulation laboratory parameter:

- - Observed and change from baseline in Standard International (SI) units by visit;
  - Shift from baseline to the worst post-baseline observed value according to the Common Terminology Criteria for Adverse Events (CTCAE) toxicity grades (for quantitative parameters with available CTCAE toxicity grades; refer to Section 17.3.1)
  - Listing of subjects with at least one laboratory observed value meeting a CTCAE toxicity grade ≥3 (for quantitative parameters with available CTCAE toxicity grades; refer to Section 17.3.1)
  - Shifts from baseline to the highest/lowest post-baseline observed value according to normal range criteria (for quantitative parameters without CTCAE toxicity grades; refer to Section 17.3.2);
  - Number and percentage of subjects with maximum post-baseline ALT/AST observed value categorized as <3 x upper limit of normal (ULN), ≥3 to <5 x ULN, ≥5 to <10 x ULN or ≥10 ULN by maximum post-baseline total bilirubin observed value categorized as <2 x ULN or ≥2 x ULN;
  - Scatter plots of the maximum post-baseline observed value in ALT/AST value by the maximum post-baseline observed value in TBL value, both expressed as multiple of ULN.

All laboratory data will be listed.

Document: \\ieedc-vnasc01\BIOSdata\Jiangsu_PMBP\Meplazumab\SZA62090\Biostatistics\Documentation\SAP\

| Author: Yueh Wang, Yang Teng | Version Number: Version Date: | 2.0  13-Dec-2021 |
| --- | --- | --- |
| Template No.: CS_TP_BS016 Revision 6 Effective Date: 02Dec2019 | Reference: | CS_WI_BS005 |

Copyright © 2009, 2010, 2012, 2016, 2018, 2019 IQVIA. All rights reserved. The contents of this document are confidential and proprietary to IQVIA Holdings Inc. and its subsidiaries. Unauthorized use, disclosure or reproduction is strictly prohibited.


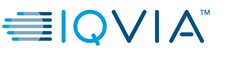
Jiangsu Pacific Meinuoke Biopharmaceutical Co., Ltd. (PMBP)

Protocol MPZ-II-02

Statistical Analysis Plan Page 54 of 82

- - 1. **CTCAE TOXICITY GRADES**

Quantitative laboratory parameters with available CTCAE toxicity grades will be categorized as follows where higher grades representing a more severe toxicity (refer to APPENDIX 3 for each parameter toxicity grade criteria):

- - Grade 1 (i.e., mild);
  - Grade 2 (i.e., moderate);
  - Grade 3 (i.e., severe)
  - Grade 4 (i.e., life-threatening)
  - Grade 5 (i.e., death)

Although not defined in the CTCAE toxicity grading system, version 5, non-missing laboratory parameter results not meeting any of the 5 grades defined in the CTCAE toxicity grading system will be categorized as ‘Grade 0’ for the purpose of the shift from baseline summaries.

- - 1. **LABORATORY NORMAL RANGES**

Quantitative laboratory parameters will be compared with the relevant laboratory normal ranges in SI units and categorized as:

- - Low: Below the lower limit of the laboratory normal range.
  - Normal: Within the laboratory normal range (upper and lower limit included).
  - High: Above the upper limit of the laboratory normal range.
    1. **SEROLOGY AND URINALYSIS AT SCREENING**

Results of serology tests performed (i.e., Hepatitis B, Hepatitis C, and HIV testing) and urinalysis at screening will be listed.

- 1. **COVID-19 VIROLOGICAL LOAD**

Qualitative and quantitative polymerase chain reaction [PCR] determination for COVID-19 in nasopharyngeal [NP]

Document: \\ieedc-vnasc01\BIOSdata\Jiangsu_PMBP\Meplazumab\SZA62090\Biostatistics\Documentation\SAP\

| Author: Yueh Wang, Yang Teng | Version Number: Version Date: | 2.0  13-Dec-2021 |
| --- | --- | --- |
| Template No.: CS_TP_BS016 Revision 6 Effective Date: 02Dec2019 | Reference: | CS_WI_BS005 |

Copyright © 2009, 2010, 2012, 2016, 2018, 2019 IQVIA. All rights reserved. The contents of this document are confidential and proprietary to IQVIA Holdings Inc. and its subsidiaries. Unauthorized use, disclosure or reproduction is strictly prohibited.


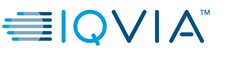
Jiangsu Pacific Meinuoke Biopharmaceutical Co., Ltd. (PMBP)

Protocol MPZ-II-02

Statistical Analysis Plan Page 55 of 82

swab will be assessed at baseline (Day 1 prior to infusion), Day 3, Day 5, Day 8 (prior to the second infusion), and on either Day 9 or Day 10 at the time of PD sample collection, and on Day 29.

- - Qualitative results: Number and percentage of subjects with a positive and negative result will be provided by treatment group and visit based on the SAF analysis set.
  - Quantitative results: Observed and change from baseline values in logarithmic form of COVID-19 viral load will be summarized by treatment group and visit based on the SAF analysis set.

All COVID-19 virological data will be listed.

- 1. **VITAL SIGNS**

The following vital sign parameters will be collected for this study as per the schedule of events (refer to protocol, Section 8.2.2):

- - Weight (kg)
  - Oral body temperature (Celsius scale with a thermometer)
  - Pulse rate (beats per minute [bpm])
  - Respiratory rate (breaths/min)
  - Systolic blood pressure (SBP) (mmHg)
  - Diastolic blood pressure (DBP) (mmHg)
  - Oxygen saturation (%)
  - Capillary Refill Time (seconds)

The following summaries will be provided by treatment group based on the SAF analysis set for each vital sign parameter:

- - Observed and change from baseline by visit;
  - Box and whisker plots over time by treatment group (for IAs only);
  - Number and percentages of subjects with at least one potentially clinically significant abnormality (PCSA) post

Document: \\ieedc-vnasc01\BIOSdata\Jiangsu_PMBP\Meplazumab\SZA62090\Biostatistics\Documentation\SAP\

| Author: Yueh Wang, Yang Teng | Version Number: Version Date: | 2.0  13-Dec-2021 |
| --- | --- | --- |
| Template No.: CS_TP_BS016 Revision 6 Effective Date: 02Dec2019 | Reference: | CS_WI_BS005 |

Copyright © 2009, 2010, 2012, 2016, 2018, 2019 IQVIA. All rights reserved. The contents of this document are confidential and proprietary to IQVIA Holdings Inc. and its subsidiaries. Unauthorized use, disclosure or reproduction is strictly prohibited.


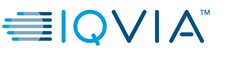
Jiangsu Pacific Meinuoke Biopharmaceutical Co., Ltd. (PMBP)

Protocol MPZ-II-02

Statistical Analysis Plan Page 56 of 82

-baseline value/change from baseline (refer to Section 17.5.1); All vital sign data will be listed.

**17.5.1. VITAL SIGNS MARKEDLY ABNORMAL CRITERIA**

Potentially clinically significant abnormality (PCSA) values are defined as abnormal values considered medically important by the Sponsor. Markedly abnormal vital sign observed values and/or change from baseline will be identified as PCSA in accordance with the following predefined markedly abnormal criteria:

| **Variable** | **Unit** | **Low** | **High** |
| --- | --- | --- | --- |
| SBP | mmHg | ≤ 90 mmHg AND  change from baseline ≤ -20 mmHg | ≥ 180 mmHg AND  change from baseline ≥ 20 mmHg |
| DBP | mmHg | ≤ 50 mmHg AND  change from ≤ -15 mmHg | ≥ 105 mmHg AND  change from baseline ≥ 15 mmHg |
| Pulse rate | bpm | ≤ 50 bpm AND  change from baseline ≤ -15 bpm | ≥ 120 bpm AND  change from baseline ≥ 15 bpm |
| Oxygen saturation | % | < 94 % | Not applicable |
| Body temperature | °C | Not applicable | ≥ 38.3 °C AND  change from baseline ≥ 1.1 °C |
| Weight | kg | Percent change from baseline  ≤ -7.0 % | Percent change from baseline  ≥ 7.0 % |

**17.6. ECG EVALUATIONS**

The following electrocardiogram (ECG) parameters will be measured for this study as per the schedule of events (refer to protocol, Section 8.2.3):

- - Heart rate (bpm);

Document: \\ieedc-vnasc01\BIOSdata\Jiangsu_PMBP\Meplazumab\SZA62090\Biostatistics\Documentation\SAP\

| Author: Yueh Wang, Yang Teng | Version Number: Version Date: | 2.0  13-Dec-2021 |
| --- | --- | --- |
| Template No.: CS_TP_BS016 Revision 6 Effective Date: 02Dec2019 | Reference: | CS_WI_BS005 |

Copyright © 2009, 2010, 2012, 2016, 2018, 2019 IQVIA. All rights reserved. The contents of this document are confidential and proprietary to IQVIA Holdings Inc. and its subsidiaries. Unauthorized use, disclosure or reproduction is strictly prohibited.


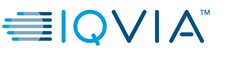
Jiangsu Pacific Meinuoke Biopharmaceutical Co., Ltd. (PMBP)

Protocol MPZ-II-02

Statistical Analysis Plan Page 57 of 82

- - PR interval (msec);
  - QRS interval (msec);
  - QT interval (msec);
  - Axis (degrees);
  - QTcF interval (msec);
  - QTcB interval (msec);
  - Overall ECG interpretation (Central interpretation):
    - Normal;
    - Abnormal, Insignificant (NCS);
    - Abnormal, significant (CS)

The following summaries will be provided by treatment group based on the SAF analysis set for each ECG parameter:

- - Observed and change from baseline by visit (for quantitative parameters);
  - Box and whisker plots over time by treatment group (for IAs only);
  - Number and percentages of subjects with at least one markedly abnormal post-baseline observed value/change from baseline (for quantitative parameters; refer to Section 17.6.1);
  - Shift from baseline in overall ECG interpretation to the worst post-baseline assessment; All ECG data will be listed.

**17.6.1. ECG MARKEDLY ABNORMAL CRITERIA**

Markedly abnormal quantitative ECG parameters will be identified in accordance with the following predefined markedly abnormal criteria:

- - Observed values for QT, QTcF, and QTcB intervals will be classified as:

Document: \\ieedc-vnasc01\BIOSdata\Jiangsu_PMBP\Meplazumab\SZA62090\Biostatistics\Documentation\SAP\

| Author: Yueh Wang, Yang Teng | Version Number: Version Date: | 2.0  13-Dec-2021 |
| --- | --- | --- |
| Template No.: CS_TP_BS016 Revision 6 Effective Date: 02Dec2019 | Reference: | CS_WI_BS005 |

Copyright © 2009, 2010, 2012, 2016, 2018, 2019 IQVIA. All rights reserved. The contents of this document are confidential and proprietary to IQVIA Holdings Inc. and its subsidiaries. Unauthorized use, disclosure or reproduction is strictly prohibited.


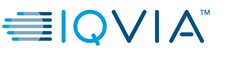
Jiangsu Pacific Meinuoke Biopharmaceutical Co., Ltd. (PMBP)

Protocol MPZ-II-02

Statistical Analysis Plan Page 58 of 82

- >470 msec;
- ≥501 msec
  - Change from baseline for QT, QTcF, and QTcB intervals will be classified as:
    - >30 msec increase from baseline
    - >60 msec increase from baseline

It is to be noted that the previous categories are not mutually exclusive, but cumulative. For example, if a subject worst post-baseline QT post-baseline observed value is 5100 mmHg, then this subject will be reported once under QT >470 msec and once under QT ≥501 msec.

**17.7. OTHER SAFETY ASSESSMENTS**

- - 1. **GENERAL PHYSICAL EXAMINATION**

Physical examinations will be conducted as per the schedule of events (refer to protocol section 1.3). At Screening a general physical examination will be performed. At subsequent visits a symptom-directed (targeted) physical examination will be performed to evaluate for any possible AE.

All physical examination data will be listed only.

- - 1. **RADIOGRAPHIC LUNG IMAGE**

Radiographic lung image (including X-ray, CT scan) imaging will be recorded if performed/available at screen or Day 1, or during course of hospitalization.

All Radiographic lung imaging data, including findings, will be listed only.

- - 1. **ANTIDRUG - ANTIBODIES**

Antibodies to meplazumab will be evaluated in serum samples collected from all subjects according to the Schedule of Events (protocol Section 3.2).

For each antibody, the number and percentage of subjects with a positive result at baseline and at any time after

Document: \\ieedc-vnasc01\BIOSdata\Jiangsu_PMBP\Meplazumab\SZA62090\Biostatistics\Documentation\SAP\

| Author: Yueh Wang, Yang Teng | Version Number: Version Date: | 2.0  13-Dec-2021 |
| --- | --- | --- |
| Template No.: CS_TP_BS016 Revision 6 Effective Date: 02Dec2019 | Reference: | CS_WI_BS005 |

Copyright © 2009, 2010, 2012, 2016, 2018, 2019 IQVIA. All rights reserved. The contents of this document are confidential and proprietary to IQVIA Holdings Inc. and its subsidiaries. Unauthorized use, disclosure or reproduction is strictly prohibited.


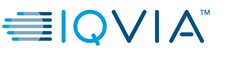
Jiangsu Pacific Meinuoke Biopharmaceutical Co., Ltd. (PMBP)

Protocol MPZ-II-02

Statistical Analysis Plan Page 59 of 82

baseline, and the ability to neutralize the activity of meplazumab will be provided by treatment group based and visit on the SAF analysis set.

All antibodies data will be listed.

- - 1. **THE NATIONAL EARLY WARNING SCORE (NEWS2)**

The National Early Warning score (NEWS2) has demonstrated an ability to discriminate subjects at risk of poor outcomes (RCP, 2017 [5]). This score is based on 7 clinical parameters, which is defined as below:


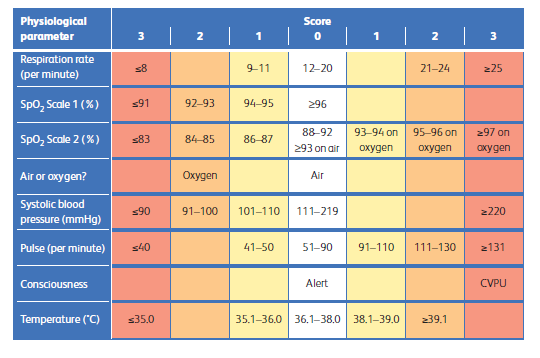


Abbreviations: C = confusion; P = arousable to pain; SpO2 = peripheral capillary oxygen saturation; U = unresponsive; V = arousable to voice.

© Royal College of Physicians 2017

The NEWS2 is one of the clinical assessment scales, and subject will be assessed according to Schedule of Events (Section 3.2)

NEWS2 data will be listed only.

- - 1. **PREGNANCY TEST**

The pregnancy test results will be listed if performed during the study period.

Document: \\ieedc-vnasc01\BIOSdata\Jiangsu_PMBP\Meplazumab\SZA62090\Biostatistics\Documentation\SAP\

| Author: Yueh Wang, Yang Teng | Version Number: Version Date: | 2.0  13-Dec-2021 |
| --- | --- | --- |
| Template No.: CS_TP_BS016 Revision 6 Effective Date: 02Dec2019 | Reference: | CS_WI_BS005 |

Copyright © 2009, 2010, 2012, 2016, 2018, 2019 IQVIA. All rights reserved. The contents of this document are confidential and proprietary to IQVIA Holdings Inc. and its subsidiaries. Unauthorized use, disclosure or reproduction is strictly prohibited.


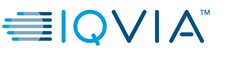
Jiangsu Pacific Meinuoke Biopharmaceutical Co., Ltd. (PMBP)

Protocol MPZ-II-02

Statistical Analysis Plan Page 60 of 82

- - 1. **COVID SYMPTOM ASSESSMENT**

COVID symptoms occurrence and severity, including shortness of breath, cough, fever, fatigue/malaise and myalgia will be assessed during the study period.

The COVID symptoms assessment results will be listed.

1. **PHARMACOKINETIC AND PHARMACODYNAMIC ENDPOINTS**

Derivation of the PK parameters from concentrations will be the responsibility of the clinical pharmacokineticist at IQVIA. The PK and PD summaries, listings, tables, and figures will be the responsibility of the PK/PD biostatistician at IQVIA.

Pharmacokinetic concentration summaries will be based on the PKS, PK parameters summaries will be based on the PKPS, and PD summaries will be based on the PDS. All PK/PD listings will be based on the SAF.

For qualitative variables, the population size (N for sample size and n for available data) and the percentage (of available data) for each class of the variable will be presented. Quantitative variables will be summarized using descriptive statistics, including N, n, mean, standard deviation (SD), coefficient of variation (CV), median, minimum, and maximum values. The CV will not be presented for change from baseline data. Geometric mean and geometric CV will be included for serum and blood cell PK parameters, where applicable. For the PK parameter tmax, only n, median, minimum, and maximum will be presented.

Pharmacokinetic concentrations and PD values will be listed and analyzed unrounded using the same precision as source data from the bioanalytical/clinical laboratory, regardless of how many decimal places or significant figures the data carry. Elapsed time variables will be analyzed and reported with 2 decimal places and unit of hours.

Pharmacokinetic parameters will be rounded for reporting purposes only in listings and will be statistically analyzed with unrounded precision (as allowable by software). The PK parameters will be reported in listings with 3 significant figures, except:

- - Parameters derived directly from the concentration data (e.g., Cmax) will be reported with the same precision as source concentration data
  - Elapsed time parameters (e.g., tmax) will be reported with precision of 2 decimal places with unit of hours The following rounding conventions will be used for reporting descriptive statistics of PK concentrations, PK

Document: \\ieedc-vnasc01\BIOSdata\Jiangsu_PMBP\Meplazumab\SZA62090\Biostatistics\Documentation\SAP\

| Author: Yueh Wang, Yang Teng | Version Number: Version Date: | 2.0  13-Dec-2021 |
| --- | --- | --- |
| Template No.: CS_TP_BS016 Revision 6 Effective Date: 02Dec2019 | Reference: | CS_WI_BS005 |

Copyright © 2009, 2010, 2012, 2016, 2018, 2019 IQVIA. All rights reserved. The contents of this document are confidential and proprietary to IQVIA Holdings Inc. and its subsidiaries. Unauthorized use, disclosure or reproduction is strictly prohibited.


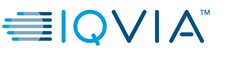
Jiangsu Pacific Meinuoke Biopharmaceutical Co., Ltd. (PMBP)

Protocol MPZ-II-02

Statistical Analysis Plan Page 61 of 82

parameters, and PD values, as applicable:

- - Mean, GeoMean, minimum, median, maximum: same precision as listed data
  - SD: 1 more digit of precision than listed data
  - CV, GeoCV: 1 decimal place as a percent

Ratios of means and any corresponding CIs for PK parameters (if generated in exploratory analyses) will be presented with two decimal places (as a percentage) to meet regulatory requirements. P-values, if any, shall be reported to four decimal places or as <0.0001.

Changes to the procedures or events which may impact the quality of the PK and/or PD data will be considered important protocol deviations or events and will be described within the clinical study report body text. These changes or events will include any circumstances that will alter the evaluation of the PK and/or PD. Examples of deviations/events for PK and/or PD include, but may not be limited to

- sample collection and/or processing errors that lead to inaccurate bioanalytical results
- missed PK and/or PD samples or deviation from scheduled sample collection at a critical time point.
- a missed dose or dose adjustment, and/or
- inaccurate dosing on one or both occasions

Important deviations/events may potentially affect an entire concentration-time profile (e.g. missed dose) or as little as a single data record (e.g. sample processing error). In the case of an important protocol deviation or event with impact on PK and/or PD, the affected PK and/or PD data will be excluded from the statistical analyses/summaries. Other changes to the procedures or events which do not impact the quality of the PK and/or PD data will not be considered important protocol deviations. Common examples of protocol deviations which are not considered to be important are a missed blood sample or deviations from blood collection times at a non-critical time in the profile. All excluded (flagged) data will be identified in the listings along with reason(s) for exclusion.

- 1. **PHARMACOKINETICS**

Pharmacokinetic endpoints:

- - Meplazumab serum and blood cell concentrations

Document: \\ieedc-vnasc01\BIOSdata\Jiangsu_PMBP\Meplazumab\SZA62090\Biostatistics\Documentation\SAP\

| Author: Yueh Wang, Yang Teng | Version Number: Version Date: | 2.0  13-Dec-2021 |
| --- | --- | --- |
| Template No.: CS_TP_BS016 Revision 6 Effective Date: 02Dec2019 | Reference: | CS_WI_BS005 |

Copyright © 2009, 2010, 2012, 2016, 2018, 2019 IQVIA. All rights reserved. The contents of this document are confidential and proprietary to IQVIA Holdings Inc. and its subsidiaries. Unauthorized use, disclosure or reproduction is strictly prohibited.


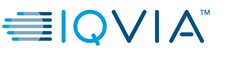
Jiangsu Pacific Meinuoke Biopharmaceutical Co., Ltd. (PMBP)

Protocol MPZ-II-02

Statistical Analysis Plan Page 62 of 82

- - Derived PK parameters (if calculable): may include but are not limited to maximum observed concentration (Cmax), time to Cmax (tmax), area under the concentration-time curve (AUC) calculated to the last quantifiable concentration and to infinity [AUC(0-last), AUC(0-inf)], elimination half-life (t1/2), volume of distribution (Vz, Vss), and systemic clearance (CL).

Pharmacokinetic concentrations for the control treatment will not be analyzed. Subjects/data will be analyzed according to actual treatment received.

- - 1. **MEPLAZUMAB SERUM AND BLOOD CELL CONCENTRATION DATA**

A listing of PK blood sample collection times as well as derived sampling time deviations will be provided.

Serum and blood cell concentrations will be listed based on the SAF and summarized based on the PKS by nominal time using descriptive statistics for each active treatment and Stage. Concentrations that are BLQ will be treated as zero for the computation of descriptive statistics. Predose and end-of-infusion samples collected outside the protocol-specified windows will be summarized except in the following situations:

- - - - The sample scheduled prior to the 2nd infusion is collected after start of the 2nd infusion or on a calendar day prior to the infusion day
      - A sample scheduled for the end of infusion is collected more than 15 minutes before the end of infusion (both serum and blood cell concentrations) or more than 15 minutes (serum) or 1 hour (blood cells) after the end of infusion

Sample collection deviations for all other scheduled samples will be evaluated on a case-by-case basis as to whether exclusions are warranted.

Plots of arithmetic mean (±SD on linear plot, as appropriate) meplazumab concentration-time for all treatments will be provided by analyte on a linear and semi-logarithmic scale, based on the PKS for each Stage. Results may be combined for both Stages, if appropriate. Plots of individual meplazumab concentration-time results will be provided by analyte on a linear and semi-logarithmic scale, based on the SAF. Individual plots will be generated using actual elapsed time of sample collection. Individual concentrations which are BLQ will be displayed as zero in the graphic presentations on linear scale; but will not be plotted on semi-logarithmic scale. Similarly, means which fall BLQ will be displayed as zero in the graphic presentations on linear scale, but will not be plotted on semi- logarithmic scale.

Meplazumab concentrations may be summarized and displayed by subgroups (anti-drug antibody [ADA] status;

Document: \\ieedc-vnasc01\BIOSdata\Jiangsu_PMBP\Meplazumab\SZA62090\Biostatistics\Documentation\SAP\

| Author: Yueh Wang, Yang Teng | Version Number: Version Date: | 2.0  13-Dec-2021 |
| --- | --- | --- |
| Template No.: CS_TP_BS016 Revision 6 Effective Date: 02Dec2019 | Reference: | CS_WI_BS005 |

Copyright © 2009, 2010, 2012, 2016, 2018, 2019 IQVIA. All rights reserved. The contents of this document are confidential and proprietary to IQVIA Holdings Inc. and its subsidiaries. Unauthorized use, disclosure or reproduction is strictly prohibited.


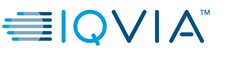
Jiangsu Pacific Meinuoke Biopharmaceutical Co., Ltd. (PMBP)

Protocol MPZ-II-02

Statistical Analysis Plan Page 63 of 82

gender; age; etc), if appropriate. If subgroup analyses are performed, data from both Stages may be combined for these analyses.

- - 1. **MEPLAZUMAB SERUM AND BLOOD PHARMACOKINETIC PARAMETERS**

The PK parameters in Table 1 will be estimated for meplazumab serum and blood cell concentrations by

non-compartmental methods using validated software and actual elapsed time from dosing (rounded to 2 decimal places). The PK parameters will be calculated for elapsed time from first dose across both dosing intervals, unless otherwise indicated below. A minimum of 3 quantifiable post-dose concentration-time data points will be required for calculation of PK parameters.

The following PK parameters will be computed, data permitting:

**Table 1. Pharmacokinetic Parameters**

Cmax Maximum observed concentration, obtained directly from the observed concentration versus time data. Calculated for first dosing interval and overall across both dosing intervals.

tmax Time of Cmax, obtained directly from the observed concentration versus time data.

Calculated for first dosing interval and overall across both dosing intervals.

AUC(0-inf) Area under the concentration-time curve from time zero extrapolated to infinite time, calculated by linear up/log down trapezoidal summation and extrapolated to infinity by addition of the observed last quantifiable concentration (Clastobs) divided by the terminal rate constant: AUC(0-last) + Clastobs/λz.

AUC(0-last) Area under the concentration-time curve from time zero to the time of the last quantifiable concentration, calculated by linear up/log down trapezoidal summation.

AUC(0-168) Area under the concentration-time curve from time zero to 168 hours, calculated by linear up/log down trapezoidal summation. Calculated for the first dosing interval only.

AUC(0-t) Area under the concentration-time curve from time zero to time t, calculated by linear up/log down trapezoidal summation. This parameter will only be calculated if warranted by the data, where time t is a common last quantifiable time point of

Document: \\ieedc-vnasc01\BIOSdata\Jiangsu_PMBP\Meplazumab\SZA62090\Biostatistics\Documentation\SAP\ Author: Yueh Wang, Yang Teng Version Number: 2.0

Version Date: 13-Dec-2021

Template No.: CS_TP_BS016 Revision 6 Reference: CS_WI_BS005 Effective Date: 02Dec2019

Copyright © 2009, 2010, 2012, 2016, 2018, 2019 IQVIA. All rights reserved. The contents of this document are confidential and proprietary to IQVIA Holdings Inc. and its subsidiaries. Unauthorized use, disclosure or reproduction is strictly prohibited.


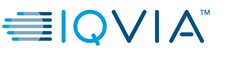
Jiangsu Pacific Meinuoke Biopharmaceutical Co., Ltd. (PMBP)

Protocol MPZ-II-02

Statistical Analysis Plan Page 64 of 82

interest, for the first dosing interval only.

CL Systemic clearance (L/h), calculated as:

- - - - 2 × dose/AUC(0-inf) for treatments receiving 2 active doses (meplazumab medium or high dose), or
      - dose/AUC(0-inf) for treatments receiving 1 active dose (meplazumab low dose)

λz Terminal rate constant (1/h), determined by linear regression of the terminal points of the log-linear concentration-time curve.

t1/2 Elimination half-life (h), determined as (ln2/λz).

Vss Volume of distribution at steady state following intravenous dosing (L), calculated as mean residence time (extrapolated to infinity) multiplied by systemic clearance.

Vz Volume of distribution (L), estimated by dividing the systemic clearance by λz.

In accordance with molecular formula and formulation for meplazumab, no dose adjustment calculations will be made for any molecular conversions between administered drug product and analyte measure.

In subjects with sufficient serum and/or blood cell meplazumab data, an attempt will be made to calculate PK parameters following the infusion regimen. Subjects with partial data will be evaluated on a case-by-case basis to determine if sufficient data are available for reliable estimation of PK parameters.

For PK parameter calculations for first dose, concentrations at time 0 hour (predose first dose) will be assigned a numerical value of zero. Otherwise, for calculating parameters, no other imputation/substitution will be made for missing predose and/or trough concentrations. For all dosing intervals, any concentrations that are BLQ (including predose before the 2nd infusion) will be assigned a value of zero if they precede quantifiable samples in the initial portion of the profile. A BLQ value that occurs between quantifiable data points, especially prior to Cmax, will be evaluated to determine if an assigned concentration of zero makes sense, or if exclusion of the data is warranted. Following Cmax (first infusion) and a quantifiable post-dose concentration after start of the 2nd infusion, BLQ values embedded between 2 quantifiable data points will be treated as missing when calculating PK parameters. If a BLQ value occurs at the end of the collection interval (after the last quantifiable concentration), it will be set to zero. If consecutive BLQ concentrations are followed by quantifiable concentration(s) in the terminal portion of the concentration curve, these quantified values will be excluded from the PK analysis by setting them to missing,

Document: \\ieedc-vnasc01\BIOSdata\Jiangsu_PMBP\Meplazumab\SZA62090\Biostatistics\Documentation\SAP\

| Author: Yueh Wang, Yang Teng | Version Number: Version Date: | 2.0  13-Dec-2021 |
| --- | --- | --- |
| Template No.: CS_TP_BS016 Revision 6 Effective Date: 02Dec2019 | Reference: | CS_WI_BS005 |

Copyright © 2009, 2010, 2012, 2016, 2018, 2019 IQVIA. All rights reserved. The contents of this document are confidential and proprietary to IQVIA Holdings Inc. and its subsidiaries. Unauthorized use, disclosure or reproduction is strictly prohibited.


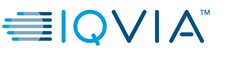
Jiangsu Pacific Meinuoke Biopharmaceutical Co., Ltd. (PMBP)

Protocol MPZ-II-02

Statistical Analysis Plan Page 65 of 82

unless otherwise warranted by the concentration-time profile.

Pharmacokinetic parameters will be listed (FAS) and summarized (PKPS) by treatment and Stage, as appropriate. Subgroup analyses as described for meplazumab concentrations may also be performed. Data permitting, plots of individual and geometric mean PK parameters [Cmax and AUC(0-inf)] versus dose will be provided, based on the PKPS. A similar plot will be constructed for AUC(0-last) or, if more appropriate, a partial AUC that is common to most of the subjects [AUC(0-t)].

The following PK parameters (Table 33) will be calculated for diagnostic purposes, as appropriate, to assess reliability of the PK parameter estimates. These parameters will be listed but will not be summarized.

**Table 3. Calculation of PK Parameters for Diagnostic Purposes**

**PK Parameter Definition**

λZ_low The starting time point (h) of the time interval of the log-linear regression to determine λZ.

λZ_upp The ending time point (h) of the time interval of the log-linear regression to determine λZ.

t1/2, Interval The time interval duration (h) of the log-linear regression to determine λZ, calculated as λZ_upp − λZ_low.

t1/2, N Number of data points included in the log-linear regression analysis to determine λZ. A minimum of 3 data points will be used for determination.

Rsq_adj Adjusted coefficient of determination for calculation of λZ. If Rsq_adj

<0.800, then λZ and parameters derived from it will be listed but flagged and excluded from summaries.

%AUCex Percentage of AUC(0-inf) obtained by extrapolation, calculated as [(Clast/λz)/AUC(0-inf)×100]. If the %AUCex is greater than 30.0% of AUC(0-inf), then AUC(0-inf) will be listed but flagged and excluded from summaries.

Protocol deviations or events that have potential to affect the PK/PD are described above. In addition, the following may lead to exclusion of PK parameters from the PKPS as follows. It should be noted that a protocol deviation and/or event may lead to unreliable PK results for only one matrix (e.g., serum) but not the other (e.g., blood cells):

Document: \\ieedc-vnasc01\BIOSdata\Jiangsu_PMBP\Meplazumab\SZA62090\Biostatistics\Documentation\SAP\

| Author: Yueh Wang, Yang Teng | Version Number: Version Date: | 2.0  13-Dec-2021 |
| --- | --- | --- |
| Template No.: CS_TP_BS016 Revision 6 Effective Date: 02Dec2019 | Reference: | CS_WI_BS005 |

Copyright © 2009, 2010, 2012, 2016, 2018, 2019 IQVIA. All rights reserved. The contents of this document are confidential and proprietary to IQVIA Holdings Inc. and its subsidiaries. Unauthorized use, disclosure or reproduction is strictly prohibited.


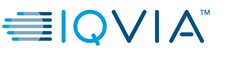
Jiangsu Pacific Meinuoke Biopharmaceutical Co., Ltd. (PMBP)

Protocol MPZ-II-02

Statistical Analysis Plan Page 66 of 82

- - *Dosing errors, inaccurate dosing, and/or missed dose(s):* all PK parameters that are associated with the affected dose. Parameters will be calculated, if possible, but excluded from summaries and exploratory analyses.
  - *End-of-infusion sample collected outside the window described in Section 18.1.1*, unless the concentration results indicate that the deviation is unlikely to affect the reliability of the PK parameter (affected parameters Cmax and/or AUC). Parameters will be calculated, if possible, but excluded from summaries and exploratory analyses. The affected subject PK parameters will be identified in the listings.
  - *Predose sample (2nd infusion) collected after start of the infusion*: the AUCs across both dosing intervals (and any parameters derived from the AUC) will not be calculated.
  - *Predose sample for the 2nd dosing interval is missing:* the AUCs across both dosing intervals (and any parameters derived from the AUC) will not be calculated for blood cell concentrations. For serum meplazumab concentrations, if results in other subjects indicate that concentrations return to BLQ, the missing predose sample concentration may be set to zero for AUC calculation. Handling of these situations will be described in the clinical study report (CSR) and affected subject PK parameters will be identified in the listings.
  - *Other missed samples or sample handling and/or processing errors that lead to missing data*: when appropriate, an attempt will be made to calculate PK parameters; however, unreliable PK data will be excluded from summaries and/or exploratory analyses. The affected subject PK parameters will be identified in the listings.

# PHARMACODYNAMICS

Pharmacodynamic endpoints:

- Changes from baseline in cytokines and chemokines related to inflammatory and immune status including but not limited to CyPA, IL-6, IL-8, MCP-1, MIP-1α, MIP-1β, TNF-α, IFN-γ, IL-1 RA, IL-2, IL-2Rα, IL-4, IL-7, IL-10, IL-15, IL-17A, IL-12 p70, G-CSF, M-CSF, CXCL10, hsCRP

A listing of PD blood sample collection times as well as derived sampling time deviations will be provided.

Observed results and change-from-baseline PD and biomarker exploratory results will be listed and summarized by Stage, treatment, and nominal time point, as available and appropriate. Additional variables, e.g., percent change from baseline and/or ratio to baseline, may be listed/summarized as appropriate. Pharmacodynamic values that are BLQ will be treated as ½ × the lower limit of quantification for the computation of summary statistics and baseline correction. Pharmacodynamic and biomarker exploratory analyses may be presented separately from the main CSR. Arithmetic mean change from baseline results for select PD endpoints may be graphically displayed on linear scale, Document: \\ieedc-vnasc01\BIOSdata\Jiangsu_PMBP\Meplazumab\SZA62090\Biostatistics\Documentation\SAP\

| Author: Yueh Wang, Yang Teng | Version Number: Version Date: | 2.0  13-Dec-2021 |
| --- | --- | --- |
| Template No.: CS_TP_BS016 Revision 6 Effective Date: 02Dec2019 | Reference: | CS_WI_BS005 |

Copyright © 2009, 2010, 2012, 2016, 2018, 2019 IQVIA. All rights reserved. The contents of this document are confidential and proprietary to IQVIA Holdings Inc. and its subsidiaries. Unauthorized use, disclosure or reproduction is strictly prohibited.


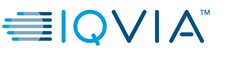
Jiangsu Pacific Meinuoke Biopharmaceutical Co., Ltd. (PMBP)

Protocol MPZ-II-02

Statistical Analysis Plan Page 67 of 82

as appropriate.

- 1. **EXPOSURE RESPONSE CORRELATIONS**

Meplazumab exposure versus (PD and/or efficacy) response variables may be graphically and statistically displayed in the CSR for select endpoints, as appropriate.

Modeling and simulation of PK results relationships may be also performed. Exposure and response data obtained from this study may be modeled and/or combined with data from other studies and used for modeling and simulations. If modeling and/or simulations are performed, a modeling and simulation data analysis plan will be prepared and the results will be reported separately from the main CSR.

1. **DATA NOT SUMMARIZED OR PRESENTED**

Data that will not be summarized or listed are:

- - Comments

These data will not be summarized or listed but will be available in the Study Data Tabulation Model (SDTM) and/or Analysis Dataset Modelling (ADaM) datasets.

Document: \\ieedc-vnasc01\BIOSdata\Jiangsu_PMBP\Meplazumab\SZA62090\Biostatistics\Documentation\SAP\

| Author: Yueh Wang, Yang Teng | Version Number: Version Date: | 2.0  13-Dec-2021 |
| --- | --- | --- |
| Template No.: CS_TP_BS016 Revision 6 Effective Date: 02Dec2019 | Reference: | CS_WI_BS005 |

Copyright © 2009, 2010, 2012, 2016, 2018, 2019 IQVIA. All rights reserved. The contents of this document are confidential and proprietary to IQVIA Holdings Inc. and its subsidiaries. Unauthorized use, disclosure or reproduction is strictly prohibited.


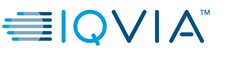
Jiangsu Pacific Meinuoke Biopharmaceutical Co., Ltd. (PMBP)

Protocol MPZ-II-02

Statistical Analysis Plan Page 68 of 82

1. **REFERENCES**

Brookmeyer R, Crowley J. A (1982) Confidence Interval for the Median Survival Time. Biometrics 38:29-41.

Bian, H., Zheng, ZH., Wei, D. et al. Safety and efficacy of meplazumab in healthy volunteers and COVID-19 patients: a randomized phase 1 and an exploratory phase 2 trial. Sig Transduct Target Ther 6, 194 (2021). https://doi.org/10.1038/s41392-021-00603-6

Chow S, Shao J, Wang H. 2008. Sample Size Calculations in Clinical Research. 2nd Ed. Chapman & Hall/CRC Biostatistics Series. page 89.

Chuang-Stein C (1992). Summarizing laboratory data with different reference ranges in multi-center clinical trials. Drug Information Journal 26(1);77-84.

Lawless JF (1982) Statistical Models and Methods for Lifetime Data, pages 365-366. John Wiley and Sons. Marik PE, Taeb AM (2017). SIRS, qSOFA and new sepsis definition. J Thorac Dis 9(4);943-945.

Prytherch DR, Smith GB, Schmidt PE, Featherstone PI (2010). ViEWS – Towards a national early warning score for detecting adult inpatient deterioration. Resuscitation 81(8);932-937

RCP (2017). National Early Warning Score (NEWS) 2. Royal College of Physicians. [https://www.rcplondon.ac.uk/projects/outputs/national-early-warning-score-news-2,](https://www.rcplondon.ac.uk/projects/outputs/national-early-warning-score-news-2) Accessed April 7th, 2020

Siddiqui O, Hung HMJ, O’Neill R (2009) MMRM vs. LOCF: A comprehensive comparison based on simulation study and 25 NDA datasets. J Biopharm Stat 19:227–246. <https://doi.org/10.1080/10543400802609797>

Smith GB, Prytherch DR, Meredith P, Schmidt PE, Featherstone PI (2013). The ability of the National Early Warning Score (NEWS) to discriminate patients at risk of early cardiac arrest, unanticipated intensive care unit admission, and death. Resuscitation 84(4);465-470

Tan TL, Tang YJ, Ching LJ, Abdullah N, Neon HM (2018). Comparison of prognostic accuracy of the quick sepsis- related organ failure assessment between short- & long-term mortality in patients presenting outside of the intensive care unit – a systematic review & meta-analysis. Sci Rep. 8(1);16698.

Therneau TM, Grambsch PM (2000). Modeling Survival Data: Extending the Cox Model. Springer New York.

Document: \\ieedc-vnasc01\BIOSdata\Jiangsu_PMBP\Meplazumab\SZA62090\Biostatistics\Documentation\SAP\

| Author: Yueh Wang, Yang Teng | Version Number: Version Date: | 2.0  13-Dec-2021 |
| --- | --- | --- |
| Template No.: CS_TP_BS016 Revision 6 Effective Date: 02Dec2019 | Reference: | CS_WI_BS005 |

Copyright © 2009, 2010, 2012, 2016, 2018, 2019 IQVIA. All rights reserved. The contents of this document are confidential and proprietary to IQVIA Holdings Inc. and its subsidiaries. Unauthorized use, disclosure or reproduction is strictly prohibited.


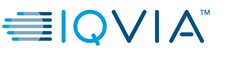
Jiangsu Pacific Meinuoke Biopharmaceutical Co., Ltd. (PMBP)

Protocol MPZ-II-02

Statistical Analysis Plan Page 69 of 82

Document: \\ieedc-vnasc01\BIOSdata\Jiangsu_PMBP\Meplazumab\SZA62090\Biostatistics\Documentation\SAP\

| Author: Yueh Wang, Yang Teng | Version Number: Version Date: | 2.0  13-Dec-2021 |
| --- | --- | --- |
| Template No.: CS_TP_BS016 Revision 6 Effective Date: 02Dec2019 | Reference: | CS_WI_BS005 |

Copyright © 2009, 2010, 2012, 2016, 2018, 2019 IQVIA. All rights reserved. The contents of this document are confidential and proprietary to IQVIA Holdings Inc. and its subsidiaries. Unauthorized use, disclosure or reproduction is strictly prohibited.


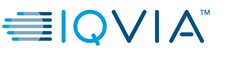
Jiangsu Pacific Meinuoke Biopharmaceutical Co., Ltd. (PMBP)

Protocol MPZ-II-02

Statistical Analysis Plan Page 70 of 82

APPENDIX 1. **PARTIAL DATE CONVENTIONS**

**ALGORITHM FOR TREATMENT EMERGENCE OF ADVERSE EVENTS**

| **START DATE** | **END DATE** | **ACTION** |
| --- | --- | --- |
| Known | Known/Partial/ Missing | If AE start date < randomization date then not TEAE;  If AE start date = date of randomization and time of onset of AE is available and is before the time of randomization then not a TEAE;  Otherwise, TEAE. |
|  |  |  |
| Partial, but known components show that AE started before randomization date | Known/Partial/ Missing | Not TEAE. |
|  |  |  |
| Partial and known components show that AE started on or after study drug start date  OR  Missing | Known | If AE end date < randomization, then not TEAE; Otherwise, TEAE. |
|  | Partial | If known components of AE end date show that AE stopped before randomization date), then not TEAE;  Otherwise, TEAE. |
|  | Missing | Assume TEAE. |

**ALGORITHM FOR PRIOR / CONCOMITANT MEDICATIONS**

| **START DATE** | **STOP DATE** | **ACTION** |
| --- | --- | --- |
| Known | Known or ongoing | If medication stop date < randomization date, assign as prior; Otherwise, assign as concomitant; |

Document: \\ieedc-vnasc01\BIOSdata\Jiangsu_PMBP\Meplazumab\SZA62090\Biostatistics\Documentation\SAP\

| Author: Yueh Wang, Yang Teng | Version Number: Version Date: | 2.0  13-Dec-2021 |
| --- | --- | --- |
| Template No.: CS_TP_BS016 Revision 6 Effective Date: 02Dec2019 | Reference: | CS_WI_BS005 |

Copyright © 2009, 2010, 2012, 2016, 2018, 2019 IQVIA. All rights reserved. The contents of this document are confidential and proprietary to IQVIA Holdings Inc. and its subsidiaries. Unauthorized use, disclosure or reproduction is strictly prohibited.


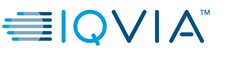
Jiangsu Pacific Meinuoke Biopharmaceutical Co., Ltd. (PMBP)

Protocol MPZ-II-02

Statistical Analysis Plan Page 71 of 82

| **START DATE** | **STOP DATE** | **ACTION** |
| --- | --- | --- |
|  | Partial | If known components of medication stop date show that medication stopped before randomization date, assign as prior;  Otherwise, assign as concomitant. |
|  | Missing, not ongoing | If medication stop date is missing, then it can never be assigned as prior only; assign as concomitant. |
|  |  |  |
| Partial | Known or ongoing | If medication stop date < randomization date, assign as prior;  Otherwise, assign as concomitant. |
|  | Partial | If known components of medication stop date show that medication stopped before randomization date, assign as prior;  Otherwise, assign as concomitant. |
|  | Missing, not  ongoing | Cannot be assigned as prior; assign as concomitant. |
|  |  |  |
| Missing | Known or ongoing | If medication stop date < randomization date, assign as prior;  Otherwise, assign as concomitant. |
|  | Partial | If known components of medication stop date show that medication stopped before randomization date, assign as prior;  Otherwise, assign as concomitant. |
|  | Missing, not ongoing | Assign as concomitant. |

Document: \\ieedc-vnasc01\BIOSdata\Jiangsu_PMBP\Meplazumab\SZA62090\Biostatistics\Documentation\SAP\

| Author: Yueh Wang, Yang Teng | Version Number: Version Date: | 2.0  13-Dec-2021 |
| --- | --- | --- |
| Template No.: CS_TP_BS016 Revision 6 Effective Date: 02Dec2019 | Reference: | CS_WI_BS005 |

Copyright © 2009, 2010, 2012, 2016, 2018, 2019 IQVIA. All rights reserved. The contents of this document are confidential and proprietary to IQVIA Holdings Inc. and its subsidiaries. Unauthorized use, disclosure or reproduction is strictly prohibited.


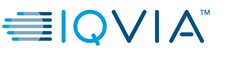
Jiangsu Pacific Meinuoke Biopharmaceutical Co., Ltd. (PMBP)

Protocol MPZ-II-02

Statistical Analysis Plan Page 72 of 82

APPENDIX 2. **PROGRAMMING CONVENTIONS FOR OUTPUTS**

**DATES & TIMES**

Depending on data available, dates and times will take the form yyyy-mm-dd hh:mm:ss.

**SPELLING FORMAT**

English US.

**PAPER SIZE, ORIENTATION, AND MARGINS**

The size of paper will be letter and the page orientation will be landscape. Margins will provide at least 1 inch (2.54 centimeters) of white space all around the page.

**FONTS**

The font type ‘Courier New’ will be used, with a font size of 8. The font color will be black with no bolding, underlining, italics or subscripting.

**PRESENTATION OF TREATMENT GROUPS**

For outputs, treatment groups will be represented as follows and in the given order:

| **Treatment Group** | **Tables and Graphs** | **Listings** |
| --- | --- | --- |
| Meplazumab low-dose + SoC | 1 | 1 |
| Meplazumab medium-dose + SoC | 2 | 2 |
| Meplazumab high-dose +SoC | 3 | 3 |
| Meplazumab placebo +SoC | 4 | 4 |
| Total for Stage 1 (Meplazumab low-dose + medium-dose + high-dose + control) [1] | 5 | NA |

Document: \\ieedc-vnasc01\BIOSdata\Jiangsu_PMBP\Meplazumab\SZA62090\Biostatistics\Documentation\SAP\

| Author: Yueh Wang, Yang Teng | Version Number: Version Date: | 2.0  13-Dec-2021 |
| --- | --- | --- |
| Template No.: CS_TP_BS016 Revision 6 Effective Date: 02Dec2019 | Reference: | CS_WI_BS005 |

Copyright © 2009, 2010, 2012, 2016, 2018, 2019 IQVIA. All rights reserved. The contents of this document are confidential and proprietary to IQVIA Holdings Inc. and its subsidiaries. Unauthorized use, disclosure or reproduction is strictly prohibited.


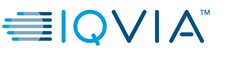
Jiangsu Pacific Meinuoke Biopharmaceutical Co., Ltd. (PMBP)

Protocol MPZ-II-02

Statistical Analysis Plan Page 73 of 82

| **Treatment Group** | **Tables and Graphs** | **Listings** |
| --- | --- | --- |
| Total for Stage 2 (Meplazumab selected dose+ control) [1] | 6 | NA |
| Randomized, Not Treated | n/a | 5 |
| Screen Failure | n/a | 6 |

[1] Not applicable for efficacy tables, safety tables and graphs.

**PRESENTATION OF NOMINAL VISITS**

For outputs, analysis visits will be represented as follows and in that order:

| **Long Name (default)** | **Short Name** |
| --- | --- |
| Screening | Scrn |
| Baseline | Base |
| Day x, where x = 1 to 29 | Day x, where x= 1 to 29 |
| Follow-up | FU |
| End-of-study | EOS |

**DESCRIPTIVE STATISTICS**

If the original data has N decimal places, then the summary statistics will have the following decimal places:

- Minimum and maximum: N;
- Mean, median, lower and upper bounds of two-sided 95% CI: N + 1;
- SD and SE: N + 2

Document: \\ieedc-vnasc01\BIOSdata\Jiangsu_PMBP\Meplazumab\SZA62090\Biostatistics\Documentation\SAP\

| Author: Yueh Wang, Yang Teng | Version Number: Version Date: | 2.0  13-Dec-2021 |
| --- | --- | --- |
| Template No.: CS_TP_BS016 Revision 6 Effective Date: 02Dec2019 | Reference: | CS_WI_BS005 |

Copyright © 2009, 2010, 2012, 2016, 2018, 2019 IQVIA. All rights reserved. The contents of this document are confidential and proprietary to IQVIA Holdings Inc. and its subsidiaries. Unauthorized use, disclosure or reproduction is strictly prohibited.


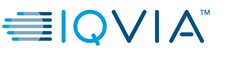
Jiangsu Pacific Meinuoke Biopharmaceutical Co., Ltd. (PMBP)

Protocol MPZ-II-02

Statistical Analysis Plan Page 74 of 82

**PERCENTAGES**

Percentages will be reported to one decimal place. Rounding will be applied, except for percentages < 0.1 but > 0.0 which will be presented as ‘< 0.1’ and percentages < 100.0 but >99.9 which will be presented as ‘>99.9’. Where counts are zero, no percentages will appear in the output.

**P-VALUES**

P-values will be reported to three decimal places. Rounding will be applied, except for the p-values < 0.001 which will be presented as ‘< 0.001’ and p-values < 1.000 but > 0.999 which will be presented as ‘> 0.999’.

**LISTINGS**

All listings will be ordered by the following (unless otherwise indicated in the output template):

- - Randomized treatment group (or treatment received if it’s a safety output);
  - Subject ID;
  - Parameter, when applicable;
  - Date/Time, when applicable;
  - Timepoint, when applicable.

Document: \\ieedc-vnasc01\BIOSdata\Jiangsu_PMBP\Meplazumab\SZA62090\Biostatistics\Documentation\SAP\

| Author: Yueh Wang, Yang Teng | Version Number: Version Date: | 2.0  13-Dec-2021 |
| --- | --- | --- |
| Template No.: CS_TP_BS016 Revision 6 Effective Date: 02Dec2019 | Reference: | CS_WI_BS005 |

Copyright © 2009, 2010, 2012, 2016, 2018, 2019 IQVIA. All rights reserved. The contents of this document are confidential and proprietary to IQVIA Holdings Inc. and its subsidiaries. Unauthorized use, disclosure or reproduction is strictly prohibited.


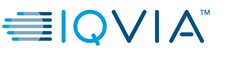
Jiangsu Pacific Meinuoke Biopharmaceutical Co., Ltd. (PMBP)

Protocol MPZ-II-02

Statistical Analysis Plan Page 75 of 82

APPENDIX 3. **CTCAE TOXICITY GRADE, VERSION 5.0**

<https://ctep.cancer.gov/protocolDevelopment/electronic_applications/ctc.htm>(accessed on 22-Apr-2020)

| **CTCAE Term** | **Laboratory Test** | **No Event** | **Grade 1** | **Grade 2** | **Grade 3** | **Grade 4** | **Grade 5** |
| --- | --- | --- | --- | --- | --- | --- | --- |
| Anemia | Hemoglobin (g/L) | ≥ LLN | ≥ 100 g/L -  < LLN | ≥ 80 -  < 100 g/L | < 80 g/L | n/a | n/a |
| Hemoglobin increased | Hemoglobin (g/L) | No increase from baseline | Increase from baseline > 0 - ≤ 20 g/L | Increase from baseline > 20 -  ≤ 40 g/L | Increase from baseline  > 40 g/L | n/a | n/a |
| Platelet count decreased | Platelet count (x10E9/L) | ≥ LLN | ≥ 75 x 10E9/L  – < LLN | ≥ 50 -  < 75 x 10E9/L | ≥ 25 -  < 50 x 10E9/L | < 25 x 10E9/L | n/a |
| CD4 lymphocytes decreased | CD4 T-cell count (x 10E9/L) | ≥ LLN | ≥ 0.5 x 10E9/L  – < LLN | ≥ 0.2 -  < 0.5 x 10E9/L | ≥ 0.05 -  < 0.2 x 10E9/L | < 0.05 x 10E9/L | n/a |
| White blood cell (WBC) decreased | WBC (x 10E9/L) | ≥ LLN | ≥ 3.0 x 10E9/L  – < LLN | ≥ 2.0 -  < 3.0 x 10E9/L | ≥ 1.0 - < 2.0 x 10E9/L | < 1.0 x 10E9/L | n/a |
| Leukocytosis | WBC (x 10E9/L) | ≤ 100 x 10E9/L | n/a | n/a | > 100 x 10E9/L | n/a | n/a |

Document: \\ieedc-vnasc01\BIOSdata\Jiangsu_PMBP\Meplazumab\SZA62090\Biostatistics\Documentation\SAP\

| Author: Yueh Wang, Yang Teng | Version Number: Version Date: | 2.0  13-Dec-2021 |
| --- | --- | --- |
| Template No.: CS_TP_BS016 Revision 6 Effective Date: 02Dec2019 | Reference: | CS_WI_BS005 |

Copyright © 2009, 2010, 2012, 2016, 2018, 2019 IQVIA. All rights reserved. The contents of this document are confidential and proprietary to IQVIA Holdings Inc. and its subsidiaries. Unauthorized use, disclosure or reproduction is strictly prohibited.


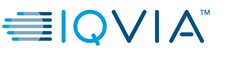
Jiangsu Pacific Meinuoke Biopharmaceutical Co., Ltd. (PMBP)

Protocol MPZ-II-02

Statistical Analysis Plan Page 76 of 82

| **CTCAE Term** | **Laboratory Test** | **No Event** | **Grade 1** | **Grade 2** | **Grade 3** | **Grade 4** | **Grade 5** |
| --- | --- | --- | --- | --- | --- | --- | --- |
| Absolute neutrophils count decreased | Absolute neutrophils count (x 10E9/L) | ≥ LLN | ≥ 1.5 x 10E9/L  – < LLN | ≥ 1.0 -  < 1.5 x 10E9/L | ≥ 0.5 - < 1.0 x 10E9/L | < 0.5 x 10E9/L | n/a |
| Absolute lymphocytes count decreased | Absolute lymphocytes count (x 10E9/L) | ≥ LLN | ≥ 0.8 x 10E9/L  – < LLN | ≥ 0.5 -  < 0.8 x 10E9/L | ≥ 0.2 -  < 0.5 x 10E9/L | < 0.2 x 10E9/L | n/a |
| Absolute lymphocytes count increased | Absolute lymphocytes count (x 10E9/L) | ≤ 4 x 10E9/L | n/a | > 4 –  ≤ 20 x 10E9/L | > 20 x 10E9/L | n/a | n/a |
| Eosinophilia | Absolute eosinophils | ≤ ULN or  ≤ Baseline | > ULN and  > Baseline | n/a | n/a | n/a | n/a |
| Fibrinogen decreased | Fibrinogen (g/L) | ≥ LLN if baseline normal;  no decrease from baseline if baseline abnormal | ≥ 0.75 -  < 1 x LLN if baseline normal;  > 0 - < 25%  decrease from baseline if baseline abnormal | ≥ 0.5 -  < 0.75 x LLN  if baseline normal;  ≥ 25 - < 50%  decrease from baseline if baseline abnormal | ≥ 0.25 -  < 0.5 x LLN if baseline normal;  ≥ 50 - < 75%  decrease from baseline if baseline abnormal | < 0.25 x LLN if  baseline normal;  ≥ 75% decrease from baseline if baseline abnormal |  |

Document: \\ieedc-vnasc01\BIOSdata\Jiangsu_PMBP\Meplazumab\SZA62090\Biostatistics\Documentation\SAP\

| Author: Yueh Wang, Yang Teng | Version Number: Version Date: | 2.0  13-Dec-2021 |
| --- | --- | --- |
| Template No.: CS_TP_BS016 Revision 6 Effective Date: 02Dec2019 | Reference: | CS_WI_BS005 |

Copyright © 2009, 2010, 2012, 2016, 2018, 2019 IQVIA. All rights reserved. The contents of this document are confidential and proprietary to IQVIA Holdings Inc. and its subsidiaries. Unauthorized use, disclosure or reproduction is strictly prohibited.


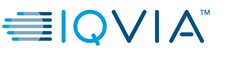
Jiangsu Pacific Meinuoke Biopharmaceutical Co., Ltd. (PMBP)

Protocol MPZ-II-02

Statistical Analysis Plan Page 77 of 82

| **CTCAE Term** | **Laboratory Test** | **No Event** | **Grade 1** | **Grade 2** | **Grade 3** | **Grade 4** | **Grade 5** |
| --- | --- | --- | --- | --- | --- | --- | --- |
| Hypernatremia | Sodium (mmol/L) | ≤ ULN | > ULN – ≤ 150  mmol/L | > 150 –  ≤ 155 mmol/L | > 155 –  ≤ 160 mmol/L | > 160 mmol/L | n/a |
| Hyponatremia | Sodium (mmol/L) | ≥ LLN | ≥ 130 mmol/L  – < LLN | ≥ 125 -  < 130 mmol/L | ≥ 120 -  < 125 mmol/L | < 120 mmol/L | n/a |
| Hyperkalemia | Potassium (mmol/L) | ≤ ULN | > ULN –  ≤ 5.5 mmol/L | > 5.5 –  ≤ 6.0 mmol/L | > 6.0 –  ≤ 7.0 mmol/L | > 7.0 mmol/L | n/a |
| Hypokalemia | Potassium (mmol/L) | ≥ LLN | ≥ 3.0 mmol/L –  < LLN | n/a | ≥ 2.5 -  < 3.0 mmol/L | < 2.5 mmol/L | n/a |
| Hypercalcemia | Ionized calcium (mmol/L) | ≤ ULN | > ULN –  ≤ 1.5 mmol/L | > 1.5 –  ≤ 1.6 mmol/L | > 1.6 –  ≤ 1.8 mmol/L | > 1.8 mmol/L | n/a |
| Hypocalcemia | Ionized calcium (mmol/L) | ≥ LLN | ≥ 1.0 mmol/L –  < LLN | ≥ 0.9 -  < 1.0 mmol/L | ≥ 0.8 -  < 0.9 mmol/L | < 0.8 mmol/L | n/a |
| Hypermagnesemia | Magnesium (mmol/L) | ≤ ULN | > ULN –  ≤ 1.23 mmol/L | n/a | > 1.23 –  ≤ 3.30 mmol/L | > 3.30 mmol/L | n/a |
| Hypomagnesemia | Magnesium (mmol/L) | ≥ LLN | ≥ 0.5 mmol/L –  < LLN | ≥ 0.4 -  < 0.5 mmol/L | ≥ 0.3 -  < 0.4 mmol/L | < 0.3 mmol/L | n/a |

Document: \\ieedc-vnasc01\BIOSdata\Jiangsu_PMBP\Meplazumab\SZA62090\Biostatistics\Documentation\SAP\

| Author: Yueh Wang, Yang Teng | Version Number: Version Date: | 2.0  13-Dec-2021 |
| --- | --- | --- |
| Template No.: CS_TP_BS016 Revision 6 Effective Date: 02Dec2019 | Reference: | CS_WI_BS005 |

Copyright © 2009, 2010, 2012, 2016, 2018, 2019 IQVIA. All rights reserved. The contents of this document are confidential and proprietary to IQVIA Holdings Inc. and its subsidiaries. Unauthorized use, disclosure or reproduction is strictly prohibited.


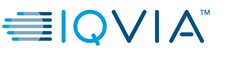
Jiangsu Pacific Meinuoke Biopharmaceutical Co., Ltd. (PMBP)

Protocol MPZ-II-02

Statistical Analysis Plan Page 78 of 82

| **CTCAE Term** | **Laboratory Test** | **No Event** | **Grade 1** | **Grade 2** | **Grade 3** | **Grade 4** | **Grade 5** |
| --- | --- | --- | --- | --- | --- | --- | --- |
| Hypoglycemia | Glucose (mmol/L) | ≥ LLN | ≥ 3.0 mmol/L –  < LLN | ≥ 2.2 -  < 3.0 mmol/L | ≥ 1.7 -  < 2.2 mmol/L | < 1.7 mmol/L | n/a |
| Creatinine increased | Creatinine (µmol/L) | ≤ ULN | > ULN – | > 1.5 – | > 3.0 – | > 6.0 x ULN | n/a |
|  |  |  | ≤ 1.5 x ULN | ≤ 3.0 x ULN | ≤ 6.0 x ULN |  |  |
|  |  |  |  | **or** | **or** |  |  |
|  |  |  |  | > 1.5 – ≤3.0 x | > 3.0 x baseline |  |  |
|  |  |  |  | baseline |  |  |  |
| Alkaline phosphatase | ALP (U/L) | ≤ ULN if | > ULN – | > 2.5 – | > 5.0 – | > 20.0 x ULN if | n/a |
| (ALP) increased |  | baseline | ≤ 2.5 x ULN if | ≤ 5.0 x ULN if | ≤ 20.0 x ULN if | baseline |  |
|  |  | normal; | baseline | baseline | baseline | normal; |  |
|  |  | ≤ 2.0 x | normal; | normal; | normal; | > 20.0 x |  |
|  |  | baseline if | > 2.0 - | > 2.5 – | > 5.0 – ≤ 20.0 x | baseline if |  |
|  |  | baseline | ≤ 2.5 x baseline | ≤ 5.0 x baseline | baseline if | baseline |  |
|  |  | abnormal | if baseline | if baseline | baseline | abnormal |  |
|  |  |  | abnormal | abnormal | abnormal |  |  |

Document: \\ieedc-vnasc01\BIOSdata\Jiangsu_PMBP\Meplazumab\SZA62090\Biostatistics\Documentation\SAP\

| Author: Yueh Wang, Yang Teng | Version Number: Version Date: | 2.0  13-Dec-2021 |
| --- | --- | --- |
| Template No.: CS_TP_BS016 Revision 6 Effective Date: 02Dec2019 | Reference: | CS_WI_BS005 |

Copyright © 2009, 2010, 2012, 2016, 2018, 2019 IQVIA. All rights reserved. The contents of this document are confidential and proprietary to IQVIA Holdings Inc. and its subsidiaries. Unauthorized use, disclosure or reproduction is strictly prohibited.


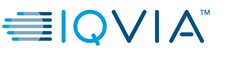
Jiangsu Pacific Meinuoke Biopharmaceutical Co., Ltd. (PMBP)

Protocol MPZ-II-02

Statistical Analysis Plan Page 79 of 82

| **CTCAE Term** | **Laboratory Test** | **No Event** | **Grade 1** | **Grade 2** | **Grade 3** | **Grade 4** | **Grade 5** |
| --- | --- | --- | --- | --- | --- | --- | --- |
| Alanine transaminase | ALT (U/L) | ≤ ULN if | > ULN – | > 3.0 – | > 5.0 – ≤ 20.0 x | > 20.0 x ULN if | n/a |
| (ALT) increased |  | baseline | ≤ 3.0 x ULN if | ≤ 5.0 x ULN if | ULN if baseline | baseline |  |
|  |  | normal; | baseline | baseline | normal; | normal; |  |
|  |  | ≤ 1.5 x | normal; | normal; | > 5.0 – ≤ 20.0 x | > 20.0 x |  |
|  |  | baseline if | > 1.5 - ≤ 3.0 x | > 3.0 – ≤ 5.0 x | baseline if | baseline if |  |
|  |  | baseline | baseline if | baseline if | baseline | baseline |  |
|  |  | abnormal | baseline | baseline | abnormal | abnormal |  |
|  |  |  | abnormal | abnormal |  |  |  |
| Aspartate transaminase | AST (U/L) | ≤ ULN if | > ULN – | > 3.0 – | > 5.0 – ≤ 20.0 x | > 20.0 x ULN if | n/a |
| (AST) increased |  | baseline | ≤ 3.0 x ULN if | ≤ 5.0 x ULN if | ULN if baseline | baseline |  |
|  |  | normal; | baseline | baseline | normal; | normal; |  |
|  |  | ≤ 1.5 x | normal; | normal; | > 5.0 – ≤ 20.0 x | > 20.0 x |  |
|  |  | baseline if | > 1.5 - ≤ 3.0 x | > 3.0 – ≤ 5.0 x | baseline if | baseline if |  |
|  |  | baseline | baseline if | baseline if | baseline | baseline |  |
|  |  | abnormal | baseline | baseline | abnormal | abnormal |  |
|  |  |  | abnormal | abnormal |  |  |  |

Document: \\ieedc-vnasc01\BIOSdata\Jiangsu_PMBP\Meplazumab\SZA62090\Biostatistics\Documentation\SAP\

| Author: Yueh Wang, Yang Teng | Version Number: Version Date: | 2.0  13-Dec-2021 |
| --- | --- | --- |
| Template No.: CS_TP_BS016 Revision 6 Effective Date: 02Dec2019 | Reference: | CS_WI_BS005 |

Copyright © 2009, 2010, 2012, 2016, 2018, 2019 IQVIA. All rights reserved. The contents of this document are confidential and proprietary to IQVIA Holdings Inc. and its subsidiaries. Unauthorized use, disclosure or reproduction is strictly prohibited.


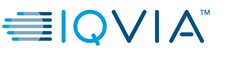
Jiangsu Pacific Meinuoke Biopharmaceutical Co., Ltd. (PMBP)

Protocol MPZ-II-02

Statistical Analysis Plan Page 80 of 82

| **CTCAE Term** | **Laboratory Test** | **No Event** | **Grade 1** | **Grade 2** | **Grade 3** | **Grade 4** | **Grade 5** |
| --- | --- | --- | --- | --- | --- | --- | --- |
| Blood bilirubin | Total bilirubin | ≤ ULN if | > ULN – | > 1.5 – | > 3.0 – ≤ 10.0 x | > 10.0 x ULN if | n/a |
| increased | (µmol/L) | baseline | ≤ 1.5 x ULN if | ≤ 3.0 x ULN if | ULN if baseline | baseline |  |
|  |  | normal; | baseline | baseline | normal; | normal; |  |
|  |  | ≤ baseline if | normal; | normal; | > 3.0 - ≤ 10.0 x | > 10.0 x |  |
|  |  | baseline | > baseline - ≤ | > 1.5 - ≤ 3.0 x | baseline if | baseline if |  |
|  |  | abnormal | 1.5 x baseline | baseline if | baseline | baseline |  |
|  |  |  | if baseline | baseline | abnormal | abnormal |  |
|  |  |  | abnormal | abnormal |  |  |  |
| Gamma glutamyl | GGT (U/L) | ≤ ULN if | > ULN – | > 2.5 – | > 5.0 – ≤ 20.0 x | > 20.0 x ULN if | n/a |
| transferase (GGT) |  | baseline | ≤ 2.5x ULN if | ≤ 5.0 x ULN if | ULN if baseline | baseline |  |
| increased |  | normal; | baseline | baseline | normal; | normal; |  |
|  |  | ≤ 2.0 x | normal; | normal; | > 5.0 - ≤ 20.0 x | > 20.0 x |  |
|  |  | baseline if | > 2.0 - | > 2.5 - | baseline if | baseline if |  |
|  |  | baseline | ≤ 2.5 x baseline | ≤ 5.0 x baseline | baseline | baseline |  |
|  |  | abnormal | if baseline | if baseline | abnormal | abnormal |  |
|  |  |  | abnormal | abnormal |  |  |  |
| Blood bicarbonate decreased | Bicarbonate (mmol/L) | ≥ LLN | < LLN | n/a | n/a | n/a | n/a |

Document: \\ieedc-vnasc01\BIOSdata\Jiangsu_PMBP\Meplazumab\SZA62090\Biostatistics\Documentation\SAP\

| Author: Yueh Wang, Yang Teng | Version Number: Version Date: | 2.0  13-Dec-2021 |
| --- | --- | --- |
| Template No.: CS_TP_BS016 Revision 6 Effective Date: 02Dec2019 | Reference: | CS_WI_BS005 |

Copyright © 2009, 2010, 2012, 2016, 2018, 2019 IQVIA. All rights reserved. The contents of this document are confidential and proprietary to IQVIA Holdings Inc. and its subsidiaries. Unauthorized use, disclosure or reproduction is strictly prohibited.


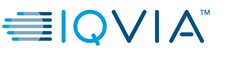
Jiangsu Pacific Meinuoke Biopharmaceutical Co., Ltd. (PMBP)

Protocol MPZ-II-02

Statistical Analysis Plan Page 81 of 82

| **CTCAE Term** | **Laboratory Test** | **No Event** | **Grade 1** | **Grade 2** | **Grade 3** | **Grade 4** | **Grade 5** |
| --- | --- | --- | --- | --- | --- | --- | --- |
| Hypoalbuminemia | Albumin (g/L) | ≥ LLN | ≥ 30 g/L - < LLN | ≥ 20 - < 30 g/L | < 20 g/L | n/a | n/a |
| CPK increased | Creatine kinase (U/L) | ≤ ULN | > ULN –  ≤ 2.5 x ULN | > 2.5 –  ≤ 5 x ULN | > 5 –  ≤ 10 x ULN | > 10 x ULN | n/a |
| Cholesterol high | Total cholesterol (mmol/L) | ≤ ULN | > ULN –  ≤ 7.75 mmol/L | > 7.75 -  ≤ 10.34  mmol/L | > 10.34 -  ≤ 12.92 mmol/L | > 12.92 mmol/L | n/a |
| Hypertriglyceridemia | Triglycerides (mmol/L) | ≤ 1.71 mmol/L | > 1.71 –  ≤ 3.42 mmol/L | > 3.42 -  ≤ 5.70 mmol/L | > 5.70 -  ≤ 11.40 mmol/L | > 11.40 mmol/L | n/a |
| Chronic kidney disease | eGRF (mL/min/1.73 m2) | ≥ LLN | ≥ 60 mL/min/1.73 m2 - < LLN | ≥ 30 - < 60  mL/min/1.73 m2 | ≥ 15 - < 30  mL/min/1.73 m2 | < 15  mL/min/1.73 m2 | n/a |

Document: \\ieedc-vnasc01\BIOSdata\Jiangsu_PMBP\Meplazumab\SZA62090\Biostatistics\Documentation\SAP\

| Author: Yueh Wang, Yang Teng | Version Number: Version Date: | 2.0  13-Dec-2021 |
| --- | --- | --- |
| Template No.: CS_TP_BS016 Revision 6 Effective Date: 02Dec2019 | Reference: | CS_WI_BS005 |

Copyright © 2009, 2010, 2012, 2016, 2018, 2019 IQVIA. All rights reserved. The contents of this document are confidential and proprietary to IQVIA Holdings Inc. and its subsidiaries. Unauthorized use, disclosure or reproduction is strictly prohibited.


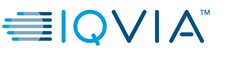
Jiangsu Pacific Meinuoke Biopharmaceutical Co., Ltd. (PMBP)

Protocol MPZ-II-02

Statistical Analysis Plan Page 82 of 82

| **CTCAE Term** | **Laboratory Test** | **No Event** | **Grade 1** | **Grade 2** | **Grade 3** | **Grade 4** | **Grade 5** |
| --- | --- | --- | --- | --- | --- | --- | --- |
| International normalized | INR | ≤ 1.2 if not on | > 1.2 – ≤1.5 if | > 1.5 – ≤2.5 if | > 2.5 if not on | n/a | n/a |
| ratio (INR) increased |  | anticoagulant; | not on | not on | anticoagulant; |  |  |
|  |  | ≤ baseline if | anticoagulant; | anticoagulant; | > 2.5 x baseline |  |  |
|  |  | on | > baseline - | > 1.5 - ≤ 2.5 x | if on |  |  |
|  |  | anticoagulant | ≤ 1.5 x baseline | baseline if on | anticoagulant |  |  |
|  |  |  | if on | anticoagulant |  |  |  |
|  |  |  | anticoagulant |  |  |  |  |

Document: \\ieedc-vnasc01\BIOSdata\Jiangsu_PMBP\Meplazumab\SZA62090\Biostatistics\Documentation\SAP\

| Author: Yueh Wang, Yang Teng | Version Number: Version Date: | 2.0  13-Dec-2021 |
| --- | --- | --- |
| Template No.: CS_TP_BS016 Revision 6 Effective Date: 02Dec2019 | Reference: | CS_WI_BS005 |

Copyright © 2009, 2010, 2012, 2016, 2018, 2019 IQVIA. All rights reserved. The contents of this document are confidential and proprietary to IQVIA Holdings Inc. and its subsidiaries. Unauthorized use, disclosure or reproduction is strictly prohibited.
