# Supplementary material for: Meplazumab in hospitalized adults with severe COVID-19 (DEFLECT): a multicenter, seamless phase 2/3, randomized, third-party double-blind clinical trial
Source: Signal Transduct Target Ther. 2023 Jan 30;8:46. doi: 10.1038/s41392-023-01323-9 (PMC9885411; doi:10.1038/s41392-023-01323-9)
Supplement: Supplementary file 4 — Sigtrans_Supplementary_Note_3 [file 41392_2023_1323_MOESM4_ESM.docx]

STATISTICAL ANALYSIS PLAN

| **Protocol Title:** | A Multicenter, Seamless, Randomized, Third-Party-Blind Clinical Trial to Evaluate the Safety and Efficacy of Meplazumab in Addition to Standard of Care for the Treatment of COVID-19 in Hospitalized Adults |
| --- | --- |
| **Protocol Number:** | MPZ-II-02 |
| **Phase:** | Phase Ⅱ/Ⅲ |
| **Sponsor:** | Jiangsu Pacific Meinuoke Biopharmaceutical Co., Ltd. (PMBP) |
| **Author:** | Jiangsu Pacific Meinuoke Biopharmaceutical Co., Ltd. (“PMBP”), having a place of business at No. 128 W Hehai Rd, Xinbei District, Changzhou, Jiangsu Province, China |
| **SAP Date:** | 2022-04-20 |
| **Status:** | V1.0 |

| **Statistical Analysis Plan Approval Form** | | | |
| --- | --- | --- | --- |
|  | |  | |
|  | |  | |
| **Sponsor:** | | Jiangsu Pacific Meinuoke Biopharmaceutical Co., Ltd. (PMBP) | |
| **Protocol:** | | MPZ-II-02 | |
| **Protocol Title:** | | A Multicenter, Seamless, Randomized, Third-Party-Blind Clinical Trial to Evaluate the Safety and Efficacy of Meplazumab in Addition to Standard of Care for the Treatment of COVID-19 in Hospitalized Adults | |
| **SAP Version:** | | V1.0 | |
| **SAP Date:** | | 2022-04-20 | |
| The statistical analysis plan has been reviewed and approved. | | | |
| **Signed:** | *Hongwei Shi* | |  |
|  | *Sponsor Representative* | |  |
|  | *Jiangsu Pacific Meinuoke Biopharmaceutical Co., Ltd.* | |  |
|  | _______________________________________ | | ________________________ |
|  | Signature | | Date |
|  |  | |  |
|  |  | |  |
|  |  | |  |
| **Signed:** | *Ye Tian Fen Chi* | |  |
|  | *Statistician* | |  |
|  | *Beijing KeyTech Statistical Consulting Co., Ltd* | |  |
|  | _______________________________________ | | ________________________ |
|  | Signature | | Date |
|  |  | |  |
|  |  | |  |
|  |  | |  |
| **Signed:** | *Zhiwei Jiang, PhD* | |  |
|  | *General Manager* | |  |
|  | *Beijing KeyTech Statistical Consulting Co., Ltd* | |  |
|  | _______________________________________ | | ________________________ |
|  | Signature | | Date |

# TABLE OF CONTENTS

[TABLE OF CONTENTS 3](#_Toc107936778)

[1. Introduction 4](#_Toc107936779)

[2. Study endpoints 4](#_Toc107936780)

[2.1 Viral Load 4](#_Toc107936781)

[2.2 Cytokines/ Chemokines /Inflammatory factor 4](#_Toc107936782)

[3. Analysis Populations 4](#_Toc107936783)

[4. Statistical Analysis methods 4](#_Toc107936784)

[4.1 General Considerations 4](#_Toc107936785)

[4.2 Statistical Analysis 5](#_Toc107936786)

[4.2.1 Viral Load 5](#_Toc107936787)

[4.2.2 Cytokines 6](#_Toc107936788)

[4.3 Missing Data 6](#_Toc107936789)

[Version History 7](#_Toc107936790)

# 1. Introduction

This document is a Statistical Analysis Plan (SAP) for viral load and cytokines in A Multicenter, Seamless, Randomized, Third-Party-Blind Clinical Trial to Evaluate the Safety and Efficacy of Meplazumab in Addition to Standard of Care for the Treatment of COVID-19 in Hospitalized Adults, specific statistical analysis methods for viral load and cytokines used for analysis and reporting are described. The relevant statistical analysis results of this study will be used for the registration and application of this product.

# 2. Study endpoints

## 2.1 Viral Load

## 2.2 Cytokines/ Chemokines /Inflammatory factor

- IL-2, IL-4, IL-6, IL-7, IL-8, IL-10, IL-12p70, IL-15, IL-17A, IL-1RA, IL-2Rα, MCP-1, MIP-1β, IP-10, TNFα, IFN-γ

# 3. Analysis Populations

- The analysis for viral load will be based on people with positive results at baseline.
- The analysis for cytokines will be based on people with detection results of cytokine data.

# 4. Statistical Analysis methods

## 4.1 General Considerations

- **Descriptive Statistics**

Unless otherwise specified, the following summary of descriptive statistics will be given by variable type.

- Standard descriptive statistics, such as mean, standard deviation, minimum, and maximum, will be calculated for continuous variables.
- For categorical or ordered variables, descriptive analyses will be based on numbers of subjects and related percentages, the percentage calculation will be based on the number of non-empty subjects in the corresponding analysis set. The 95% confidence interval of percentage will be calculated according to Clopper-Pearson.
- **Decimal Places**

Unless otherwise specified, the decimal places in the statistical analysis report will follow the following rules:

- The minimum and maximum values are consistent with the maximum decimal number of the original data;
- The decimal places of median, mean, standard deviation, and 95% confidence interval are one more than the maximum decimal number of the original data;
- Retain percentages to 2 decimal places;
- Derived data is reserved to 2 decimal places.
- **Baseline**

Unless otherwise specified, baseline will be defined as the last non-missing value prior to the first treatment.

- **Visit**

For post-baseline visits, statistical analysis will be performed at scheduled visits, when analysed by visit. All inspection results, including scheduled visits and unscheduled visits, will be listed.

- **Tables**

This study will be summarized by group (0.12mg/kg, 0.2mg/kg, 0.3mg/kg and placebo). Analysis groups will generally be presented in columns.

- **Listings**

Unless otherwise specified, all listings will include groups and subject numbers, and will preferentially present raw data in SDTM.

- **Baseline**

All statistical analyses will be generated by using SAS^®^ Version 9.4.

## 4.2 Statistical Analysis

For both viral load and cytokines, analysis results will be based on values in logarithmic form.

**4.2.1 Viral Load**

Observed and change from baseline values on Day 3, Day 5, Day 8, Day 9, Day 10, Day 29 after initial treatment will be statistically described by each group, and difference among groups will be statistically tested according to logarithmic converted ANOVA. Meanwhile, the logarithmic converted group t-test will be used to statistically examine the difference between the two groups.

Qualitative results of viral load on Day 3, Day 5, Day 8, Day 9, Day 10, Day 29 after initial treatment will be summarized by each group, and difference among groups will be statistically tested according to Chi-square Test or Fisher's exact probability method.

Kaplan-Meier method will be used to calculate negative conversion rate of coronavirus and the corresponding 95% confidence interval (Greenwood method: log-log transformation) for each group on Day 3, Day 5, Day 8, Day 9, Day 10, Day 29 after initial treatment. Kaplan-Meier method will be used to estimate the lower quartile, median and upper quartile of negative conversion time and the corresponding 95% confidence intervals (Brookmeyer-Crowley method: log-log transformation). Meanwhile, Cox proportional hazard regression model will be used to estimate hazard ratio and the corresponding 95% confidence interval between two groups with negative conversion time of coronavirus as the dependent variable, group as the fixed effect and subjects as the random effect.

Viral negative conversion will be defined as the time from a subject with a positive viral test result at baseline to the last negative viral test result after initial treatment. Meanwhile, sensitivity analysis will be performed under the circumstances that people who turned positive again will be regarded as positive.

Viral negative conversion time will be calculated by below formula:

Viral negative conversion time (days) = date of first negative conversion – date of baseline viral load test + 1 day.

The censoring rule for viral negative conversion time is as follows: if the subject does not turn negative or complete this study or withdraw from this study after initial treatment, the last viral load examination date will be regarded as censoring date.

Draw the Kaplan-Meier curve of viral negative conversion time for each group.

**4.2.2 Cytokines**

Observed and change from baseline values on Day 2, Day 8, Day 9, Day 29 after initial treatment will be statistically described by each group, and difference among groups will be statistically tested according to logarithmic converted ANOVA. Meanwhile, the logarithmic converted group t-test will be used to statistically examine the difference between the two groups.

## 4.3 Missing Data

The treatment rules for those endpoints with missing viral load and cytokine data are shown in Table 4.1.

**Table 4.1 Treatment Rules for Missing Data**

| **Endpoints** | **Indicator** | **Treatment Rules** |
| --- | --- | --- |
| Viral load | Observed and change from baseline viral load values on Day 3, Day 5, Day 8, Day 9, Day 10, Day 29 after initial treatment | LOCF |
| Cytokines | Observed and change from baseline cytokines values on Day 2, Day 8, Day 9, Day 29 after initial treatment | LOCF |

**Version History**

| **Version Number** | **Version Date** | **Description of Change** |
| --- | --- | --- |
| V1.0 | 2022-04-20 | Initial Version |
